# The stereodivergent formation of 2,6-*cis* and 2,6-*trans*-tetrahydropyrans: experimental and computational investigation of the mechanism of a thioester oxy-Michael cyclization†
†Electronic supplementary information (ESI) available: Experimental procedures, compound characterization data and details of the computational studies. See DOI: 10.1039/c6sc03478k



**DOI:** 10.1039/c6sc03478k

**Published:** 2016-08-30

**Authors:** Kristaps Ermanis, Yin-Ting Hsiao, Uğur Kaya, Alan Jeuken, Paul A. Clarke

**Affiliations:** a Department of Chemistry , University of York , Heslington , York , North Yorkshire YO10 5DD , UK . Email: paul.clarke@york.ac.uk

## Abstract

The origins of the stereodivergence in the thioester oxy-Michael cyclization for the formation of 4-hydroxy-2,6-*cis*- or 2,6-*trans*-substituted tetrahydropyran rings under different conditions was investigated both computationally and experimentally. Synthetic studies showed that the 4-hydroxyl group was essential for stereodivergence. When the 4-hydroxyl group was present, TBAF-mediated conditions gave the 2,6-*trans*-tetrahydropyran and trifluoroacetic acid-mediated conditions gave the 2,6-*cis*-tetrahydropyran. This stereodivergence vanished when the hydroxyl group was removed or protected. Computational studies revealed that: (i) the trifluoroacetic acid catalysed formation of 2,6-*cis*-tetrahydropyrans was mediated by a trifluoroacetate-hydroxonium bridge and proceeded *via* a chair-like transition state; (ii) the TBAF-mediated formation of 2,6-*trans*-tetrahydropyrans proceeded *via* a boat-like transition state, where the 4-hydroxyl group formed a crucial hydrogen bond to the cyclizing alkoxide; (iii) both reactions are under kinetic control. The utility of this stereodivergent approach for the formation of 4-hydroxy-2,6-substituted tetrahydropyran rings has been demonstrated by the total syntheses of the anti-osteoporotic natural products diospongin A and B.

## Abstract 2

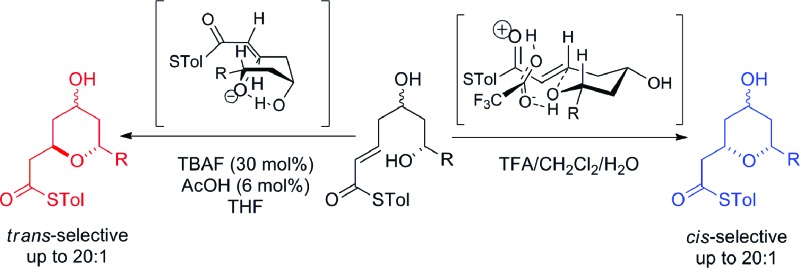
Computational and synthetic studies have elucidated the origins of stereodivergence in an oxy-Michael synthesis of 2,6-disubstituted tetrahydropyrans.

## Introduction

**Scheme 1:**
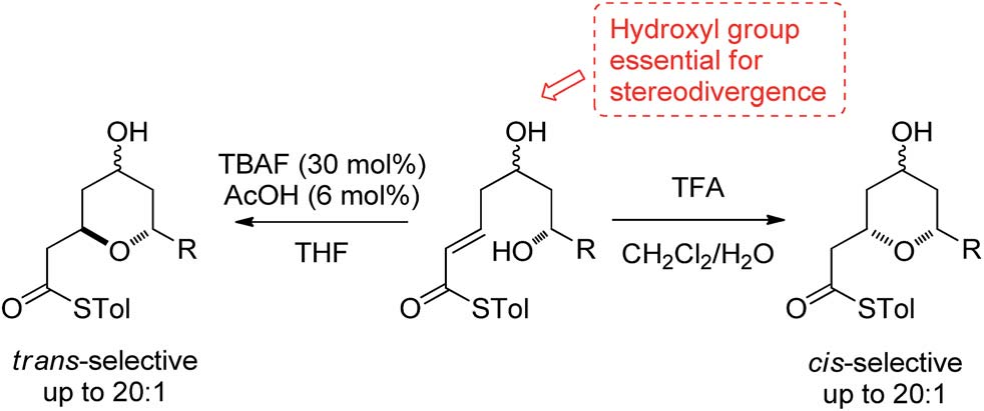
The stereodivergent thioester oxy-Michael cyclization.

2,6-Disubstituted tetrahydropyran (THP) rings form key structural motifs in many potent biologically active natural products,^1^ including the phorboxazoles,^2^ lasonolide A,^3^ the diospongins^4^ and psymberin.^5^ These biological activities and complex molecular frameworks have prompted a large amount of work aimed at increasing the efficiency of the syntheses of THP rings,^6^ which, problematically, are often formed as mixtures of 2,6-*cis*- and 2,6-*trans*-diastereomers. One fundamental strategy regularly used for their formation is the oxy-Michael cyclization onto an α,β-unsaturated carbonyl group, which often leads to the formation of both possible diastereomeric THPs. Here, we report a stereodivergent oxy-Michael reaction which can lead to the diastereoselective formation of either the 2,6-*cis*- or the 2,6-*trans*-THP in up to 20 : 1 diastereoselectivity (Scheme 1). We have also conducted computational and experimental studies which elucidate the origin of this stereodivergence and show the importance of a H-bond between the 4-hydroxyl group and the cyclizing alkoxide in the oxy-Michael cyclization. These studies allow us to propose a general set of guidelines for future syntheses of 2,6-disubstituted THPs.

Previous studies have investigated oxy-Michael cyclizations in order to gain an understanding of the factors governing the stereoselectivity of the cyclization. This has led to the general opinion that the formation of the 2,6-*cis*-THP may be favored by performing an oxy-Michael reaction onto an α,β-unsaturated ester under thermodynamic conditions, while the 2,6-*trans*-THP may be favored by performing the same reaction under kinetically controlled conditions. In practice, the situation is not so straightforward. While 2,6-*trans*-THPs tend to be formed in good yields with moderate to good diastereoselectivities,^11^ the higher temperatures and longer reaction times required for the formation of the 2,6-*cis*-THP ring often result in lower diastereoselectivities and yields.^12^ The origin of this moderate 2,6-*trans*-selectivity is generally accepted as arising from better orbital overlap in the transition state of the kinetic cyclization leading to the 2,6-*trans*-THP compared to the 2,6-*cis*-THP.^8^ Although there is no generally accepted mechanism for the acid-mediated cyclization, it has been proposed that the formation of the 2,6-*cis*-THP is favored due to greater stereoelectronic stabilization of the transition state from both the FMO coefficients of the allylic cation and orbital overlap with the oxygen lone pair, compared to the transition state leading to the 2,6-*trans*-THP.^9^

**Scheme 2:**
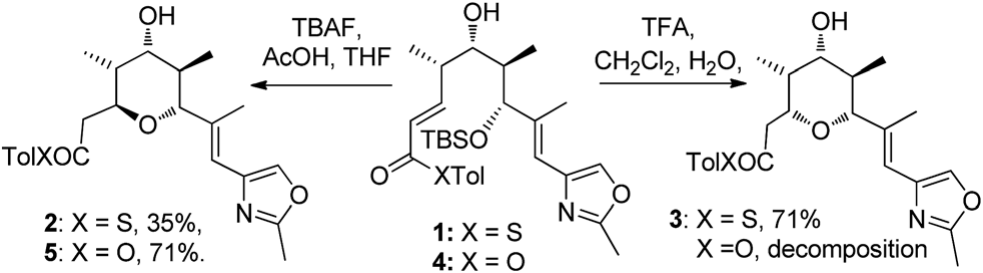
Stereodivergence in the thioester oxy-Michael cyclization to form the C20–C32 fragment of the phorboxazoles.

In our studies on the synthesis of the C20–C32 pentasubstituted tetrahydropyran core of the phorboxazoles we encountered an occurrence of stereodivergence^13^ while utilizing thioesters as electophiles in an oxy-Michael cyclization.^14^ In this case, stereodivergence occurred when the conditions for the deprotection of a TBS-ether were changed. Deprotection of **1** with AcOH buffered TBAF led to the formation of the 2,6-*trans*-THP **2** with no trace of **3** being detected. However, deprotection with TFA resulted in the formation of the 2,6-*cis*-THP product **3** in >13 : 1 selectivity (Scheme 2). It is worth noting that when the conventional oxoester **4** was submitted to these conditions the 2,6-*trans*-THP **5** resulted from treatment with TBAF buffered with AcOH in THF; no trace of the *cis*-diastereomer was seen. However, only decomposition occurred when **4** was treated with TFA.^13^ Intrigued by these results, especially by the dramatic change in diastereoselectivity seen in the thioester substrate, we resolved to carry out synthetic and computational studies to elucidate the mechanistic origins of this stereodivergence and to establish more general synthetic guidelines for the diastereoselective synthesis of 2,6-disubstituted THPs. The results of these studies are reported in this paper.

## Results and discussion

### Synthetic investigation into the generality of stereodivergence

We initially decided to probe whether the stereodivergence was specific to **1** or whether it was a more general phenomenon. To this end we synthesized cyclization substrates **6a–c,** which had the same relative configuration as **1** and **9a–c**, which had the opposite relative configuration.^15^ Each substrate was submitted to both the buffered TBAF and the TFA mediated conditions (Tables 1 and 2).

**Table 1:** Stereodivergent oxy-Michael cyclizations of 4-hydroxyl-containing substrates **6a**, **b** and **c**

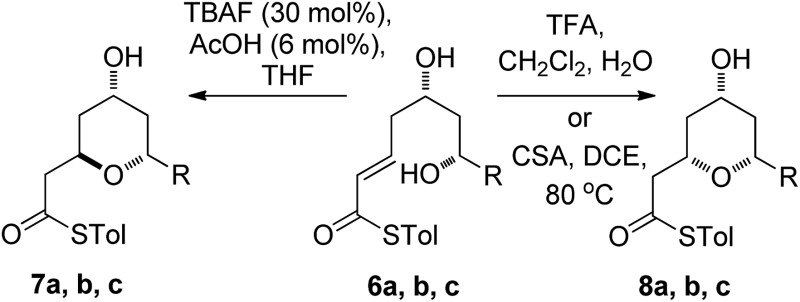					
Entry	Ratio *cis* : *trans* ^*a*^	TBAF yield^*b*^ (%)	R	TFA/CSA^*b*^ yield (%)	Ratio *cis* : *trans* ^*a*^
a	1 : 7	69	iPr	66^*c*^	20 : 1
b	1 : 6	40	Ph	74^*d*^	10 : 1
c	2 : 3	41	C_7_H_14_	47^*c*^	20 : 1

^*a*^Ratios obtained from integration of ^1^H NMR signals.

^*b*^Isolated yields after chromatography.

^*c*^TFA, CH_2_Cl_2_, H_2_O.

^*d*^CSA, DCE, 80 °C.

**Table 2:** Stereodivergent oxy-Michael cyclizations of 4-hydroxyl-containing substrates **9a**, **b** and **c**

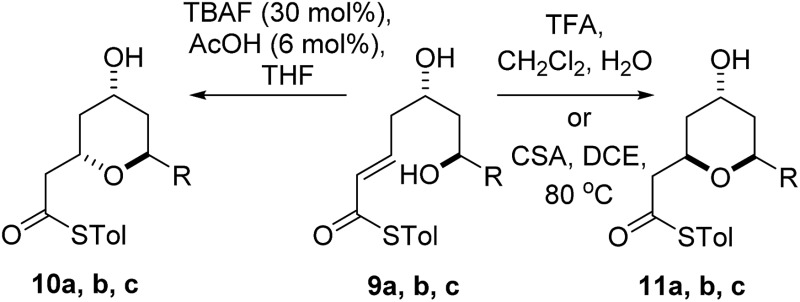					
Entry	Ratio *cis* : *trans* ^*a*^	TBAF yield^*b*^ (%)	R	TFA/CSA yield^*b*^ (%)	Ratio *cis* : *trans* ^*a*^
a	1 : 8	69	iPr	66^*c*^	20 : 1
b	1 : 20	40	Ph	74^*d*^	7 : 1
c	1 : 20	48	C_7_H_15_	65^*c*^	20 : 1

^*a*^Ratios obtained from integration of ^1^H NMR signals.

^*b*^Isolated yields after chromatography.

^*c*^TFA, CH_2_Cl_2_, H_2_O.

^*d*^CSA, DCE, 80 °C.

Substrates **6a**, **b**, **c**, which contain the 4-hydroxyl group were submitted to both the TBAF-mediated and the Brønsted acid promoted cyclization conditions (Table 1). In this case, TBAF mediated reactions smoothly generated 2,6-*trans*-THP products **7a–c** in good yields and with excellent selectivity (with the exception of **6c**), while Brønsted acid promoted conditions gave the 2,6-*cis*-THP products **8a–c** with excellent selectivity and in good yields. In the case of the phenyl substituted compound **6b**, the TFA conditions led to decomposition although the CSA conditions led to 2,6-*cis*-THP product being isolated in 74% yield.

Diastereomeric diol substrates **9a**, **b**, **c** were studied next (Table 2). Once again, TBAF-mediated cyclizations generated the 2,6-*trans*-THP predominantly. As before, Brønsted acid promoted conditions gave the 2,6-*cis*-THP products **11a–c** with excellent selectivity and in good yields. In the case of **9b** (R = Ph), CSA promoted conditions had to be used to avoid decomposition under TFA conditions. Thus it would appear that the stereodivergence seen in the cyclization of **1** is not limited to that particular system.

In order to ascertain if the reactions were under thermodynamic control 2,6-*cis*-substrate **11b** was submitted to the TBAF conditions and found to be unchanged after several hours and 2,6-*trans*-substrate **10b** was submitted to the Brønsted acid conditions and was also re-isolated unchanged. These results imply that both the TBAF and TFA-mediated reactions are *not* under thermodynamic control.

### Computational studies on the stereodivergence

With stereodivergent behavior being exhibited by all the substrates investigated, we decided to conduct DFT studies in order to elucidate the origin of this behaviour. DFT investigations were conducted on both the buffered TBAF-mediated reaction which produced the 2,6-*trans*-THPs **2**, **7a–c** and **10a–c** and the TFA-mediated reactions which produced 2,6-*cis*-THPs **3**, **8a–c** and **11a–c**. Conformation searches were conducted at the molecular mechanics level and using MacroModel and MMFF force field.^16^ DFT geometries were optimized and energies calculated using the B3LYP density functional,^17^ and split-valence polarized 6-31G*+ basis set with diffuse functions.^18^ Geometries were first optimized in gas-phase and afterwards in the solvent indicated using PBF solvent model.^19^

### Fluoride-mediated cyclization

**Scheme 3:**
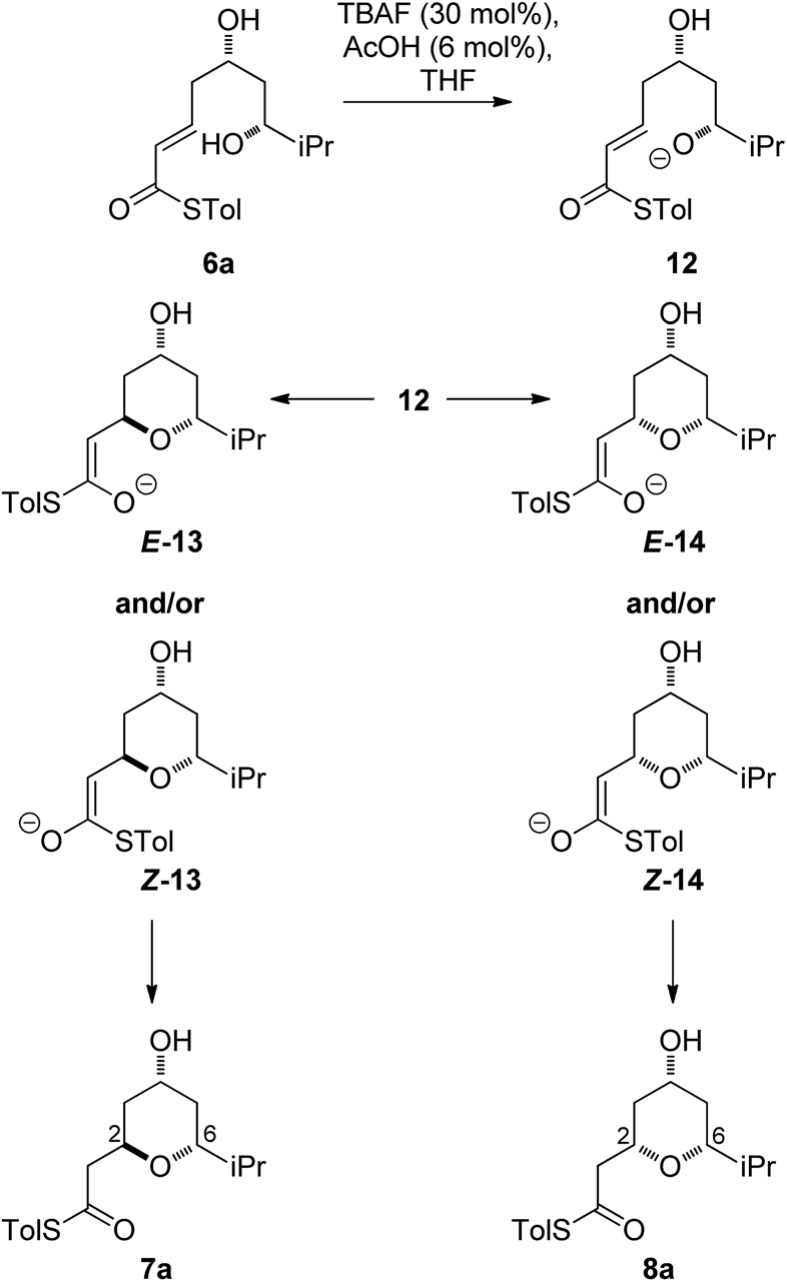
Mechanistic considerations for the TBAF cyclization.

As all of the TBAF-mediated reactions were *trans*-selective we initially chose to model the cyclization of **6a** to **7a** (Scheme 3). We rationalized that the active molecule is the alkoxide **12**, which then attacks the conjugate double bond to form either the enolate of the 2,6-*trans*-THP **13** or of 2,6-*cis*-THP **14**. The enolates can be formed as either E or Z-isomers. Since the interconversion would likely be slow, the enolate geometry should be determined by the thioester conformation (*s*-*cis* or *s*-*trans*) in the transition state.

**Fig. 1:**
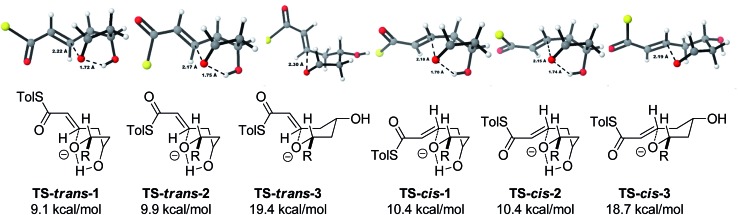
Transition states for the TBAF mediated cyclization. Activation enthalpies calculated in THF implicit solvent model and shown relative to the ground state conformation of alkoxide **12**. Tolyl and iPr groups omitted for clarity.

With this in mind, a thorough search for the transition states leading to the four possible enolates **E-13**, **Z-13**, **E-14**, **Z-14** was conducted. Several transition states leading to each of the enolates were found, lowest energy of which are shown in Fig. 1. Notably, the conformations of the lowest energy TSs were boat-like instead of the more common chair-like conformation. A strong intramolecular hydrogen bond between the 4-hydroxyl and the alkoxide stabilizes this conformation and makes it more favourable.^20,21^

Alternative chair-like transition states leading to both products were also found and are shown in Fig. 1. These, however, are significantly higher in energy, and therefore not significant.

E-transition states are lower than the corresponding Z-transition states, but the differences are not large. Similarly, the Z-thioenolates **Z-13** and **Z-14** are higher in energy than E-thioenolates because of the increased steric interactions. Once the product is formed, the boat conformation is no longer favourable and the THP thioenolates relax to the chair conformations, all of which were calculated to be lower in energy by 2–4 kcal mol^–1^.

While normal ester enolates are much more basic than alkoxides, thioester enolate p*K*_a_ is much lower and comparable to alkoxides.^22^ It is therefore not surprising that the E*-*thioenolates and the 4-alkoxides were found to be quite similar in energy. For the 2,6-*trans*-substrate the reaction end point before reprotonation would be the E-enolate, with the alkoxide being 1.5 kcal mol^–1^ higher in energy. For the 2,6-*cis*-substrate the 4-alkoxide is favoured over the E-thioenolate by 1.6 kcal mol^–1^. The overall thermodynamic product of the reaction should be the 2,6-*cis*-4-alkoxide, which is 0.5 kcal mol^–1^ lower in energy than the 2,6-*trans*-E-thioenolate. This adds further support to kinetic control in this reaction, because the major observed product is the 2,6-*trans*-THP.

**Fig. 2:**
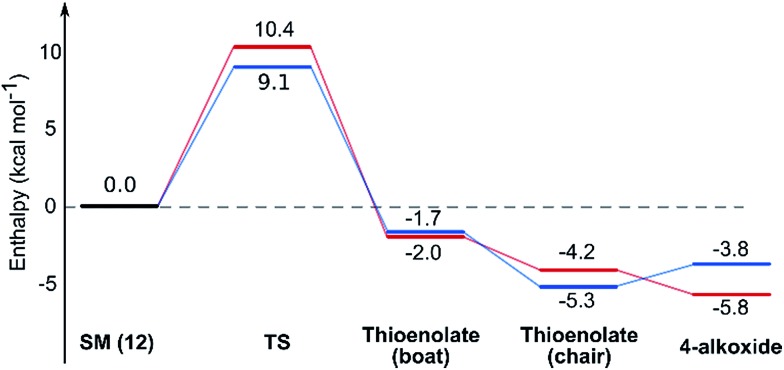
Energy diagram for the TBAF mediated lowest energy pathways to the 2,6-*trans* **7a** (blue) and 2,6-*cis* **8a** (red). Enthalpies calculated in THF implicit solvent model and are relative to the ground state conformation of alkoxide **12**.

Using all of this information a reaction energy profile was constructed (Fig. 2). The barrier for the forward reaction is 9.1 kcal mol^–1^ for the 2,6-*trans*-product and 10.4 kcal mol^–1^ for the 2,6-*cis*-product. Both are very low and are consistent with the observed speed of the reaction, which is usually complete in less than 10 minutes at or below room temperature. For the reverse reaction, the total energy barrier is 14.4 kcal mol^–1^, making it several orders of magnitude slower. This clearly shows that the reaction is under kinetic control, which matches experimental observations. Therefore the activation energies for the diastereomeric pathways are also determining the diastereoselectivity of the reaction. The 2,6-*trans*-boat transition state is 1.3 kcal mol^–1^ lower than the 2,6-*cis*-boat transition state, matching the observed diastereoselectivity well. One possible reason for this energy difference is the semi-eclipsed interaction of the β and γ-hydrogen atoms of the α,β-unsaturated thioester. This is present in the **TS-*cis*-1** (dihedral angle 37°), but not in the **TS-*trans*-1** (dihedral angle 161°). Another contributing factor would be the increased steric clash in the **TS-*cis*-1** from a pseudo-1,3-diaxial interaction between the protons at the 2- and 6- positions in the forming ring. This interaction is absent in the *trans*-transition states, but could be particularly pronounced in the *cis*-transition states because the protons are pointing slightly towards each other to allow the alkoxide attack from a favourable trajectory.

### Acid-mediated cyclization

**Scheme 4:**
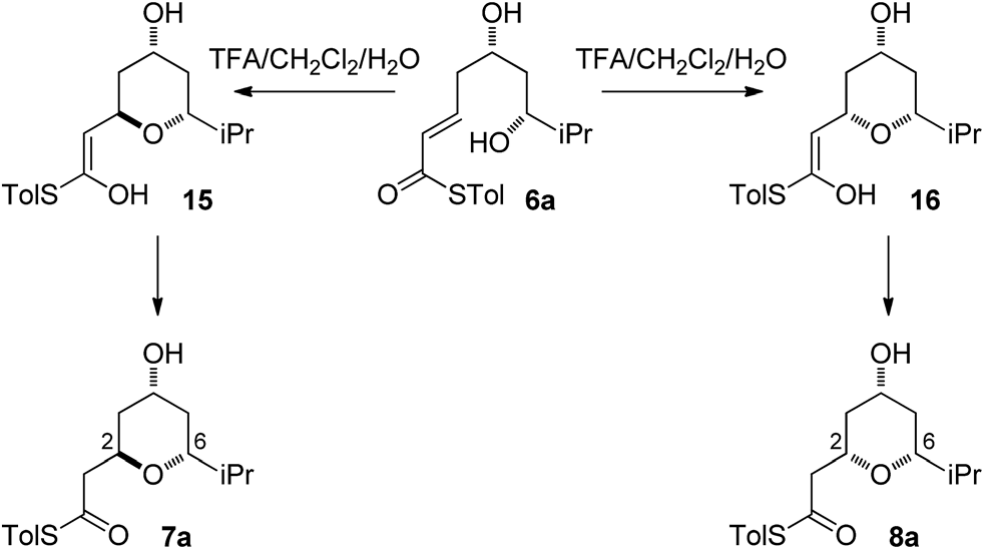
Mechanistic considerations for the acid-mediated cyclization.

With an explanation in hand for the *trans*-selectivity of the buffered TBAF-mediated reaction we turned our attention to the TFA-mediated *cis*-selective cyclization reaction (Scheme 4). As shown by Fuwa and ourselves in interconversion experiments, the process is likely to be under kinetic control.^13,14^ Therefore a simple thermodynamic preference for the 2,6-*cis*-diastereomers is not an adequate explanation for the observed stereoselectivity.

**Scheme 5:**
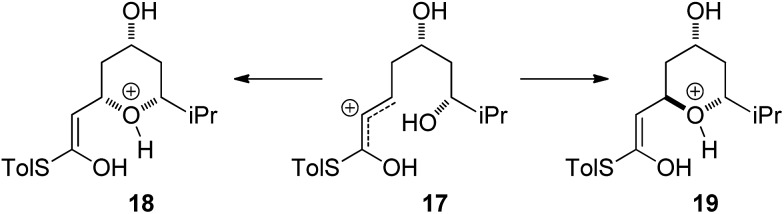
Allylic cation mechanism.

Currently there is no generally accepted mechanism for the acid mediated oxy-Michael cyclization. Based on studies by Houk,^8*a*^ Fuwa proposed an allylic cation type mechanism^23^ (Scheme 5), although no further experimental evidence to support this proposal has been reported.

Firstly, the viability of the allylic carbocation mechanism was tested by DFT calculations. When the lowest energy conformations of the cyclized intermediates **18** and **19** were submitted to geometry optimization at the DFT level, the THP rings opened back up during the process. This implies that there is no energy barrier for the opening of the ring and that the protonated cyclized intermediates **18** and **19** are unstable. Therefore it is highly unlikely that a simple protonation is the mechanism for the acid catalysis in this reaction. This mechanism would also provide little room to explain the different levels of diastereoselectivity achieved by the use of different acids as reported by Fuwa.^14^

**Fig. 3:**
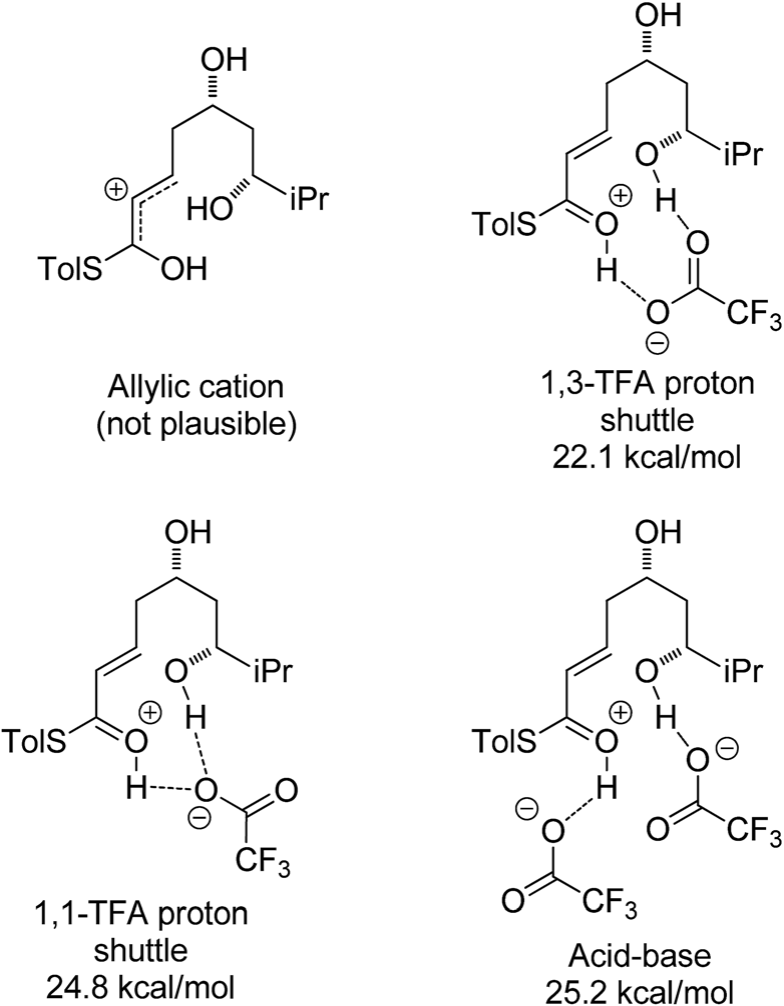
Possible activation modes in TFA mediated cyclization. Enthalpies shown are the activation enthalpies calculated in gas phase relative to the corresponding starting material-TFA complex ground state conformation.

Other potential modes of activation were then explored (Fig. 3). Among the mechanisms identified were two where TFA acts as proton shuttle, protonating the thioester and deprotonating the alcohol almost simultaneously. Another potential mechanism requires two different molecules of TFA, one of which acts as an acid and protonates the thioester and the other one acts as a base and deprotonates the alcohol nucleophile during the attack. Of these three mechanisms the 1,3-TFA proton shuttle mechanism was calculated to have the smallest activation energy, and the rest of the computational study focused on investigating it.

**Fig. 4:**
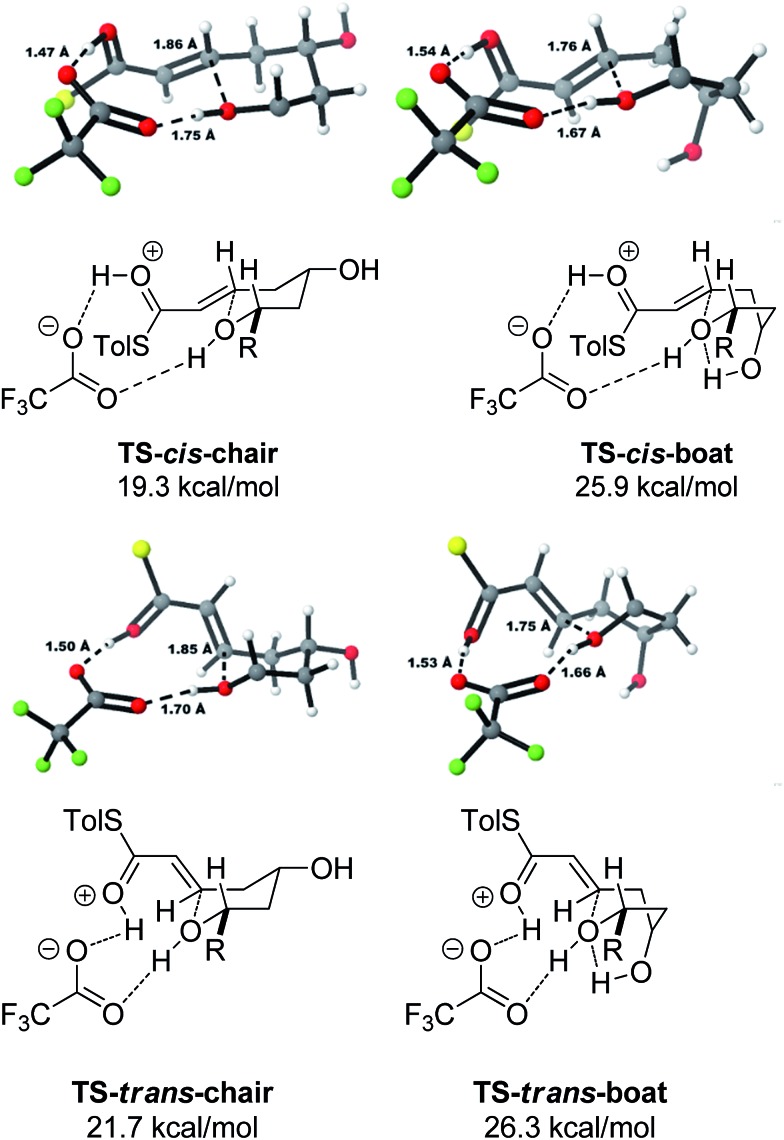
Transition states for the TFA mediated cyclization. Activation enthalpies calculated in DCM implicit solvent model and shown relative to the ground state conformation of diol **7a** complex with TFA. Tolyl and iPr groups omitted for clarity.

A thorough search for transition states leading to the two diastereomeric enols **15** and **16** revealed several chair- and boat-like transition states, of which the lowest energy ones are shown in Fig. 4. Because the TFA proton shuttle imposes a distance constraint between the alcohol and the thioester carbonyl group, only the transition states leading to the E-enols **15** and **16** are possible. The activation enthalpies were calculated to be 19.3 kcal mol^–1^ for the 2,6-*cis*-chair-like transition state and 21.7 kcal mol^–1^ for the 2,6-*trans*-chair-like transition state. **TS-*cis*-chair** is 1.9 kcal mol^–1^ lower in energy, consistent with the observed diastereoselectivity. The higher energy of the *trans*-transition state appears to be caused by an increased pseudo-1,3-diaxial steric clash between the 6-proton and the 2-thioester substituent. This interaction is not present in the *cis*-transition state. In all of the transition states, there are two hydrogen bonds between the TFA anion and the alcohol, and between the TFA anion and the protonated thioester. This allows the TFA to act as a proton shuttle and to simultaneously improve the electrophilicity of the thioester and the nucleophilicity of the alcohol.

**Fig. 5:**
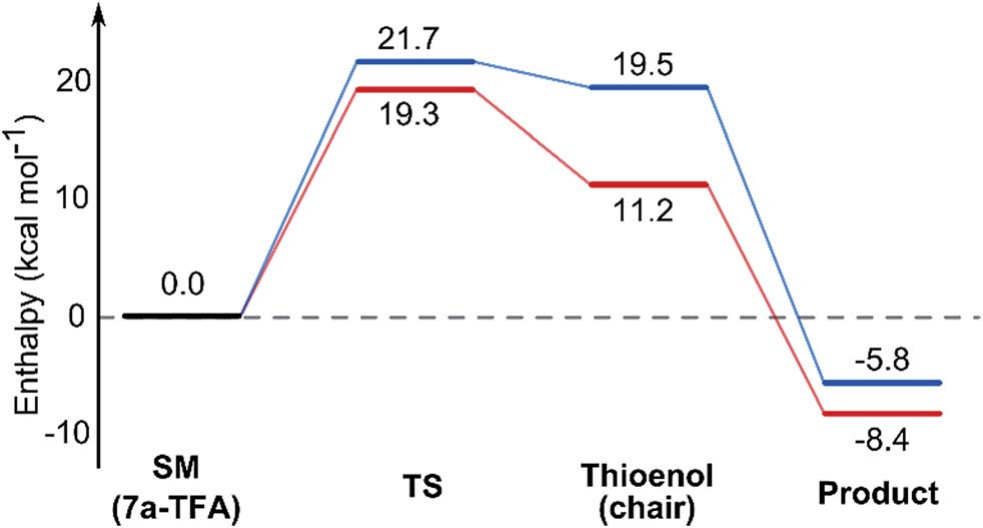
Energy diagram for the TBAF mediated lowest energy pathways to the 2,6-*cis* **9a** (red) and 2,6-*trans* **8a** (blue). Enthalpies calculated in DCM implicit solvent model and are relative to the ground state conformation of **7a** complex with TFA.

In contrast with the TBAF case, the 4-hydroxyl group is not directly involved in the stabilization of the transition state, and the boat-like transition states are both some 5 kcal mol^–1^ higher in energy than the corresponding chair-like transition states. Calculations also *confirmed kinetic control* in this reaction, as the activation energy of the reverse reaction is 8.4 kcal mol^–1^ higher than the forward reaction (Fig. 5).

### Synthetic investigation of the role of the 4–OH group

As the computational studies had implicated the 4-hydroxyl as the source of the stereodivergence, it was decided to test this hypothesis on a number of substrates. Substrates chosen for study were **20a**, **20b** and **20c**, which did not have the 4-hydroxyl group present and a substrate where the hydroxyl group was protected as a methyl ether **22**.

When TFA was used the cyclization of **20a**, **b**, **c** proceeded smoothly to form the 2,6-*cis*-THP products **21a**, **b**, **c** in moderate to good yields and with reasonable diastereoselectivities, however, the products of the TBAF mediated cyclization reactions were also the 2,6-*cis*-THP **21a**, **b**, **c** and were formed with even higher diastereoselectivity than under the TFA-mediated reaction conditions (Table 3).

**Table 3:** Cyclization of substrates without the 4-hydroxyl

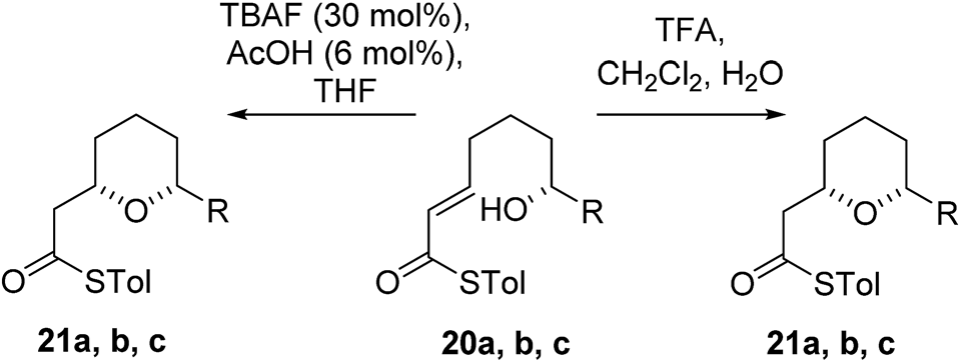					
Entry	Ratio *cis* : *trans* ^*a*^	TBAF yield^*b*^ (%)	R	TFA yield^*b*^ (%)	Ratio *cis* : *trans* ^*a*^
a	>20 : 1	27	iPr	47	4 : 1
b	>20 : 1	53	Ph	56	8 : 1
c	>20 : 1	25	C_7_H_15_	36	5 : 1

^*a*^Ratios obtained from integration of ^1^H NMR signals.

^*b*^Isolated yields after chromatography.

**Scheme 6:**
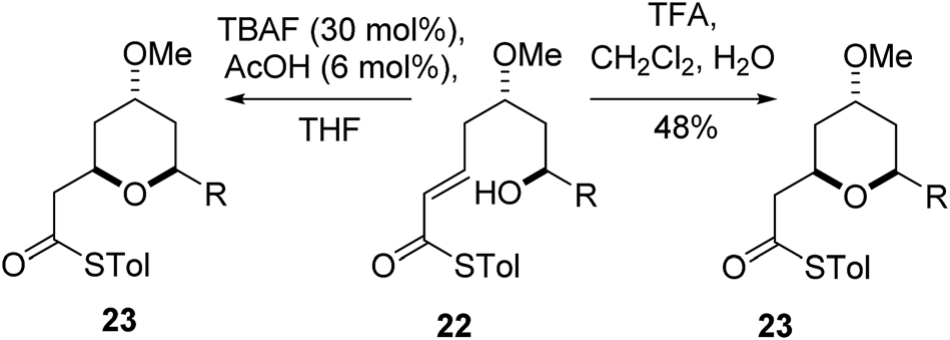
Cyclization of methyl ether **23**.

The final substrate studied was where the 4-hydroxyl group was capped as a methyl ether **22** (Scheme 6). Cyclization precursor **22** was subjected to both sets of cyclization conditions. While the TFA-mediated conditions yielded the *cis*-diastereomer **23** cleanly, the TBAF-mediated conditions proceeded very slowly, with substantial hydrolysis of the thioester. However, despite this ^1^H NMR analysis of the crude reaction mixture showed that only the *cis*-product **23** had been formed, confirming our hypothesis and previous results that a hydrogen-bond donor in the 4-position is required for the formation of the 2,6-*trans*-diastereomer under buffered TBAF-mediated conditions. The results of these synthetic studies are in complete agreement with the predictions of the computational studies. The computational studies showed that chair-like transition states in the TBAF-mediated reaction would provide the reverse diastereoselectivity with the 2,6-*cis*-chair TS being 1.5 kcal mol^–1^ lower in energy than the 2,6-*trans*-chair TS. This explains why the 2,6-*trans* selectivity is not observed in the 4-dehydroxy substrates **20a–c** and the methyl ether **22**, because in those cases there is no way of stabilizing the boat-like TS and the chair-like transition state would be more favoured, giving the 2,6-*cis* products.

### Differences in the reactivity of thioester 1 and oxoester 4

With explanations for the origins of the stereodivergence in the cyclization reactions, the last remaining question for the computational study was the dramatic difference in the reactivity of thioester **1** and oxoester **4** in this oxy-Michael cyclization. Only the 2,6-*cis*-pathways were investigated as we were primarily interested in the reactivity of the substrate: thioester **1** cyclised to the 2,6-*cis*-THP whereas oxoester **4** decomposed. We identified a similar transition state for the oxoester as in the case of thioester, however, the energy profile of the reaction showed a much higher transition state energy for oxoester. This difference of 7.6 kcal mol^–1^ compared to the thioester would make the oxoester cyclization much slower. The slower cyclization would allow for competing decomposition reactions to dominate. One possible decomposition pathway is that the potentially acid sensitive styrenyl alcohol's ionization competes with conjugate addition under the Brønsted acid conditions. The transition state geometries of the thioester and oxoester cyclizations are very similar and therefore it appears very unlikely that steric effects would be the cause for the dramatic difference. An alternative cause would be the differences in the electronic structure; that the oxoester **4** is a less efficient electrophile than the thioester **1**, which would manifest itself in the LUMOs of the substrates.

**Fig. 6:**
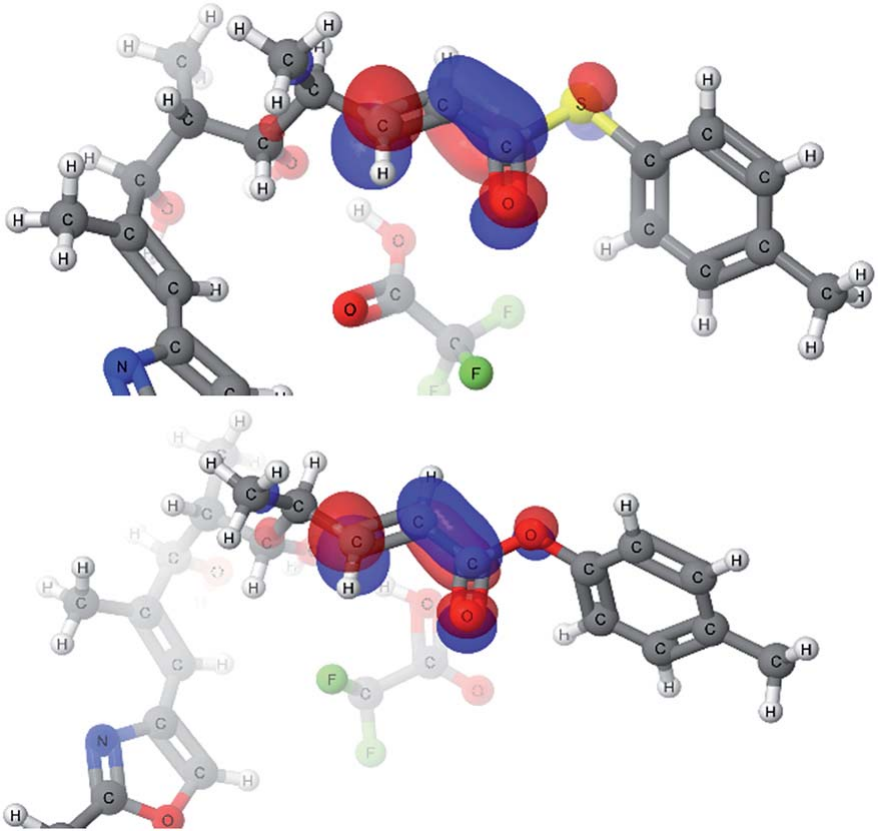
LUMOs of the thioester-TFA complex (top) and oxoester-TFA complex (bottom).

Two very similar low lying conformations of the thioester-TFA and the oxoester-TFA complex were compared (Fig. 6). The electron density distribution is very similar for both substrates but the energy for the thioester-TFA complex LUMO is –1.43 eV and –1.06 eV for the oxoester-TFA complex. While this difference is relatively small, it is significant and shows that the sulfur atom makes the LUMO more accessible for nucleophiles.

A possible explanation for the difference in the LUMO energies between thioesters and oxoesters might be that sulfur lone pair (in sp3 orbital made up from a contribution of the 3p orbitals) has a smaller overlap with the C=O π* orbital of the ester than the oxygen lone pair (in sp3 orbital made up from a contribution of the 2p orbitals). This would have the effect of making the thioester electrophile more enone-like and more electrophilic in comparison with the α,β-unsaturated oxoester,^24^ this reactivity profile has recently been reported in an intramolecular oxy-Michael cyclization onto an enone *versus* an α,β-unsaturated oxoester.^10*a*^

### Stereodivergent synthesis of diospongins A and B

**Scheme 7:**
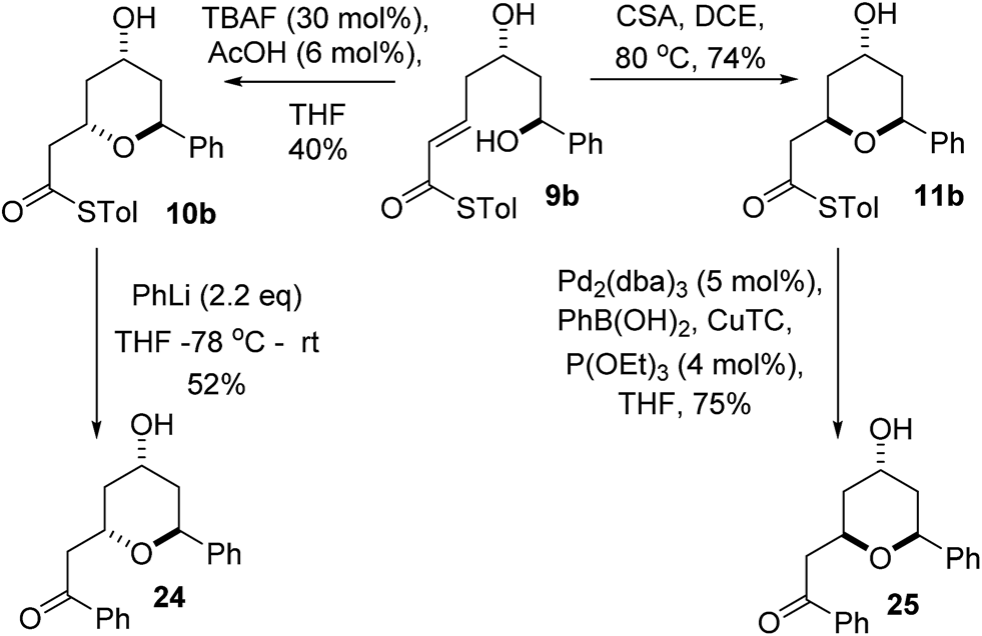
Stereodivergent synthesis of diospongins A and B.

We realized that compounds **10b** and **11b**, the products of the stereodivergent oxy-Michael reactions of **9b**, were possible precursors to the natural products diospongins B and A respectively. Diospongins A and B are members of the biaryl heptanoid class of natural products and have been reported to exhibit anti-osteoporotic activity.^4^ There have been several syntheses of these molecules,^25^ but none to date have exploited the potential for a stereodivergent synthesis from a common precursor, and so we sought to showcase the stereodivergent oxy-Michael reaction with a synthesis of both these natural products. In theory both diospongin A and B could be accessed in one step from these precursors by a Liebeskind–Srogl type coupling reaction of the thioester with phenyl boronic acid under Pd-catalysis.^26,14a^ Indeed this reaction was successfully employed in the synthesis of diospongin A **25**, from **11b** in 75% yield (Scheme 7). However, application of the same conditions to **10b** resulted only in re-isolation of starting material. Despite the investigation of several different solvents, temperatures and catalyst loadings and ligands we were unable to get **10b** to react. Application of the alternative Liebeskind organostannane conditions^27^ also resulted in no reaction taking place. We are unable to explain this lack of reactivity for the 2,6-*trans*-diastereomer **10b** at this time. We therefore had to adopt an alternative strategy for the conversion of the thioester to the desired phenyl ketone. Tetrahydropyran-4-ol **10b** was instead treated with PhLi at –78 °C and warmed to RT overnight, which did give diospongin B **24** in 52% yield (Scheme 7). Spectroscopic data of both diospongins were identical to those reported previously in the literature.^25^ This marks the first syntheses of these diastereomeric natural products using a stereodivergent process from the same common precursor.

## Conclusions

A stereodivergence was observed in the oxy-Michael reaction of α,β-unsaturated thioesters to form THP rings. These THP rings are present in a large number of structurally complex and biologically active natural products. Computational investigations of this stereodivergence indicated that it resulted from the participation of the 4-hydroxyl group in a hydrogen bond with the cyclising alkoxide which enforced a boat-like transition state in the buffered TBAF mediated reaction which led to the 2,6-*trans*-THP product. The Brønsted acid mediated reaction had no such interaction and proceeded through a chair-like transition state to generate the 2,6-*cis*-THPs. These computational studies suggested that *both reactions were under kinetic control*. Synthetic studies confirmed these computational predictions. When the 4-hydroxyl group was present the substrates exhibited stereodivergent reaction pathways under the reaction conditions. However, when the 4-hydroxyl group was removed or protected no stereodivergence was seen. These results allow us to suggest guidelines for the future diastereoselective synthesis of 2,6-disubstituted THPs.

• Use α,β-unsaturated thioesters: they are more reactive in cyclizations than α,β-unsaturated oxoesters.

• For the formation of 2,6-*cis*-THPs use a Brønsted acid promoted cyclization.

• For the formation of 2,6-*trans*-THPs, a 4-hydroxyl group is essential in a buffered TBAF promoted cyclization.

The utility of these guidelines and the stereodivergent oxy-Michael reaction was further demonstrated by the stereodivergent synthesis of the anti-osteoporotic natural products diospongin A and B from a common precursor.

In summary, we have used a combined computational and experimental approach to develop a robust and simple procedure for the synthesis of 4-hydroxy-2,6-*cis*- and 4-hydroxy-2,6-*trans*-THP rings and elucidated the mechanism of this stereodivergence. We believe this knowledge will be extremely useful for those seeking to synthesize functionalized THP rings in high yields and with high selectivities in the context of natural product synthesis.

## References

[cit1] Nasir N. M., Ermanis K., Clarke P. A. (2014). Org. Biomol. Chem..

[cit2] Searle P. A., Molinski T. F. (1995). J. Am. Chem. Soc..

[cit3] Horton P. A., Koehn F. E., Longley R. E., McConnell O. J. (1994). J. Am. Chem. Soc..

[cit4] Yin J., Kouda K., Tezuka Y., Le Tran Q., Miyahara T., Chen Y., Kadota S. (2004). Planta Med..

[cit5] Cichewicz F. A., Crews P. (2004). Org. Lett..

[cit6] (a) Perry M. A., Rychnovsy S. D. and Sizemore N., Synthesis of Saturated Oxygen Heterocycles I, Topics in Heterocyclic Chemistry 35, ed. J. Cossy, Springer-Verlag, Berlin, Heidelberg, 2014, pp. 43–95.

[cit7] (a) For a review see: Nising C. F. Bräse S., Chem. Soc. Rev., 2012, 41 , 988 . 21796323 10.1039/c1cs15167c

[cit8] Banwell M. G., Bui C. T., Pham H. T. T., Simpson G. W. (1996). J. Chem. Soc., Perkin Trans. 1.

[cit9] Houk K. N., Strozier R. W. (1973). J. Am. Chem. Soc..

[cit10] (a) For computational and synthetic studies on an intramolecular system see: Hari T. P. A. Wilke B. I. Davey J. A. Boddy C. N., J. Org. Chem., 2016, 81 , 415 . 26675500 10.1021/acs.joc.5b02014

[cit11] Lee K., Kim H., Hong J. (2011). Org. Lett..

[cit12] Ball M., Bradshaw B. J., Dumeunier R., Gregson T. J., MacCormick S., Omori H., Thomas E. J. (2006). Tetrahedron Lett..

[cit13] Clarke P. A., Ermanis K. (2012). Org. Lett..

[cit14] Fuwa H., Noto K., Sasaki M. (2011). Org. Lett..

[cit15] See ESI for details

[cit16] Halgren T. A. (1996). J. Comput. Chem..

[cit17] Becke A. D. (1988). Phys. Rev. A: At., Mol., Opt. Phys..

[cit18] Krishnan R., Binkley J. S., Seeger R., Pople J. A. (1980). J. Chem. Phys..

[cit19] Tannor D. J., Marten B., Murphy R., Friesner R. A., Sitkoff D., Nicholls A., Ringnalda M., Goddard III W. A., Honig B. (1994). J. Am. Chem. Soc..

[cit20] Pellicena M., Krämer K., Romea P., Urpí F. (2011). Org. Lett..

[cit21] Paterson I., Haslett G. W. (2013). Org. Lett..

[cit22] Bordwell F. G., Fried H. E. (1991). J. Org. Chem..

[cit23] Fuwa H. (2012). Heterocycles.

[cit24] Thioesters have previously been shown to exhibit characteristics similar to ketones: El-Asar A. M. M. Nash C. P. Ingraham L. L., Biochemistry, 1982, 21 , 1972 . 7082657 10.1021/bi00537a042

[cit25] Sawant K. B., Jennings M. P. (2006). J. Org. Chem..

[cit26] Liebeskind L. S., Srogl J. (2000). J. Am. Chem. Soc..

[cit27] Wittenberg R., Srogl J., Egi M., Liebeskind L. S. (2003). Org. Lett..

