# Supplementary material for: The stereodivergent formation of 2,6-cis and 2,6-trans-tetrahydropyrans: experimental and computational investigation of the mechanism of a thioester oxy-Michael cyclization
Source: Chem Sci. 2016 Aug 30;8(1):482–90. doi: 10.1039/c6sc03478k (PMC5298201; doi:10.1039/c6sc03478k)

Electronic Supplementary Material (ESI) for Chemical Science.
This journal is © The Royal Society of Chemistry 2016

Supporting Information

The Stereodivergent Formation of 2,6-*cis* and 2,6-*trans*-Tetrahydropyrans: Experimental and Computational Investigation of the Mechanism of a Thioester Oxy-Michael Cyclization.

Kristaps Ermanis, Yin-Ting Hsiao, Uğur Kaya, Alan Jeuken and Paul A. Clarke*

General Experimental 1
Experimental Procedures for the Cyclization Reactions 2
Experimental Procedures for t73he Synthesis of the Cyclization Substrates 19
References 41
Details of the Computational Studies 41
Calculations on TBAF mediated cyclizations 42
Calculations on TFA mediated cyclizations 60
Copies of ^{1}H and ^{13}C NMR Spectra for Compounds in the Manuscript 73

General Experimental

Melting points were determined using a Stuart SMP3 apparatus. Infra-red spectra were acquired on a ThermoNicolet Avatar 370 FT-IR spectrometer. Nuclear magnetic resonance spectra were recorded on a Jeol ECX-400, a Jeol ECS-400, Bruker DRX 500 or a Bruker AV700 spectrometer at ambient temperature; chemical shifts are quoted in parts per million (ppm) and were referenced as follows: chloroform-*d*, 7.26 ppm; DMSO-d_6, 2.54 ppm for ^{1}H NMR; chloroform-*d*, 77.0 ppm; DMSO, 128.0 ppm for ^{13}C NMR. Coupling constants (*J*) are quoted in Hertz. Mass spectrometry was performed by the University of York mass spectrometry service using electron spray ionisation (ESI) technique. Thin layer chromatography was performed on glass-backed plates coated with Merck Silica gel 60 F_{254}. The plates were developed using ultraviolet light, acidic aqueous ceric ammonium molybdate, basic aqueous potassium permanganate or ethanolic anisaldehyde. Liquid

chromatography was performed using forced flow (flash column) with the solvent systems indicated. The stationary phase was silica gel 60 (220–240 mesh) supplied by Fluorochem or silica gel Merck TLC grade 11695 supplied by Sigma-Aldrich. Hexane, DCM, toluene, THF were all purified using Innovative Technology Solvent Purification System; triethylamine were distilled from calcium hydride. All other solvents and reagents were used as received from commercial suppliers. All numbering on the structures below is for the benefit of characterisation and does not necessarily conform to IUPAC rules.

Cyclisation Procedures

(±)-S-p-tolyl 2-((2R,4S,6R)-4-hydroxy-6-isopropyltetrahydro-2H-pyran-2-yl)ethanethioate (7a)

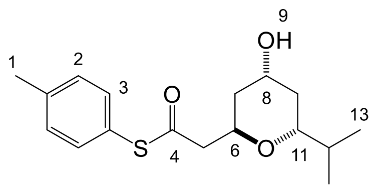

Thioester **6a** (7.5 mg, 0.024 mmol) was dissolved in dry THF (0.3 mL). A solution of acetic acid (0.002 mmol, 6 mol%) and tetrabutylammonium fluoride (0.008 mmol, 30 mol%) was added dropwise to the reaction mixture at 0 °C under N_2 atmosphere. After stirring for 2 hours at 0 °C and 1 hour at room temperature the reaction was quenched with saturated aqueous solution of $NaHCO_3$ (2 mL). The phases were separated and the aqueous layer was extracted with diethyl ether (3x2 mL). The combined organics were dried over $MgSO_4$, filtered, concentrated *in vacuo* and purified by flash chromatography (20% ethyl acetate in petroleum ether) on a silica gel column to yield **7a** as a colourless oil (5.2 mg, 69%).

IR (film): υ_{max} 3450, 2959, 2918, 1730, 1467, 1368, 1247, 1057 cm^{-1}; **^{1}H-NMR** (400 MHz, $CDCl_3$) δ 7.35 (2H, d, *J* = 8.0 Hz, H-2), 7.15 (2H, d, *J* = 8.0 Hz, H-3), 4.55 (1H, dddd, *J* = 12.4, 9.8, 2.6 and 2.6 Hz, H-8), 3.77 (1H, ddd, *J* = 10.6, 5.2 and 2.1 Hz, H-11), 3.44 – 3.36 (1H, m, H-6), 2.91 (1H, dd, *J* = 17.7 and 6.0 Hz, H-5), 2.43 (1H, dd, *J* = 17.7 and 10.7

Hz, H-5), 2.35 (3H, s, H-1), 2.20 (1H, ddd, *J* = 13.8, 4.4 and 2.6 Hz, H-7), 1.72 (1H, ddd, *J* = 14.4, 9.8 and 2.1 Hz, H-10), 1.65 – 1.50 (3H, m, H-7 + H-10 + H-12), 0.90 (3H, d, *J* = 6.8 Hz, H-13), 0.90 (3H, d, *J* = 6.8 Hz, H-13) ppm. **^{13}C-NMR** (101 MHz, $CDCl_3$): δ 193.3, 138.8, 134.4, 130.0, 125.0, 77.5, 77.2, 71.6, 39.6, 39.4, 36.2, 34.0, 29.7, 21.2, 18.5, 17.1 ppm. **MS (ESI)**: m/z 331 ($M+Na^+$); HRMS: found: ($M+Na^+$) 331.1726, $C_{17}H_{24}NaO_3S$ requires ($M+Na^+$) 331.1338.

(±)-*S*-(*p*-Tolyl) 2-((2*S*,4*R*,6*S*)-4-hydroxy-6-phenyltetrahydro-2H-pyran-2-yl)ethanethioate (7b)

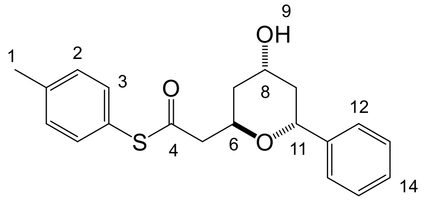

(5*S*,7*S*,*E*)-*S*-*p*-Tolyl 5,7-dihydroxy-7-phenylhept-2-enethioate (**6b**) (17.0 mg, 0.050 mmol) was dissolved in dry THF (1.3 mL, 0.04 M). A solution of acetic acid (0.0030 mmol, 0.06 eq.) and tetrabutylammonium fluoride (0.015 mmol, 0.3 eq.) was added over a period of 3 minutes to the reaction mixture at -10 °C under N_2 atmosphere. The reaction mixture was treated after 1.5 and 2 hours with additional solution (0.1 mL) of acetic acid and tetrabutylammonium fluoride. After stirring for 3 hours at -10 °C the reaction was quenched with saturated aqueous solution of $NaHCO_3$ (3 mL). The phases were separated and the aqueous layer was extracted with diethyl ether (3 × 3 mL). The combined organics were dried over $MgSO_4$, filtered, concentrated *in vacuo* and purified by flash silica gel column chromatography (20 to 50 % ethyl acetate in petroleum ether) to yield **7b** (6.8 mg, 0.020 mmol, 40 % yield) as a yellow oil.

IR (film): ν_{max} 3437, 2964, 2923, 2852, 1735, 1630, 1489, 1452, 1253, 1073 cm^{-1}. **^{1}H-NMR** (400 MHz, $CDCl_3$): δ = 7.39 – 7.31 (7H, m, Ar-H), 7.15 (2H, d, *J* = 7.8 Hz, H-3), 5.06 (1H, dd, *J* = 7.8 and 5.3 Hz, H-11), 4.64 (1H, dddd, *J* = 11.9, 10.6, 5.8 and 2.9 Hz, H-6), 3.46 – 3.35 (1H, m, H-8), 2.92 (1H, dd, *J* = 17.7 and 5.8 Hz, H-5), 2.44 (1H, dd, *J* = 17.7 and 10.6 Hz, H-5), 2.35 (3H, s, H-1), 2.09 (1H, br, H-9), 1.92 (2H, m, H-10), 1.57

(2H, m, H-7) ppm. **^{13}C-NMR** (101 MHz, $CDCl_3$): δ 169.6, 144.2, 139.0, 134.6, 130.2, 128.7, 127.9, 127.8, 125.6, 77.3, 76.4, 69.6, 45.2, 39.6, 36.6, 21.2 ppm. **MS (ESI)**: m/z 343 (M+H$^+$); HRMS: found: (M+H$^+$) 343.1341, $C_{20}H_{23}O_3S$ requires (M+H$^+$) 343.1362

(±)-*S*-*p*-Tolyl 2-((2*R*,4*R*,6*S*)-6-heptyl-4-hydroxytetrahydro-2H-pyran-2-yl)ethanethioate (7c)

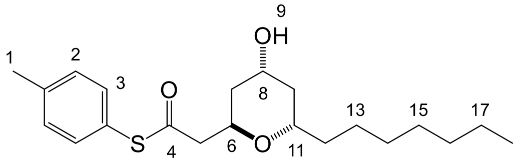

Thioester **6c** (15.0 mg, 0.041 mmol) was dissolved in dry THF (0.3 mL). A solution of acetic acid (0.002 mmol, 6 mol%) and tetrabutylammonium fluoride (0.012 mmol, 30 mol%) was added to the reaction mixture over a period of 3 minutes at 0 °C under N_2 atmosphere. After stirring for 5 hours at 0 °C the reaction was quenched with saturated aqueous solution of $NaHCO_3$ (2 mL). The phases were separated and the aqueous layer was extracted with diethyl ether (3 × 2 mL). The combined organics were dried over $MgSO_4$, filtered, concentrated *in vacuo* and purified by flash chromatography (20% ethyl acetate in petroleum ether) on a silica gel column to yield **7c** as a colourless oil (6.2 mg, 41%).

. **IR** (film): $υ_{max}$ 3424, 2924, 2854, 1715, 1489, 1464, 1378, 1250, 1060 cm^{-1}; **^{1}H-NMR** (400 MHz, $CDCl_3$) δ 7.35 (2H, d, *J* = 8.1 Hz, H-2), 7.15 (2H, d, *J* = 8.1 Hz, H-3), 4.56 (1H, dddd, *J* = 12.3, 10.3, 5.9 and 2.4 Hz, H-6), 3.99 – 3.91 (1H, m, H-8), 4.39 (1H, dddd, *J* = 10.6, 10.6, 5.9 and 4.5 Hz, H-11), 2.91 (1H, dd, *J* = 17.7 and 5.9 Hz, H-5), 2.43 (1H, dd, *J* = 17.7 and 10.3 Hz, H-5), 2.35 (3H, s, H-1), 2.22 – 2.15 (1H, m, H-10), 1.74 (1H, ddd, *J* = 14.5, 9.8 and 2.4 Hz, H-7), 1.64 – 1.39 (4H, m, H-7 + H-10 + H-12), 1.33 – 1.17 (10H, m, H-13 + H-14 + H-15 + H-16 + H-17), 0.88 (3H, t, *J* = 6.6 Hz) ppm. **^{13}C-NMR** (101 MHz, $CDCl_3$): δ 193.3, 138.9, 134.4, 130.0, 124.2, 77.2, 68.0, 67.3, 43.1, 38.0, 36.5, 36.1, 31.8, 29.5, 29.3, 29.2, 25.5, 22.6, 21.2, 14.1 ppm **MS (ESI)**: m/z 365 (M+H$^+$) 387 (M+Na$^+$);

HRMS: found: (M+H$^+$) 365.2149, (M+Na$^+$) 387.1969, $C_{21}H_{33}O_3S$ requires (M+H$^+$) 365.2145, $C_{21}H_{32}NaO_3S$ requires (M+Na$^+$) 387.1964

(±)-*S*-*p*-Tolyl 2-((2*S*,4*S*,6*R*)-4-hydroxy-6-isopropyltetrahydro-2H-pyran-2-yl)ethanethioate (8a)

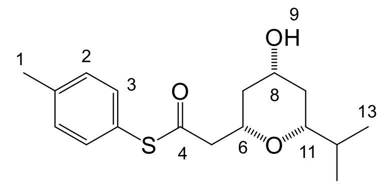

Thioester **6a** (15.0 mg, 0.049 mmol) was dissolved in DCM (0.5 mL) and water (0.05 mL) was added. The mixture was cooled down to 0 °C and trifluoroacetic acid (0.45 mL) was added dropwise. After stirring for 5.5 hours at room temperature the reaction was quenched with saturated aqueous solution of $NaHCO_3$ (2 mL) and diluted with DCM (2 mL). The aqueous layer was extracted with DCM (2 × 2 mL). The combined organics were dried over $MgSO_4$, filtered, concentrated *in vacuo* and purified by flash chromatography (20% ethyl acetate in petroleum ether) on a silica gel column to yield **8a** as a colourless oil (9.9 mg, 66%).

IR (film): υ_{max} 3419, 2925, 2850, 1704, 1464, 1364, 1221, 1020 cm^{-1}; **^{1}H-NMR** (400 MHz, $CDCl_3$) δ 7.28 (2H, d, *J* = 8.1 Hz, H-2), 7.21 (2H, d, *J* = 8.1 Hz, H-3), 3.86 – 3.75 (2H, m, H-6 + H-8), 3.00 (1H, ddd, *J* = 11.1, 6.7 and 1.7 Hz, H-11), 2.92 (1H, dd, *J* = 14.6 and 8.1 Hz, H-5), 2.69 (1H, dd, *J* = 14.6 and 4.9 Hz, H-5), 2.37 (3H, s, H-1), 2.04 – 1.95 (2H, m, H-7), 1.85 (1H, ddd, *J* = 15.2, 7.0 and 6.7 Hz, H-10), 1.75 – 1.65 (1H, m, H-10), 1.43 (1H, septd, *J* = 6.7 and 1.7 Hz, H-12), 0.96 (3H, d, *J* = 6.7 Hz, H-13), 0.90 (3H, d, *J* = 6.7 Hz, H-13) ppm. **^{13}C-NMR** (101 MHz, $CDCl_3$): δ 193.4, 139.7, 134.4, 130.0, 123.8, 80.8, 72.3, 68.3, 49.6, 37.8, 34.1, 33.0, 29.7, 21.3, 18.6 ppm. **MS (ESI)**: m/z 331 (M+Na$^+$); HRMS: found: (M+Na$^+$) 331.1343, $C_{17}H_{24}NaO_3S$ requires (M+Na$^+$) 331.1338

(±)-*S*-(*p*-Tolyl) 2-((2*S*,4*R*,6*R*)-4-hydroxy-6-phenyltetrahydro-2H-pyran-2-yl) ethanethioate (8b)

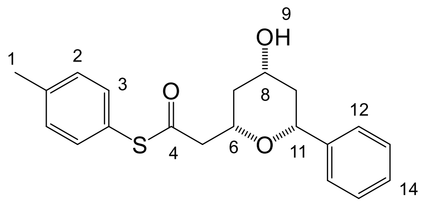

Diol **6b** (10 mg, 0.029 mmol) was dissolved in DCE (2.0 mL) and CSA (3.4 mg, 0.014 mmol, 50 mol %) was added in one portion. The reaction was heated to 60 ºC and left to stir for 24 hours. Another portion of CSA (3.4 mg, 0.014 mmol, 50 mol %) was added and the reaction was stirred for another 24 hours. A final portion of CSA (13..6 mg, 0.58 mmol, 2 eq.) was added and the reaction mixture was heated to 80 ºC and left to stir for 24 hours. The reaction was quenched with Et_3N, washed with $NaHCO_3$ (2 × 5 mL) and brine (2 × 5 mL), dried over $MgSO_4$, filtered and concentrated *in vacuo*. The residue was purified by flash silica gel column chromatography (50% diethyl ether in petroleum ether) to yield **8b** as a colourless oil (5.8 mg, 58%).

IR (film): ν_{max} 3396, 2922, 2855, 1703, 1495, 1454, 1368, 1064 cm^{-1}. **^{1}H NMR** (400 MHz, $CDCl_3$): δ 7.39 – 7.27 (7H, m, Ar-H), 7.20 (2H, d, *J* = 8.0 Hz, H-3), 4.42 (1H, dd, *J* = 11.5, 2.0 Hz, H-11), 4.08 – 3.95 (2H, m, H-6 + H-8), 3.06 (1H, dd, *J* = 14.9 and 7.0 Hz, H-5), 2.83 (1H, dd, *J* = 14.9, 5.9 Hz, H-5), 2.37 (3H, s, H-1), 2.28 – 2.10 (2H, m, H-10), 1.55 – 1.32 (2H, m, H-7) ppm. **^{13}C-NMR** (101 MHz, $CDCl_3$): δ 195.6, 142.5, 139.7, 134.4, 130.0, 128.3, 127.2, 125.7, 124.2, 73.5, 69.1, 64.6, 49.7, 39.9, 37.9, 21.3 ppm. **MS (ESI)**: m/z 365 ($M+Na^+$); HRMS: found: ($M+Na^+$) 365.1151, $C_{20}H_{22}NaO_3S$ requires ($M+Na^+$) 365.1182

(±)-*S*-*p*-Tolyl 2-((2*S*,4*R*,6*S*)-6-heptyl-4-hydroxytetrahydro-2H-pyran-2-yl)ethanethioate (8c)

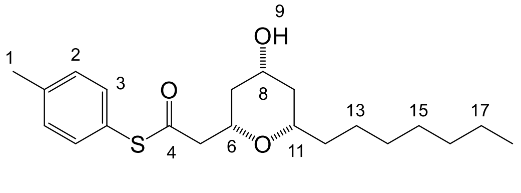

Thioester **6c** (15.0 mg, 0.041 mmol) was dissolved in DCM (0.5 mL) and water (0.05 mL) was added. The mixture was cooled down to 0 °C and trifluoroacetic acid (0.45 mL) was added dropwise. After stirring for 5.5 hours at room temperature the reaction was quenched with saturated aqueous solution of $NaHCO_3$ (2 mL) and diluted with DCM (2 mL). The aqueous layer was extracted with DCM (2 × 2 mL). The combined organics were dried over $MgSO_4$, filtered, concentrated *in vacuo* and purified by flash chromatography (20% ethyl acetate in petroleum ether) on a silica gel column to yield **8c** as a colourless oil (7.1 mg, 47%).

IR (film): υ_{max} 3394, 2925, 2850, 1704, 1464, 1371, 1085, 1035 cm^{-1}; **^{1}H-NMR** (400 MHz, $CDCl_3$) δ 7.28 (2H, d, *J* = 8.0 Hz, H-2), 7.21 (2H, d, *J* = 8.0 Hz, H-3), 3.86 – 3.76 (2H, m, H-6 + H-8), 3.33 – 3.25 (1H, m, H-11), 2.94 (1H, dd, *J* = 14.7, 7.8 Hz, H-5), 2.70 (1H, dd, *J* = 14.7, 5.2 Hz, H-5), 2.37 (3H, s, H-1), 2.05-1.90 (2H, m, H-7 + H-10), 1.64 – 1.39 (4H, m, H-7 + H-10 + H-12), 1.33 – 1.17 (10H, m, H-13 + H-14 + H-15 + H-16 + H-17), 0.87 (3H, t, *J* = 6.6 Hz, H-18) ppm. **^{13}C-NMR** (101 MHz, $CDCl_3$): δ 195.7, 139.7, 134.4, 130.0, 124.2, 75.8, 72.2, 68.0, 49.6, 41.0, 40.7, 36.0, 31.8, 29.5, 29.3, 25.6, 22.7, 21.3, 14.1 ppm. **MS (ESI)**: m/z 365 ($M+H^+$) 387 ($M+Na^+$); HRMS: found: ($M+H^+$) 365.2142, ($M+Na^+$) 387.1958, $C_{21}H_{33}O_3S$ requires ($M+H^+$) 365.2145, $C_{21}H_{32}NaO_3S$ requires ($M+Na^+$) 387.1964

(±)-*S*-*p*-Tolyl 2-((2*S*,4*S*,6*S*)-4-hydroxy-6-isopropyltetrahydro-2H-pyran-2-yl)ethanethioate (10a)

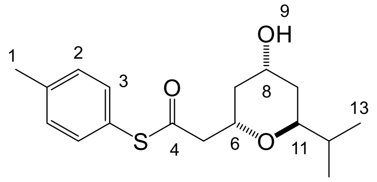

Thioester **9a** (8.0 mg, 0.026 mmol) was dissolved in dry THF (0.3 mL). A solution of acetic acid (0.002 mmol, 6 mol%) and tetrabutylammonium fluoride (0.008 mmol, 30 mol%) was added to the reaction mixture over a period of 3 minutes at 0 °C under N_2 atmosphere. After 5 hours TLC still showed starting material, therefore solution containing

acetic acid (0.004 mmol, 12 mol%) and tetrabutylammonium fluoride (0.016 mmol, 60 mol%) was added and the reaction warmed to room temperature. After stirring for another 1 hour the reaction was quenched with saturated aqueous solution of $NaHCO_3$ (2 mL). The phases were separated and the aqueous layer was extracted with diethyl ether (3 × 2 mL). The combined organics were dried over $MgSO_4$, filtered, concentrated *in vacuo* and purified by flash chromatography (20% ethyl acetate in petroleum ether) on a silica gel column to yield **10a** as a colourless oil (5.5 mg, 69%).

IR (film): υ_{max} 3448, 2957, 2925, 2853, 1736, 1467, 1439, 1385, 1246, 1053 cm^{-1}; **^{1}H-NMR** (400 MHz, $CDCl_3$) δ 7.35 (2H, d, *J* = 8.0 Hz, H-2), 7.11 (2H, d, *J* = 8.0 Hz, H-3), 4.49 (1H, dddd, *J* = 11.8, 6.7, 6.6 and 2.8 Hz, H-8), 3.65 (1H, ddd, *J* = 9.5, 4.8 and 2.6 Hz, H-11), 3.37 (1H, dddd, *J* = 11.2, 11.0, 5.9 and 4.3 Hz, H-6), 2.90 (1H, dd, *J* = 17.7 and 5.9 Hz, H-5), 2.43 (1H, dd, *J* = 17.7 and 11.0 Hz, H-5), 2.35 (3H, s, H-1), 2.34 - 2.26 (2H, m, H-7), 1.85 (1H, ddd, *J* = 14.3, 9.5 and 6.7 Hz, H-10), 1.72 (1H, ddd, *J* = 14.3, 6.6 and 2.6 Hz, H-10), 1.66 (1H, heptd, *J* = 6.5 and 4.8 Hz, H-12), 0.92 (3H, d, *J* = 6.5 Hz, H-13), 0.90 (3H, d, *J* = 6.5 Hz, H-13) ppm. **^{13}C-NMR** (101 MHz, $CDCl_3$): δ 193.4, 139.0, 134.5, 130.2, 125.6, 79.2, 77.3, 73.8, 39.7, 39.5, 35.5, 33.9, 30.4, 21.3, 18.4, 17.2 ppm. **MS (ESI)**: m/z 309 ($M+H^+$), 331 ($M+Na^+$); HRMS: found: ($M+H^+$) 309.1548, ($M+Na^+$) 331.1338, $C_{17}H_{25}O_3S$ requires ($M+H^+$) 309.1519, $C_{17}H_{24}NaO_3S$ requires ($M+Na^+$) 331.1338

(±)-*S*-(*p*-Tolyl) 2-((2*R*,4*R*,6*R*)-4-hydroxy-6-phenyltetrahydro-2H-pyran-2-yl)ethanethioate (10b)

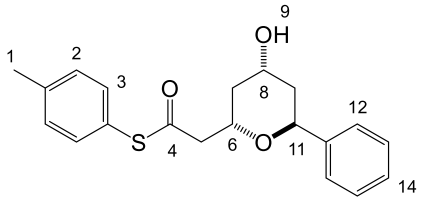

(5*R*,7*S*,*E*)-*S*-*p*-Tolyl 5,7-dihydroxy-7-phenylhept-2-enethioate (**9b**) (20.0 mg, 0.058 mmol) was dissolved in dry THF (1.5 mL, 0.04 M). A solution of acetic acid (0.004 mmol, 0.06 eq.) and tetrabutylammonium fluoride (0.017 mmol, 0.3 eq.) was added to the reaction mixture over a period of 3 minutes at -10 °C under N_2 atmosphere. The reaction mixture was

treated after 1.5 and 2 hours with additional solution (0.12 mL) of acetic acid and tetrabutylammonium fluoride. After stirring for 3 hours at -10 °C the reaction was quenched with saturated aqueous solution of $NaHCO_3$ (3 mL). The phases were separated and the aqueous layer was extracted with diethyl ether (3 × 3 mL). The combined organics were dried over $MgSO_4$, filtered, concentrated *in vacuo* and purified by flash silica gel column chromatography (20 to 50 % ethyl acetate in petroleum ether) to yield **10b** (7.9 mg, 0.023 mmol, 40% yield) as a yellow oil.

IR (film): ν_{max} 3448, 2954, 2924, 2852, 1734, 1630, 1495, 1391, 1243, 1065 cm^{-1}. **^{1}H-NMR** (400 MHz, $CDCl_3$): δ = 7.40 – 7.27 (m, 7H, Ar-H), 7.14 (2H, d, *J* = 7.7 Hz, H-3), 4.96 (1H, dd, *J* = 6.9 and 6.9 Hz, H-11), 4.24 – 4.12 (1H, m, H-6), 3.35 – 3.23 (1H, m, H-8), 2.87 (1H, dd, *J* = 17.8 and 5.8 Hz, H-5), 2.42 (1H, dd, *J* = 17.8 and 10.9 Hz, H-5), 2.34 (3H, s, H-1), 2.30 – 2.13 (2H, m, H-10), 1.91 (1H, ddd, *J* = 13.9, 6.6 and 4.3 Hz, H-7) 1.63 (1H, ddd, *J* = 13.9, 11.7 and 11.7 Hz, H-7) ppm. **^{13}C-NMR** (101 MHz, $CDCl_3$): δ 169.1, 143.3, 138.9, 134.5, 130.0, 128.7, 128.5, 127.6, 126.0, 77.7, 77.2, 71.2, 44.6, 39.3, 36.6, 21.2 ppm. **MS (ESI)**: m/z 365 ($M+Na^+$); HRMS: found: ($M+Na^+$) 365.1198, $C_{20}H_{22}NaO_3S$ requires ($M+Na^+$) 365.1182

(±)-*S*-*p*-Tolyl 2-((2*S*,4*R*,6*R*)-6-heptyl-4-hydroxytetrahydro-2H-pyran-2-yl)ethanethioate (10c)

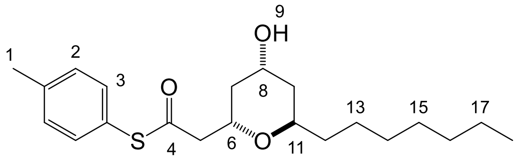

Thioester **9c** (10.0 mg, 0.028 mmol) was dissolved in dry THF (0.3 mL). A solution of acetic acid (0.002 mmol, 6 mol%) and tetrabutylammonium fluoride (0.008 mmol, 30 mol%) was added to the reaction mixture over a period of 3 minutes at 0 °C under N_2 atmosphere. After stirring for 5 hours at 0 °C the reaction was quenched with saturated aqueous solution of $NaHCO_3$ (2 mL). The phases were separated and the aqueous layer was

extracted with diethyl ether (3 × 2 mL). The combined organics were dried over $MgSO_4$, filtered, concentrated *in vacuo* and purified by flash chromatography (20% ethyl acetate in petroleum ether) on a silica gel column to yield **10c** as a colourless oil (4.8 mg, 48%).

IR (film): υ_{max} 3433, 2928, 2853, 1732, 1460, 1378, 1246, 1052 cm^{-1}; **^{1}H-NMR** (400 MHz, $CDCl_3$) δ 7.35 (2H, d, *J* = 8.0 Hz, H-2), 7.15 (2H, d, *J* = 8.0 Hz, H-3), 4.46 (1H, dddd, *J* = 11.3, 7.5, 5.8 and 2.7 Hz, H-6), 3.83 – 3.76 (1H, m, H-8), 3.32 – 3.42 (1H, m, H-11), 2.89 (1H, dd, *J* = 17.8 and 5.8 Hz, H-5), 2.43 (1H, dd, *J* = 17.8, 11.3 Hz, H-5), 2.35 (3H, s, H-1), 1.87 (1H, ddd, *J* = 14.6, 8.6 and 7.5 Hz, H-7), 1.72 (1H, ddd, *J* = 14.5, 5.6 and 3.6 Hz, H-10), 1.59 (1H, ddd, *J* = 13.7, 11.7 and 11.7 Hz, H-12), 1.59 (1H, ddd, *J* = 13.7, 11.7 and 11.7 Hz, H-12), 1.48 – 1.41 (2H, m, H-7 + H-10), 1.34 – 1.20 (10H, m, H-13 + H-14 + H-15 + H-16 + H-17), 0.88 (3H, t, *J* = 6.6 Hz, H-18) ppm. **^{13}C-NMR** (101 MHz, $CDCl_3$): δ 193.3, 138.9, 134.5, 130.1, 127.7, 78.6, 77.2, 69.1, 42.9, 37.7, 36.7, 35.5, 31.7, 29.5, 29.2, 25.4, 22.6, 21.2, 14.1 ppm. **MS (ESI)**: 387 ($M+Na^+$); HRMS: found: ($M+Na^+$) 387.1963, $C_{21}H_{32}NaO_3S$ requires ($M+Na^+$) 387.1964

(±)-*S*-*p*-Tolyl 2-((2*R*,4*S*,6S)-4-hydroxy-6-isopropyltetrahydro-2H-pyran-2-yl)ethanethioate (11a)

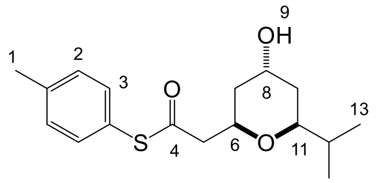

Thioester **9a** (8.0 mg, 0.026 mmol) was dissolved in DCM (0.5 mL) and water (0.05 mL) was added. The mixture was cooled down to 0 °C and trifluoroacetic acid (0.45 mL) was added dropwise. After stirring for 5.5 hours at room temperature the reaction was quenched with saturated aqueous solution of $NaHCO_3$ (2 mL) and diluted with DCM (2 mL). The aqueous layer was extracted with DCM (2 × 2 mL). The combined organics were dried over $MgSO_4$, filtered, concentrated *in vacuo* and purified by flash chromatography (20% ethyl

acetate in petroleum ether) on a silica gel column to yield **11a** as a colourless oil (5.3 mg, 66%).

IR (film): υ_{max} 3446, 2959, 2924, 2877, 2851, 1706, 1467, 1435, 1381, 1066 cm^{-1}; **^{1}H-NMR** (400 MHz, $CDCl_3$) δ 7.28 (2H, d, *J* = 8.1 Hz, H-2), 7.21 (2H, d, *J* = 8.1 Hz, H-3), 4.32 – 4.22 (2H, m, H-6 + H-8), 3.45 (1H, ddd, *J* = 11.9, 7.0 and 1.9 Hz, H-11), 2.86 (1H, dd, *J* = 14.5 and 8.2 Hz, H-5), 2.65 (1H, dd, *J* = 14.5 and 4.9 Hz, H-5), 2.37 (3H, s, H-1), 1.73 – 1.68 (1H, m, H-7), 1.66 - 1.58 (1H, m, H-10), 1.54 – 1.41 (3H, m, H-7 + H-10 + H-12), 0.96 (3H, d, *J* = 6.7 Hz, H-13), 0.88 (3H, d, *J* = 6.8 Hz, H-13) ppm. **^{13}C-NMR** (101 MHz, $CDCl_3$): δ 193.4, 139.6, 134.4, 130.0, 124.4, 77.2, 69.0, 64.7, 49.9, 38.3, 35.3, 33.1, 30.3, 21.3, 18.5 ppm. **MS (ESI)**: 331 ($M+Na^+$); HRMS: found: ($M+Na^+$) 331.1333, $C_{17}H_{24}NaO_3S$ requires ($M+Na^+$) 331.1338

(±)-*S*-(*p*-Tolyl) 2-((2*R*,4*R*,6*S*)-4-hydroxy-6-phenyltetrahydro-2H-pyran-2-yl) ethanethioate (11b)

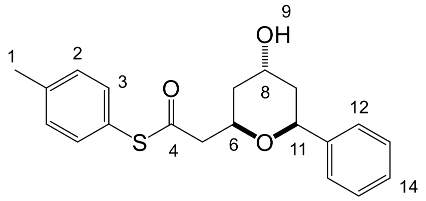

Diol **9b** (10 mg, 0.029 mmol) was dissolved in DCE (2.0 mL) and CSA (20.4 mg, 0.086 mmol, 3 eq.) was added in one portion. The reaction was heated to 80 ºC and left to stir for 20 hours. The reaction was quenched with Et_3N, washed with $NaHCO_3$ (2 × 5 mL) and brine (2 × 5 mL), dried over $MgSO_4$, filtered and concentrated *in vacuo*. The residue was purified by flash silica gel column chromatography (50% diethyl ether in petroleum ether) to yield **11b** as a colourless oil (7.4 mg, 74%).

IR (film): ν_{max} 3435, 2924, 2876, 1705, 1495, 1452, 1381, 1217, 1062 cm^{-1}. **^{1}H NMR** (400 MHz, $CDCl_3$): δ 7.40 – 7.22 (7H, m, Ar-H), 7.22 – 7.16 (2H, d, *J* = 8.0 Hz, H-3), 4.91 (1H, dd, *J* = 11.8, 2.2 Hz, H-11), 4.50 (1H, dddd, *J* = 7.2, 6.9, 6.0 and 2.2 Hz, H-6), 4.40 – 4.32 (1H, m, H-8), 2.99 (1H, dd, *J* = 14.8 and 6.9 Hz, H-5), 2.78 (1H, dd, *J* = 14.8 and 6.0

Hz, H-5), 2.36 (3H, s, H-1), 1.95 (1H, ddd, *J* = 13.9, 7.2 and 2.3 Hz, H-7), 1.84 (1H, ddd, *J* = 13.9, 5.2 and 2.2 Hz, H-7), 1.71 (2H, m, H-10) ppm. **^{13}C-NMR** (101 MHz, $CDCl_3$): δ 195.7, 142.6, 139.7, 134.5, 130.1, 128.4, 127.4, 125.8, 124.3, 73.6, 69.2, 64.7, 49.8, 40.0, 38.0, 21.4 ppm. **MS (ESI)**: m/z 365 ($M+Na^+$); HRMS: found: ($M+Na^+$) 365.1164, $C_{20}H_{22}NaO_3S$ requires ($M+Na^+$) 365.1182

(±)-*S*-*p*-Tolyl 2-((2*R*,4*R*,6*R*)-6-heptyl-4-hydroxytetrahydro-2H-pyran-2-yl)ethanethioate (11c)

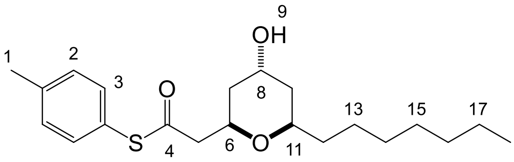

Thioester **9c** (10.0 mg, 0.028 mmol) was dissolved in DCM (0.5 mL) and water (0.05 mL) was added. The mixture was cooled down to 0 °C and trifluoroacetic acid (0.45 mL) was added over a period of 3 minutes. After stirring for 5.5 hours at room temperature the reaction was quenched with saturated aqueous solution of $NaHCO_3$ (2 mL) and diluted with DCM (2mL). The aqueous layer was extracted with DCM (2 × 2 mL). The combined organics were dried over $MgSO_4$, filtered, concentrated *in vacuo* and purified by flash chromatography (20% ethyl acetate in petroleum ether) on a silica gel column to yield **11c** as a colourless oil (6.5 mg, 65%).

IR (film): υ_{max} 3419, 2921, 2857, 1708, 1468, 1375, 1099, 1070 cm^{-1}; **^{1}H-NMR** (400 MHz, $CDCl_3$) δ 7.28 (2H, d, *J* = 8.1 Hz, H-2), 7.21 (2H, d, *J* = 8.1 Hz, H-3), 4.33 – 4.24 (2H, m, H-6 + H-8), 3.80 – 3.71 (1H, m, H-11), 2.87 (1H, dd, *J* = 14.6, 8.1 Hz, H-5), 2.64 (1H, dd, *J* = 14.6, 5.3 Hz, H-5), 2.37 (3H, s, H-1), 1.73 (1H, ddd, *J* = 13.7, 4.9, 2.2 Hz, H-7), 1.65 (1H, ddd, *J* = 14.0, 4.9, 2.2 Hz, H-10), 1.57 – 1.41 (4H, m, H-7 + H-10 + H-12), 1.33 – 1.18 (10H, m, H-13 + H-14 + H-15 + H-16 + H-17), 0.87 (3H, t, *J* = 6.9 Hz, H-18) ppm. **^{13}C-NMR** (101 MHz, $CDCl_3$): δ 195.7, 139.6, 134.4, 130.0, 124.3, 71.9, 68.9, 64.6, 49.9, 38.4, 36.4, 31.8, 29.6, 29.3, 25.5, 22.7, 21.2, 14.1 ppm. **MS (ESI)**: m/z 365 ($M+H^+$) 387 ($M+Na^+$);

HRMS: found: $(M+H^+)$ 365.2147, $(M+Na^+)$ 387.1967, $C_{21}H_{33}O_3S$ requires $(M+H^+)$ 365.2145, $C_{21}H_{32}NaO_3S$ requires $(M+Na^+)$ 387.1964

(±)-*S*-*p*-Tolyl 2-((2*R*,6*S*)-6-isopropyltetrahydro-2H-pyran-2-yl)ethanethioate (21a)

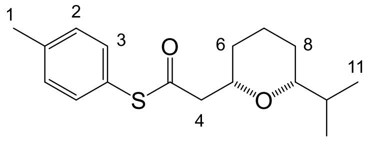

TBAF conditions: (*E*)-*S*-*p*-tolyl 7-hydroxy-8-methylnon-2-enethioate (**20a**) (18.0 mg, 0.062 mmol) was dissolved in dry THF (0.5 mL, 0.1 M). A solution of acetic acid (0.004 mmol, 0.06 eq.) and tetrabutylammonium fluoride (0.019 mmol, 0.3 eq.) was added over 3 minutes to the reaction mixture at -10 °C under N_2 atmosphere. After stirring for 1 hour at -10 °C, the reaction was quenched with saturated aqueous solution of $NaHCO_3$ (3 mL). The phases were separated and the aqueous layer was extracted with diethyl ether (3 × 2 mL). The combined organics were dried over $MgSO_4$, filtered, concentrated *in vacuo* and purified by flash silica gel column chromatography (5 % ethyl acetate in petroleum ether) to yield **21a** (5.0 mg, 0.017 mmol, 27% yield) as a yellow oil.

Acidic conditions: (*E*)-*S*-*p*-tolyl 7-hydroxy-8-methylnon-2-enethioate (**20a**) (17.0 mg, 0.058 mmol) was dissolved in DCM (1.0 mL, 0.05 M) and water (0.1 mL, 0.5 M) was added. The mixture was cooled down to 0 °C and trifluoroacetic acid (0.9 mL, 0.06 M) was added over 3 minutes. After stirring for 5.5 hours at room temperature the reaction was quenched with saturated aqueous solution of $NaHCO_3$ (2 mL) and diluted with DCM (2 mL). The aqueous layer was extracted with DCM (2 × 2 mL). The combined organics were dried over $MgSO_4$, filtered, concentrated *in vacuo* and purified by flash silica gel column chromatography (5 % ethyl acetate in petroleum ether) to yield **21a** (8.0 mg, 0.027 mmol, 47% yield) as a yellow oil.

IR (film, NaCl): ν_{max} 2887, 2816, 1681, 1471, 1435, 1357, 1056, 1032, 981, 794 cm^{-1}. **^{1}H-NMR** (400 MHz, $CDCl_3$) δ 7.28 (2H, d, *J* = 8.3 Hz, H-2), 7.21 (2H, *J* = 8.3 Hz, H-

3), 3.80 (1H, dddd, J = 10.9, 8.1, 4.9 and 2.0 Hz, H-5), 2.96 (1H, ddd, J = 11.1, 7.0 and 1.8 Hz, H-9), 2.87 (1H, dd, J = 14.4 and 8.1 Hz, H-4), 2.65 (1H, dd, J = 14.4, 4.9 Hz, H-4), 2.37 (3H, s, H-1), 1.85 (1H, dhept, J = 7.0 and 6.8, H-10), 1.68 – 1.41 (6H, m, H-6 + H-7 + H-8), 0.95 (3H, d, J = 6.8 Hz, H-11), 0.87 (3H, d, J = 6.8 Hz, H-11) **^{13}C-NMR** (101 MHz, $CDCl_3$) δ 196.3, 139.7, 134.6, 130.1, 124.6, 83.4, 75.0, 50.5, 33.5, 31.5, 28.2, 23.7, 21.5, 18.8 ppm. **MS** (ESI): m/z 293 ($M+H^+$), 315 ($M+Na^+$); HRMS: found: ($M+H^+$) 293.1572, ($M+Na^+$) 315.1386; $C_{17}H_{25}O_2S$ requires ($M+H^+$) 293.1575, $C_{17}H_{24}NaO_2S$ requires ($M+Na^+$) 315.1395

(±)-*S*-*p*-Tolyl 2-((2*R*,6*S*)-6-phenyltetrahydro-2H-pyran-2-yl)ethanethioate (21b)

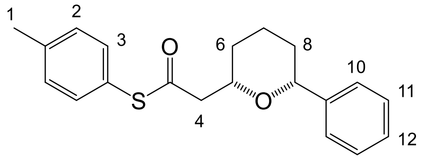

TBAF conditions: (*E*)-*S*-*p*-tolyl 7-hydroxy-7-phenylhept-2-enethioate (**20b**) (7.5 mg, 0.023 mmol) was dissolved in dry THF (0.5 mL, 0.05 M). A solution of acetic acid (0.0010 mmol, 0.06 eq.) and tetrabutylammonium fluoride (0.0070 mmol, 0.3 eq.) in THF (0.5 mL) was added over 3 minutes to the reaction mixture at -10 °C under N_2 atmosphere. After stirring for 45 minutes at -10 °C the reaction was quenched with saturated solution of $NaHCO_3$ (1 mL). The phases were separated and the aqueous layer was extracted with diethyl ether (3 × 1 mL). The combined organics were dried over $MgSO_4$, filtered, concentrated *in vacuo* to yield **21b** (4.0 mg, 0.013 mmol, 53% yield) as a crude yellow oil.

Acidic conditions: (*E*)-*S*-*p*-tolyl 7-hydroxy-7-phenylhept-2-enethioate (**20b**) (7.5 mg, 0.023 mmol) was dissolved in DCM (0.4 mL, 0.06 M) and water (0.4 mL, 0.06 M) was added. The mixture was cooled down to 0 °C and trifluoroacetic acid (0.3 mL, 0.08 M) was added over 3 minutes. After stirring for 4 hours at room temperature the reaction was quenched with saturated aqueous $NaHCO_3$ solution and the aqueous layer was extracted with DCM (2 × 1 mL). The combined organics were dried over $MgSO_4$, filtered, concentrated *in*

vacuo and purified by flash silica gel column chromatography (5 % ethyl acetate in petroleum ether) to yield **21b** (4.2 mg, 0.013 mmol, 56% yield) as a yellow oil.

IR (film, NaCl): ν_{max} 2882, 2811, 1680, 1470, 1430, 1241, 1073, 793, 734, 687 cm^{-1}. **^{1}H-NMR** (400 MHz, $CDCl_3$) δ 7.40 – 7.27 (m, 7H, Ar-H), 7.20 (2H, d, *J* = 7.9 Hz, H-3), 4.42 (1H, dd, *J* = 11.3 and 2.1 Hz, H-9), 4.08 – 3.98 (1H, m, H-5), 3.01 (1H, dd, *J* = 14.7, 6.9 Hz, H-4), 2.79 (1H, dd, *J* = 14.7, 5.9 Hz, H-4), 2.37 (3H, s, H-1), 2.00 – 1.84 (2H, m, H-8), 1.80 – 1.72 (2H, m, H-6), 1.71 – 1.63 (2H, m, H-7) ppm. **^{13}C-NMR** (101 MHz, $CDCl_3$) δ 196.0, 139.8, 134.7, 130.1, 128.3, 127.3, 125.9, 124.4, 79.7, 75.1, 50.3, 33.1, 31.0, 29.9, 23.9, 21.5 ppm. **MS** (ESI): m/z 327 ($M+H^+$), 349 ($M+Na^+$); HRMS: found: ($M+H^+$) 327.1413, ($M+Na^+$) 349.1233 $C_{20}H_{23}O_2S$ requires ($M+H^+$) 327.1419, $C_{20}H_{23}NaO_2S$ requires ($M+Na^+$) 349.1238

(±)-*S*-*p*-Tolyl 2-((2*R*,6*R*)-6-octyltetrahydro-2H-pyran-2-yl)ethanethioate (21c)

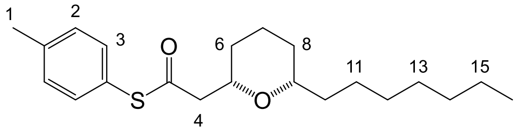

TBAF conditions: (*E*)-*S*-*p*-tolyl 7-hydroxypentadec-2-enethioate (**20c**) (18.6 mg, 0.051 mmol) was dissolved in dry THF (0.5 mL, 0.1 M). A solution of acetic acid (0.003 mmol, 0.06 eq.) and tetrabutylammonium fluoride (0.015 mmol, 0.3 eq.) was added over 3 minutes to the reaction mixture at -10 °C under N_2 atmosphere. After stirring for 1.5 hours at -10 °C the reaction was quenched with saturated aqueous solution of $NaHCO_3$ (3 mL). The phases were separated and the aqueous layer was extracted with diethyl ether (3 × 2 mL). The combined organics were dried over $MgSO_4$, filtered, concentrated *in vacuo* and purified by flash silica gel column chromatography (5 % ethyl acetate in petroleum ether) to yield **21c** (4.6 mg, 0.018 mmol, 25% yield) as a yellow oil.

Acidic conditions: (*E*)-*S*-*p*-tolyl 7-hydroxypentadec-2-enethioate (**20c**) (19.0 mg, 0.052 mmol) was dissolved in DCM (1.0 mL, 0.05 M) and water (0.1 mL, 0.5 M) was added.

The mixture was cooled down to 0 °C and trifluoroacetic acid (0.9 mL, 0.06 M) was added over 3 minutes. After stirring for 5.5 hours at room temperature the reaction was quenched with saturated aqueous solution of $NaHCO_3$ (2 mL) and diluted with DCM (2mL). The aqueous layer was extracted with DCM (2 × 2 mL). The combined organics were dried over $MgSO_4$, filtered, concentrated *in vacuo* to yield **21c** (6.7 mg, 0.019 mol, 36% yield) as a yellow oil.

IR (film, NaCl): ν_{max} 2881, 2811, 1754, 1681, 1434, 1202, 1146, 1071, 1055, 794 cm^{-1}. **^{1}H-NMR** (400 MHz, $CDCl_3$) δ 7.28 (2H, d, *J* = 8.0 Hz, H-2), 7.21 (2H, d, *J* = 8.0 Hz, H-3), 3.87 – 3.75 (1H, m, H-5), 3.33 – 3.21 (1H, m, H-9), 2.88 (1H, dd, *J* = 14.6, 7.8 Hz, H-4), 2.66 (1H, dd, *J* = 14.6, 5.3 Hz, H-4), 2.37 (3H, s, CH_3), 1.70 – 1.49 (6H, m, H-6 + H-8 + H-10), 1.35 – 1.20 (12H, m, H-7 + H-11 + H-12 + H-13 + H-14 + H-15), 0.87 (3H, t, *J* = 6.8 Hz, H-16) ppm. **^{13}C-NMR** (101 MHz, $CDCl_3$) δ 196.1, 139.7, 134.5, 130.0, 124.5, 78.3, 74.8, 50.7, 36.6, 32.0, 31.4, 29.8, 29.4, 25.7, 23.6, 22.8, 22.5 21.4, 14.2 ppm. **MS** (ESI): m/z 363 ($M+H^+$), 385 ($M+Na^+$); HRMS: found: ($M+H^+$) 363.2345, ($M+Na^+$) 385.2175; $C_{22}H_{35}O_2S$ requires ($M+H^+$) 363.2358, $C_{22}H_{34}NaO_2S$ requires ($M+Na^+$) 385.2177

(±)-*S*-*p*-Tolyl 2-((2*S*,4*S*,6*R*)-6-isopropyl-4-methoxytetrahydro-2H-pyran-2-yl)ethanethioate (23)

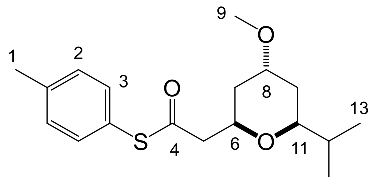

Thioester **22** (5.0 mg, 0.015 mmol) was dissolved in DCM (0.5 mL) and water (0.05 mL) was added. The mixture was cooled down t17o 0 °C and trifluoroacetic acid (0.45 mL) was added over a period of 3 minutes. After stirring for 5.5 hours at room temperature the reaction was quenched with saturated aqueous solution of $NaHCO_3$ (2 mL) and diluted with DCM (2mL). The aqueous layer was extracted with DCM (2 × 2 mL). The combined organics were dried over $MgSO_4$, filtered, concentrated *in vacuo* and purified by flash

chromatography (10% ethyl acetate in petroleum ether) on a silica gel column to yield **23** as a colourless oil (2.4 mg, 48%).

IR (film): υ_{max} 2954, 2929, 2875, 2853, 1708, 1464, 1381, 1347, 1222, 1170, 1094 cm^{-1}; **^{1}H-NMR** (400 MHz, $CDCl_3$) δ 7.30 (2H, d, *J* = 7.9 Hz, H-2), 7.21 (2H, d, *J* = 7.9 Hz, H-3), 4.21 – 4.13 (1H, m, H-6), 3.69 – 3.64 (1H, m, H-8), 3.42 – 3.28 (4H, m, H-11 + H-9), 2.84 (1H, dd, *J* = 14.4 and 8.2 Hz, H-5), 2.64 (1H, dd, *J* = 14.4 and 5.0 Hz, H-5), 2.36 (3H, s, H-1), 1.92 – 1.90 (1H, m, H-7), 1.88 (1H, ddd, *J* = 7.2, 2.3 and 2.3 Hz, H-10), 1.85 – 1.82 (1H, m, H-7), 1.65 – 1.55 (2H, m, H-10 + H-12), 0.95 (3H, d, *J* = 6.6 Hz, H-13), 0.88 (3H, d, *J* = 6.8 Hz, H-13) ppm. **^{13}C-NMR** (101 MHz, $CDCl_3$): δ 193.2, 139.5, 134.4, 130.0, 123.6, 77.2, 73.5, 69.3, 56.0, 50.0, 34.6, 33.1, 32.3, 22.7, 21.3, 18.5 ppm. **MS (ESI)**: m/z 323 ($M+H^+$), 345 ($M+Na^+$); HRMS: found: ($M+H^+$) 323.1678, ($M+Na^+$) 345.1473, $C_{18}H_{27}O_3S$ requires ($M+H^+$) 323.1675, $C_{18}H_{26}NaO_3S$ requires ($M+Na^+$) 345.1495.

Diospongin B (24)

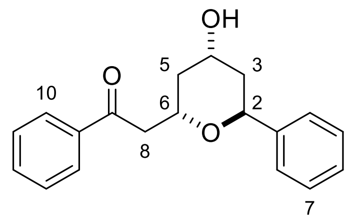

Thioester **10b** (25mg, 0.073 mmol) was dissolved in 2mL THF, under N_2, phenyllithium (0.1mL, 0.16 mmol, 2.2 eq.) was added at -78 ºC for 5hours. The reaction mixture was quenched with trimethylsilyl chloride (0.09 mL, 0.73 mmol, 10 eq.) and was diluted with diethyl ether, washed with saturated aqueous $NaHCO_3$ (3 x 5 mL) solution and brine (3 x 5 mL). The combined organic layers were dried over $MgSO_4$, filtered and concentrated *in vacuo* to give a yellow oil. The residue was purified by flash silica gel column chromatography (50 : 50 / ethyl acetate : hexane) to yield **25** as a colourless oil (12 mg, 55.5 %). **IR** (film): ν_{max} 3375, 2916, 2846, 1557, 1411, 1129 cm-1. **^{1}H NMR** (400 MHz,

$CDCl_3$): δ 7.98 (2H, dd, J = 8.0, 1.7 Hz, Ar), 7.58 (1H, tt, J = 7.1, 1.3 Hz, Ar), 7.47 (2H, td, J = 7.6, 2.2 Hz, Ar), 7.37-7.3 (5H, m, Ar), 5.19 (1H, t, J = 4.3 Hz, H-2), 4.23 (1H, dddd, J = 9.5, 7.1, 6.0, 3.0 Hz, H-6), 4.03 (1H, dddd, J = 12.4, 9.9, 5.5, 4.5 Hz, H-4), 3.46 (1H, dd, J = 15.8, 7.1 Hz, H-8), 3.18 (1H, dd, J = 15.8, 6.0 Hz, H-8), 2.52 (1H, ddd, J = 13.4, 5.5, 3.8 Hz, H-3eq), 2.06 (1H, ddd, J = 12.4, 4.5, 3.0 Hz, H-5eq), 1.92 (1H, ddd, J = 13.4, 9.9, 5.2 Hz, H-3ax), 1.51 (1H, dt, J = 12.4, 9.5 Hz, H-5ax)ppm; **^{13}C NMR** (101 MHz, $CDCl_3$): δ 198.4 (C-9), 140.3, 137.3, 133.3, 128.7, 128.6, 127.2 (Ar), 126.4 (Ar), 125.9 (Ar), 72.4 (C-2), 66.9 (C-6), 64.4 (C-4), 44. 7, 40.2 (C-5) and 36.8 (C-3) ppm; **MS (ESI)**: *m/z* = 319.1305 [M+Na$^+$].

Diospongin A (25)

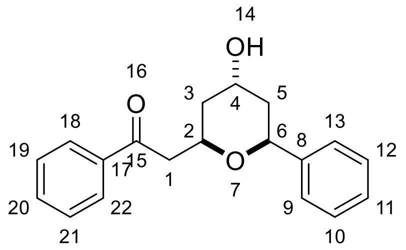

To a solution of thioester **11b** (50.0 mg, 0.16 mmol), $PhB(OH)_2$ (57.68 mg, 0.47 mmol, 3 eq.), $Pd_2(dba)_3$ (14.43 mg, 0.016 mmol, 10 mol %), and CuTC (90.21 mg, 0.47 mmol, 3 eq.) in dry THF (5.0 mL) was added a solution of $(EtO)_3P$ (0.0021 mL, 8 mol %) in dry THF (0.5 mL), and the mixture was stirred at room temperature for 2 hour and 30min. The reaction mixture was diluted with diethyl ether, washed with saturated aqueous $NaHCO_3$ (3 x 5 mL) solution and brine (3 x 5 mL), dried with $MgSO_4$, filtered and concentrated *in vacuo*. The residue was purified by flash silica gel column chromatography (50 : 50 / ethyl acetate : hexane) to yield **26** as a colourless oil (41.5 mg, 96%). **IR** (film): ν_{max} 3439, 3065, 3028, 2917, 2850, 1681, 1449, 1058, 751, 697 cm^{-1}. **^{1}H NMR** (400 MHz, $CDCl_3$): δ 7.99 (2H, dd, J = 8.2, 1.2 Hz, Ar-H), 7.56 (1H, t, J = 7.6 Hz, Ar-H), 7.46 (2H, t, J = 7.6 Hz, Ar-H), 7.33-7.18 (5H, m, Ar-H), 4.93 (1H, dd, J = 12.0, 2.1 Hz, H-6), 4.65 (1H, dt, J = 11.1, 5.6 Hz, H-2), 4.35 (1H, p, J = 4.2 Hz, H-4), 3.42 (1H, dd, J = 16.0, 5.7 Hz, H-1), 3.07 (1H, dd, J = 16.0, 6.9 Hz, H-1), 1.96 (2H, d, J = 13.3 Hz, H-3+H-5) 1.84-1.51 (2H, m, H-3+H-5). **^{13}C NMR** (101 MHz,

CDCl3): δ 198.61, 142.79, 137.32, 133.27, 128.66, 128.45, 128.04, 127.36, 73.93, 69.15, 64.72, 45.26, 40.10, 38.54. **MS (ESI)**: *m*/*z* = 319.1305 [M+Na$^+$].

Synthesis of Cyclization Substrates

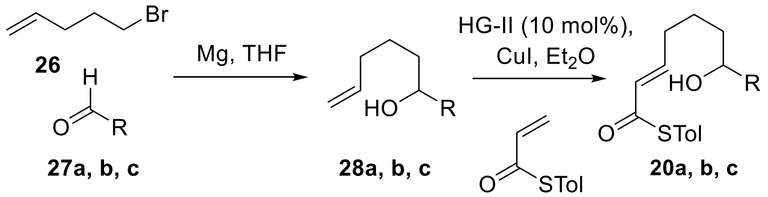

**27a, b, c**	**28a, b, c**	**20a, b, c**
**a**, R=iPr; R=Ph;	**a**, 26%	**a**, 88%
**b**, R=Ph;	**b**, 72%	**b**, 67%
**c**, R=C_7H_{11}	**c**, 53%	**c**, 71%

(±)-2-methyloct-7-en-3-ol (28a)

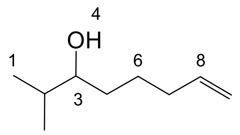

5-Bromo-1-pentene (**26**) (894 mg, 6.00 mmol) in dry THF (2.0 mL) was added over a period of 5 minutes to a suspension of magnesium turnings (146 mg, 6.00 mmol) in dry THF (8.5 mL) under N_2 atmosphere at 0 °C. After stirring for 40 minutes at room temperature the Grignard reagent (5.65 mL, 1.5 eq.) was added over a period of 5 minutes to a solution of isobutyraldeyhde (144 mg, 2.0 mmol) in dry THF (1 mL) at 0 °C. After stirring for 1 h the reaction was quenched with ice water (6 mL) and treated with sulfuric acid (5 M, 1.5 mL) until the magnesium salt dissolved. After seperation of the phases the aqueous layer was extracted with diethyl ether (2 × 5 mL). The combined organic phases were dried over $MgSO_4$, filtered and concentrated *in vacuo* to give a yellow oil, which was further purified by flash silica gel column chromatography (10 % ethyl acetate in petroleum ether) to yield **28a** as a yellow oil (73 mg, 26 %).

IR (film, NaCl): ν_{max} 3345, 3031, 2915, 2830, 1686, 1616, 1446, 1364, 1347, 1250, 979, 896 cm^{-1}. **^{1}H-NMR** (400 MHz, $CDCl_3$) δ 5.82 (1H, dddd, *J* = 17.0, 10.2, 6.7 and 6.7 Hz, H-8), 5.01 (1H, dddd, *J* = 17.0, 2.0, 1.6 and 1.6 Hz, H-9), 4.95 (1H, dddd, *J* = 10.2, 2.0, 1.2

and 1.2 Hz, H-9), 3.37 (1H, ddd, *J* = 8.5, 5.1 and 3.5 Hz, H-3), 2.13 – 2.05 (1H, m, H-2), 1.71 – 1.55 (2H, m, H-7), 1.53 – 1.43 (2H, m, H-5), 1.29 – 1.03 (2H, m, H-6), 0.92 (3H, d, *J* = 4.3 Hz, H-1), 0.90 (3H, d, *J* = 4.3 Hz, H-1) ppm. **^{13}C-NMR** (101 MHz, $CDCl_3$) δ 138.9, 127.8, 114.7, 76.7, 33.9, 33.6, 25.4, 19.0, 17.2 ppm. **MS** (ESI): m/z 165 ($M+Na^+$); HRMS: found: ($M+Na^+$) 165.1250 $C_9H_{18}NaO$ requires ($M+Na^+$) 165.1255

(±)-1-phenylhex-5-en-1-ol (28b)

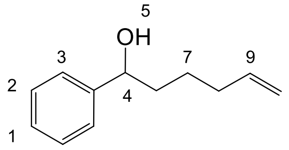

5-Bromo-1-pentene (**26**) (894 mg, 6.00 mmol) in dry THF (2 mL) was added to a suspension of magnesium turnings (146 mg, 6.00 mmol) in dry THF (8.5 mL) over a period of 5 minutes under N_2 atmosphere at 0 °C. After stirring for 1 h at room temperature the Grignard reagent (3.34 mL, 1.2 eq.) was added to a solution of benzaldehyde (160 mg, 1.5 mmol) in dry THF (1 mL) over a period of 5 minutes at 0 °C. After a further 1 h Grignard reagent (1.41 mL, 0.5 eq.) was added over 5 minutes at the same temperature. After stirring for 1.5 h the reaction was quenched with ice water (6 mL) and treated with sulfuric acid (5 M, 1.5 mL) until the magnesium salt dissolved. After separation of the phases the aqueous layer was extracted with diethyl ether (2 × 4 mL). The combined organic phases were dried over $MgSO_4$, filtered and concentrated *in vacuo* to give a yellow oil, which was further purified by flash silica gel column chromatography (5 to 15 % ethyl acetate in petroleum ether) to yield **28b** as a yellow oil (189 mg, 72 %).

IR (film, NaCl): ν_{max} 3321, 3017, 2888, 2816, 1431, 1047, 1012, 981, 897, 750, 690 cm^{-1}. **^{1}H-NMR** (400 MHz, $CDCl_3$) δ 7.37 – 7.31 (3H, m, H-1 + H-2), 7.30 – 7.27 (2H, m, H-3), 5.78 (1H, dddd, *J* = 17.0, 10.2, 6.7 and 6.7 Hz, H-9), 4.99 (1H, ddd, *J* = 17.0, 3.5 and 1.9 Hz, H-10), 4.94 (1H, dddd, *J* = 10.2, 1.9 and 1.2 Hz, H-10) 4.68 (1H, dd, *J* = 7.5 and 5.8 Hz, H-4), 2.13 – 2.02 (2H, m, H-8), 1.88 – 1.65 (2H, m, H-5), 1.53 (1H, ddddd, *J* = 10.8, 7.4, 7.4,

7.4 and 5.3 Hz, H-7), 1.45 – 1.30 (1H, m, H-7) ppm. **^{13}C-NMR** (101 MHz, $CDCl_3$) δ 144.9, 138.7, 128.5, 127.6, 126.0, 114.8, 74.6, 38.6 , 33.7, 25.2 ppm.

(±)-Tetradec-1-en-6-ol (28c)

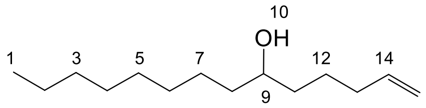

5-Bromo-1-pentene (**26**) (894 mg, 6.00 mmol) in dry THF (2.0 mL) was added over a period of 5 minutes to a suspension of magnesium turnings (146 mg, 6.00 mmol) in dry THF (8.5 mL) under N_2 atmosphere at 0 °C. After stirring for 40 minutes at room temperature the Grignard reagent (5.65 mL, 1.5 eq.) was added over a period of 5 minutes to a solution of nonanal (284 mg, 2.0 mmol) in dry THF (1 mL) at 0 °C. After stirring for 1 h the reaction was quenched with ice water (6 mL) and treated with sulfuric acid (5 M, 1.5 mL) until the magnesium salt dissolved. After separation of the phases the aqueous layer was extracted with diethyl ether (2 × 5 mL). The combined organic phases were dried over $MgSO_4$, filtered and concentrated *in vacuo* to give a yellow oil, which was further purified by flash silica gel column chromatography (10 % ethyl acetate in petroleum ether) to yield **28c** as a yellow oil (224 mg, 53 %).

IR (film, NaCl): ν_{max} 3290, 3030, 2882, 2812, 1689, 1616, 1437, 1356, 1158, 1109, 1051, 978, 895, 815, 711 cm^{-1}. **^{1}H-NMR** (400 MHz, $CDCl_3$) δ 5.81 (1H, dddd, *J* = 17.0, 10.2, 6.7 and 6.7 Hz, H-14), 5.01 (1H, ddd, *J* = 17.0, 3.6 and 1.9 Hz, H-15), 4.95 (1H, dddd, *J* = 10.2, 1.9, 1.2 and 1.2 Hz, H-15) 3.65 – 3.54 (1H, m, H-9), 2.12 – 2.05 (2H, m, H-13), 1.58 – 1.38 (6H, m, H-8 + H-11 + H-12), 1.36 – 1.21 (12H, m, H-2 + H-3 + H-4 + H-5 + H-6 + H-7), 0.88 (3H, t, *J* = 6.8 Hz, m, H-1) ppm **^{13}C-NMR** (101 MHz, $CDCl_3$) δ 138.9, 114.6, 71.9, 37.60, 36.94, 33.86, 31.96, 29.82, 29.71, 29.40, 25.76, 25.03, 22.78, 14.20. **MS** (ESI): m/z 213 (M+H^{+}), 235 (M+Na^{+}); HRMS: found: (M+H^{+}) 213.2213, (M+Na^{+}) 235.2032 $C_{14}H_{29}O$ requires (M+H^{+}) 213.2218, $C_{14}H_{28}NaO$ requires (M+Na^{+}) 235.2038

S-*p*-Tolyl prop-2-enethioate (29)

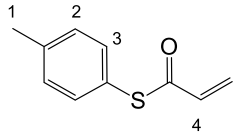

Butylated hydroxytoluene (35.7 mg, 0.162 mmol, 0.01 eq.) and acryloyl chloride (1.34 mL, 16.5 mmol, 1.5 eq.) were dissolved in cyclohexane (7 mL). In a separate flask $NaBH_4$ (13.4 mg, 0.35 mmol, 0.03 eq.) and 4-methylbenzenethiol (1.38 g, 11.0 mmol) were added in order to 15% aq. NaOH (5 mL). This mixture was stirred for 1 hour at room temperature. Under ice-cooling, this mixture was added over a period of 10 minutes to the acryloyl chloride solution. After the reaction mixture was stirred for 30 minutes at 55-60 ºC, it was allowed to cool to room temperature and then extracted with diethyl ether (3 x 4 mL). The combined organic layers were washed with $NaHCO_3$ (3 x 4 mL) and brine (3 x 4 mL), dried over $MgSO_4$, filtered and concentrated *in vacuo* to a yellow oil, which was purified by flash silica gel column chromatography (3% ethyl acetate in *n*-hexane) to yield **29** as a yellow oil (690 mg, 45%). The proton NMR spectrum matched that given in literature.[1]

^{1}H NMR (400 MHz, $CDCl_3$): δ 7.33 (2H, d, *J* = 8.2 Hz, H-2), 7.24 (2H, d, *J* = 8.2 Hz, H-3), 6.46 (1H, dd, *J* = 17.2, 9.6, H-4), 6.38 (1H, dd, *J* = 17.2, 1.6 Hz, H-5), 5.76 (1H, dd, *J* = 9.6, 1.6 Hz, H-5), 2.39 (3H, s, H-1) ppm.

(±)-(*E*)-*S*-*p*-Tolyl 7-hydroxy-8-methylnon-2-enethioate (20a)

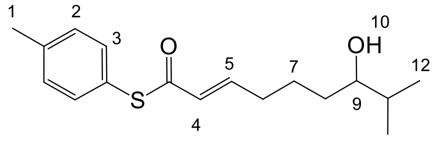

S-p-Tolyl prop-2-enethioate (**29**) (87 mg, 0.489 mmol, 3.0 eq.) and 2-methyloct-7-en-3-ol (**28a**) (24 mg, 0.169 mmol) were dissolved in dry diethyl ether (2 mL) under an N_2 atmosphere at room temperature. To this, copper(I) iodide (5 mg, 0.026 mmol, 0.15 eq.) and Hoveyda-Grubbs 2nd generation catalyst (10 mg, 0.016 mmol, 10 mol%) were then added as solids in one portion and the reaction mixture was heated under reflux. After 2 hours the

reaction mixture was concentrated *in vacuo* and purified by flash silica gel column chromatography (10 % to 15 % ethyl acetate in petroleum ether) to yield **20a** as a brown oil (40.8 mg, 88 %).

IR (film, NaCl): ν_{max} 3379, 2889, 2827, 1680, 1056, 1031, 981, 795 cm^{-1}. **^{1}H-NMR** (400 MHz, $CDCl_3$) δ 7.31 (2H, d, *J* = 8.1 Hz, H-2), 7.22 (2H, d, *J* = 8.1 Hz, H-3), 6.97 (1H, ddd, *J* = 15.5, 6.9 and 6.9 Hz, H-5), 6.19 (1H, ddd, *J* = 15.5, 1.5 and 1.5 Hz, H-4), 3.36 (1H, ddd, *J* = 8.5, 5.1, 3.3 Hz, H-9), 2.37 (3H, s, H-1), 2.30 – 2.21 (2H, m, H-6), 1.72 – 1.60 (m, 2H, H-8), 1.55 – 1.32 (3H, m, H-7 + H-11), 0.92 (3H, d, *J* = 3.6 Hz, H-12), 0.90 (3H, d, *J* = 3.5 Hz, H-12) ppm. **^{13}C-NMR** (101 MHz, $CDCl_3$) δ 188.7, 146.5, 139.7, 134.6, 130.1, 128.1, 83.4, 76.6, 33.7, 32.4, 31.4, 24.6, 21.5, 19.0, 17.2 ppm. **MS** (ESI): m/z 293 ($M+H^+$), 315 ($M+Na^+$); HRMS: found: ($M+H^+$) 293.1570, ($M+Na^+$) 315.1389; $C_{17}H_{25}O_2S$ requires ($M+H^+$) 293.1575, $C_{17}H_{24}NaO_2S$ requires ($M+Na^+$) 315.1395

(±)-(*E*)-*S*-*p*-Tolyl 7-hydroxy-7-phenylhept-2-enethioate (20b)

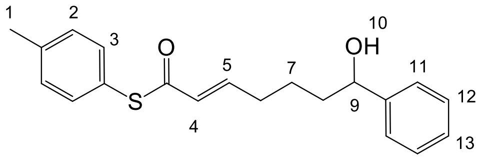

S-p-Tolyl prop-2-enethioate (**29**) (13 mg, 0.073 mmol, 1.1 eq.) and 1-phenylhex-5-en-1-ol (**28b**) (12 mg, 0.068 mmol) were dissolved in dry diethyl ether (1 mL) under an N_2 atmosphere at room temperature. To this, copper (I) iodide (4 mg, 0.021 mmol, 0.3 eq.) and Hoveyda-Grubbs 2nd generation catalyst (10 mg, 0.016 mmol, 20 mol%) were then added as solids in one portion and the reaction mixture was heated under reflux. After 3.5 hours the reaction mixture was concentrated *in vacuo* and purified by flash silica gel column chromatography (10 % to 15 % ethyl acetate in petroleum ether) to yield **20b** as a brown oil (16.9 mg, 67 %).

IR (film, NaCl): ν_{max} 2882, 2812, 1661, 1470, 1429, 1072, 1028, 984, 795, 739, 688 cm^{-1}. **^{1}H-NMR** (400 MHz, $CDCl_3$) δ 7.40 – 7.28 (m, 7H, Ar-H), 7.22 (2H, d, *J* = 8.4 Hz, H-

3), 6.94 (ddd, J = 15.5, 7.1 and 7.1 Hz, 1H, H-5), 6.16 (1H, ddd, J = 15.5, 1.5 and 1.5 Hz, H-4), 4.69 (1H, dd, J = 7.4, 5.6 Hz, H-9), 2.38 (3H, s, H-1), 2.25 (2H, m, H-6), 1.90 – 1.71 (2H, m, H-8), 1.56 – 1.44 (2H, m, H-7) ppm. **^{13}C-NMR** (101 MHz, $CDCl_3$) δ 146.1, 134.7, 130.1, 128.7, 128.3, 75.1, 74.5, 50.3, 38.5, 33.2, 32.3, 31.0, 29.9, 24.3, 23.9, 21.5 ppm. **MS** (ESI): m/z 327 ($M+H^+$), 349 ($M+Na^+$); HRMS: found: ($M+H^+$) 327.1413, ($M+Na^+$) 349.1233 $C_{20}H_{23}O_2S$ requires ($M+H^+$) 327.1419, $C_{20}H_{23}NaO_2S$ requires ($M+Na^+$) 349.1238

(±)-(*E*)-*S*-p-Tolyl 7-hydroxypentadec-2-enethioate (20c)

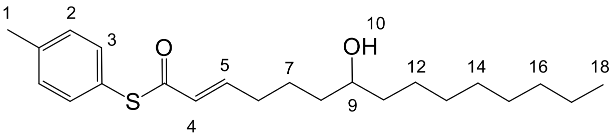

S-p-Tolyl prop-2-enethioate (**29**) (85 mg, 0.478 mmol, 3.0 eq.) and tetradec-1-en-6-ol (**28c**) (34 mg, 0.160 mmol) were dissolved in dry diethyl ether (2 mL) under an N_2 atmosphere at room temperature. To this, copper(I) iodide (4 mg, 0.02 mmol, 0.13 eq.) and Hoveyda-Grubbs 2nd generation catalyst (10 mg, 0.016 mmol, 10 mol%) were then added as solids in one portion and the reaction mixture was heated under reflux. After 2 hours the reaction mixture was concentrated *in vacuo* and purified by flash silica gel column chromatography (10 % to 15 % ethyl acetate in petroleum ether) to yield **20c** as a brown oil (40.9 mg, 71 %).

IR (film, NaCl): ν_{max} 3390, 2882, 2811, 1663, 1607, 1471, 1436, 1355, 1001, 795 cm^{-1}. **^{1}H-NMR** (400 MHz, $CDCl_3$) δ 7.31 (2H, d, J = 8.3 Hz, H-2), 7.22 (2H, d, J = 8.3 Hz, H-3), 6.97 (1H, ddd, J = 15.5, 6.9 and 6.9 Hz, H-5), 6.19 (1H, ddd, J = 15.5, 1.5 and 1.5 Hz, 1H, H-4), 3.64 – 3.55 (1H, m, H-9), 2.37 (3H, s, CH_3), 2.29 – 2.21 (2H, m, H-6), 1.73 – 1.59 (2H, m, H-8), 1.58 – 1.50 (2H, m, H-11), 1.31 – 1.24 (14H, m, H-7 + H-12 + H-13 + H-14 + H-15 + H-16 + H-17), 0.88 (3H, t, J = 6.9 Hz, H-18) ppm. **^{13}C-NMR** (101 MHz, $CDCl_3$) δ 188.7, 146.4, 139.7, 134.7, 130.1, 128.1, 124.2, 71.8, 60.5, 37.7, 36.9, 32.4, 32.0, 29.8, 29.4, 25.8, 24.2, 22.8, 21.5, 14.3 ppm. **MS** (ESI): m/z 363 ($M+H^+$), 385 ($M+Na^+$); HRMS: found:

(M+H$^+$) 363.2352, (M+Na$^+$) 385.2172; $C_{22}H_{35}O_2S$ requires (M+H$^+$) 363.2358, $C_{22}H_{34}NaO_2S$ requires (M+Na$^+$) 385.2177

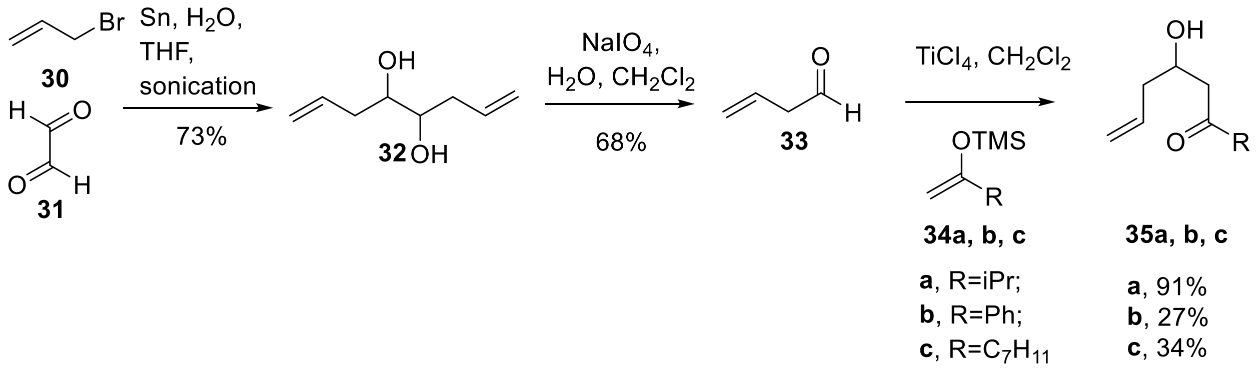

Octa-1,7-diene-4,5-diol (32)

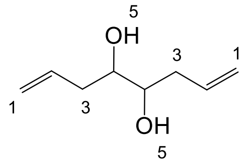

Allyl bromide (7.15 mL, 82.7 mmol, 2.4 eq.) and 40% aqueous glyoxal (3.94 mL, 34.5 mmol) were dissolved in 1:1 THF/H_2O (35 mL). Tin powder (9.82 g, 82.7 mmol, 2.4 eq.) was added and the mixture was sonicated for 6 hours. The reaction was quenched with a 25% KOH solution (28 mL, w:w in H_2O) and diluted with diethyl ether (30 mL). Solid NaCl was added until the aqueous layer was saturated and then the mixture was filtered through celite. The aqueous layer was extracted with diethyl ether (3 x 10 mL) and the combined organics were dried over $MgSO_4$, filtered and concentrated *in vacuo* to give a yellow oil, which was purified by flash silica gel column chromatography (20% to 50% ethyl acetate in petroleum ether) to yield **32** as a yellow oil (3.04 g, 62%). The proton NMR spectrum matched that given in literature.[2]

^{1}H NMR (400 MHz, $CDCl_3$): δ 5.93 – 5.78 (2H, m, H-2), 5.19 (2H, ddd, J = 8.1, 3.1, 1.6 Hz, H-1), 5.18 – 5.13 (2H, m, H-1), 3.71 – 3.64 (1H, m, H-4), 3.59 – 3.51 (1H, m, H-4), 2.43 – 2.32 (2H, m, H-3), 2.31 – 2.20 (2H, m, H-3), 2.06 (2H, br m, H-5) ppm.

But-3-enal (33)

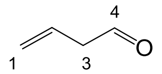

Octa-1,7-diene-4,5-diol **32** (600 mg, 4.22 mmol) was dissolved in DCM (4.5 mL), H_2O (4.5 mL) and cooled to 0 °C. Sodium periodate (1.084 g, 5.07 mmol, 1.2 eq.) was added to the mixture in portions after which it was warmed up to room temperature and stirred for 7 hours. The organic layer was washed with water (2 × 5 mL), brine (2 × 5 mL), dried over $MgSO_4$ and filtered to yield **33** as a colourless solution in DCM (290 mg by NMR, 4.11 mmol, 49 %). The proton NMR spectrum matched that given in literature.[2]

^{1}H NMR (400 MHz, $CDCl_3$): δ 9.66 (1H, t, *J* = 1.7 Hz, H-4), 5.92 (1H, ddt, *J* = 17.2, 10.3 and 6.8 Hz, H-2), 5.27 (1H, dd, *J* = 10.3 and 1.5 Hz, H-1), 5.22 (1H, ddd, *J* = 17.2, 3.0 and 1.5 Hz, H-1), 3.17 (2H, ddd, *J* = 6.8, 3.0 and 1.7 Hz, H-3) ppm.

5-hydroxy-2-methyloct-7-en-3-one (35a)

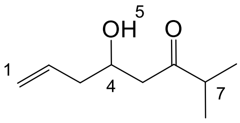

To a solution of silyl enol ether **34a** (1.0 g, 6.3 mmol) in dry DCM (20 mL) was added a 2M solution of butenal **33** (3.2 mL, 6.3 mmol) in dry DCM over a period of 3 minutes at -78 ºC and stirred under N_2 for 15 minutes. A solution of $TiCl_4$ (760 µL, 6.9 mmol) in dry DCM (5 mL) was then added to the reaction mixture over a period of 3 minutes. After leaving the reaction to stir for 2 hours at -78 ºC, it was quenched with cold water (10 mL). Saturated $NaHCO_3$ solution (5 mL) was added and the layers separated. The aqueous layer was extracted with DCM (2 × 10 mL) and the combined organic layers were dried over $MgSO_4$, filtered and concentrated *in vacuo* to give a yellow oil. The crude reaction mixture was purified by flash chromatography (10% ethyl acetate in petroleum ether) on a silica gel column (deadened with 0.5% Et_3N solution) to yield **35a** as a yellow oil (890 mg, 91%).

IR (film): υ_{max} 3428, 3078, 2976, 2934, 1703, 1639, 1467, 1382, 1292, 1035 cm^{-1}; **^{1}H NMR** (400 MHz, $CDCl_3$): δ 5.82 (1H, dddd, *J* = 16.4, 10.6, 7.1 and 7.1 Hz, H-2), 5.16 – 5.09 (2H, m, H-1), 4.10 (1H, ddddd, *J* = 12.2, 7.6, 3.2, 3.1 and 3.1 Hz, H-4), 3.10 (1H, d, *J* = 3.2 Hz, H-5), 2.66 (1H, dd, *J* = 17.7, 3.1 Hz, H-6), 2.60 (1H, hept, *J* = 6.9 Hz, H-7), 2.55 (1H, dd, *J* = 17.7, 7.6 Hz, H-6), 2.26 (2H, m, H-3) 1.10 (3H, d, *J* = 6.9 Hz, H-8), 1.10 (3H, d, *J* = 6.9 Hz, H-8) ppm. **^{13}C-NMR** (101 MHz, $CDCl_3$): δ 171.4, 134.4, 118.0, 67.2, 45.8, 41.6, 41.0, 18.1, 29.2, 29.1, 23.7, 22.7, 14.1 ppm. **MS (ESI)**: m/z 179 ($M+Na^+$); HRMS: found: ($M+Na^+$) 179.1053, $C_9H_{16}O_2Na$ requires ($M+Na^+$) 179.1043

(±)-3-hydroxy-1-phenylhex-5-en-1-one (35b)

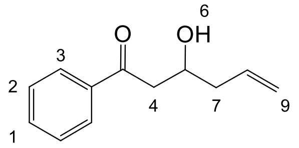

To a solution of silyl enol ether **34b** (273 mg, 1.44 mmol) in dry DCM (10 mL) was added a but-3-enal **33** (100 mg, 1.43 mmol) solution in dry DCM over 3 minutes at -78 ºC under N_2. After stirring for 15 minutes at the same temperature, a solution of $TiCl_4$ in dry DCM was added over 3 minutes to the reaction mixture. After leaving the reaction to stir for 4 hours at -78 ºC, it was quenched with cold water (7 mL). Saturated $NaHCO_3$ solution (5 mL) was added and the layers separated. The aqueous layer was extracted with DCM (2 x 5 mL) and the combined organic layers were dried over $MgSO_4$, filtered and concentrated *in vacuo* to give a yellow oil, which was purified by flash silica gel column chromatography (10% ethyl acetate in petroleum ether) to yield **35b** as a yellow oil (73 mg, 27%). The proton NMR spectrum matched that given in literature.[3]

^{1}H NMR (400 MHz, $CDCl_3$): δ 7.98 - 7.94 (2H, m, H-3), 7.62 – 7.56 (1H, m, H-1), 7.51 – 7.45 (2H, m, H-2), 5.89 (1H, dddd, *J* = 17.2, 10.2, 7.1 and 7.1 Hz, H-8), 5.21 – 5.12 (2H, m, H-9), 4.36 – 4.26 (1H, m, H-5), 3.20 (1H, dd, *J* = 18.2, 3.1 Hz, H-4), 3.07 (1H, dd, *J* = 18.2 and 8.9 Hz, H-4), 2.38 (2H, m, H-7) ppm.

4-hydroxytridec-1-en-6-one (35c)

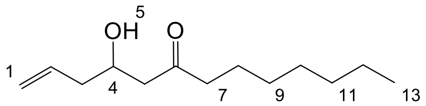

To a solution of silyl enol ether **34c** (850 mg, 4.0 mmol) in dry DCM (20 mL) was added a 2 M solution of butenal **34** (2.0 mL, 4.0 mmol) in dry DCM over a period of 3 minutes at -78 ºC and stirred under N_2 for 15 minutes. A solution of $TiCl_4$ (530 µL) in dry DCM (3 mL) was then added over a period of 3 minutes to the reaction mixture. After leaving the reaction to stir for 2 hours at -78 ºC, it was quenched with cold water (10 mL). Saturated $NaHCO_3$ solution (5 mL) was added and the layers separated. The aqueous layer was extracted with DCM (2 × 10 mL) and the combined organic layers were dried over $MgSO_4$, filtered and concentrated *in vacuo* to give a yellow oil. The crude reaction mixture was purified by flash chromatography (10% ethyl acetate in petroleum ether) on a silica gel column (deadened with 0.5% Et_3N solution) to yield **35c** as a yellow oil (290 mg, 34%).

IR (film): υ_{max} 3422, 3075, 2928, 2857, 1704, 1640, 1461, 1407, 1375, 1045 cm^{-1}; **^{1}H NMR** (400 MHz, $CDCl_3$): δ 5.81 (1H, dddd, 18.3, 9.3, 7.1 and 7.1 Hz, H-2), 5.16 – 5.09 (2H, m, H-1), 4.05-4.16 (1H, m, H-4), 3.07 (1H, br s, H-5), 2.61 (1H, dd, *J* = 17.6 and 3.1 Hz, H-6), 2.51 (1H, dd, *J* = 17.6 and 8.9 Hz, H-6), 2.41 (1H, t, *J* = 7.4 Hz, H-7), 2.17-2.31 (2H, m, H-3) 1.34 - 1.18 (10H, m, H-8 + H-9 + H-10 + H-11 + H-12), 0.87 (3H, t, *J* = 6.8, H-13) ppm. **^{13}C-NMR** (101 MHz, $CDCl_3$): δ 212.3, 134.3, 118.0, 67.1, 48.2, 43.4, 41.0, 31.7, 29.2, 29.1, 23.7, 22.7, 14.1 ppm. **MS (ESI)**: m/z 235 ($M+Na^+$); HRMS: found: ($M+Na^+$) 235.1666, $C_{13}H_{24}O_2Na$ requires ($M+Na^+$) 235.1669

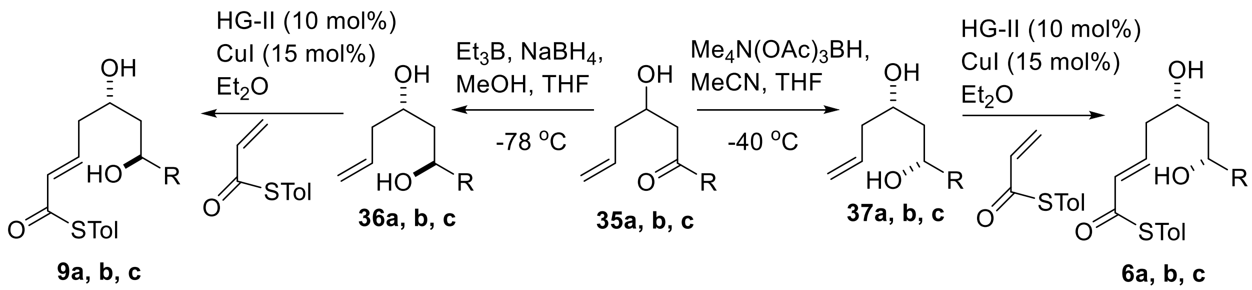

(±)-(3*S*,5*S*)-2-methyloct-7-ene-3,5-diol (36a)

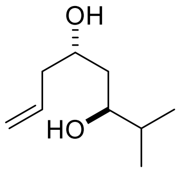

1 M Triethyl borane solution in hexanes (0.46 mL, 0.46 mmol, 1.1 eq.) was added to a mixture of dry THF (4 mL) and methanol (1 mL) at room temperature under a N_2 atmosphere. After stirring the mixture for 1 hour and 30 minutes it was cooled down to -78 ºC, followed by addition of β-hydroxy ketone **35a** (70 mg, 0.44 mmol) solution in dry THF (1.5 mL) over a period of 3 minutes. After stirring for 30 minutes $NaBH_4$ (20 mg, 0.54 mmol, 1.1 eq.) was added in one portion. After stirring the reaction for another 3 hours the reaction mixture was quenched with saturated aqueous NH_4Cl (5 mL) and diluted with ethyl acetate (5 mL). The aqueous layer was extracted with ethyl acetate (2 × 5 mL) and the combined organics were dried over $MgSO_4$, filtered and concentrated *in vacuo* to give a yellow oil, which was azeotroped with methanol (8 × 2 mL). The oil was then purified by flash chromatography (30% ethyl acetate in petroleum ether) on a silica gel column to yield **36a** as a yellow oil (31 mg, 43%)

IR (film): υ_{max} 3357, 3080, 2959, 2878, 1645, 1464, 1435, 1330, 1146, 1072 cm^{-1}; **^{1}H NMR** (400 MHz, $CDCl_3$): δ 5.88 – 5.77 (1H, m, H-2), 5.17 – 5.08 (2H, m, H-1), 3.94 – 3.85 (1H, m, H-4), 3.64 (1H, ddd, *J* = 10.4, 5.8, 2.1 Hz, H-7), 2.99 (2H, br s, H-5 + H-8), 2.33 – 2.18 (2H, m, H-3), 1.67 (1H, ddd, *J* = 14.0, 5.8 and 1.9 Hz, H-6), 1.62 (1H, ddd, *J* = 14.0, 2.1 and 2.1 Hz, H-6), 1.46 (1H, heptd, *J* = 10.4 and 6.8 Hz, H-9), 0.92 (3H, d, *J* = 6.8 Hz, H-10), 0.92 (3H, d, *J* = 6.8 Hz, H-10) ppm. **^{13}C-NMR** (101 MHz, $CDCl_3$): δ 134.5,

118.4, 77.8, 72.2, 42.7, 39.0, 34.2, 18.3, 17.5 ppm. **MS (ESI)**: m/z 181 ($M+Na^+$); HRMS: found: ($M+Na^+$) 181.1192, $C_9H_{18}O_2Na$ requires ($M+Na^+$) 181.1199

(±)-(1*S*,3*S*)-1-phenylhex-5-ene-1,3-diol (36b)

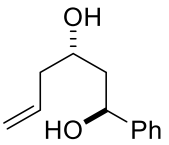

1 M Triethyl borane solution in hexanes (0.75 mL, 0.75 mmol, 1.1 eq.) was added to a mixture of dry THF (6 mL) and methanol (1.5 mL) at room temperature under a N_2 atmosphere. After stirring the mixture for 1 hour and 45 minutes it was cooled down to -78 ºC, followed by addition of β-hydroxy ketone **35b** (130 mg, 0.68 mmol) solution in dry THF (1 mL) over a period of 3 minutes. After stirring for 30 minutes $NaBH_4$ (29 mg, 0.75 mmol, 1.1 eq.) was added in one portion. After stirring the reaction for another 4 hours the reaction mixture was quenched with saturated aqueous NH_4Cl (6 mL) and diluted with ethyl acetate (7 mL). The aqueous layer was extracted with ethyl acetate (2 × 4 mL) and the combined organics were dried over $MgSO_4$, filtered and concentrated *in vacuo* to give a yellow oil, which was azeotroped with methanol (8 × 5 mL). The oil was then purified by flash silica gel column chromatography (20% to 40% ethyl acetate in petroleum ether) to yield **36b** as a yellow oil (76 mg, 58%). The proton NMR spectrum matched that given in literature.[5]

^{1}H NMR (400 MHz, $CDCl_3$): δ 7.40 – 7.32 (4H, m, H-2 + H-3), 7.31 – 7.27 (1H, m, H-1), 5.81 (1H, dddd, *J* = 11.7, 9.2, 7.5 and 6.9 Hz, H-10), 5.18 – 5.13 (1H, m, H-11), 5.13 – 5.10 (1H, m, H-11), 4.96 (1H, dd, *J* = 9.2 and 3.8 Hz, H-4), 4.05 – 3.95 (1H, m, H-7), 2.48 (2H, br, H-5 + H-8), 2.35 – 2.19 (2H, m, H-9), 1.92 – 1.78 (2H, m, H-6) ppm.

(±)-(4*S*,6*R*)-tridec-1-ene-4,6-diol (36c)

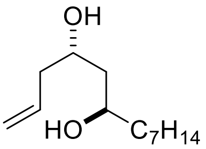

1 M Triethyl borane solution in hexanes (0.23 mL, 0.23 mmol, 1.1 eq.) was added to a mixture of dry THF (2 mL) and methanol (0.5 mL) at room temperature under a N_2 atmosphere. After stirring the mixture for 1 hour and 30 minutes it was cooled down to -78 ºC, followed by addition of β-hydroxy ketone **35c** (45 mg, 0.21 mmol) solution in dry THF (1 mL) over a period of 3 minutes. After stirring for 30 minutes $NaBH_4$ (10 mg, 0.40 mmol, 1.1 eq.) was added in one portion. After stirring the reaction for another 3 hours the reaction mixture was quenched with saturated aqueous NH_4Cl (3 mL) and diluted with ethyl acetate (3 mL). The aqueous layer was extracted with ethyl acetate (2 × 3 mL) and the combined organics were dried over $MgSO_4$, filtered and concentrated *in vacuo* to give a yellow oil, which was azeotroped with methanol (8 × 2 mL). The oil was then purified by flash chromatography (30% ethyl acetate in petroleum ether) on a silica gel column to yield **36c** as a yellow oil (35 mg, 78%)

IR (film): υ_{max} 3344, 3079, 2928, 2857, 1643, 1461, 1325, 1085 cm^{-1}; **^{1}H NMR** (400 MHz, $CDCl_3$): δ 5.80 (1H, dddd, *J* = 14.1, 9.5, 7.2 and 7.2 Hz, H-2), 5.17 – 5.06 (2H, m, H-1), 3.95 – 3.77 (2H, m, H-4 + H-7), 3.15 (2H, br s, H-5 + H-8), 2.30 – 2.15 (2H, m, H-3), 1.62 (1H, ddd, *J* = 14.5, 2.2 Hz, H-6), 1.52 – 1.17 (13H, m, H-6 + H-9 + H-10 + H-11 + H-12 + H-13 + H-14), 0.87 (3H, t, *J* = 6.8 Hz, H-15) ppm. **^{13}C-NMR** (101 MHz, $CDCl_3$): δ 134.4, 118.3, 73.0, 72.1, 42.3, 42.4, 38.2, 31.9, 29.7, 29.4, 25.5, 22.7, 14.2 ppm. **MS (ESI)**: m/z 215 (M+H$^+$) 237 (M+Na$^+$); HRMS: found: (M+H$^+$) 215.2007, (M+Na$^+$) 237.1834, $C_{13}H_{27}O_2$ requires (M+H$^+$) 215.2011, $C_{13}H_{26}O_2Na$ requires (M+Na$^+$) 237.1825

(±)-(5*S*,7*S*,*E*)-*S*-*p*-Tolyl 5,7-dihydroxy-8-methylnon-2-enethioate (9a)

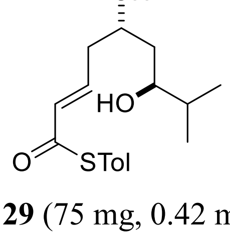

S-*p*-Tolyl prop-2-enethioate **29** (75 mg, 0.42 mmol, 3 eq.) and diol **36a** (30 mg, 0.14 mmol) were dissolved in dry diethyl ether (2 mL) under an N_2 atmosphere. Copper (I) iodide

(4 mg, 0.021 mmol, 15 mol %) and Hoveyda-Grubbs 2nd generation catalyst (8.8 mg, 0.014 mmol, 10 mol %) were added as solids in a single portion, and the mixture was left to stir under reflux for 3 hours. The mixture was then concentrated *in vacuo* and purified by flash chromatography (40% ethyl acetate in petroleum ether) on a silica gel column to yield **9a** as a colourless oil (44 mg, 75%).

IR (film): υ_{max} 3383, 2957, 2921, 2871, 1686, 1633, 1496, 1435, 1303, 1142, 1018 cm^{-1}; **^{1}H NMR** (400 MHz, $CDCl_3$): δ 7.31 (2H, d, *J* = 8.1 Hz, H-2), 7.22 (1H, d, *J* = 8.1 Hz, H-3), 6.99 (1H, ddd, *J* = 15.5, 7.4 and 7.4 Hz, H-6), 6.26 (1H, ddd, *J* = 15.5, 1.4 and 1.4 Hz, H-5), 4.31 – 4.22 (1H, br m, H-9), 4.07 – 3.97 (1H, m, H-8), 3.78 – 3.71 (1H, br m, H-12), 3.67 (1H, ddd, *J* = 10.2, 5.0 and 2.2 Hz, H-11), 2.45 – 2.30 (5H, m, H-1 + H-7), 1.71 – 1.56 (3H, m, H-10 + H-13), 0.92 (3H, d, *J* = 6.8 Hz, H-14), 0.92 (3H, d, *J* = 6.8 Hz, H-14) ppm. **^{13}C-NMR** (101 MHz, $CDCl_3$): δ 188.4, 142.1, 139.7, 134.6, 130.1, 130.0, 123.9, 77.9, 71.5, 40.7, 39.0, 34.3, 21.3, 18.2, 17.2 ppm. **MS (ESI)**: m/z 309 ($M+H^+$), 331 ($M+Na^+$); HRMS: found: ($M+H^+$) 309.1532, ($M+Na^+$) 331.1327, $C_{17}H_{25}O_3S$ requires ($M+H^+$) 309.1519, $C_{17}H_{24}NaO_3S$ requires ($M+Na^+$) 331.1338

(±)-(5*S*,7*S*,*E*)-*S*-*p*-Tolyl 5,7-dihydroxy-7-phenylhept-2-enethioate (9b)

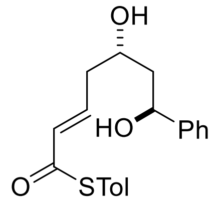

S-*p*-Tolyl prop-2-enethioate **39** (111 mg, 0.63 mmol, 3 eq.) and diol **36b** (40 mg, 0.21 mmol) were dissolved in dry diethyl ether (5 mL) under an N_2 atmosphere. Copper (I) iodide (5 mg, 0.021 mmol, 10 mol %) and Hoveyda-Grubbs 2nd generation catalyst (13.2 mg, 0.021 mmol, 10 mol %) were added as solids in a single portion, and the mixture was left to stir under reflux for 3 hours. The mixture was then concentrated *in vacuo* and purified by flash silica gel column chromatography (30% to 70% ethyl acetate in petroleum ether) to yield **9b** as a colourless oil (67 mg, 94%).

IR (film): ν_{max} 3374, 2928, 2872, 1679, 1630, 1493, 1455, 1307 cm^{-1}. **^{1}H NMR** (400 MHz, $CDCl_3$): δ 7.38 – 7.27 (7H, m, Ar-H), 7.21 (2H, d, *J* = 8.0 Hz, H-3), 6.95 (1H, ddd, *J* = 15.2, 7.4 and 7.4 Hz, H-5), 6.22 (1H, ddd, *J* = 15.2, 1.2 and 1.2 Hz, H-4), 4.93 (1H, dd, *J* = 9.1 and 8.0 Hz, H-10), 4.11 (1H, m, H-7), 3.64 (1H, br, H-11), 3.10 (1H, br, H-8), 2.48 – 2.33 (5H, m, H-1 + H-6), 1.86 (1H, ddd, *J* = 14.5, 10.0 and 9.1 Hz, H-9), 1.75 (1H, ddd, *J* = 14.5, 8.0 and 2.6 Hz, H-9) ppm. **^{13}C-NMR** (101 MHz, $CDCl_3$): δ 188.4, 144.0, 141.7, 139.7, 134.5, 130.2, 130.0, 128.6, 127.9, 125.6, 123.8, 75.3, 71.0, 44.8, 40.6, 21.3 ppm. **MS** (ESI): m/z 343 ($M+H^+$), 365 ($M+Na^+$); HRMS: found: ($M+H^+$) 343.1358, ($M+Na^+$) 365.1180; $C_{20}H_{23}O_3S$ requires ($M+H^+$) 343.1368, $C_{20}H_{22}NaO_3S$ requires ($M+Na^+$) 365.1187

(±)-(5*S*,7*R*,*E*)-*S*-*p*-Tolyl 5,7-dihydroxytetradec-2-enethioate (9c)

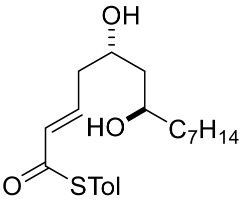

S-*p*-Tolyl prop-2-enethioate **29** (50 mg, 0.28 mmol, 3 eq.) and diol **37c** (20 mg, 0.09 mmol) were dissolved in dry diethyl ether (0.5 mL) under an N_2 atmosphere. Copper (I) iodide (2.7 mg, 0.014 mmol, 15 mol%) and Hoveyda-Grubbs 2nd generation catalyst (5.8 mg, 0.009 mmol, 10 mol %) were added as solids in a single portion, and the mixture was left to stir under reflux for 3 hours. The mixture was then concentrated *in vacuo* and purified by flash chromatography (40% ethyl acetate in petroleum ether) on a silica gel column to yield **9c** as a colourless oil (28 mg, 86%).

IR (film): υ_{max} 3372, 2924, 2930, 2853, 1679, 1632, 1500, 1457, 1303, 1138, 1024 cm^{-1}; **^{1}H NMR** (400 MHz, $CDCl_3$): δ 7.31 (2H, d, *J* = 8.1 Hz, H-2), 7.22 (2H, d, *J* = 8.1 Hz, H-3), 6.97 (1H, ddd, *J* = 15.5, 7.5 and 7.5 Hz, H-6), 6.25 (1H, d, *J* = 15.5 Hz, H-5), 4.08 – 3.99 (1H, m, H-8), 3.91 – 3.82 (1H, m, H-11), 2.46 – 2.27 (5H, m, H-1 + H-7), 1.65 – 1.18 (14H, m, H-10 + H-13 + H-14 + H-15 + H-16 + H-17 + H-18), 0.88 (3H, t, *J* = 6.4 Hz, H-19) ppm. **^{13}C-NMR** (101 MHz, $CDCl_3$): δ 188.5, 142.0, 139.7, 134.6, 130.1, 130.0, 123.9, 73.2,

71.3, 42.4, 40.1, 38.3, 31.8, 29.5, 29.2, 25.2, 22.6, 21.3, 14.1 ppm. **MS (ESI)**: m/z 365 $(M+H^+)$ 387 $(M+Na^+)$; HRMS: found: $(M+H^+)$ 365.2141, $(M+Na^+)$ 387.1962, $C_{21}H_{33}O_3S$ requires $(M+H^+)$ 365.2145, $C_{21}H_{32}NaO_3S$ requires $(M+Na^+)$ 387.1964

(±)-(3R,5S)-2-methyloct-7-ene-3,5-diol (37a)

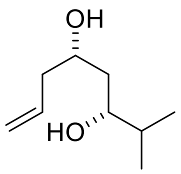

To a solution of $NMe_4BH(OAc)_3$ (412 mg, 1.6 mmol, 7 eq.) in dry MeCN (1 mL) and AcOH (1.2 mL) at -35 ºC under an N_2 atmosphere was added a solution of β-hydroxy ketone **35a** (35 mg, 0.22 mmol) in dry MeCN (1 mL) over a period of 3 minutes. The reaction mixture was stirred for 4 hours at -35 ºC. The reaction was quenched with 10% Rochelle's salt (5 mL) and warmed to room temperature. The mixture was partitioned between ethyl acetate (10 mL) and a saturated aqueous solution of $NaHCO_3$ (10 mL) and the aqueous layer was extracted with ethyl acetate (2 x 10 mL). The combined organics were dried over $MgSO_4$, filtered and concentrated *in vacuo* to give a yellow oil. The oil was then purified by flash chromatography (30% ethyl acetate in petroleum ether) on a silica gel column to yield **37a** as a yellow oil (29 mg, 83%).

IR (film): υ_{max} 3390, 3081, 2959, 2928, 2875, 1642, 1405, 1333, 1288, 1143, 1051 cm^{-1}; **^{1}H NMR** (400 MHz, $CDCl_3$): δ 5.89 – 5.73 (1H, m, H-2), 5.19 – 5.01 (2H, m, H-1), 3.99 (1H, dddd, *J* = 7.7, 7.4, 5.5 and 3.3 Hz, H-4), 3.68 (1H, ddd, *J* = 8.9, 6.1 and 2.8 Hz, H-7), 2.36 – 2.16 (2H, m, H-3), 1.76 – 1.62 (2H, m, H-6 + H-9), 1.58 (1H, ddd, *J* =14.5, 7.7, 2.8 Hz, H-6), 0.95 (3H, d, *J* = 6.7 Hz, H-10), 0.90 (3H, d, *J* = 6.8 Hz, H-10) ppm. **^{13}C-NMR** (101 MHz, $CDCl_3$): δ 134.7, 118.3, 73.9, 68.3, 42.0, 38.9, 33.4, 18.6, 17.9 ppm. **MS (ESI)**: m/z 181 $(M+Na^+)$; HRMS: found: $(M+Na^+)$ 181.1202, $C_9H_{18}O_2Na$ requires $(M+Na^+)$ 181.1199

(±)-(1*S*,3*R*)-1-phenylhex-5-ene-1,3-diol (37b)

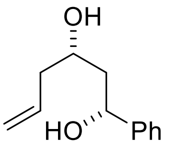

To a solution of $NMe_4BH(OAc)_3$ (1.09 g, 4.12 mmol, 7 eq.) in dry MeCN (4.4 mL) and AcOH (4.5 mL) at -35 ºC under an N_2 atmosphere was added a solution of β-hydroxy ketone **35b** (112 mg, 0.59 mmol) in dry MeCN (4.4 mL) over a period of 3 minutes. The reaction mixture was stirred for 6 hours at -35 ºC and 18 hours at -20 ºC. The reaction was quenched with 10% Rochelles salt (7 mL) and warmed to room temperature. The mixture was partitioned between ethyl acetate (40 mL) and a saturated aqueous solution of $NaHCO_3$ (40 mL) and the aqueous layer was extracted with ethyl acetate (2 × 40 mL). The combined organics were dried over $MgSO_4$, filtered and concentrated *in vacuo* to give a yellow oil, which was purified by flash silica gel column chromatography (20% to 50% ethyl acetate in petroleum ether) to yield **37b** as a yellow oil (68 mg, 61%). The proton NMR spectrum matched that given in literature.[5]

^{1}H NMR (400 MHz, $CDCl_3$): δ 7.40 – 7.32 (4H, m, H-2 + H-3), 7.30 – 7.27 (1H, m, H-1), 5.85 – 5.71 (1H, m, H-10), 5.19 – 5.10 (2H, m, H-11), 5.08 (1H, dd, *J* = 7.8, 3.6 Hz, H-4), 3.92 (1H, dddd, *J* = 8.3, 8.3, 5.1, and 3.2 Hz, H-7), 2.88 (2H, br, H-5 + H-8), 2.38 – 2.20 (2H, m, H-8), 1.99 – 1.84 (2H, m, H-6) ppm.

(±)-(4*S*,6*S*)-tridec-1-ene-4,6-diol (37c)

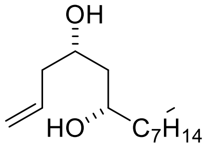

To a solution of $NMe_4BH(OAc)_3$ (1.04 g, 3.95 mmol, 7 eq.) in dry MeCN (5 mL) and AcOH (6 mL) at -35 ºC under an N_2 atmosphere was added a solution of β-hydroxy ketone **35c** (120 mg, 0.56 mmol) in dry MeCN (5 mL) over a period of 3 minutes. The reaction mixture was stirred for 3 hours at -35 ºC. The reaction was quenched with 10% Rochelle's salt (7 mL) and warmed to room temperature. The mixture was partitioned

between ethyl acetate (20 mL) and a saturated aqueous solution of $NaHCO_3$ (20 mL) and the aqueous layer was extracted with ethyl acetate (2 × 20 mL). The combined organics were dried over $MgSO_4$, filtered and concentrated *in vacuo* to give a yellow oil. The oil was then purified by flash chromatography (30% ethyl acetate in petroleum ether) on a silica gel column to yield **37c** as a yellow oil (103 mg, 86%).

IR (film): υ_{max} 3378, 3078, 2925, 2855, 1642, 1404, 1334, 1143, 1073 cm^{-1}; **^{1}H NMR** (400 MHz, $CDCl_3$): δ 5.82 (1H, dddd, *J* = 16.6, 9.5, 7.2 and 7.2 Hz, H-2), 5.18 – 4.96 (2H, m, H-1), 4.39 – 3.60 (3H, m, H-5 + H-8 + H-4), 2.61 – 2.17 (4H, m, H-7 + H-3), 1.64 – 1.20 (14H, m, H-6 + H-9 + H-10 + H-11 + H-12 + H-13 + H-14), 0.87 (3H, t, *J* = 6.8 Hz, H-15) ppm. **^{13}C-NMR** (101 MHz, $CDCl_3$): δ 134.8, 118.3, 69.4, 68.3, 42.1, 41.9, 37.6, 31.9, 29.7, 29.4, 25.9, 22.7, 14.2 ppm. **MS (ESI)**: m/z 237 ($M+Na^+$); HRMS: found: ($M+H^+$) 215.2006, ($M+Na^+$) 237.1826, $C_{13}H_{27}O_2$ requires ($M+H^+$) 215.2011, $C_{13}H_{26}O_2Na$ requires ($M+Na^+$) 237.1825

(±)-(5*S*,7*R*,*E*)-*S*-*p*-Tolyl 5,7-dihydroxy-8-methylnon-2-enethioate (6a)

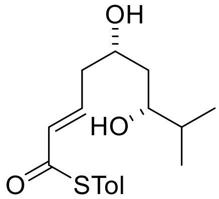

S-*p*-Tolyl prop-2-enethioate **29** (135 mg, 0.75 mmol, 3 eq.) and diol **37a** (40 mg, 0.25 mmol) were dissolved in dry diethyl ether (2 mL) under an N_2 atmosphere. Copper (I) iodide (7.2 mg, 0.038 mmol, 15 mol %) and Hoveyda-Grubbs 2nd generation catalyst (16.0 mg, 0.025 mmol, 10 mol %) were added as solids in a single portion, and the mixture was left to stir at reflux for 3 hours. The mixture was then concentrated *in vacuo* and purified by flash chromatography (40% ethyl acetate in petroleum ether) on a silica gel column to yield **6a** as a colourless oil (41 mg, 54%).

IR (film): υ_{max} 3401, 2960, 2921, 2871, 1683, 1629, 1493, 1464, 1400, 1142, 1013 cm^{-1}; **^{1}H NMR** (400 MHz, $CDCl_3$): δ 7.31 (2H, d, *J* = 8.1 Hz, H-2), 7.22 (1H, d, *J* = 8.1 Hz,

H-3), 6.98 (1H, ddd, *J* = 15.5, 7.3 and 7.3 Hz, H-6), 6.27 (1H, ddd, *J* = 15.5, 1.4 and 1.4 Hz, H-5), 4.16 – 4.08 (1H, m, H-8), 3.85 – 3.72 (2H, br m, H-9 + H-12), 3.68 (1H, ddd, *J* = 9.0, 6.1 and 3.2 Hz, H-11), 2.49 – 2.34 (5H, m, H-1 + H-7), 1.99 (1H, heptd, *J* = 6.8 and 6.1 Hz, H-13), 1.71 – 1.60 (2H, m, H-10), 0.95 (3H, d, *J* = 6.8 Hz, H-14), 0.91 (3H, d, *J* = 6.8 Hz, H-14) ppm. **^{13}C-NMR** (101 MHz, $CDCl_3$): δ 188.4, 142.3, 139.7, 134.6, 130.1, 130.0, 123.9, 73.9, 68.1, 40.3, 39.2, 33.4, 21.3, 18.6, 17.9 ppm. **MS (ESI)**: m/z 331 ($M+Na^+$); HRMS: found: ($M+Na^+$) 331.1344, $C_{17}H_{24}NaO_3S$ requires ($M+Na^+$) 331.1338

(±)- (5*R*,7*S*,*E*)-*S*-*p*-Tolyl 5,7-dihydroxy-7-phenylhept-2-enethioate (6b)

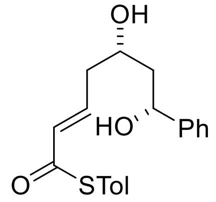

S-*p*-Tolyl prop-2-enethioate **29** (111 mg, 0.63 mmol, 3 eq.) and diol **37b** (40 mg, 0.21 mmol) were dissolved in dry diethyl ether (5 mL) under an N_2 atmosphere. Copper (I) iodide (5 mg, 0.021 mmol, 10 mol %) and Hoveyda-Grubbs 2nd generation catalyst (13.2 mg, 0.021 mmol, 10 mol %) were added as solids in a single portion, and the mixture was left to stir under reflux for 3 hours. The mixture was then concentrated *in vacuo* and purified by flash silica gel column chromatography (30% to 70% ethyl acetate in petroleum ether) to yield **6b** as a colourless oil (44 mg, 62%).

IR (film): ν_{max} 3390, 2923, 2867, 1676, 1630, 1494, 1454, 1304 cm^{-1}. **^{1}H NMR** (400 MHz, $CDCl_3$): δ 7.38 – 7.27 (7H, m, Ar-H), 7.21 (2H, d, *J* = 7.9 Hz, H-3), 6.94 (1H, ddd, *J* = 15.3, 7.1 and 7.1 Hz, H-5), 6.23 (1H, d, *J* = 15.3 Hz, H-4), 5.05 (1H, dd, *J* = 6.0 and 6.0 Hz, H-10), 4.09 – 4.00 (1H, m, H-7), 2.94, (2H, br, H-8 + H-11), 2.43 – 2.38 (2H, m, H-6), 2.37 (3H, s, H-1), 1.95 – 1.83 (2H, m, H-9) ppm. **^{13}C-NMR** (101 MHz, $CDCl_3$): δ 188.5, 144.0, 142.0, 139.7, 134.5, 130.1, 130.0, 128.6, 127.6, 125.5, 123.8, 71.6, 67.6, 44.3, 40.2, 21.3 ppm. **MS** (ESI): m/z 365 ($M+Na^+$); HRMS: found: ($M+Na^+$) 365.1182; $C_{20}H_{22}NaO_3S$ requires ($M+Na^+$) 365.1187

(±)-(5*S*,7*S*,*E*)-*S*-*p*-Tolyl 5,7-dihydroxytetradec-2-enethioate (6c)

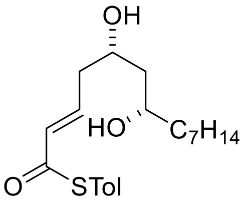

S-*p*-Tolyl prop-2-enethioate **29** (100 mg, 0.56 mmol, 3 eq.) and diol **37c** (40 mg, 0.19 mmol) were dissolved in dry diethyl ether (2 mL) under an N_2 atmosphere. Copper (I) iodide (5.3 mg, 0.028 mmol, 15 mol %) and Hoveyda-Grubbs 2nd generation catalyst (11.7 mg, 0.019 mmol, 10 mol %) were added as solids in a single portion, and the mixture was left to stir under reflux for 3 hours. The mixture was then concentrated *in vacuo* and purified by flash chromatography (40% ethyl acetate in petroleum ether) on a silica gel column to yield **6c** as a colourless oil (36 mg, 53%).

IR (film): υ_{max} 3387, 2925, 2856, 1683, 1629, 1500, 1464, 1138, 1013 cm^{-1}; **^{1}H NMR** (400 MHz, $CDCl_3$): δ 7.31 (2H, d, *J* = 8.1 Hz, H-2), 7.23 (2H, d, *J* = 8.1 Hz, H-3), 6.98 (1H, ddd, *J* = 15.5, 7.3 and 7.3 Hz, H-6), 6.27 (1H, d, *J* = 15.5 Hz, H-5), 4.18 – 4.10 (1H, m, H-8), 3.99 – 3.92 (1H, m, H-11), 2.50 – 2.29 (5H, m, H-1 + H-7), 1.72 – 1.18 (14H, m, H-10 + H-13 + H-14 + H-15 + H-16 + H-17 + H-18), 0.88 (3H, t, *J* = 6.8 Hz, H-19) ppm. **^{13}C-NMR** (101 MHz, $CDCl_3$): δ 188.4, 142.2, 139.7, 134.6, 130.1, 130.0, 123.9, 69.5, 67.9, 42.1, 40.3, 37.4, 31.8, 29.5, 29.2, 25.7, 22.6, 21.3, 14.1 ppm. **MS (ESI)**: m/z 387 ($M+Na^+$); HRMS: found: ($M+Na^+$) 387.1961, $C_{21}H_{32}NaO_3S$ requires ($M+Na^+$) 387.1964

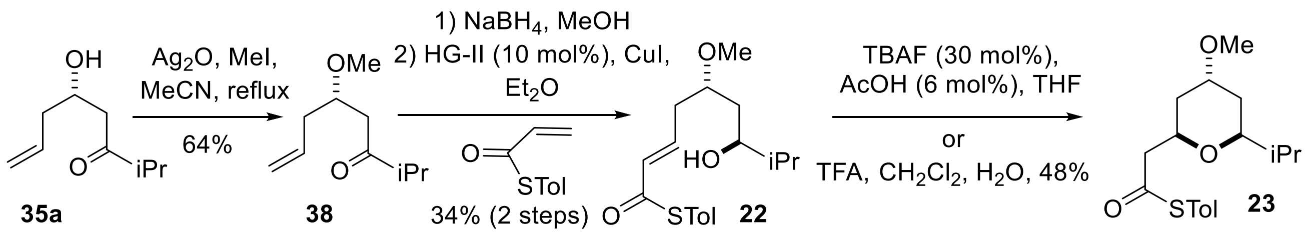

5-methoxy-2-methyloct-7-en-3-one (38)

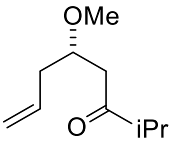

Methyl iodide (5 mL) and silver (I) oxide (460 mg, 2.0 mmol) were added to a solution of β-hydroxyketone **35a** (156 mg, 1.0 mmol) in MeCN (5 ml). The mixture was heated under reflux for 18 hours. Another portion of silver (I) oxide (460 mg, 2.0 mmol) was added and heating continued for another 5 hours. The solids were filtered off and the reaction quenched with saturated aqueous solution of $NaHCO_3$ (15 ml) and extracted with diethyl ether (3 × 10 ml). The organic fraction was dried with $MgSO_4$, filtered and concentrated *in vacuo* and purified by flash chromatography (10% diethyl ether in pentane) on a silica gel column to yield **38** as a colourless oil (100 mg, 64%).

IR (film): υ_{max} 2966, 2931, 1709, 1461, 1365, 1260, 1098 cm^{-1}; **^{1}H NMR** (400 MHz, $CDCl_3$): δ 5.76 (1H, dddd, *J* = 19.1, 9.5 and 7.1 Hz, H-2), 5.09 – 5.02 (2H, m, H-1), 3.77 (1H, m, H-4), 3.30 (3H, s, H-5), 2.68 (1H, dd, *J* = 16.6 and 7.4 Hz, H-6), 2.57 (1H, hept, *J* = 6.9 Hz, H-7), 2.56 (1H, dd, *J* = 16.6 and 5.2 Hz, H-6), 2.30 - 2.24 (2H, m, H-3), 1.06 (3H, d, *J* = 6.9 Hz, H-8), 1.06 (3H, d, *J* = 6.9 Hz, H-8) ppm. **^{13}C-NMR** (101 MHz, $CDCl_3$): δ 213.3, 134.0, 117.7, 76.4, 57.1, 44.5, 41.6, 37.9, 17.9, 17.8 ppm. **MS (ESI)**: m/z 193 ($M+Na^+$); HRMS: found: ($M+Na^+$) 193.1202, $C_{10}H_{18}NaO_2$ requires ($M+Na^+$) 193.1199

(±)-(5*S*,7*R*,*E*)-*S*-*p*-Tolyl 7-hydroxy-5-methoxy-8-methylnon-2-enethioate (22)

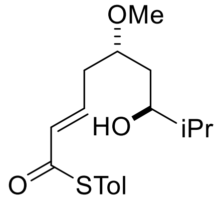

To a solution of β-methoxyketone **38** (80 mg, 0.47 mmol) in methanol (10 ml) $NaBH_4$ (18 mg, 0.47 mmol) was added in one portion. The mixture was stirred for 1 hour at room temperature and then quenched with saturated aqueous solution of NH_4Cl. The solvents were removed *in vacuo* and the resultant brown oil was dissolved in ethyl acetate (15 mL) and H_2O (15 mL) added. The layers were separated and the organic layer was washed with brine (15 mL), dried ($MgSO_4$) and concentrated *in vacuo* to give the methoxy alcohol as a mixture of diastereomers.

S-*p*-Tolyl prop-2-enethioate **29** (250 mg, 1.4 mmol, 3 eq.) and mixture of diasatereomers of methoxy alcohol (80 mg, 0.46 mmol) were dissolved in dry diethyl ether (8 ml) under an N_2 atmosphere. Copper (I) iodide (13 mg, 0.070 mmol, 15 mol %) and Hoveyda-Grubbs 2nd generation catalyst (30 mg, 0.046 mmol, 10 mol %) were added as solids in a single portion, and the mixture was left to stir at reflux for 3 hours. The mixture was then concentrated *in vacuo* and purified by repeated preparative thin layer chromatography (0.5% methanol in DCM) on a silica gel plate to yield the 1,3-*syn* methoxy alcohol ***syn*-22** (50 mg, 34%) and 1,3-*anti* methoxy alcohol ***anti*-22** (45 mg, 30%) as colorless oils.

IR (film): υ_{max} 3487, 2960, 2928, 2871, 1679, 1632, 1489, 1464, 1364, 1181, 1092, 1018 cm^{-1}; ^{1}**H NMR** (400 MHz, $CDCl_3$): δ 7.32 (2H, d, *J* = 8.1 Hz, H-2), 7.23 (1H, d, *J* = 8.1 Hz, H-3), 6.93 (1H, ddd, *J* = 15.3, 7.4 and 7.4 Hz, H-6), 6.27 (1H, d, *J* = 15.3 Hz, H-5), 3.65 –3.52 (2H, m, H-8 + H-11), 3.41 (3H, s, H-9), 3.35 – 3.29 (1H, br s, H-12), 2.54 – 2.45 (2H, m, H-7), 2.38 (3H, s, H-1), 1.65 (1H, ddd, *J* = 13.4, 11.9 and 6.7 Hz, H-10), 1.62 – 1.54 (1H, m, H-12), 1.39 (1H, m, H-10), 0.91 (3H, d, *J* = 6.8 Hz, H-14), 0.91 (3H, d, *J* = 6.8 Hz, H-14) ppm. ^{13}**C-NMR** (101 MHz, $CDCl_3$): δ 188.2, 141.2, 139.7, 134.6, 130.3, 130.0, 80.9, 76.1,

56.7, 37.6, 36.2, 33.8, 21.3, 18.4, 17.4 ppm. **MS (ESI)**: m/z 323 ($M+H^+$), 345 ($M+Na^+$); HRMS: found: ($M+H^+$) 323.1679, ($M+Na^+$) 345.1491, $C_{18}H_{27}O_3S$ requires ($M+H^+$) 323.1675, $C_{18}H_{26}NaO_3S$ requires ($M+Na^+$) 345.1495

References

1. Gilbert, A. B; Peters, F. B; Johnson, H. W. *J. Org. Chem.*, **1983**, *48*, 2724.
2. Crimmins, M.T.; Kirincich, S. J.; Wells, A. J.; Choy, A. L. *Synth Comm.* **1998**, *28*, 3675.
3. Hiebel, M.A.; Pelotier, B.; Piva, O. *Tetrahedron*, **2007**, *63*, 7874.
4. Karlubíková, O.; Babjak, M.; Gracza, T. *Tetrahedron*, **2011**, *67*, 4980.
5. Kumaraswamy, G.; Rambabu, D. *Tetrahedron Asym*., **2013**, *24*, 196.

Details of the Computational Studies

All of the molecular mechanics conformation analyses were done using MacroModel (Verion 9.9) and MMFF forcefield. The geometries were fully optimized at the B3LYP/6-31G(d) level of theory in gas phase and then reoptimized in solvent using PCM implicit solvent model. All of the optimized geometries were verified by frequency analysis as minima (zero imaginary frequencies) of transition structures (a single imaginary frequency). All of the quantum chemical computations were done using Jaguar (version 7.9). All of the structural representations were generated with jmol-14.2.4.

Calculations on TBAF mediated cyclizations

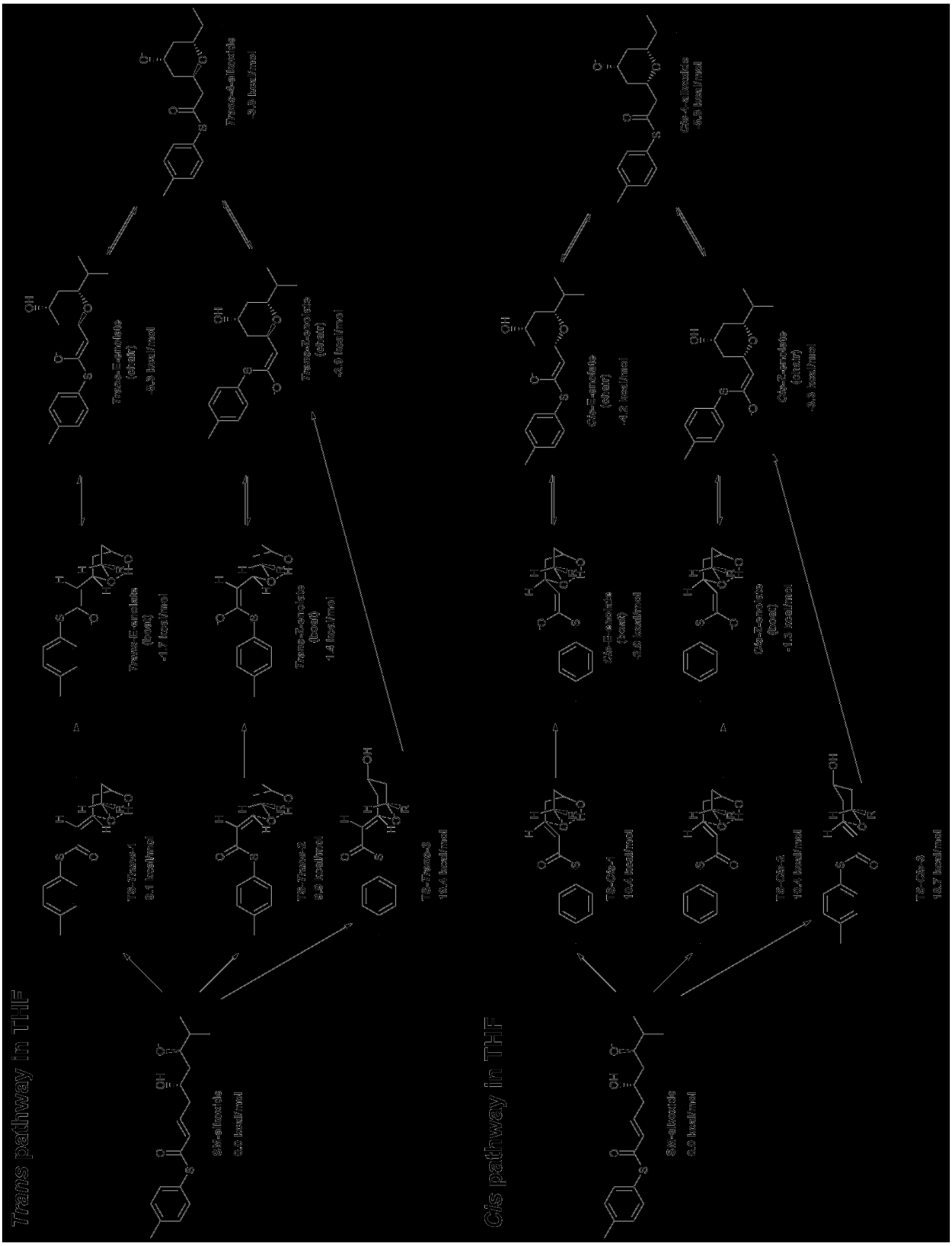

Figure S1 *Overview of TBAF mediated cyclization pathways explored in this study*

Lowest energy conformation for the ^{i}Pr alkoxide (12)

```
Cartesian Coordinates (Angstroms)
Atom X Y Z
--------- ------------- ------------- -------------
C1 -0.2564582662 -6.2087693724 -1.0098489127
C2 0.5325215149 -6.1118040910 -2.1653825698
C3 1.3801312738 -7.1658343981 -2.5199963299
C4 1.4527681450 -8.3363427889 -1.7456202718
C5 0.6526209412 -8.4191655001 -0.5943141683
C6 -0.1917122942 -7.3691504461 -0.2242763342
S7 -1.4705362844 -4.9568566543 -0.5900388314
H8 0.4755778866 -5.2261995855 -2.7938539178
H9 1.9840340517 -7.0807951740 -3.4231209402
H11 0.6821677416 -9.3182445264 0.0199427736
H12 -0.8104396981 -7.4577047175 0.6659575103
C12 2.3773795016 -9.4704039783 -2.1300879615
H13 1.9960715632 -10.4346628262 -1.7748563249
H14 2.5076399165 -9.5326269090 -3.2166372892
H15 3.3746666457 -9.3311149573 -1.6897141573
C16 -0.5388550906 -3.6166328511 0.2326363102
O17 0.6817437619 -3.5867611558 0.2774512098
C18 -1.4685211958 -2.6354646492 0.8307389890
C21 -1.0338021062 -1.5918150727 1.5678868499
C24 -1.8980695271 -0.6515575612 2.3548215882
H25 -2.9620708958 -0.8341713853 2.1587333207
H26 -1.7228407988 -0.8848226025 3.4170820773
H23 -2.5346106995 -2.8286920157 0.7087343674
H24 0.0446063382 -1.4456632667 1.6607543717
C25 -1.5463100035 0.8548222857 2.1867039302
H28 -0.4514129144 0.9134876528 2.0791061681
C28 -2.2011354116 1.5044384112 0.9460344241
H29 -1.6206982635 2.3974572857 0.6778284063
H30 -2.1311813116 0.8024646665 0.1001586043
C31 -3.6764367413 1.9424674022 1.1827062532
H33 -4.2280687283 1.0183664229 1.4826382018
O34 -3.7490265621 2.9049082977 2.1996824683
C37 -3.7306822291 3.5918607754 -0.8245793706
C38 -4.3536488594 2.3532895975 -0.1640716358
C39 -5.8676094710 2.5448863691 0.0101552697
H43 -4.2509067352 3.8319386803 -1.7622527394
H44 -6.3544005181 2.7499605078 -0.9537826210
O45 -1.9046186760 1.5742213208 3.3735303208
H46 -2.6737895536 2.2045533766 3.0802329217
H40 -2.6718871709 3.4415783232 -1.0680304899
H41 -3.8048413141 4.4619518145 -0.1621756261
H42 -6.0793009544 3.3830985541 0.6850630217
H45 -6.3340923301 1.6450122377 0.4346024368
H47 -4.2101133844 1.4994764440 -0.8488367251
Gas Phase Energy = -1285.58196406 hartrees
Solution Phase Energy (THF) = -1285.66829426 hartrees
```

Lowest energy 2,6-*trans*-*E*-boat-like transition state (TS-*trans*-1)

Cartesian Coordinates (Angstroms)

Atom	X	Y	Z
C1	-1.0418289504	-10.1328017332	0.7593627192
C2	-1.5249489698	-9.4063283159	1.8593203723
C3	-1.4857362667	-9.9588375380	3.1429977854
C4	-0.9627729854	-11.2429349294	3.3670463291
C5	-0.4891013683	-11.9647816242	2.2588157986
C6	-0.5249618279	-11.4206702472	0.9713549379
S7	-0.9048881181	-9.3518684743	-0.8554934489
H8	-1.9114347621	-8.4006789578	1.7140701824
H9	-1.8524405901	-9.3730386740	3.9855072755
H11	-0.0703220564	-12.9601667628	2.4043483565
H12	-0.1316182010	-11.9887343077	0.1315511296
C12	-0.9181596560	-11.8337683296	4.7594798848
H13	-1.8427040638	-12.3835696873	4.9871148899
H14	-0.0850552896	-12.5381495921	4.8685632172
H15	-0.8070000399	-11.0537365018	5.5223905091
C16	-2.4290392664	-9.6491106421	-1.8873568813
O17	-2.3576658673	-9.0992002002	-2.9923705856
C18	-3.4673727696	-10.4684651026	-1.3552961067
C21	-4.6333639539	-10.7236156416	-2.0516468310
C24	-5.5649950064	-11.8219634121	-1.5903168017
H25	-5.4738318208	-12.6506879637	-2.3092194327
H26	-5.2291418134	-12.2087624102	-0.6176616098
H23	-3.3373041676	-10.8589886454	-0.3512009529
H24	-4.6901205968	-10.4298054952	-3.0930816386
C25	-7.0623286085	-11.4574883196	-1.4761556320
H28	-7.6190295741	-12.3844148804	-1.2908592800
C28	-7.3273188923	-10.4547410652	-0.3399553773
H29	-8.3812491560	-10.1452647009	-0.4111827516
H30	-7.2027772903	-10.9583348342	0.6302970315
C31	-6.3821635960	-9.2225186191	-0.4595996418
H33	-5.4617287683	-9.4532210056	0.1224913371
O34	-6.0382189241	-9.0240691688	-1.7999964608
C37	-5.9964501149	-6.7785085013	0.1029222307
C38	-6.9719727571	-7.9578317764	0.2158557272
C39	-7.3295786437	-8.2068737397	1.6923040143
H43	-6.4338719788	-5.8581792729	0.5134642703
H44	-6.4432391235	-8.5356692297	2.2538328264
O45	-7.5532799992	-10.9271812375	-2.7208955840
H46	-7.1090247862	-10.0267768925	-2.7045360779
H40	-5.7173568746	-6.6035928552	-0.9398473625
H41	-5.0732177794	-6.9888085537	0.6620838182
H42	-8.1040768191	-8.9733161394	1.8135273151
H45	-7.6974453465	-7.2879338112	2.1681048735
H47	-7.8960865060	-7.6984074379	-0.3258746178

Gas Phase Energy = -1285.57795431 hartrees

Solution Phase Energy (THF) = -1285.6537395 hartrees

Lowest energy 2,6-*trans*-*Z*-boat-like transition state (TS-*trans*-2)

```
Cartesian Coordinates (Angstroms)
Atom X Y Z
--------- ------------- ------------- -------------
C1 -3.0196281760 -6.3281719169 -0.0972870468
C2 -1.9996248888 -5.6549012974 -0.7842127064
C3 -1.3629095446 -4.5542201587 -0.2026880465
C4 -1.7152536513 -4.1096044576 1.0800791175
C5 -2.7285354444 -4.8028088416 1.7648195643
C6 -3.3830065465 -5.8922027327 1.1858789180
S7 -3.9390434225 -7.5987739109 -0.9570077488
H8 -1.7133333470 -5.9842705251 -1.7803664965
H9 -0.5843321233 -4.0342372152 -0.7598429515
H11 -3.0258716960 -4.4733232603 2.7597168710
H12 -4.1856628449 -6.3927712099 1.7219242916
C12 -1.0165396178 -2.9264789936 1.7125716698
H13 -1.7105639568 -2.3234416705 2.3102810862
H14 -0.2096070539 -3.2542372138 2.3828016804
H15 -0.5676142771 -2.2764257265 0.9530706089
C16 -3.5701072772 -9.1471339127 -0.0055652607
O17 -2.9475108933 -9.0864625731 1.0629940826
C18 -4.0606724377 -10.3495976276 -0.6086155738
C21 -4.6766897520 -10.4717428345 -1.8415738547
C24 -4.8801702728 -11.8528523191 -2.4306361656
H25 -4.1778833669 -11.9650167542 -3.2705680682
H26 -4.6098344718 -12.6150240972 -1.6864243248
H23 -3.9281110333 -11.2342142203 0.0143454452
H24 -4.6274207259 -9.6530657208 -2.5496077203
C25 -6.2955110366 -12.1744748235 -2.9569098585
H28 -6.2521882704 -13.1514775297 -3.4541215599
C28 -7.3423394408 -12.2138671347 -1.8296562957
H29 -8.3251716514 -12.3391583457 -2.3033074439
H30 -7.1719627997 -13.1030713425 -1.2046352452
C31 -7.2745889711 -10.9135804664 -0.9713893625
H33 -6.5764395567 -11.1196496542 -0.1316310232
O34 -6.7556646895 -9.8639217721 -1.7360504956
C37 -9.0427115100 -11.6264903580 0.7369526171
C38 -8.6120118838 -10.5470828471 -0.2715451214
C39 -9.7517530554 -10.2096874974 -1.2427546849
H43 -9.9104638525 -11.2979503540 1.3245854648
H44 -10.0978024742 -11.0970716650 -1.7895918236
O45 -6.6914742328 -11.2241036176 -3.9646281726
H46 -6.8537770673 -10.4191326851 -3.3909691370
H40 -8.2318004803 -11.8576346355 1.4411662539
H41 -9.3235018308 -12.5618883446 0.2335890218
H42 -9.4252121024 -9.4666508473 -1.9790512004
H45 -10.6169418101 -9.8022340919 -0.7024712590
H47 -8.3831409387 -9.6327616161 0.2970798487
Gas Phase Energy = -1285.5781272 hartrees
Solution Phase Energy (THF) = -1285.65258357 hartrees
```

Lowest energy 2,6-*trans*-*Z*-chair-like transition state (TS-*trans*-3)

Cartesian Coordinates (Angstroms)

Atom	X	Y	Z
C1	0.4738706630	-9.5331060980	-1.9581420105
C2	0.9468407797	-9.5569296882	-0.6360392771
C3	2.3181732659	-9.6304285307	-0.3842599962
C4	3.2553584405	-9.6586478830	-1.4318655098
C5	2.7707931457	-9.6277085258	-2.7473570262
C6	1.3968021174	-9.5755559904	-3.0116386785
S7	-1.2615566761	-9.2838978991	-2.3290033355
H8	0.2463806487	-9.5076252372	0.1943700400
H9	2.6674009972	-9.6476886074	0.6477315478
H11	3.4722926954	-9.6423088011	-3.5819703340
H12	1.0436011682	-9.5556493350	-4.0405696477
C12	4.7394967689	-9.7210295890	-1.1394687438
H13	5.3315339256	-9.5239634699	-2.0391013622
H14	5.0272975518	-8.9842343823	-0.3757684477
H15	5.0315728724	-10.7109548560	-0.7611202886
C16	-2.0753156706	-10.7764565709	-1.6170909643
O17	-1.4211156341	-11.5843118511	-0.9470747891
C18	-3.4915112007	-10.8800100141	-1.8518096913
C21	-4.2692438188	-10.1514143437	-2.7250058849
C24	-5.7339844462	-10.4886573151	-2.9143394909
H25	-6.1970704617	-9.7547778667	-3.5793572507
H26	-5.8128312253	-11.4710713638	-3.4049455855
H23	-3.9521903222	-11.6419177293	-1.2245711323
H24	-3.8044477191	-9.5410477008	-3.4904483333
C25	-6.5295726666	-10.5590863100	-1.6006118650
H28	-6.0251525452	-11.2539021917	-0.9148613895
C28	-6.6958409372	-9.2052010109	-0.9067292524
H29	-7.3648769281	-8.5819482313	-1.5165651528
H30	-7.1929081850	-9.3824261558	0.0616636920
C31	-5.3459878624	-8.4484410036	-0.7275607548
H33	-4.6455020893	-9.1638725113	-0.2324243507
O34	-4.8433571187	-8.0642052794	-1.9591221343
C37	-5.5778325160	-7.7580130228	1.7412918068
C38	-5.4515024000	-7.2696129960	0.2891055932
C39	-6.5550124664	-6.2576807643	-0.0469956270
H43	-5.5504732201	-6.9179911301	2.4484484259
H44	-6.4847737722	-5.9382311022	-1.0927419794
O45	-7.8211911641	-11.1447762035	-1.9299330772
H46	-8.3433340144	-11.2062247880	-1.1071285118
H40	-4.7590979941	-8.4414462401	2.0006901332
H41	-6.5213780805	-8.2951691459	1.9100084004
H42	-6.4790886758	-5.3638043855	0.5886312648
H45	-7.5552348402	-6.6873218037	0.1104253596
H47	-4.4852365526	-6.7535643261	0.1999421662

Gas Phase Energy = -1285.5663119 hartrees

Solution Phase Energy (THF) = -1285.64478143 hartrees

Lowest energy 2,6-*cis*-*E*-boat-like transition state (TS-*cis*-1)

Cartesian Coordinates (Angstroms)

```
Atom X Y Z
--------- ------------- ------------- -------------
C1 0.5367579469 -9.8297101095 -2.8741229446
C2 0.7719220689 -8.7133936759 -2.0560585256
C3 2.0715844043 -8.4120254357 -1.6451110961
C4 3.1678433308 -9.1962912371 -2.0457805286
C5 2.9177468723 -10.3106658087 -2.8592509278
C6 1.6176056328 -10.6348679373 -3.2604452890
S7 -1.0811882703 -10.1725294003 -3.5472357172
H8 -0.0542161590 -8.0723570718 -1.7565376243
H9 2.2378118323 -7.5387285239 -1.0157245911
H11 3.7467381401 -10.9376824749 -3.1858805244
H12 1.4448936836 -11.5080764480 -3.8855953575
C12 4.5719827333 -8.8532103691 -1.6003153916
H13 4.7714000011 -9.2328610918 -0.5887634252
H14 5.3214386332 -9.2918782728 -2.2689138125
H15 4.7302409561 -7.7685935668 -1.5767292115
C16 -2.1951505311 -10.3968859475 -2.0557186732
O17 -1.7170360787 -10.4823180248 -0.9272835795
C18 -3.5573054045 -10.4962744235 -2.4854145148
C21 -4.5933363817 -10.6911325086 -1.5956914910
C24 -5.9520825200 -11.1660713834 -2.0682413138
H25 -5.9222393256 -11.3270462670 -3.1532659733
H26 -6.1466296457 -12.1463671160 -1.6041005547
H23 -3.7612930114 -10.3943325730 -3.5503403590
H24 -4.3227336794 -10.9081099942 -0.5686257947
C25 -7.1644731658 -10.2526592120 -1.7736930981
H28 -8.0573945140 -10.7643978595 -2.1537777590
C28 -7.3469466714 -9.9322928219 -0.2819666451
H29 -8.1843318027 -9.2231712683 -0.2040680903
H30 -7.6467626517 -10.8428903852 0.2585321357
C31 -6.0426102410 -9.3196500246 0.3237918625
H33 -5.5041181700 -10.1479343873 0.8428330692
O34 -5.2343811206 -8.8088865454 -0.6955008386
C37 -5.0296851444 -7.7121824919 2.0025053960
C38 -6.3370141749 -8.2871975528 1.4415122877
C39 -7.1806972307 -8.8955488298 2.5760444405
H43 -5.2262815162 -6.9288215728 2.7469322422
H44 -8.1612696622 -9.2430936640 2.2300781576
O45 -7.0499045891 -9.0258653660 -2.5193444759
H46 -6.2916860102 -8.5989769758 -2.0149884898
H40 -4.4149482828 -7.2942780830 1.2003420837
H41 -4.4409765698 -8.5008892003 2.4932164421
H42 -7.3532778718 -8.1624885926 3.3751673364
H45 -6.6618851990 -9.7552663042 3.0244593491
H47 -6.9115634447 -7.4657678692 0.9831702356
Gas Phase Energy = -1285.57900028 hartrees
Solution Phase Energy(THF) = -1285.6517652 hartrees
```

Lowest energy 2,6-*cis*-*Z*-boat-like transition state (TS-*cis*-2)

Cartesian Coordinates (Angstroms)

Atom	X	Y	Z
C1	0.1665674160	-9.4899341026	-1.7937159027
C2	0.9995122618	-10.3782193070	-1.0969304947
C3	2.3910425870	-10.2745448247	-1.2023052891
C4	2.9851370150	-9.2966346050	-2.0142662169
C5	2.1398536139	-8.4186308752	-2.7153456547
C6	0.7497923714	-8.5029767911	-2.6031928990
S7	-1.5983247508	-9.5366956616	-1.4891781140
H8	0.5599471172	-11.1437043649	-0.4611677724
H9	3.0206810297	-10.9650212547	-0.6413132728
H11	2.5745939884	-7.6434998333	-3.3459023330
H12	0.1174008142	-7.7942841199	-3.1311148688
C12	4.4898243096	-9.1938975106	-2.1425845590
H13	4.8437369875	-9.6791965066	-3.0636270185
H14	4.8174015258	-8.1478239307	-2.1824531098
H15	4.9975803125	-9.6775169462	-1.2997543793
C16	-2.3287261656	-10.0369315513	-3.1286870310
O17	-1.6509186482	-9.9418479189	-4.1605072878
C18	-3.6843400061	-10.4925314794	-3.0653500725
C21	-4.3937646983	-10.7768175464	-1.9106548371
C24	-5.6710614445	-11.5939180773	-1.9762031697
H25	-5.8556829776	-11.8926053277	-3.0155027228
H26	-5.5146188258	-12.5202419789	-1.4006247518
H23	-4.1522976306	-10.6415415636	-4.0387302825
H24	-3.8395488759	-10.8340757218	-0.9808219588
C25	-6.9606504593	-10.9221595005	-1.4523877541
H28	-7.7783489324	-11.6431765686	-1.5739208292
C28	-6.8684573954	-10.4866413093	0.0179498074
H29	-7.7943125835	-9.9408544709	0.2527644435
H30	-6.8455173871	-11.3746803580	0.6675691917
C31	-5.6173053637	-9.5829758427	0.2580117387
H33	-4.8126480711	-10.2391834499	0.6689522256
O34	-5.1876212644	-9.0261189208	-0.9533898999
C37	-4.6120558989	-7.6642620359	1.5854164193
C38	-5.8643005066	-8.5218686318	1.3609373051
C39	-6.3101430829	-9.1616648908	2.6887653563
H43	-4.2635711791	-7.2332843836	0.6428346784
H44	-7.2539142273	-9.7116282807	2.5959953071
O45	-7.2965185996	-9.7876580131	-2.2737093696
H46	-6.5624556971	-9.1603321453	-2.0077045707
H40	-3.7939697635	-8.2756141118	1.9932200846
H41	-4.8090589327	-6.8524978375	2.2983751064
H42	-6.4494736153	-8.3968287194	3.4644132989
H45	-5.5477497117	-9.8651798428	3.0543257944
H47	-6.6751715707	-7.8670001860	1.0025192262

Gas Phase Energy = -1285.57788159 hartrees

Solution Phase Energy (THF) = -1285.65168075 hartrees

Lowest energy 2,6-*cis*-*Z*-chair-like transition state (TS-*cis*-3)

Cartesian Coordinates (Angstroms)

Atom	X	Y	Z
C1	0.3690120893	-10.1369817375	-1.6693652766
C2	1.3106471186	-11.1488665951	-1.4335148515
C3	2.6693987374	-10.8367696160	-1.2998279235
C4	3.1242689867	-9.5167320068	-1.4201735725
C5	2.1720315037	-8.5135441201	-1.6796303402
C6	0.8122807338	-8.8094897992	-1.7909634997
S7	-1.3673256570	-10.5620372636	-1.6191260236
H8	0.9827250726	-12.1823532728	-1.3443899490
H9	3.3824722704	-11.6371216559	-1.1029225444
H11	2.4974968433	-7.4783484105	-1.7779674490
H12	0.0968184476	-8.0089620630	-1.9621328844
C12	4.5886259618	-9.1696826369	-1.2587452876
H13	5.2063973144	-10.0709578756	-1.1719616555
H14	4.9592156261	-8.5868038193	-2.1122925556
H15	4.7562943926	-8.5625232365	-0.3588088387
C16	-2.0709097310	-9.9731300162	-3.2504310692
O17	-1.2990195953	-9.6420676152	-4.1602301861
C18	-3.4956482442	-10.0354525127	-3.3406397220
C21	-4.3621828955	-10.3055312723	-2.2945953707
C24	-5.7903068546	-10.7427876682	-2.5249077924
H25	-6.2978401391	-10.0260054759	-3.1843922856
H26	-5.7689401584	-11.7059817597	-3.0608707420
H23	-3.8901270984	-9.8245628107	-4.3350423283
H24	-3.9313045671	-10.6052616006	-1.3440013748
C25	-6.6152663409	-10.9701772268	-1.2454649125
H28	-6.1143411936	-11.7415283086	-0.6453910323
C28	-6.8213734462	-9.7250972058	-0.3808005382
H29	-7.4943516876	-9.0327853496	-0.9088856445
H30	-7.3214775172	-10.0362739850	0.5502966698
C31	-5.4883289085	-8.9874562981	-0.0871618535
H33	-4.8090157418	-9.7571549671	0.3604431571
O34	-4.9453967247	-8.4746566844	-1.2537866156
C37	-6.5860220216	-6.7833851163	0.7090440905
C38	-5.6396560499	-7.9395265863	1.0568031446
C39	-4.2633711171	-7.4106167247	1.4854663525
H43	-6.2244414201	-6.2432850585	-0.1736702586
H44	-3.5999294165	-8.2337864616	1.7830298046
O45	-7.8871369555	-11.5857004289	-1.5868353292
H46	-8.4355831681	-10.9277325519	-2.0547410255
H40	-6.6536505188	-6.0707839097	1.5424824870
H41	-7.6023389935	-7.1369120826	0.4955821155
H42	-4.3488083080	-6.7214288171	2.3369955964
H45	-3.7802601471	-6.8811341958	0.6564195249
H47	-6.0675986722	-8.4821383095	1.9163585618

Gas Phase Energy = -1285.56884939 hartrees

Solution Phase Energy (THF) = -1285.64581591 hartrees

Lowest energy 2,6-*trans*-*E*-enolate boat conformation (*E*-13-boat)

```
Cartesian Coordinates (Angstroms)
Atom X Y Z
--------- ------------- ------------- -------------
C1 0.3339049511 -16.6241190238 2.1558640708
C2 0.9141917298 -15.8925858129 3.2035872288
C3 1.5578673614 -16.5530063070 4.2547920580
C4 1.6511571741 -17.9525939130 4.3041879937
C5 1.0631714846 -18.6759732987 3.2532949307
C6 0.4117919003 -18.0296481609 2.1993529579
S7 -0.4296085825 -15.8759737600 0.7171028274
H8 0.8587374913 -14.8083202276 3.1996024602
H9 2.0061328577 -15.9588730417 5.0499481526
H11 1.1120743776 -19.7655315473 3.2556875217
H12 -0.0356186796 -18.6226435202 1.4038748949
C12 2.3318717306 -18.6563377666 5.4590384057
H13 3.1146348102 -18.0313594343 5.9037985607
H14 1.6170926624 -18.8989315100 6.2581261324
H15 2.7958258760 -19.5979155988 5.1395037050
C16 -0.7524472541 -13.9878196642 1.0964277652
O17 -0.1275911653 -13.1998812140 0.3348325978
C18 -1.6813285432 -13.7942492988 2.0862414126
C21 -2.1445256256 -12.4375640612 2.5073492044
C24 -3.6882154144 -12.3165407495 2.5686956194
H25 -4.0713552911 -12.0576009120 1.5740626317
H26 -4.1340273549 -13.2850287974 2.8392933808
H23 -2.1232400188 -14.6620788180 2.5680349588
C25 -4.1323047323 -11.2830895745 3.6117183483
H28 -5.2218359354 -11.1699418231 3.5934552040
C28 -3.6726288538 -11.7359628391 5.0118968781
H29 -3.5946711256 -10.8504995089 5.6565898972
H30 -4.4349239101 -12.3910120235 5.4528045118
C31 -2.3168940040 -12.4795276533 4.9368638056
H33 -2.4979765195 -13.5564848254 4.7893264944
O34 -1.5830897376 -11.9857876556 3.8037342989
C37 -0.1189756667 -13.0738857625 6.0690693098
C38 -1.4509372917 -12.3204614272 6.1979292799
C39 -2.2078015544 -12.7884407287 7.4526439349
H43 0.4221048797 -12.7733980804 5.1694935892
H44 -2.4916396663 -13.8465604525 7.3634795220
O45 -3.6289106495 -9.9609526124 3.2922127623
H46 -2.6569718560 -9.9982374525 3.3785893673
H40 -0.2897659280 -14.1578363118 6.0096881258
H41 0.5192547911 -12.8810719376 6.9399105586
H42 -3.1213926369 -12.2094738327 7.6333792744
H45 -1.5728017167 -12.6896315739 8.3427020268
H47 -1.2347042588 -11.2460584498 6.3054208763
H48 -1.7436284672 -11.6943191517 1.8139320141
Gas Phase Energy = -1285.59637602 hartrees
Solution Phase Energy (THF) = -1285.6710276 hartrees
```

Lowest energy 2,6-*trans*-*Z*-enolate boat conformation (*Z*-13-boat)

```
Cartesian Coordinates (Angstroms)
Atom X Y Z
--------- ------------- ------------- -------------
C1 -1.0401764643 -7.2700257631 -3.4286631842
C2 -0.6501468909 -6.7512032845 -2.1852667530
C3 0.1010301578 -7.5268803669 -1.2954266297
C4 0.4990181068 -8.8331103478 -1.6218246380
C5 0.1142122182 -9.3405259770 -2.8732724866
C6 -0.6471446343 -8.5776775363 -3.7620588147
S7 -1.9574658054 -6.2861900019 -4.6193748765
H8 -0.9367674095 -5.7391240358 -1.9126983516
H9 0.3869083478 -7.1041870778 -0.3314858454
H11 0.3983643891 -10.3539140231 -3.1546825517
H12 -0.9486110579 -9.0027726920 -4.7156710593
C12 1.3356917139 -9.6609061206 -0.6697724789
H13 0.9630722684 -10.6914505234 -0.5968835802
H14 2.3812130994 -9.7191316163 -1.0064796469
H15 1.3382430011 -9.2297768505 0.3371017290
C16 -3.5152444098 -5.5474577038 -3.7437227604
O17 -3.6230718759 -4.3035342870 -3.9501001987
C18 -4.3410126064 -6.3968902041 -3.0467623626
C21 -4.2941105555 -7.8834072256 -2.9466082634
C24 -4.6086652498 -8.4021665592 -1.5194709858
H25 -3.6710319901 -8.5214559082 -0.9623479948
H26 -5.2139634385 -7.6641240852 -0.9744635727
H23 -5.1726923998 -5.8939292922 -2.5489385817
C25 -5.3967885945 -9.7169590055 -1.5448553454
H28 -5.5746342998 -10.0663298910 -0.5233432856
C28 -6.7399488772 -9.4962166590 -2.2633640279
H29 -7.1423125085 -10.4761460684 -2.5535434635
H30 -7.4553589157 -9.0479243449 -1.5612590590
C31 -6.5664318192 -8.5766744923 -3.5021020163
H33 -6.8343452280 -7.5422238009 -3.2334618588
O34 -5.1902935711 -8.5883569765 -3.9049349501
C37 -7.1705317470 -8.1176808682 -5.9314467716
C38 -7.4381667602 -8.9909738307 -4.6985285120
C39 -8.9301552428 -8.9466303269 -4.3259733332
H43 -6.1157032079 -8.1451584507 -6.2185343664
H44 -9.2286480045 -7.9244144132 -4.0535870762
O45 -4.6425841430 -10.7885926807 -2.1627450083
H46 -4.5128966873 -10.5244887893 -3.0941838231
H40 -7.4353388215 -7.0705757429 -5.7306970906
H41 -7.7725571977 -8.4601840591 -6.7831505928
H42 -9.1725111691 -9.6039224463 -3.4830358967
H45 -9.5493280643 -9.2591169495 -5.1771069755
H47 -7.1741838422 -10.0318160478 -4.9478050657
H48 -3.3222875763 -8.2591456937 -3.2588175964
Gas Phase Energy = -1285.59196305 hartrees
Solution Phase Energy (THF) = -1285.66609438 hartrees
```

Lowest energy 2,6-*cis*-*E*-enolate boat conformation (*E*-14-boat)

Cartesian Coordinates (Angstroms)

Atom	X	Y	Z
C1	-3.4300482422	-1.0206706413	11.5864298283
C2	-4.0061389015	0.1016357056	10.9691243711
C3	-5.2138533998	0.6299252630	11.4343625141
C4	-5.8796488526	0.0614079481	12.5326197637
C5	-5.2872204461	-1.0460336226	13.1593372924
C6	-4.0829086271	-1.5828359361	12.6953034385
S7	-1.8717257475	-1.6923178901	11.0010488326
H8	-3.5080210930	0.5566223544	10.1170957752
H9	-5.6443434479	1.4975443364	10.9338140745
H11	-5.7759309914	-1.4998164733	14.0205754510
H12	-3.6481209859	-2.4461911590	13.1915052497
C12	-7.2167708308	0.5966248389	12.9961033367
H13	-7.3041572387	1.6746996564	12.8173397958
H14	-8.0405617927	0.1060111884	12.4571885463
H15	-7.3757134825	0.4149320017	14.0665364089
C16	-2.2363382482	-2.9958305770	9.6209661332
O17	-1.1561558354	-3.5303358148	9.2369956746
C18	-3.5479579398	-3.1308213304	9.2558730045
C21	-4.0290714600	-4.1251447813	8.2618319613
C24	-5.0706163300	-3.5435428415	7.2677135924
H25	-5.4906954035	-2.6089893244	7.6590175264
H26	-4.5747552163	-3.3018041636	6.3169633251
H23	-4.3021359802	-2.5292319094	9.7530437325
C25	-6.2237789858	-4.5245302311	7.0065068743
H28	-6.7792896276	-4.2218376540	6.1125513990
C28	-5.6978765525	-5.9551410309	6.8347486936
H29	-6.5547504241	-6.6249482720	6.7031410448
H30	-5.1066999946	-6.0022159842	5.9103077210
C31	-4.8341965442	-6.3810304153	8.0620300667
H33	-3.8296807124	-6.6832972553	7.7158224624
O34	-4.6633893250	-5.2654216954	8.9484250409
C37	-4.6871765855	-7.7764608380	10.1875266868
C38	-5.4301239718	-7.5551060118	8.8617929213
C39	-5.4148818204	-8.8433256860	8.0199763625
H43	-5.1189202473	-8.6278042987	10.7298870435
H44	-5.8577654238	-9.6759606268	8.5816858405
O45	-7.2013201548	-4.4749895985	8.0767508836
H46	-6.7030169987	-4.6743565402	8.8937235958
H40	-4.7427127199	-6.8909674134	10.8269845847
H41	-3.6250718089	-7.9930009187	10.0080157835
H42	-4.3836924395	-9.1245146285	7.7630745846
H45	-5.9757249575	-8.7401736105	7.0839823351
H47	-6.4775441307	-7.3055076322	9.0920463485
H48	-3.1743851656	-4.5437687754	7.7127286338

Gas Phase Energy = -1285.59470493 hartrees

Solution Phase Energy (THF) = -1285.67170284 hartrees

Lowest energy 2,6-*cis*-*Z*-enolate boat conformation (*Z*-14-boat)

```
Cartesian Coordinates (Angstroms)
Atom X Y Z
--------- ------------- ------------- -------------
C1 4.5696140423 -6.1775455884 -6.1176086507
C2 5.2553663668 -7.0442930850 -6.9844709950
C3 5.2173072945 -8.4288428792 -6.7885383179
C4 4.4732942487 -8.9965981154 -5.7424343097
C5 3.7679648723 -8.1238753046 -4.8967122903
C6 3.8174866609 -6.7368480468 -5.0713912154
S7 4.8507697328 -4.4224011641 -6.3031782244
H8 5.8335484444 -6.6318220448 -7.8090571929
H9 5.7743132272 -9.0752442187 -7.4660300586
H11 3.1836151843 -8.5335060882 -4.0721419475
H12 3.2856689703 -6.0875075878 -4.3796460258
C12 4.4179680914 -10.4975701633 -5.5461733336
H13 5.3264730243 -10.9827955742 -5.9224189259
H14 4.3079254622 -10.7631366169 -4.4874984696
H15 3.5664872950 -10.9391901441 -6.0816432205
C16 3.1607146035 -3.5624829210 -6.0840212819
O17 2.1446511524 -4.1385482931 -6.5655381346
C18 3.2705374130 -2.3179657596 -5.4924615485
C21 4.4628076990 -1.7417382910 -4.8123743185
C24 4.0969768458 -0.6491863179 -3.7679608491
H25 3.0527017801 -0.3348752861 -3.8805844478
H26 4.2018737985 -1.0596575487 -2.7539942983
H23 2.3633105399 -1.7118070635 -5.5125655067
C25 4.9899555184 0.5936295176 -3.8977786934
H28 4.8483232304 1.2447642958 -3.0287618165
C28 6.4698522435 0.2042863828 -4.0148848939
H29 7.0452343583 1.1120221198 -4.2337357417
H30 6.8166315594 -0.1676076002 -3.0407713599
C31 6.6834400938 -0.8761092969 -5.1175721929
H33 7.0148323835 -1.8185078380 -4.6451218755
O34 5.4296456125 -1.1491416712 -5.7727479353
C37 7.8332668410 -1.5293826997 -7.2969200585
C38 7.7419275729 -0.4903078036 -6.1686047137
C39 9.1227185592 -0.3153326663 -5.5079270813
H43 6.8698956877 -1.6635727612 -7.7953526664
H44 9.1243533302 0.4545287729 -4.7267811671
O45 4.5836038257 1.4053651567 -5.0283520269
H46 4.6603188367 0.8215111757 -5.8091608147
H40 8.1432090594 -2.5074876863 -6.8998591086
H41 8.5754539861 -1.2202071466 -8.0458678222
H42 9.8712725539 -0.0260788813 -6.2582983135
H45 9.4580254629 -1.2590558210 -5.0503698926
H47 7.4414682030 0.4766269327 -6.6053440370
H48 5.0361346653 -2.5390879256 -4.3204373798
Gas Phase Energy = -1285.5918311 hartrees
Solution Phase Energy (THF) = -1285.67031384 hartrees
```

Lowest energy 2,6-*trans*-*E*-enolate chair conformation (*E*-13-chair)

```
Cartesian Coordinates (Angstroms)
Atom X Y Z
--------- ------------- ------------- -------------
C1 -6.3046531592 -16.2414751819 -3.9950803372
C2 -7.6892801584 -16.0090779787 -4.0565541194
C3 -8.3019806248 -15.6599013771 -5.2650443410
C4 -7.5593411048 -15.5371292043 -6.4493828151
C5 -6.1768884203 -15.7823015531 -6.3850389223
C6 -5.5572088280 -16.1270366389 -5.1789318227
S7 -5.5267990238 -16.7517478401 -2.4574651547
H8 -8.2879413925 -16.1097373455 -3.1546401839
H9 -9.3776896345 -15.4884445531 -5.2880976388
H11 -5.5738344738 -15.7052915125 -7.2903197934
H12 -4.4872765080 -16.3121765978 -5.1536326880
C12 -8.2229171169 -15.1372345979 -7.7501999446
H13 -9.3025351142 -15.3255771735 -7.7222097794
H14 -8.0812222977 -14.0659660850 -7.9561370122
H15 -7.8054191944 -15.6885762577 -8.6013574363
C16 -5.2063298547 -15.1781097367 -1.3694254363
O17 -4.8895087616 -15.5154535204 -0.1966176539
C18 -5.3295225299 -13.9763756692 -2.0160065956
C21 -5.2285267923 -12.6597372662 -1.3104598866
C24 -4.5450355809 -11.5516556907 -2.1458763259
H25 -4.3564032494 -10.6823323113 -1.5011582981
H26 -3.5771382945 -11.9152264920 -2.5157356188
H23 -5.5857440968 -13.9871592928 -3.0679865909
C25 -5.4248553326 -11.0947772468 -3.3137815115
H28 -5.5492526171 -11.9176949115 -4.0315431627
C28 -6.8008319099 -10.6742147438 -2.7978366969
H29 -6.6934065377 -9.7602479134 -2.1976745483
H30 -7.4666466022 -10.4488114625 -3.6417971425
C31 -7.4056270108 -11.8050461764 -1.9402604129
H33 -7.5032144392 -12.6969099548 -2.5801416849
O34 -6.5275066504 -12.1255620428 -0.8585824773
C37 -8.8369171031 -10.4023148576 -0.3167784094
C38 -8.8134512695 -11.5049490334 -1.3859988148
C39 -9.4850782729 -12.7863195401 -0.8612506290
H43 -8.3911268298 -9.4680566694 -0.6784558462
H44 -8.9305471503 -13.2054777744 -0.0129650441
O45 -4.8398990978 -9.9749142020 -4.0243430571
H46 -4.0105845601 -10.2737684979 -4.4388914909
H40 -8.2877078329 -10.7158276898 0.5794402512
H41 -9.8711755225 -10.1843862238 -0.0204755505
H42 -9.5338923975 -13.5578118385 -1.6411506599
H45 -10.5112757340 -12.5795497326 -0.5293433567
H47 -9.4009412758 -11.1530751501 -2.2487798935
H48 -4.6843798874 -12.7867090686 -0.3706499382
Gas Phase Energy = -1285.59914778 hartrees
Solution Phase Energy (THF) = -1285.67669649 hartrees
```

Lowest energy 2,6-*trans*-*Z*-enolate chair conformation (*Z*-13-chair)

```
Cartesian Coordinates (Angstroms)
Atom X Y Z
--------- ------------- ------------- -------------
C1 -2.7861137295 -7.8497128094 0.8056090950
C2 -2.3247932736 -6.7492417784 1.5440321532
C3 -2.8259184473 -5.4686663924 1.2868444884
C4 -3.8018721174 -5.2396137206 0.3042056021
C5 -4.2602460538 -6.3487418597 -0.4239202085
C6 -3.7582557787 -7.6304254970 -0.1884916570
S7 -2.2902819351 -9.5397925587 1.1071233086
H8 -1.5792638578 -6.8906681533 2.3202297752
H9 -2.4509652190 -4.6306919310 1.8748608968
H11 -5.0235193342 -6.2116939586 -1.1900025727
H12 -4.1358005382 -8.4694672965 -0.7721643126
C12 -4.3212089526 -3.8468982757 0.0150866054
H13 -4.2965105025 -3.2125166024 0.9102044817
H14 -3.7178067087 -3.3460796716 -0.7566557823
H15 -5.3551445406 -3.8734716851 -0.3500097466
C16 -0.7021352050 -9.4563804741 2.1792290145
O17 0.2806592257 -8.8442264103 1.6606859664
C18 -0.7657124194 -10.1865770296 3.3445322235
C21 -1.9657335053 -10.8084811048 3.9955794003
C24 -1.7938480303 -12.2909871952 4.4084096968
H25 -2.7816825891 -12.7004242745 4.6622682718
H26 -1.4001364591 -12.8628595848 3.5576341296
H23 0.1906347192 -10.2683080688 3.8628489904
C25 -0.8897852879 -12.4551658138 5.6315417349
H28 0.1352224251 -12.1451863376 5.3815907603
C28 -1.4085014699 -11.5901579758 6.7794054619
H29 -2.3759429570 -11.9868427588 7.1154599254
H30 -0.7135796674 -11.6289428903 7.6290266921
C31 -1.5667713670 -10.1329174149 6.3011701911
H33 -0.5730529420 -9.7719766659 5.9884915719
O34 -2.4466054293 -10.0743377952 5.1768349359
C37 -3.4951524052 -9.4313200758 7.8762157749
C38 -2.0653150068 -9.1532882788 7.3841590432
C39 -1.9303007212 -7.6967191799 6.9091481843
H43 -3.7608067694 -8.7306828306 8.6784751562
H44 -2.5951995459 -7.4995646032 6.0609273256
O45 -0.8408165191 -13.8319041420 6.0823779382
H46 -0.4649584087 -14.3780907085 5.3650005553
H40 -3.6064193046 -10.4459817541 8.2753470464
H41 -4.2213075281 -9.3045306043 7.0654236148
H42 -0.9044209135 -7.4751137508 6.5880981725
H45 -2.1892510434 -6.9997642627 7.7167177894
H47 -1.3815886217 -9.2881882139 8.2362216947
H48 -2.8166379528 -10.7417422704 3.3181032681
Gas Phase Energy = -1285.59200157 hartrees
Solution Phase Energy (THF) = -1285.67285263 hartrees
```

Lowest energy 2,6-*cis*-*E*-enolate chair conformation (*E*-14-chair)

```
Cartesian Coordinates (Angstroms)
Atom X Y Z
--------- ------------- ------------- -------------
C1 -2.0720881261 -3.7039012717 9.9669374774
C2 -0.6688882491 -3.6817746253 9.9016854566
C3 0.0323458758 -2.4798107983 10.0447065332
C4 -0.6349635024 -1.2670467196 10.2717297375
C5 -2.0380316021 -1.2963021972 10.3439528181
C6 -2.7471872903 -2.4909115634 10.1870869555
S7 -2.9918221889 -5.2433922645 9.8835755983
H8 -0.1235589129 -4.6091371211 9.7440033952
H9 1.1205790625 -2.4900261316 9.9907609473
H11 -2.5875190989 -0.3723772470 10.5234849134
H12 -3.8345907494 -2.4878609185 10.2422923194
C12 0.1271864264 0.0340260472 10.4162515263
H13 -0.2637586032 0.6413813618 11.2421633260
H14 1.1921539214 -0.1439769065 10.6030175847
H15 0.0503785440 0.6433142651 9.5047141803
C16 -3.0297295028 -5.9407491686 8.0575648541
O17 -3.6389044987 -7.0484232781 8.0595181540
C18 -2.4409098291 -5.1447411077 7.1190864884
C21 -2.3423807715 -5.3745314127 5.6402266883
C24 -2.7455841190 -6.7603065084 5.1160554151
H25 -2.1312135747 -7.5263180168 5.6031511097
H26 -3.7926078128 -6.9617608537 5.3725115843
H23 -2.0147443095 -4.2019409791 7.4455077525
C25 -2.5446562380 -6.8243978492 3.6001440475
H28 -3.2318994714 -6.1108866018 3.1196612786
C28 -1.1096513136 -6.4454056277 3.2328258042
H29 -0.4294737870 -7.2153504611 3.6193190110
H30 -0.9933567499 -6.4058752690 2.1404775253
C31 -0.7609089573 -5.0751267683 3.8460356764
H33 -1.4508071264 -4.3280898959 3.4070489442
O34 -0.9646636927 -5.0988733348 5.2591519753
C37 1.7618240575 -5.4445584063 4.2353854715
C38 0.6762324920 -4.5854389123 3.5652168119
C39 0.8400559857 -3.1081515558 3.9633347373
H43 1.6613170973 -5.4167986857 5.3262311961
H44 0.1215729196 -2.4673297813 3.4366183248
O45 -2.8217327620 -8.1416923735 3.0675881227
H46 -3.7266324929 -8.3965451071 3.3314722389
H40 2.7580812519 -5.0646974834 3.9735000346
H41 1.7151715024 -6.4930890904 3.9181127490
H42 1.8485555092 -2.7499197004 3.7187818060
H45 0.6851996473 -2.9745172477 5.0394635286
H47 0.8104366218 -4.6535887636 2.4744901415
H48 -2.9537786067 -4.6209304285 5.1027844799
Gas Phase Energy = -1285.59893675 hartrees
Solution Phase Energy (THF) = -1285.67444488 hartrees
```

Lowest energy 2,6-*cis*-*Z*-enolate chair conformation (*Z*-14-chair)

```
Cartesian Coordinates (Angstroms)
Atom X Y Z
--------- ------------- ------------- -------------
C1 2.8292418165 -3.1257988813 -7.1035614498
C2 3.3780071525 -3.6791556078 -5.9332890283
C3 4.7628992424 -3.7257651077 -5.7496603094
C4 5.6475214190 -3.2523833144 -6.7349713767
C5 5.0941180802 -2.7201643344 -7.9090005945
C6 3.7090813956 -2.6499982455 -8.0915279041
S7 1.0608932895 -3.1545892492 -7.4478763976
H8 2.7172110288 -4.0682371109 -5.1624084671
H9 5.1639687256 -4.1487015228 -4.8274842679
H11 5.7528260801 -2.3444960708 -8.6925252904
H12 3.3077507507 -2.2193228016 -9.0053577367
C12 7.1473574435 -3.3450979203 -6.5525494412
H13 7.6683354023 -2.5464357284 -7.0963321887
H14 7.4296166602 -3.2765459480 -5.4954691024
H15 7.5333110909 -4.3016859034 -6.9304230351
C16 -0.1148940311 -2.1791807595 -6.2499426258
O17 -1.2704464801 -2.6773120424 -6.3874245740
C18 0.3021368651 -1.1212875962 -5.4840958124
C21 1.6498970731 -0.4969265122 -5.4025859644
C24 2.0439479265 -0.1065598248 -3.9641868628
H25 1.2951909469 0.5918965715 -3.5633667678
H26 2.0397418586 -1.0062131080 -3.3315008656
H23 -0.4893630428 -0.6632343350 -4.8859744123
C25 3.4244155691 0.5528046504 -3.9313448647
H28 4.1816687744 -0.1958721732 -4.2139625473
C28 3.5033009286 1.7145392274 -4.9229662394
H29 2.8565035819 2.5361027526 -4.5825273543
H30 4.5331902964 2.0894513923 -4.9593879288
C31 3.0409980145 1.2499380340 -6.3178294375
H33 3.7163678245 0.4428256371 -6.6582035999
O34 1.7126001515 0.7157939873 -6.2300965458
C37 2.6053066284 1.7548945514 -8.7586736950
C38 3.0381697277 2.3406779772 -7.4047659715
C39 4.4074895684 3.0253082549 -7.5468200102
H43 3.3388728260 1.0192642187 -9.1169119464
H44 4.6904363197 3.5877832516 -6.6512260300
O45 3.7606292365 1.0538134869 -2.6150131006
H46 3.7591915452 0.3029282479 -1.9907703296
H40 2.5321100447 2.5431581237 -9.5158934875
H41 1.6378592037 1.2538641634 -8.6833928560
H42 4.3918990510 3.7282445745 -8.3872549016
H45 5.1966652063 2.2891276672 -7.7506925522
H47 2.3011354972 3.0988363302 -7.1036112700
H48 2.4107608303 -1.1740976543 -5.8032226432
Gas Phase Energy = -1285.59586242 hartrees
Solution Phase Energy (THF) = -1285.67353022 hartrees
```

Lowest energy 2,6-*trans*-4-alkoxide chair conformation

```
Cartesian Coordinates (Angstroms)
Atom X Y Z
--------- ------------- ------------- -------------
C1 -7.5172762160 -4.2110626548 3.0801622182
C2 -7.5814237125 -5.1804293963 4.0928028143
C3 -7.6983238280 -4.7853040841 5.4280757791
C4 -7.7367762753 -3.4271072988 5.7891610303
C5 -7.6671606882 -2.4694928265 4.7647550923
C6 -7.5651911646 -2.8516752749 3.4236621164
S7 -7.2059359054 -4.6481661823 1.3660680573
H8 -7.5298178406 -6.2379772105 3.8461165994
H9 -7.7444428341 -5.5491396491 6.2040276371
H11 -7.6894414632 -1.4091428448 5.0146633281
H12 -7.5086934734 -2.0922024535 2.6471217749
C12 -7.8648529587 -3.0102736946 7.2378836722
H13 -8.9194864884 -2.9835032854 7.5479002204
H14 -7.3470402642 -3.7100803002 7.9054654182
H15 -7.4492803416 -2.0094284029 7.4044322485
C16 -8.7045550529 -5.4769459241 0.7745267607
O17 -9.6630201684 -5.7142234223 1.4855785614
C18 -8.6419517287 -5.8216290214 -0.7096299628
C21 -8.8650175455 -7.3366018391 -0.9774275751
C24 -9.1631244593 -7.6906828280 -2.4542998454
H25 -9.6525121786 -8.6756358411 -2.4598905399
H26 -9.8858782648 -6.9712932754 -2.8626330530
H23 -7.7082051043 -5.4615659014 -1.1537282925
C25 -7.9331424618 -7.7986746760 -3.3999991615
H28 -7.4806439031 -6.7759291873 -3.4497047655
C28 -6.8883348820 -8.6759084502 -2.6587312695
H29 -7.2748165088 -9.7037584848 -2.6021385745
H30 -5.9555331104 -8.7042378187 -3.2412646604
C31 -6.5851300728 -8.1463214955 -1.2485844276
H33 -6.2092237501 -7.1133451118 -1.3503084933
O34 -7.7854687849 -8.1181171002 -0.4454773799
C37 -5.9205609303 -10.3660009888 -0.1102009278
C38 -5.5091494566 -8.9273943894 -0.4631190016
C39 -5.0676598198 -8.1702778523 0.8019380879
H43 -6.1789725243 -10.9509947256 -0.9999480950
H44 -4.2397770164 -8.6944127145 1.2978523422
O45 -8.2654869861 -8.2605073563 -4.6662646059
H40 -6.7866881134 -10.3716533068 0.5619630204
H41 -5.0952036590 -10.8809075963 0.3983918765
H42 -5.8925286933 -8.0836742548 1.5187538491
H45 -4.7276648309 -7.1540886472 0.5642674087
H47 -4.6407865846 -8.9778917544 -1.1376166099
H46 -9.7286013039 -7.6331696035 -0.3722927620
H48 -9.4634052869 -5.2616822796 -1.1783923104
Gas Phase Energy = -1285.57154298 hartrees
Solution Phase Energy (THF) = -1285.6743985 hartrees
```

Lowest energy 2,6-*cis*-4-alkoxide chair conformation

Cartesian Coordinates (Angstroms)

Atom X Y Z

--------- ------------- ------------- -------------

C1 -8.2791757312 -9.8123757509 -3.7129806383
C2 -8.8450446056 -8.5303286419 -3.7007349958
C3 -9.5880553327 -8.1037680287 -2.5943802244
C4 -9.7789996632 -8.9364426152 -1.4798836553
C5 -9.2053305556 -10.2186346930 -1.5074438948
C6 -8.4673728479 -10.6578126921 -2.6094804910
S7 -7.4253132259 -10.4159571018 -5.1749625424
H8 -8.7189134238 -7.8695382868 -4.5548235621
H9 -10.0286271804 -7.1073974479 -2.6030749923
H11 -9.3468883980 -10.8896365367 -0.6610970038
H12 -8.0493843545 -11.6615196660 -2.6166919592
C12 -10.5634420915 -8.4636738501 -0.2753061319
H13 -11.0500205742 -9.3002753071 0.2393231156
H14 -9.9045178025 -7.9719715878 0.4542113644
H15 -11.3367322264 -7.7395087648 -0.5565237500
C16 -5.6627194234 -10.2940614891 -4.7259185914
O17 -5.2985396097 -9.7863090327 -3.6853362175
C18 -4.7135196609 -10.9521070246 -5.7220220463
C21 -4.6591791679 -10.3391615223 -7.1349903604
C24 -3.5315603785 -10.9664064487 -7.9796451531
H25 -2.5911767983 -10.9122275803 -7.4105654033
H26 -3.7543770682 -12.0287889625 -8.1529831713
H23 -4.9959746469 -12.0088745232 -5.8143743394
C25 -3.3052531694 -10.2580799705 -9.3493321903
H28 -4.2619856170 -10.3913318340 -9.9165033479
C28 -3.2136547919 -8.7324903395 -9.0601497929
H29 -2.2686062714 -8.5343405487 -8.5356086448
H30 -3.1841307081 -8.1844749764 -10.0134850084
C31 -4.3980227711 -8.2256111774 -8.2193865434
H33 -5.3286107864 -8.4463954846 -8.7754020349
O34 -4.4635173909 -8.9353163210 -6.9683967835
C37 -3.2578703847 -6.2749163711 -6.9823822014
C38 -4.4068752120 -6.7149702050 -7.9044036610
C39 -5.7602477188 -6.2758365250 -7.3177602938
H43 -3.2857990226 -5.1874468713 -6.8343319603
H44 -5.7813476709 -5.1897445589 -7.1558511558
O45 -2.2262148255 -10.7668626649 -10.0574710396
H40 -2.2760319194 -6.5278399374 -7.3976080962
H41 -3.3433462922 -6.7528266567 -5.9999706519
H42 -5.9454585435 -6.7668652328 -6.3552709889
H45 -6.5901040588 -6.5302223431 -7.9896451559
H47 -4.2820688944 -6.2093187427 -8.8737961453
H46 -5.6273238116 -10.5000206277 -7.6426844547
H48 -3.7202951952 -10.8998595008 -5.2639681195
Gas Phase Energy = -1285.57565063 hartrees
Solution Phase Energy (THF) = -1285.67750754 hartrees

Calculations on TFA mediated cyclizations

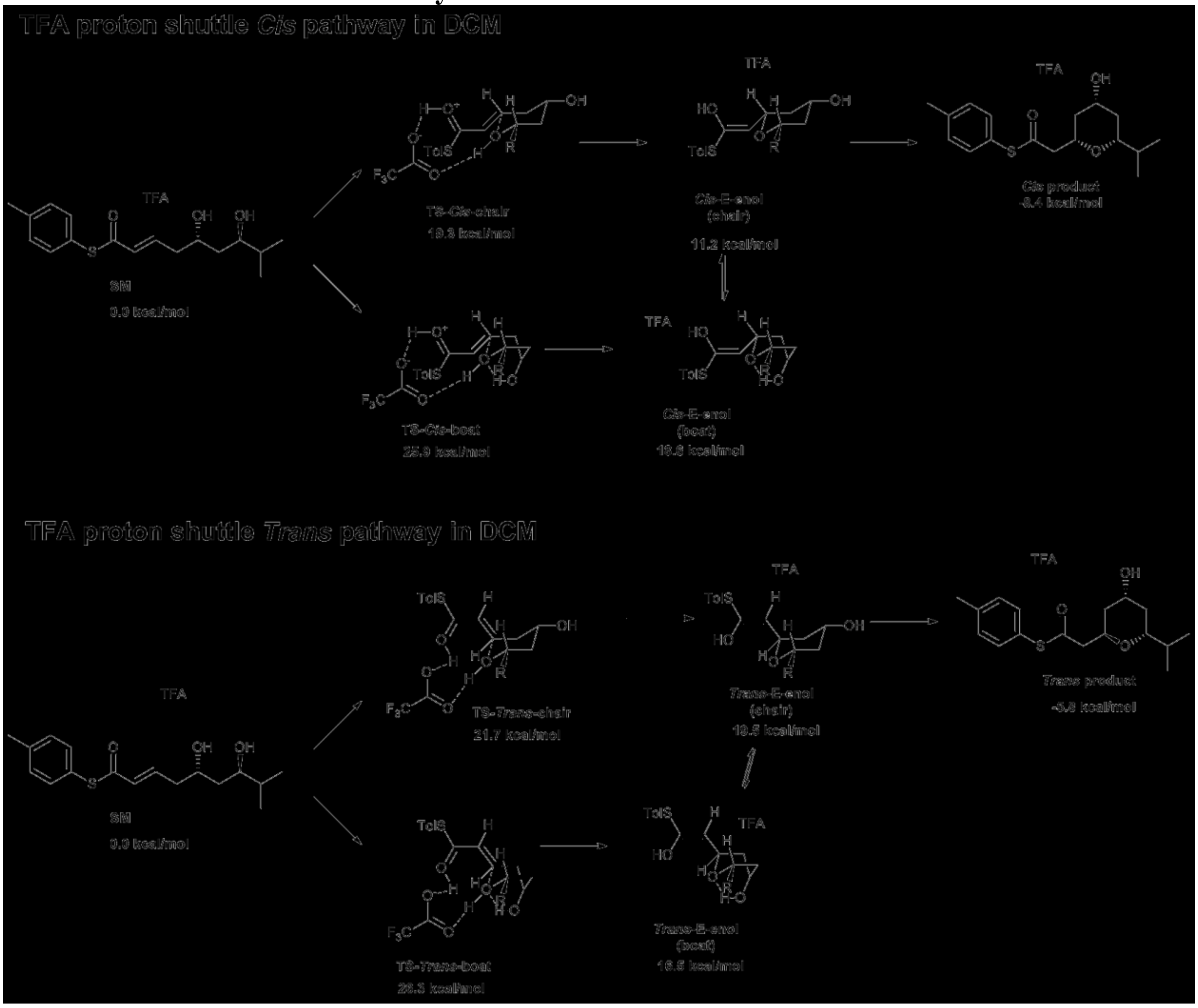

Figure S2. *Overview of TFA mediated cyclization pathways explored in this study*

Lowest energy conformation of diol 7a complex with TFA

Cartesian Coordinates (Angstroms)

Atom	X	Y	Z
C1	1.0461170193	-7.2647092023	0.9086230495
C2	2.1464286796	-6.4692209494	1.2575948037
C3	3.1114887653	-6.1516116431	0.2966962373
C4	3.0090191577	-6.6256604231	-1.0206354819
C5	1.8969240776	-7.4186208023	-1.3567459152
C6	0.9259929355	-7.7423006746	-0.4065492078
S7	-0.0783391888	-7.8377438452	2.1898257696
H8	2.2587128985	-6.1143452579	2.2795704576
H9	3.9655369199	-5.5401518683	0.5841812893
H11	1.7959637221	-7.8027558019	-2.3715742218
H12	0.0861071883	-8.3766941439	-0.6802791056
C12	4.0723257259	-6.3144699320	-2.0504405525
H13	3.6497818721	-5.7783555889	-2.9107118826
H14	4.8722966506	-5.6975865844	-1.6264508691
H15	4.5267673849	-7.2362650787	-2.4381961775
C16	-1.3921985216	-6.6124980829	2.4319173109
O17	-2.1265502969	-6.8129117577	3.3950420624
C18	-1.5274732825	-5.4918781292	1.4871607570
C21	-2.3443549673	-4.4566274110	1.7571378177
C24	-2.5143302373	-3.2563236314	0.8719981162
H25	-1.8346233417	-2.4701863313	1.2369691024
H26	-2.2026996219	-3.4803505084	-0.1569143361
H23	-0.9371606086	-5.5224791665	0.5760599708
H24	-2.8954767675	-4.4589842685	2.6985351479
C25	-3.9222294694	-2.6513694212	0.8993099263
H28	-4.1580639807	-2.3801780548	1.9362361853
C28	-4.0355909366	-1.4267709513	-0.0185147866
H29	-3.1951319821	-0.7507751471	0.1802783450
H30	-3.9142266074	-1.7567548856	-1.0592989696
C31	-5.3525404792	-0.6330716030	0.1015569916
H33	-5.3414388326	0.1492310117	-0.6722876716
O34	-6.4880105489	-1.5066313586	-0.1444078058
C37	-6.8643351400	0.9128875340	1.4180996463
C38	-5.5940072431	0.0469800753	1.4646211581
C39	-4.3904268709	0.8986936980	1.9089090735
H43	-7.7375638967	0.3293836659	1.1098568699
H44	-3.5032713826	0.2944541500	2.1306596932
O45	-4.8840719438	-3.6777294772	0.5077095146
H46	-5.7649910105	-3.2379324647	0.5079664066
H40	-6.7403603528	1.7430712462	0.7081635147
H41	-7.0720067520	1.3449294013	2.4050693998
H42	-4.6404888711	1.4562826139	2.8196671584
H45	-4.1154536435	1.6318410762	1.1371913282
H47	-5.7522331252	-0.7444159907	2.2128172074
H48	-6.4995373471	-1.7527096548	-1.0888335046
C46	-5.1379457061	-4.7045505760	-4.2295663929
F47	-5.8418624329	-4.0711538221	-5.1783245439
F48	-5.5190104350	-5.9983467323	-4.2139013680
F49	-3.8316984765	-4.6614802647	-4.5780497658
C50	-5.3468983968	-4.0396896078	-2.8393462780
O51	-6.0053273389	-3.0238941750	-2.7208069101
O52	-4.7137893695	-4.7136731487	-1.9096595255
H53	-4.7966362141	-4.2896897086	-0.9801716186

Gas Phase Energy = -1812.98613635 hartrees

Solution Phase Energy (DCM) = -1813.00657457 hartrees

Lowest energy 2,6-*cis*-chair-like transition state (TS-*cis*-chair)

Cartesian Coordinates (Angstroms)

Atom	X	Y	Z
C1	-0.8243922210	-6.5708566953	-3.1077384353
C2	-1.6468705405	-7.0529582885	-2.0811810799
C3	-2.7311164954	-7.8779599907	-2.3900863445
C4	-3.0141730904	-8.2390492788	-3.7179601779
C5	-2.1821496790	-7.7412032916	-4.7350830440
C6	-1.0900819524	-6.9221292601	-4.4386076243
S7	0.6590536942	-5.6387069742	-2.7073979579
H8	-1.4429100671	-6.7851208840	-1.0484989751
H9	-3.3640072825	-8.2462808920	-1.5834995752
H11	-2.3815816476	-8.0060484434	-5.7726364720
H12	-0.4443179725	-6.5640249887	-5.2371060045
C12	-4.1639280266	-9.1667954945	-4.0385289235
H13	-4.4595161743	-9.0890091012	-5.0905673608
H14	-3.8845340419	-10.2128442876	-3.8497344895
H15	-5.0404866471	-8.9481422905	-3.4175543723
C16	0.2484212088	-3.9344240194	-3.0419070064
O17	-1.0047094460	-3.5246613711	-2.9882980842
C18	1.2906622029	-3.0764922206	-3.3205774327
C21	1.0933574704	-1.6639045101	-3.3299056129
C24	2.0944835394	-0.7754243901	-4.0138704842
H25	3.1180860677	-1.1105800499	-3.8002140615
H26	1.9259487880	-0.9150808092	-5.0934744765
H23	2.2917069289	-3.4788755047	-3.4562235246
H24	0.0593133272	-1.3277137345	-3.3826401145
C25	1.9440310802	0.7142373533	-3.6663958548
H28	0.9126440586	1.0329086439	-3.8812665861
C28	2.2579616925	0.9867762887	-2.1918760106
H29	2.1936527176	2.0657499963	-2.0125993031
H30	3.2892029347	0.6759994122	-1.9801813593
C31	1.2829872492	0.2829604490	-1.2474278794
H33	0.2570158566	0.6091059731	-1.4684209780
O34	1.3530383415	-1.1554167396	-1.5611747376
C37	1.2670481131	1.9140630017	0.7019118326
C38	1.5359988057	0.4627443687	0.2675037512
C39	2.9225195026	-0.0112998700	0.7324316402
H43	0.2786227494	2.2594515811	0.3736773424
H44	3.1290424398	-1.0374114023	0.4100397643
O45	2.8488195053	1.5079075871	-4.4543601047
H46	2.5739953667	1.4722823507	-5.3911117425
H40	2.0212134653	2.6053687376	0.3055991349
H41	1.2981822220	1.9846907393	1.7959735594
H42	2.9713293259	0.0102187190	1.8281582620
H45	3.7240858494	0.6353139162	0.3543884848
H47	0.7852569397	-0.1676442303	0.7632291229
H48	0.6569075666	-1.6494441363	-1.0391605767
C46	-2.0917143245	-3.3076570476	1.5846406747
F47	-3.4217521984	-3.0413253065	1.5892515081
F48	-1.5230479229	-2.5154077006	2.5161044374
F49	-1.9394661600	-4.5906272152	2.0024250721
C50	-1.4808730173	-3.1136446431	0.1591601041
O51	-0.5049697492	-2.3453232443	0.0648487327

O52 -2.0726277903 -3.7912576522 -0.7268345487
H53 -1.4779041176 -3.7201865672 -2.0687960879
Gas Phase Energy = -1812.95074862 hartrees
Solution Phase Energy (DCM) = -1812.97587168 hartrees

Lowest energy 2,6-*cis*-boat-like transition state (TS-*cis*-boat)
Cartesian Coordinates (Angstroms)
Atom X Y Z
--------- ------------- ------------- -------------
C1 -0.4727840031 -6.3884151385 -3.1958380715
C2 -1.0237590226 -6.6247985550 -4.4637562192
C3 -1.9875024946 -7.6220261829 -4.6230013286
C4 -2.4000526613 -8.4189048629 -3.5397312957
C5 -1.8394880520 -8.1659093554 -2.2788131745
C6 -0.8880512701 -7.1575908811 -2.1015301738
S7 0.8927700010 -5.2482057083 -2.9625184742
H8 -0.6959795125 -6.0422721522 -5.3220338334
H9 -2.4117050209 -7.7959349016 -5.6108933098
H11 -2.1475187807 -8.7615212162 -1.4202591517
H12 -0.4653207231 -6.9762608154 -1.1170000313
C12 -3.3895799230 -9.5443064279 -3.7384469041
H13 -3.9258656474 -9.7761202963 -2.8112812166
H14 -4.1268880118 -9.2993769388 -4.5113939212
H15 -2.8744470407 -10.4615124166 -4.0577410288
C16 0.2434276494 -3.6127739766 -3.2609464152
O17 -1.0613574801 -3.3751599987 -3.1945039578
C18 1.1528348069 -2.6251789785 -3.5396640436
C21 0.7836298670 -1.2323203149 -3.5102677494
C24 1.6786923633 -0.2258120443 -4.2284201929
H25 2.2653067406 -0.7602841050 -4.9837022717
H26 1.0496960322 0.5020886980 -4.7557095436
H23 2.1919104394 -2.8936262028 -3.7132636730
H24 -0.2841623603 -1.0403216198 -3.5972280838
C25 2.6517868278 0.5541064170 -3.3207956324
H28 3.2382028785 1.2169339521 -3.9638452950
C28 1.9136018539 1.4189330966 -2.2813825707
H29 2.6477081202 1.7393253613 -1.5348893560
H30 1.5475256723 2.3254466564 -2.7784250088
C31 0.7165527239 0.7107829269 -1.6192051548
H33 -0.2106272692 0.9412034328 -2.1586988181
O34 0.9433773312 -0.7430781985 -1.8228563856
C37 0.1353572692 2.4913594111 0.0561790266
C38 0.4915881475 1.0039027371 -0.1241656956
C39 1.6608950214 0.5898005650 0.7837409955
H43 -0.7157785699 2.7816874838 -0.5726955406
H44 1.9132072308 -0.4694784423 0.6659823569
O45 3.6362743232 -0.3080717198 -2.7191865285
H46 3.2113753698 -0.8731276303 -2.0482383443
H40 0.9811072371 3.1461006117 -0.1892598592
H41 -0.1393050567 2.6831803021 1.1001426464
H42 1.3828560055 0.7469623717 1.8327624326
H45 2.5643549256 1.1817883588 0.5919193233
H47 -0.3862409972 0.4169066233 0.1745973407
H48 0.2981224257 -1.3054325949 -1.2833077409
C46 -1.8732498730 -3.3492750065 1.4749516221
F47 -1.4062470499 -4.5724547092 1.8396666011
F48 -3.2205908263 -3.3773348504 1.6172815053

F49 -1.3957692733 -2.4585560724 2.3688075740
C50 -1.4576205386 -3.0275523243 0.0028876770
O51 -0.5852128559 -2.1445774099 -0.1418277194
O52 -2.0514682795 -3.7314495108 -0.8534896577
H53 -1.4834369300 -3.6168257735 -2.2856999048
Gas Phase Energy = -1812.94164479 hartrees
Solution Phase Energy (DCM) = -1812.96534111 hartrees

Lowest energy 2,6-*trans*-chair-like transition state (TS-*trans*-chair)
Cartesian Coordinates (Angstroms)
Atom X Y Z
--------- ------------- ------------- -------------
C1 -2.2523180423 -4.8552045612 -4.4660861729
C2 -2.0743068342 -6.2268930348 -4.6850160135
C3 -3.0985616570 -7.1201301792 -4.3605266423
C4 -4.3160570719 -6.6678985176 -3.8256211125
C5 -4.4805315420 -5.2880513504 -3.6223485618
C6 -3.4632046219 -4.3849832526 -3.9404482182
S7 -0.9755284872 -3.6794261383 -4.9382159572
H8 -1.1395980053 -6.5961855330 -5.0994328742
H9 -2.9465347171 -8.1857679205 -4.5264114185
H11 -5.4153276409 -4.9121485932 -3.2086448207
H12 -3.6113721445 -3.3201673486 -3.7779679839
C12 -5.4319427870 -7.6367729620 -3.5050090928
H13 -6.0922773792 -7.7730476431 -4.3730289018
H14 -5.0410172861 -8.6239415182 -3.2336543904
H15 -6.0521035785 -7.2738658180 -2.6777279422
C16 0.0530606515 -3.5584519829 -3.4814720398
O17 -0.1589498057 -4.5131951864 -2.6041020911
C18 0.9630446005 -2.5201512743 -3.4234683695
C21 1.8986203581 -2.3338568531 -2.3523155505
C24 3.1630433098 -1.5182785552 -2.5562285033
H25 3.7433404446 -2.0014653398 -3.3542159651
H26 3.7612562939 -1.5729556455 -1.6379824503
H23 0.9522310423 -1.8161067554 -4.2522872377
H24 2.0539561925 -3.1907191240 -1.6997490896
C25 2.9459558563 -0.0373919816 -2.9257698209
H28 2.4807495755 0.0341583162 -3.9158676022
C28 2.0818985246 0.7183950199 -1.9018662285
H29 2.6579091649 0.8418915205 -0.9744578053
H30 1.8646955498 1.7190923362 -2.2946584428
C31 0.7617893674 0.0096741609 -1.5833978965
H33 0.2017850635 -0.1665553430 -2.5113570167
O34 1.1652435053 -1.3063941222 -1.0712713714
C37 0.3812390071 0.8953125487 0.8198129136
C38 -0.1662943065 0.7681218278 -0.6118828018
C39 -1.5988811176 0.2012356422 -0.6159017527
H43 0.4270528514 -0.0793008559 1.3189140597
H44 -2.0018155924 0.1469550148 -1.6355915041
O45 4.2191395104 0.6146089511 -3.0916767683
H46 4.6927011184 0.6284149960 -2.2360740068
H40 -0.2805167324 1.5404326572 1.4113117826
H41 1.3827629492 1.3408036118 0.8435120319
H42 -2.2610451344 0.8513795515 -0.0305862067
H45 -1.6478537413 -0.7997220172 -0.1730944798
H47 -0.2204294972 1.7784356685 -1.0451149471
H48 0.4693973941 -1.8434382953 -0.5591221429

C46 -0.0018283612 -4.6231821740 2.0670625556
F47 1.2188961717 -4.8795657957 2.6055202296
F48 -0.6534370629 -5.8063485366 1.9687523244
F49 -0.6780039720 -3.8632782406 2.9496332644
C50 0.1626056673 -3.9476016814 0.6714695637
O51 -0.2488971012 -2.7716270882 0.5604769180
O52 0.7221514152 -4.6753135173 -0.1892175866
H53 0.2724631022 -4.4236365748 -1.6869527200
Gas Phase Energy = -1812.94756607 hartrees
Solution Phase Energy (DCM) = -1812.97197726 hartrees

Lowest energy 2,6-*trans*-boat-like transition state (TS-*trans*-boat)

Cartesian Coordinates (Angstroms)
Atom X Y Z
--------- ------------- ------------- -------------
C1 -2.1479627691 -5.3764168001 -3.8106785550
C2 -2.9487879182 -5.6697882815 -2.6983194157
C3 -3.4917420782 -6.9480246095 -2.5488917467
C4 -3.2574207176 -7.9519719026 -3.5028451639
C5 -2.4462520824 -7.6433359550 -4.6086767285
C6 -1.8983528087 -6.3678434194 -4.7700862045
S7 -1.6038480086 -3.6879438477 -4.0928636063
H8 -3.1507087163 -4.9033349395 -1.9555224392
H9 -4.1120097927 -7.1634930550 -1.6793341163
H11 -2.2483191008 -8.4058396273 -5.3610716517
H12 -1.2894194479 -6.1422677726 -5.6428053094
C12 -3.8963471401 -9.3149668533 -3.3612297674
H13 -3.9820590886 -9.6101308420 -2.3087470933
H14 -4.9118620861 -9.3124159110 -3.7826091832
H15 -3.3238111072 -10.0849984827 -3.8903224073
C16 0.0459722363 -3.5710345938 -3.4128668687
O17 0.4810129355 -4.4749079344 -2.5440105690
C18 0.8144746950 -2.5050914207 -3.8054070726
C21 2.0239484867 -2.1481725004 -3.1011621589
C24 3.0313516552 -1.2559484522 -3.8040913581
H25 3.7641416352 -1.9211562411 -4.2798662452
H26 2.5451222168 -0.6865876108 -4.6074344510
H23 0.4600308501 -1.8658516722 -4.6111827302
H24 2.4677648468 -2.9457562153 -2.5101458867
C25 3.7614880760 -0.2660409815 -2.8750904459
H28 4.5355132757 0.2382513992 -3.4607650361
C28 2.7934497521 0.7995831369 -2.3063354653
H29 3.2161963889 1.1808102277 -1.3685079298
H30 2.7361562485 1.6430847161 -3.0044535986
C31 1.3753875751 0.2551913184 -2.0794225931
H33 0.8199876607 0.2376097810 -3.0266302914
O34 1.6069020721 -1.1635160965 -1.7156294043
C37 -0.9442170952 0.5142071870 -1.0510488315
C38 0.5225344014 0.9841781016 -1.0279192620
C39 0.5741161499 2.5086904998 -1.2643505117
H43 -1.0483970221 -0.5615572380 -0.8804238249
H44 1.5812604854 2.9216314762 -1.1418833002
O45 4.4940178865 -0.9467390464 -1.8375629355
H46 3.8729988360 -1.2646344649 -1.1565286940
H40 -1.4116104271 0.7590757975 -2.0152564568
H41 -1.5130755282 1.0251159723 -0.2646397261
H42 -0.0800813848 3.0114794957 -0.5428380675

H45 0.2159026769 2.7654552506 -2.2716837213
H47 0.9566336888 0.7737425478 -0.0388069832
H48 0.9234809922 -1.6354969448 -1.1391626256
C46 -1.0053905699 -3.5135443972 1.8795618801
F47 -0.5092742495 -2.5581184776 2.6935349160
F48 -0.7760800807 -4.7070817333 2.4751221958
F49 -2.3539419139 -3.3402506888 1.8348457845
C50 -0.4097728005 -3.4561146894 0.4361011200
O51 0.1877521691 -2.3994005311 0.1355345312
O52 -0.6459251866 -4.4821766532 -0.2498806481
H53 -0.0635518116 -4.5217989254 -1.6672294624
Gas Phase Energy = -1812.94100035 hartrees
Solution Phase Energy (DCM) = -1812.96467046 hartrees

Lowest energy boat conformation of 2,6-*cis*-*E*-enol 16 complex with TFA
Cartesian Coordinates (Angstroms)
Atom X Y Z
--------- ------------- ------------- -------------
C1 1.4423868347 -5.1234745960 4.9460531081
C2 2.0036191492 -4.7744846013 6.1827642543
C3 1.8504468062 -5.6213475945 7.2828156541
C4 1.1552181234 -6.8382233686 7.1714694673
C5 0.6002818395 -7.1733339016 5.9264598767
C6 0.7383679404 -6.3267830565 4.8213383172
S7 1.7269408854 -4.1039855377 3.4867742391
H8 2.5637852542 -3.8478933751 6.2834384628
H9 2.2896763703 -5.3373219035 8.2386384922
H11 0.0515294325 -8.1077385416 5.8145079358
H12 0.3008285434 -6.6022260394 3.8647919771
C12 1.0419649357 -7.7751113525 8.3531762424
H13 0.1711409305 -8.4333603986 8.2612271105
H14 1.9312331862 -8.4163801399 8.4301303406
H15 0.9576332841 -7.2225601642 9.2957348074
C16 0.6182628030 -2.7236567511 3.6870265146
O17 -0.6653771341 -3.0708693612 3.9827151725
C18 1.0161008577 -1.4567547283 3.4484605234
C21 0.0356268442 -0.3124349606 3.3514644237
C24 0.6965645697 1.0904604013 3.3242977346
H25 1.7778008278 1.0086709142 3.1717189257
H26 0.2979801271 1.6773002290 2.4864516698
H23 2.0693171155 -1.2555298871 3.2830526921
C25 0.4022526772 1.8591034429 4.6154032730
H28 0.8438222148 2.8569617462 4.5678532224
C28 -1.1081855682 1.9757926700 4.8630396579
H29 -1.2620945645 2.2445188577 5.9151333702
H30 -1.5024243638 2.8015426656 4.2578924657
C31 -1.8492325075 0.6620349455 4.5136271410
H33 -2.2766194343 0.7285029584 3.4998163304
O34 -0.8755289595 -0.4153707393 4.4932494129
C37 -3.6314154657 -1.0444705769 5.1819225956
C38 -2.9739794632 0.3080326840 5.4987698840
C39 -4.0352072391 1.4241516413 5.5072538913
H43 -4.4818578846 -1.2183567179 5.8527478643
H44 -4.8272090983 1.1856803256 6.2271694507
O45 1.0590265428 1.2351643850 5.7565970244
H46 0.6615405761 0.3584856028 5.9212403772
H40 -2.9411900909 -1.8835088463 5.3156957981

H41 -4.0081443099 -1.0696101156 4.1501377761
H42 -4.5021392908 1.5270872428 4.5180142324
H45 -3.6172094307 2.3979715110 5.7862355607
H47 -2.5259248837 0.2503337247 6.5023649418
H48 -0.5909602644 -0.4363006111 2.4525699242
H49 -1.1197226757 -2.2440306314 4.2707411626
O46 3.1058529070 1.9249483540 7.2087589141
H50 2.3994368208 1.6771098433 6.5230787252
C48 4.3004632224 2.2656651782 6.7736560034
O49 5.2154193176 2.5870086089 7.4987203453
C50 4.5000655773 2.2257460463 5.2293480666
F51 3.5629672422 2.9764810080 4.6029179493
F52 5.7048241819 2.6855132570 4.8804116132
F53 4.3801447361 0.9585203725 4.7702972233
Gas Phase Energy = -1812.95607592 hartrees
Solution Phase Energy (DCM) = -1812.97686826 hartrees

Lowest energy boat conformation of 2,6-*trans*-*E*-enol 15 complex with TFA
Cartesian Coordinates (Angstroms)
Atom X Y Z
--------- ------------- ------------ -------------
C1 -14.3607043008 -7.2332868879 4.6461394998
C2 -14.9445852251 -6.4064295184 3.6794140507
C3 -16.1550027572 -6.7779106928 3.0809200520
C4 -16.7957633842 -7.9778545516 3.4210118179
C5 -16.1792119486 -8.8106087326 4.3738719800
C6 -14.9839364429 -8.4415824626 4.9927689416
S7 -12.9317928080 -6.6148935765 5.5606433265
H8 -14.4652953629 -5.4711234994 3.3999309506
H9 -16.6030152220 -6.1182898411 2.3387021000
H11 -16.6533852416 -9.7512671868 4.6527366408
H12 -14.5433419843 -9.0851170215 5.7507877552
C12 -18.1272793306 -8.3567386396 2.8119147970
H13 -18.9461340880 -8.1852298997 3.5250773590
H14 -18.3415272891 -7.7648230897 1.9154666633
H15 -18.1554727938 -9.4180867263 2.5372343204
C16 -11.6729015906 -7.8835770148 5.4194842566
O17 -11.1037901773 -8.0868282295 4.1915169826
C18 -11.2450260538 -8.5167024482 6.5291527688
C21 -10.0167504315 -9.3786039275 6.6430212756
C24 -9.1139262357 -8.9222188046 7.8079329736
H25 -8.4950142240 -8.0844959170 7.4665908831
H26 -9.7313165247 -8.5469535447 8.6362661098
H23 -11.8070678511 -8.3404240791 7.4423720068
C25 -8.2257669999 -10.0472693905 8.3583780007
H28 -7.6652967416 -9.6542388767 9.2116648777
C28 -9.0916584410 -11.2474326055 8.8264563218
H29 -8.5565658303 -12.1778606219 8.5943694379
H30 -9.2047926460 -11.2093397020 9.9166513521
C31 -10.4899114460 -11.2611140918 8.1852011199
H33 -11.1490191624 -10.5561211110 8.7123029453
O34 -10.3766873187 -10.7959860110 6.8076896446
C37 -12.5856485664 -12.5619550705 7.5519871451
C38 -11.1787974970 -12.6372004003 8.1686246851
C39 -11.2524407247 -13.2348490836 9.5858143920
H43 -13.0325273185 -13.5624139994 7.4992573709
H44 -11.8006935531 -12.5688019887 10.2662941390

O45 -7.1979307897 -10.4348140461 7.4263011131
H46 -7.5807919252 -10.9242901801 6.6727517105
H40 -12.5708483139 -12.1440660516 6.5406781915
H41 -13.2442870494 -11.9334144987 8.1671070851
H42 -10.2620059945 -13.4203834119 10.0151698853
H45 -11.7851711688 -14.1931186274 9.5585525568
H47 -10.5600158547 -13.3095657851 7.5544064211
H48 -9.4576781929 -9.3590598102 5.7040581598
H49 -11.3352266714 -7.3661412809 3.5738075740
O46 -10.0010994309 -12.4045197437 4.7917457139
H50 -10.1231932863 -11.7791652508 5.5934418950
C48 -8.7363797164 -12.5696224948 4.4894112621
O49 -7.7677422113 -12.0699931278 5.0291289006
C50 -8.5818024905 -13.5483432595 3.2926477253
F51 -7.2984313550 -13.6653232641 2.9269786707
F52 -9.0392894547 -14.7755883598 3.6273870957
F53 -9.2887835161 -13.1210450084 2.2249098940
Gas Phase Energy = -1812.9602753 hartrees
Solution Phase Energy (DCM) = -1812.98026393 hartrees

Lowest energy chair conformation of 2,6-*cis*-*E*-enol 16 complex with TFA
Cartesian Coordinates (Angstroms)
Atom X Y Z
--------- ------------- ------------- -------------
C1 -1.4787655137 -5.7821050224 11.3961065006
C2 -1.0193652110 -4.4825710963 11.6497289563
C3 -0.4344302311 -4.1787485424 12.8818474796
C4 -0.2900221744 -5.1600839742 13.8779499297
C5 -0.7552636673 -6.4582125814 13.6082139330
C6 -1.3392142305 -6.7726635646 12.3796182248
S7 -2.1349608762 -6.2077162912 9.7833548653
H8 -1.1168584737 -3.7141546557 10.8857527300
H9 -0.0823309338 -3.1647906596 13.0673899099
H11 -0.6528971623 -7.2369655983 14.3638897996
H12 -1.6827493426 -7.7850123124 12.1811116419
C12 0.3847792018 -4.8364340498 15.1910702969
H13 0.1847681041 -3.8044010036 15.4982724627
H14 0.0493743681 -5.5049271258 15.9905972988
H15 1.4752059253 -4.9479657236 15.1063007688
C16 -3.9136981269 -6.0356309820 9.9631703457
O17 -4.4869161295 -6.6857719482 8.9046377840
C18 -4.5661510503 -5.3597516067 10.9235738194
C21 -6.0624030507 -5.2356714384 11.0799880267
C24 -6.5997890035 -6.0336137196 12.2858227253
H25 -6.3257803865 -7.0885484767 12.1605943475
H26 -6.1240300950 -5.6649223587 13.2033510679
H23 -3.9809209227 -4.8655693423 11.6929890827
C25 -8.1212450030 -5.8993156292 12.3731164653
H28 -8.3902410935 -4.8598041186 12.6070643198
C28 -8.7656865864 -6.3085799730 11.0473161816
H29 -8.6057001922 -7.3800633984 10.8811427381
H30 -9.8475184112 -6.1291629661 11.0807176660
C31 -8.1525756351 -5.5071137488 9.8860950385
H33 -8.3525011939 -4.4361431980 10.0600929542
O34 -6.7178301091 -5.6726496650 9.8738999475
C37 -8.4069341905 -7.3055205076 8.0373411302
C38 -8.7065786343 -5.8639023683 8.4908090762

C39 -8.2360757923 -4.8527834356 7.4302926746
H43 -7.3335981379 -7.4689684483 7.8808350610
H44 -7.1510943304 -4.9083558089 7.2810780524
O45 -8.6797702984 -6.7366729087 13.4266317940
H46 -8.3803898828 -6.4140949334 14.2995960270
H40 -8.9077518481 -7.5029853008 7.0800512730
H41 -8.7605607474 -8.0559014007 8.7536453539
H42 -8.4858915740 -3.8230013910 7.7191543627
H45 -8.7180815727 -5.0561280446 6.4652192202
H47 -9.7978523756 -5.7610528174 8.5829891712
H48 -6.3108095744 -4.1711519102 11.2294997304
H49 -5.4512911304 -6.4856757413 8.9446340915
O46 -8.6539251010 -9.3408069820 12.8218594135
H50 -8.6739805593 -8.4040517251 13.2040242753
C48 -8.0994225965 -10.2153718618 13.6334991936
O49 -7.7052764826 -10.0417669285 14.7669815262
C50 -7.9667072983 -11.5916850382 12.9223911407
F51 -7.4785779676 -12.5202124757 13.7560554488
F52 -9.1598345798 -12.0253004212 12.4626315472
F53 -7.1259546089 -11.4912569717 11.8670776689
Gas Phase Energy = -1812.96785313 hartrees
Solution Phase Energy (DCM) = -1812.98865542 hartrees

Lowest energy chair conformation of 2,6-*trans*-*E*-enol 15 complex with TFA
Cartesian Coordinates (Angstroms)
Atom X Y Z
--------- ------------- ------------- -------------
C1 6.2257110214 -8.7838665261 -2.6794124532
C2 6.2638750965 -7.4483592294 -3.1045772084
C3 6.7561900831 -6.4586025872 -2.2526963404
C4 7.2517827020 -6.7725784203 -0.9742552183
C5 7.2013991777 -8.1118907175 -0.5623879492
C6 6.6854156333 -9.1110024382 -1.3977236651
S7 5.8230400131 -10.1428883847 -3.8024151002
H8 5.9257872224 -7.1837184778 -4.1027893410
H9 6.7809129383 -5.4252144971 -2.5972782537
H11 7.5690679082 -8.3864433063 0.4256198623
H12 6.6565878380 -10.1425109922 -1.0528372361
C12 7.8483344373 -5.6966715319 -0.0936515820
H13 7.2148555204 -4.8016726771 -0.0654414141
H14 7.9849763519 -6.0504427790 0.9338602536
H15 8.8323168073 -5.3826251427 -0.4703779964
C16 4.2716840469 -9.7221706477 -4.6237896159
O17 3.1206826583 -10.0876968751 -3.9759167799
C18 4.2997182301 -9.1512015313 -5.8431981156
C21 3.2382594662 -8.8696503295 -6.8962425484
C24 1.7861053758 -9.3561813470 -6.7048424359
H25 1.3336537584 -9.3879709876 -7.7057350853
H26 1.7773975643 -10.3755842798 -6.3047054965
H23 5.2903276499 -8.8786476113 -6.1973175876
C25 0.9309469972 -8.4361109288 -5.8307190606
H28 1.1943240996 -8.5783944894 -4.7784249424
C28 1.1126680928 -6.9708289569 -6.2227703233
H29 0.6679069100 -6.8071104081 -7.2143823722
H30 0.5934572108 -6.3220545475 -5.5065612852
C31 2.6078641285 -6.6202549349 -6.2743683367
H33 3.0490823882 -6.8290950342 -5.2854407084

O34 3.2599299798 -7.4638004381 -7.2432040412
C37 2.3507291240 -4.6758339749 -7.9553189688
C38 2.8967502127 -5.1412682637 -6.5953761075
C39 4.3955470705 -4.8210268977 -6.4708573363
H43 2.5640340136 -3.6090669883 -8.0978060461
H44 4.7883960248 -5.1260869611 -5.4930345195
O45 -0.4694278491 -8.8329131405 -5.8959924017
H46 -0.8680441440 -8.5755610665 -6.7515085421
H40 1.2652817472 -4.8051847154 -8.0332595974
H41 2.8197582803 -5.2257821254 -8.7800542115
H42 4.5672750865 -3.7436700851 -6.5770933317
H45 4.9772970722 -5.3350713213 -7.2440545208
H47 2.3712365946 -4.5763805420 -5.8104066879
H48 3.6144474299 -9.3562053246 -7.8052893651
H49 3.3217743886 -10.7066124372 -3.2469914447
O46 -1.6196006721 -8.8996714251 -3.5345030320
H50 -1.3241418648 -8.7631070345 -4.4964347875
C48 -1.5951245578 -7.7899534218 -2.8295724751
O49 -1.3817117594 -6.6597933024 -3.2148945329
C50 -1.8442983232 -8.1015626403 -1.3263343631
F51 -2.0336089521 -6.9743627606 -0.6240057087
F52 -2.9209764439 -8.8935110863 -1.1524274991
F53 -0.7699019086 -8.7413628786 -0.8053017028
Gas Phase Energy = -1812.96133391 hartrees
Solution Phase Energy (DCM) = -1812.97547185 hartrees

Lowest energy conformation of the 2,6-*cis*-THP 9a complex with TFA
Cartesian Coordinates (Angstroms)
Atom X Y Z
--------- ------------- ------------- -------------
C1 0.4938595962 -13.5002673403 -1.3949482645
C2 -0.6639931963 -13.5552138078 -0.6031690758
C3 -1.3391287203 -14.7672254290 -0.4416018308
C4 -0.8731908104 -15.9470833781 -1.0495570127
C5 0.2801157594 -15.8731361556 -1.8466329337
C6 0.9644485209 -14.6657712348 -2.0169204368
S7 1.4859635443 -12.0013551618 -1.4520332550
H8 -1.0259456649 -12.6596714618 -0.1028414265
H9 -2.2336659156 -14.7982023517 0.1804024661
H11 0.6614598991 -16.7726172928 -2.3287677677
H12 1.8719501098 -14.6346671662 -2.6160695837
C12 -1.5781135813 -17.2649423758 -0.8171227297
H13 -2.6626663652 -17.1688544798 -0.9499866858
H14 -1.2147785433 -18.0398026323 -1.5015609496
H15 -1.4098281667 -17.6203490574 0.2091039231
C16 1.0402221137 -11.0608387919 -2.9334237404
O17 1.7498319353 -10.0998353347 -3.1871967779
C18 -0.1528940510 -11.4769260453 -3.7625798312
C21 -0.6643667685 -10.3849632714 -4.7121437610
C24 -1.2254692855 -9.1373273736 -4.0049287435
H25 -2.0188235417 -9.4427022259 -3.3075080416
H26 -0.4358231062 -8.6369017965 -3.4320328357
H23 -0.9628273109 -11.8274957943 -3.1127558197
C25 -1.8064949399 -8.1821470368 -5.0515629832
H28 -0.9997972018 -7.7990715546 -5.6923058004
C28 -2.8428574728 -8.9055845938 -5.9127686524
H29 -3.7088121914 -9.1647408653 -5.2912517213

H30 -3.1904156892 -8.2501464664 -6.7219255146
C31 -2.2390398110 -10.1894006131 -6.5159767154
H33 -1.4171906847 -9.8955595112 -7.1935808415
O34 -1.6876308546 -11.0194281659 -5.4817156458
C37 -4.3768217738 -11.6350923006 -6.4858273002
C38 -3.2340320508 -11.0441627893 -7.3299536152
C39 -2.5028655266 -12.1604666100 -8.0950998921
H43 -4.9707589238 -10.8631280422 -5.9847269154
H44 -3.2007832468 -12.7054077394 -8.7440438015
O45 -2.4501955536 -7.0270696373 -4.4378045755
H40 -3.9876259847 -12.3141270442 -5.7186488094
H41 -5.0580383235 -12.2068488135 -7.1289957174
H42 -2.0548063102 -12.8804204695 -7.4006494698
H45 -1.7033574675 -11.7555876420 -8.7289912278
H47 -3.6728768137 -10.3614462881 -8.0726489580
H46 0.1529958911 -10.0827263839 -5.3868724613
H48 0.1524061438 -12.3470679338 -4.3634220084
H49 -1.7925396182 -6.4933980746 -3.9482014841
C46 -6.8226258761 -7.5693002863 -2.2230246697
F47 -6.8687002034 -8.9233311550 -2.2009473574
F48 -7.2985607472 -7.1230025543 -1.0532489200
F49 -7.6442516866 -7.1435097394 -3.2038015371
C50 -5.3589537781 -7.0928340375 -2.4479556709
O51 -4.7098936129 -6.5997787217 -1.5513899901
O52 -4.9829665643 -7.3415999365 -3.6837705124
H53 -4.0059175692 -7.1359371783 -3.8521111053
Gas Phase Energy = -1812.99886544 hartrees
Solution Phase Energy (DCM) = -1813.01999821 hartrees

Lowest energy conformation of 2,6-*trans*-THP 8a complex with TFA
Cartesian Coordinates (Angstroms)
Atom X Y Z
--------- ------------- ------------- -------------
C1 -11.9961935224 1.1151461969 -2.6958315596
C2 -10.6526845757 0.8796790228 -2.3631928530
C3 -9.6456026422 1.1762216996 -3.2865872333
C4 -9.9497576175 1.7017836253 -4.5551167862
C5 -11.2977921485 1.9391090243 -4.8685422035
C6 -12.3151776770 1.6432766903 -3.9555612105
S7 -13.3130585507 0.5515753828 -1.6068001591
H8 -10.3962311047 0.4490110248 -1.3976812653
H9 -8.6072336658 0.9790617493 -3.0220068646
H11 -11.5604391454 2.3431776626 -5.8457064011
H12 -13.3549033538 1.8062640042 -4.2294728527
C12 -8.8552103754 1.9669365918 -5.5649366377
H13 -8.4508972665 1.0243255627 -5.9592457026
H14 -9.2277594501 2.5492015658 -6.4150320238
H15 -8.0182281029 2.5134347508 -5.1151689982
C16 -13.9012010432 1.9465562042 -0.6096123036
O17 -14.8921185643 1.7326034094 0.0674127322
C18 -13.1424267651 3.2537067100 -0.6357380972
C21 -13.8345452759 4.4079656989 0.1303530789
C24 -13.8280677395 4.3118198693 1.6706138886
H25 -14.5731914703 5.0260929597 2.0485786582
H26 -14.1392301238 3.3117908689 1.9896149795
H23 -13.0294668883 3.5644082437 -1.6820332181
C25 -12.4631463328 4.6623476569 2.2655426104
H28 -11.7450149758 3.8639865231 2.0493185400

C28 -11.9334359669 5.9819174206 1.7019385462
H29 -12.5671656481 6.8165053594 2.0399540001
H30 -10.9181438120 6.1543056948 2.0768148309
C31 -11.9409003252 5.9379680570 0.1638008932
H33 -11.2744164671 5.1239041819 -0.1696936320
O34 -13.2803773375 5.6637805418 -0.2976267981
C37 -11.5163703182 7.0846074236 -2.0592942989
C38 -11.4698604752 7.2305294693 -0.5281729613
C39 -10.0517449697 7.6290910837 -0.0808827166
H43 -12.5197854648 6.8280053237 -2.4128277755
H44 -10.0000413824 7.8855891374 0.9829639376
O45 -12.5092960718 4.6711091754 3.7178258936
H40 -10.8263257496 6.2979650347 -2.3966544138
H41 -11.2121692471 8.0215207580 -2.5411548711
H42 -9.7135358835 8.5055131782 -0.6466634679
H45 -9.3349766663 6.8172528285 -0.2709929440
H47 -12.1668613341 8.0300771444 -0.2345248521
H46 -14.8746192349 4.4446892105 -0.2067680594
H48 -12.1275776701 3.0447388527 -0.2723342163
H49 -13.0135634961 5.4367367579 4.0610144635
C46 -8.5650196535 2.1518552612 5.0602106058
F47 -8.8769198995 1.7193879993 6.2995972629
F48 -7.9973555125 1.1367269785 4.3927525109
F49 -7.6458855557 3.1378846125 5.1851752930
C50 -9.8164575888 2.6834436326 4.3057351812
O51 -10.0865910724 2.3033005567 3.1865265715
O52 -10.4563932994 3.5739342641 5.0362691549
H53 -11.2528917507 3.9673064206 4.5480544524
Gas Phase Energy = -1812.99537168 hartrees
Solution Phase Energy (DCM) = -1813.01575231 hartrees

Solvent: $CDCl_3$
Frequency: 400 Mhz

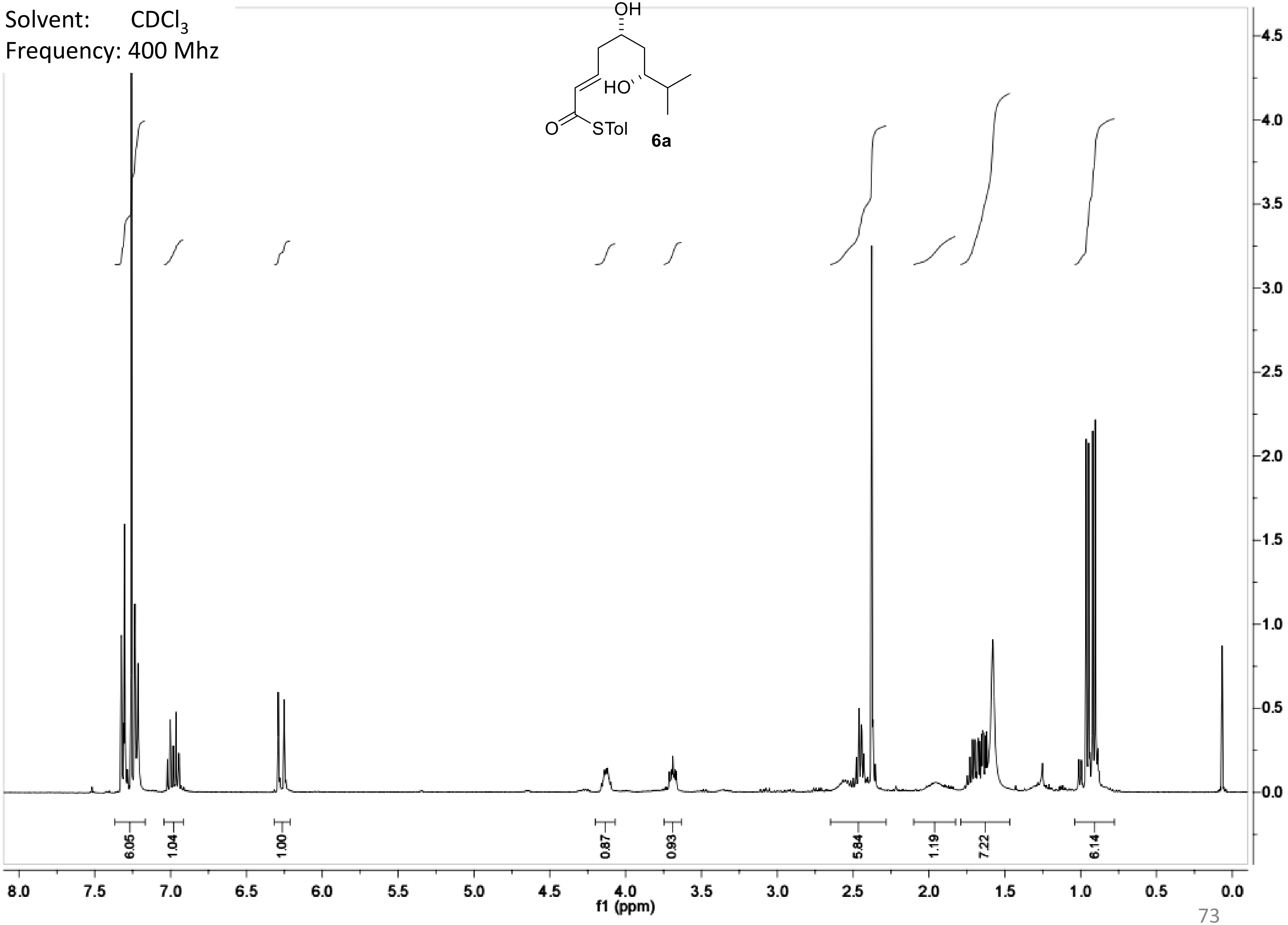

Solvent: $CDCl_3$
Frequency: 101 Mhz

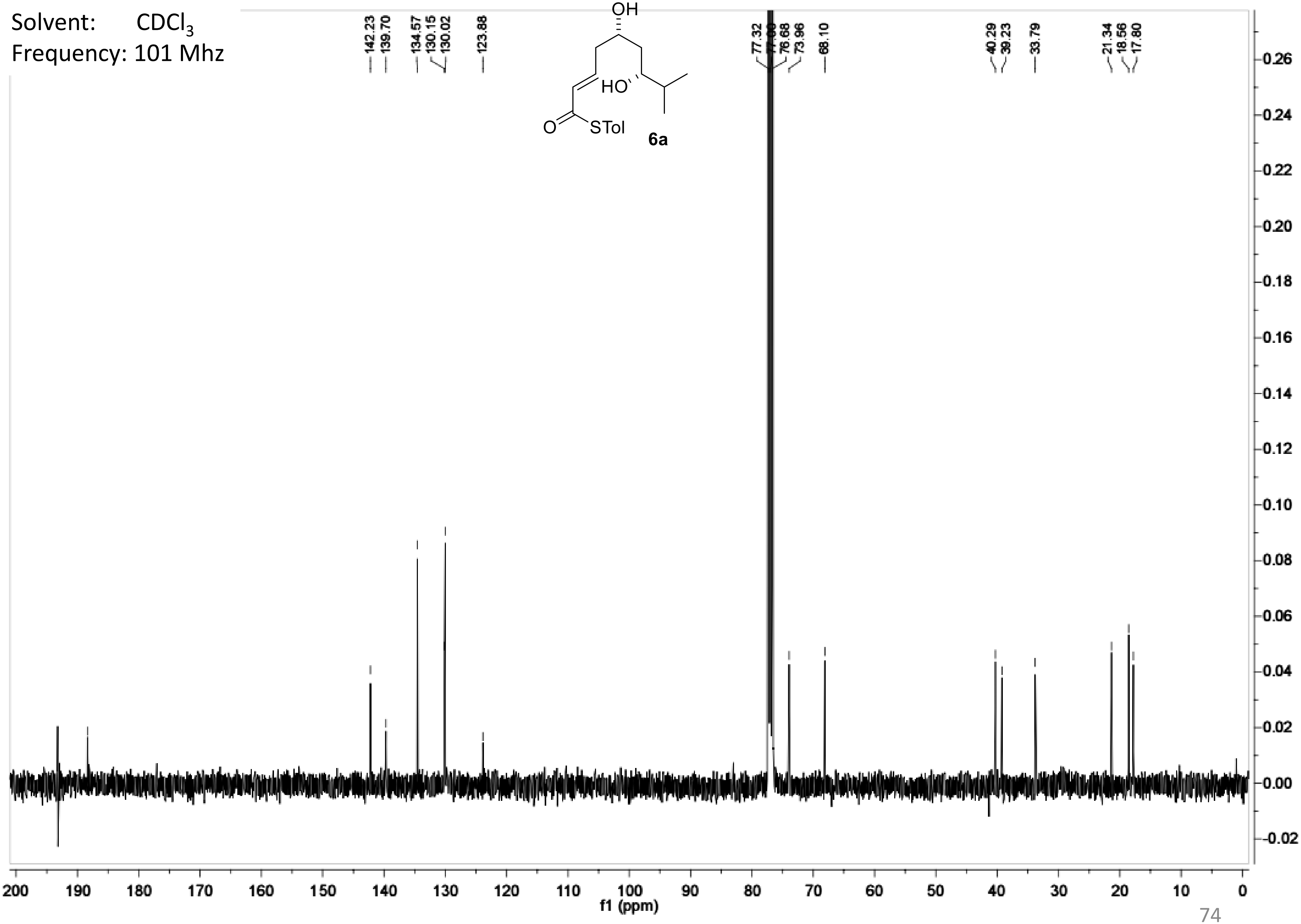

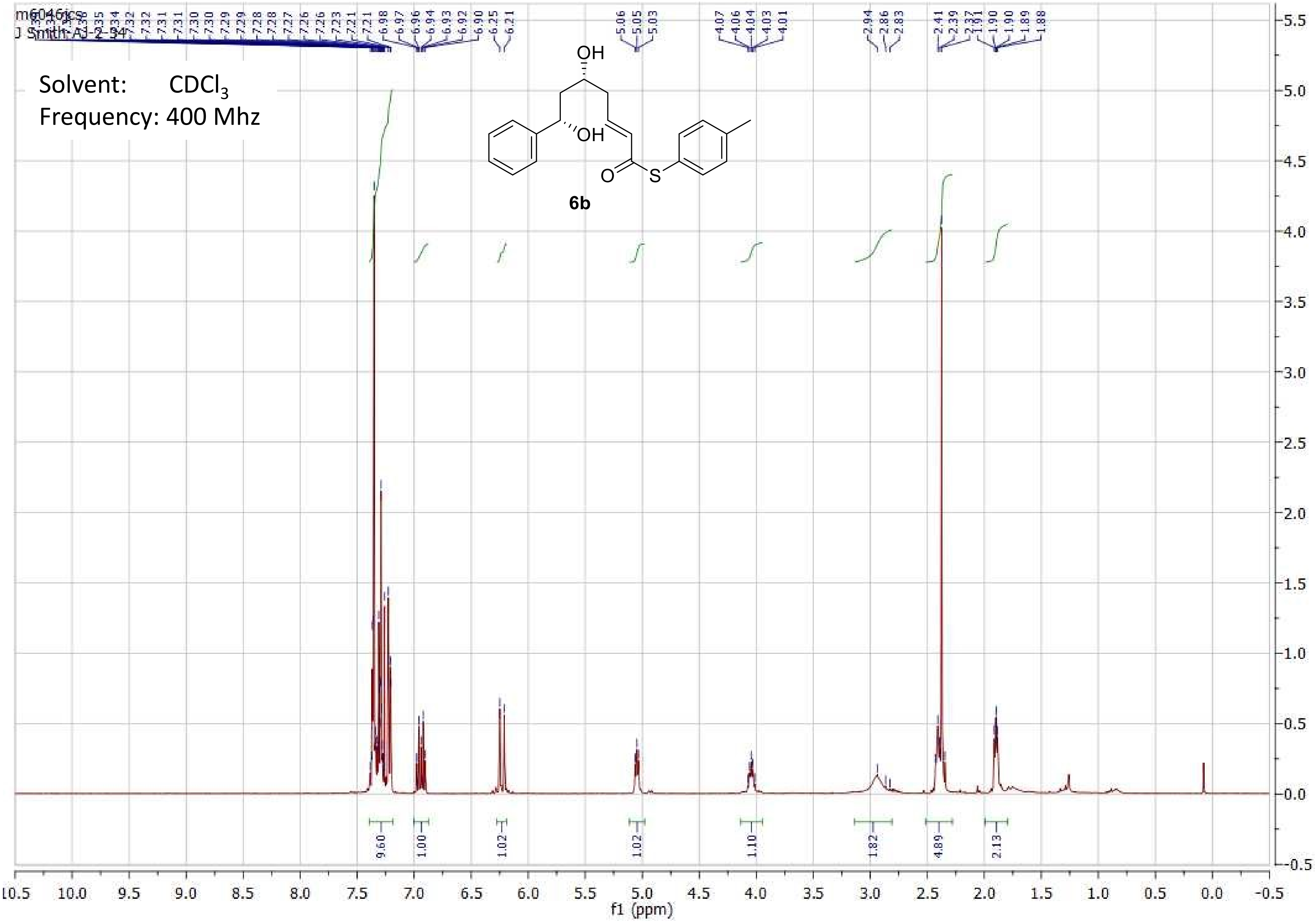

m6046jcs
J Smith-AJ-2-34
Solvent: CDCl3
Frequency: 400 Mhz
OH
OH
O
S
6b
9.60
1.00
1.02
1.02
1.10
1.82
4.89
2.13
f1 (ppm)

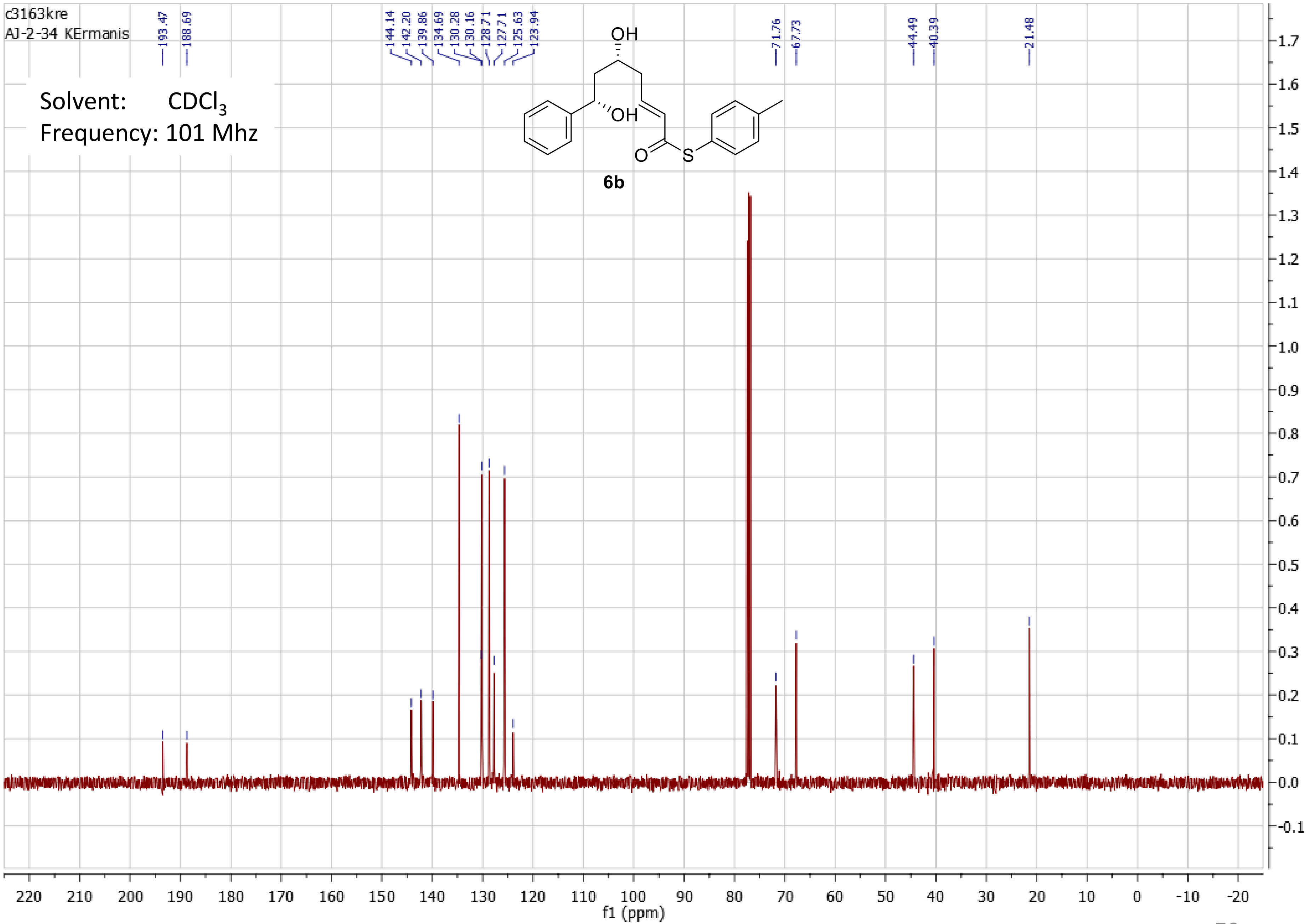
c3163kre
AJ-2-34 KErmanis
Solvent: CDCl3
Frequency: 101 Mhz
193.47
188.69
144.14
142.20
139.86
134.69
130.28
130.16
128.71
127.71
125.63
123.94
71.76
67.73
44.49
40.39
21.48
OH
OH
O
S
6b
f1 (ppm)

Solvent: $CDCl_3$
Frequency: 400 Mhz

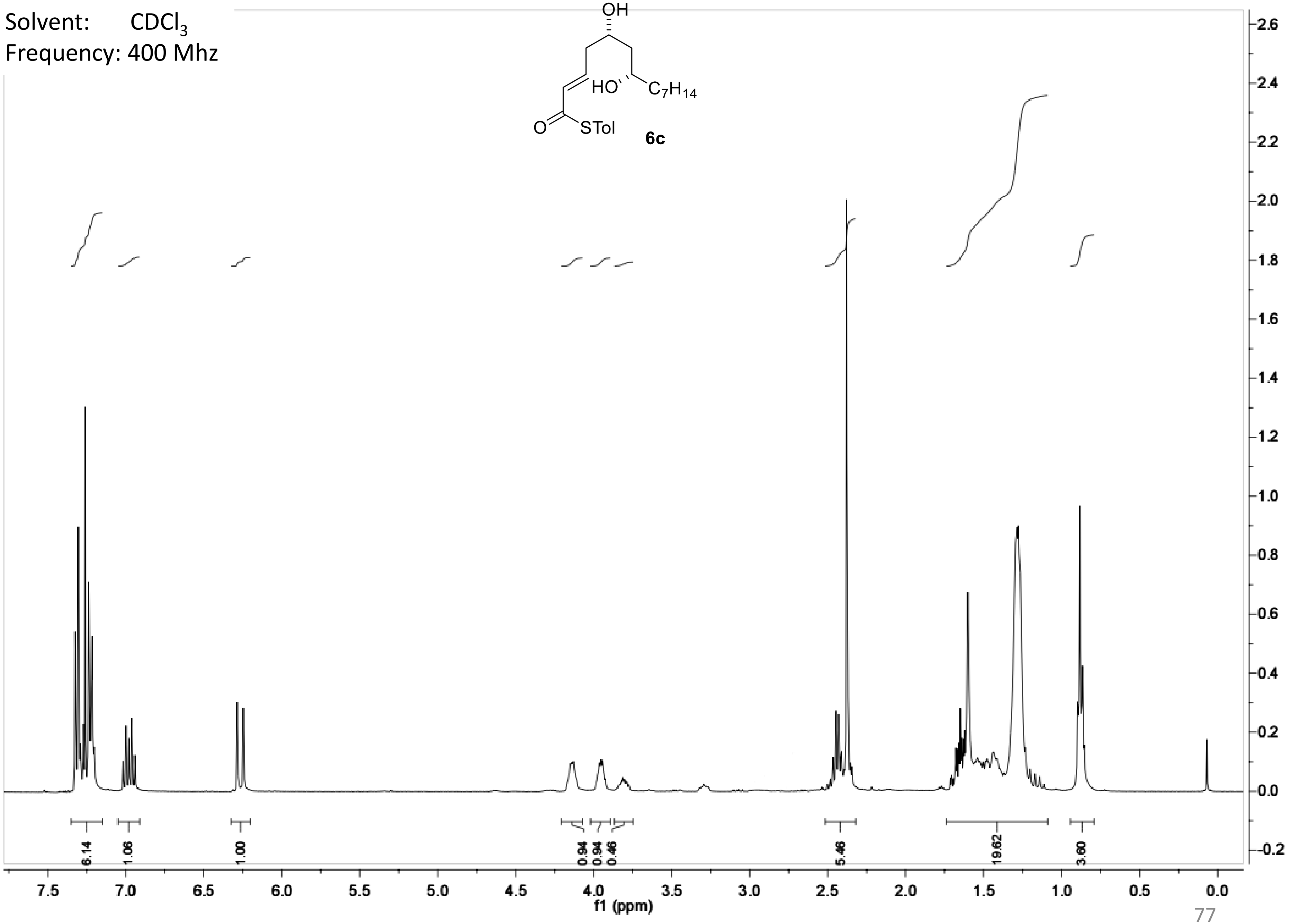

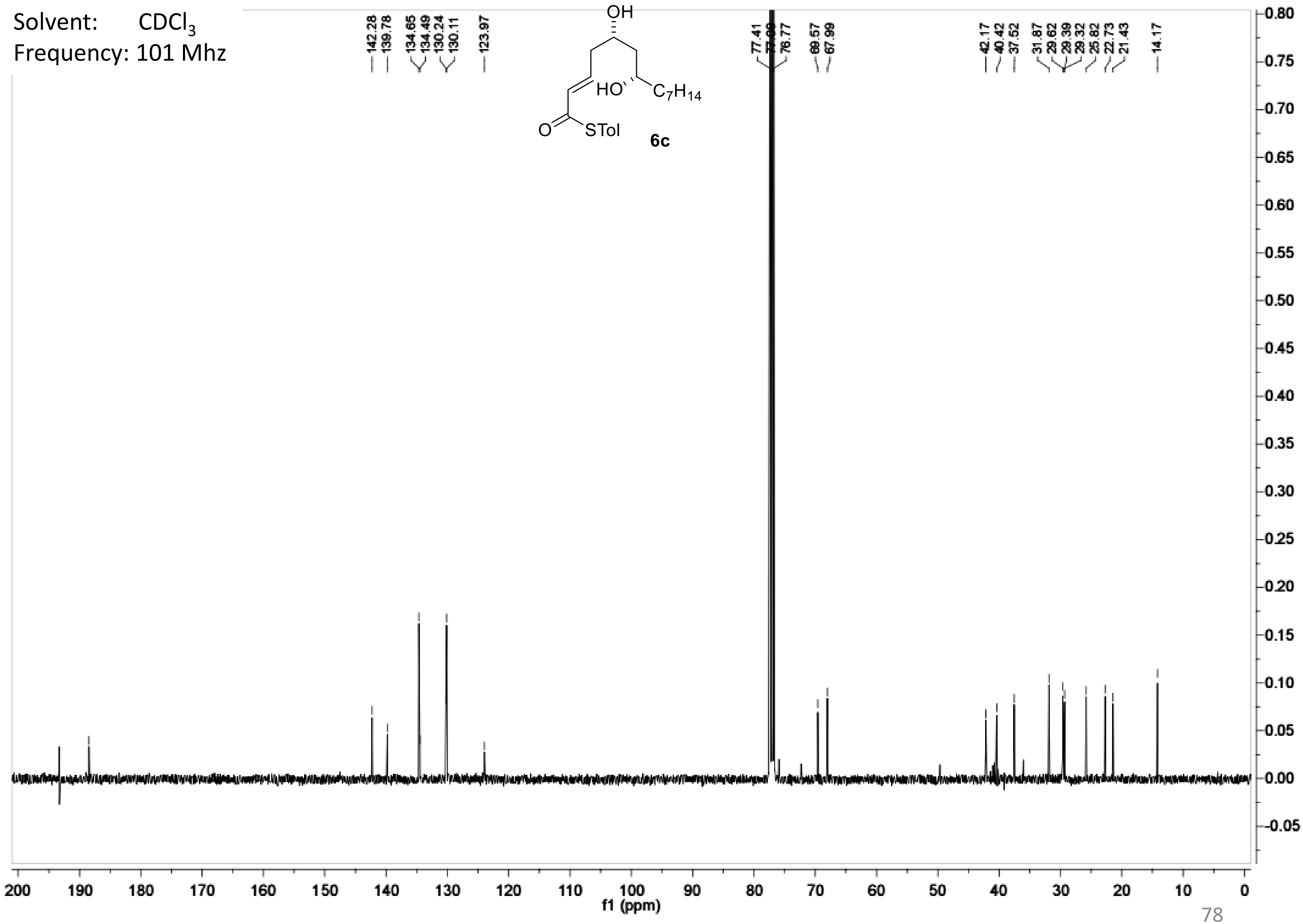
Solvent: CDCl$_3$
Frequency: 101 Mhz
142.28
139.78
134.65
134.49
130.24
130.11
123.97
OH
HO
C_7H_{14}
O
STol
6c
77.41
76.77
69.57
67.99
42.17
40.42
37.52
31.87
29.62
29.39
29.32
25.82
22.73
21.43
14.17
f1 (ppm)

Solvent: $CDCl_3$
Frequency: 400 Mhz

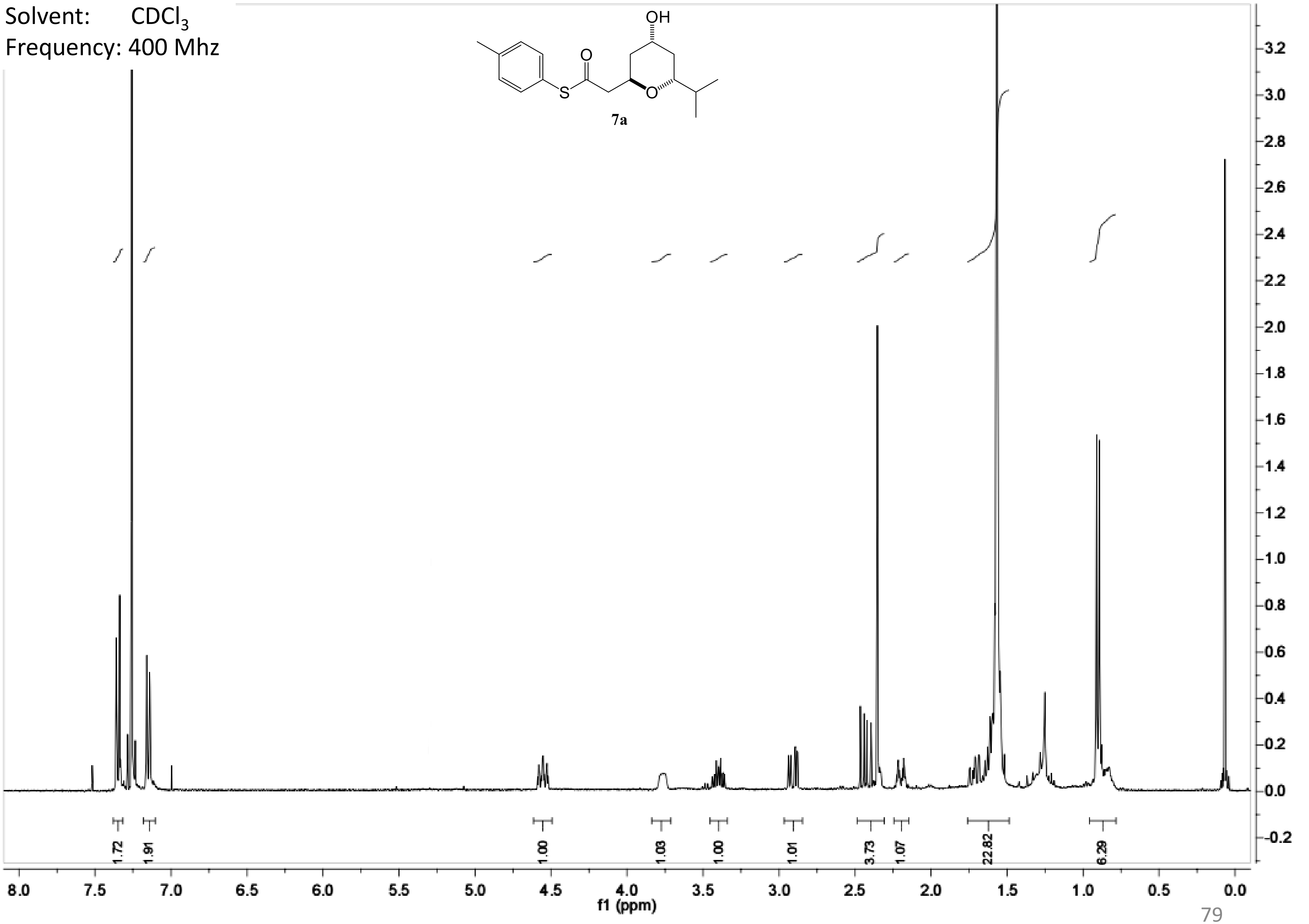

Solvent: $CDCl_3$
Frequency: 125 Mhz

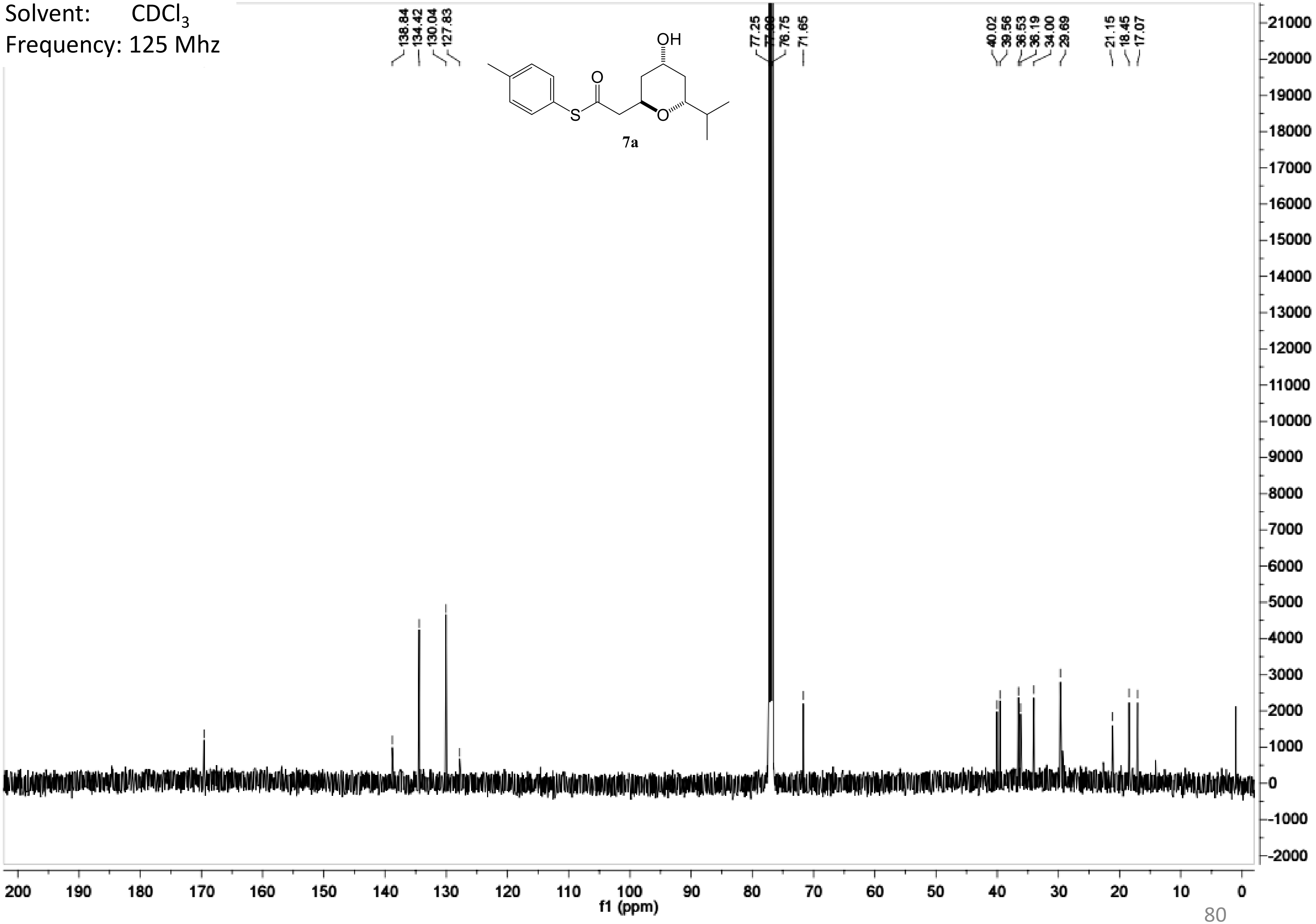

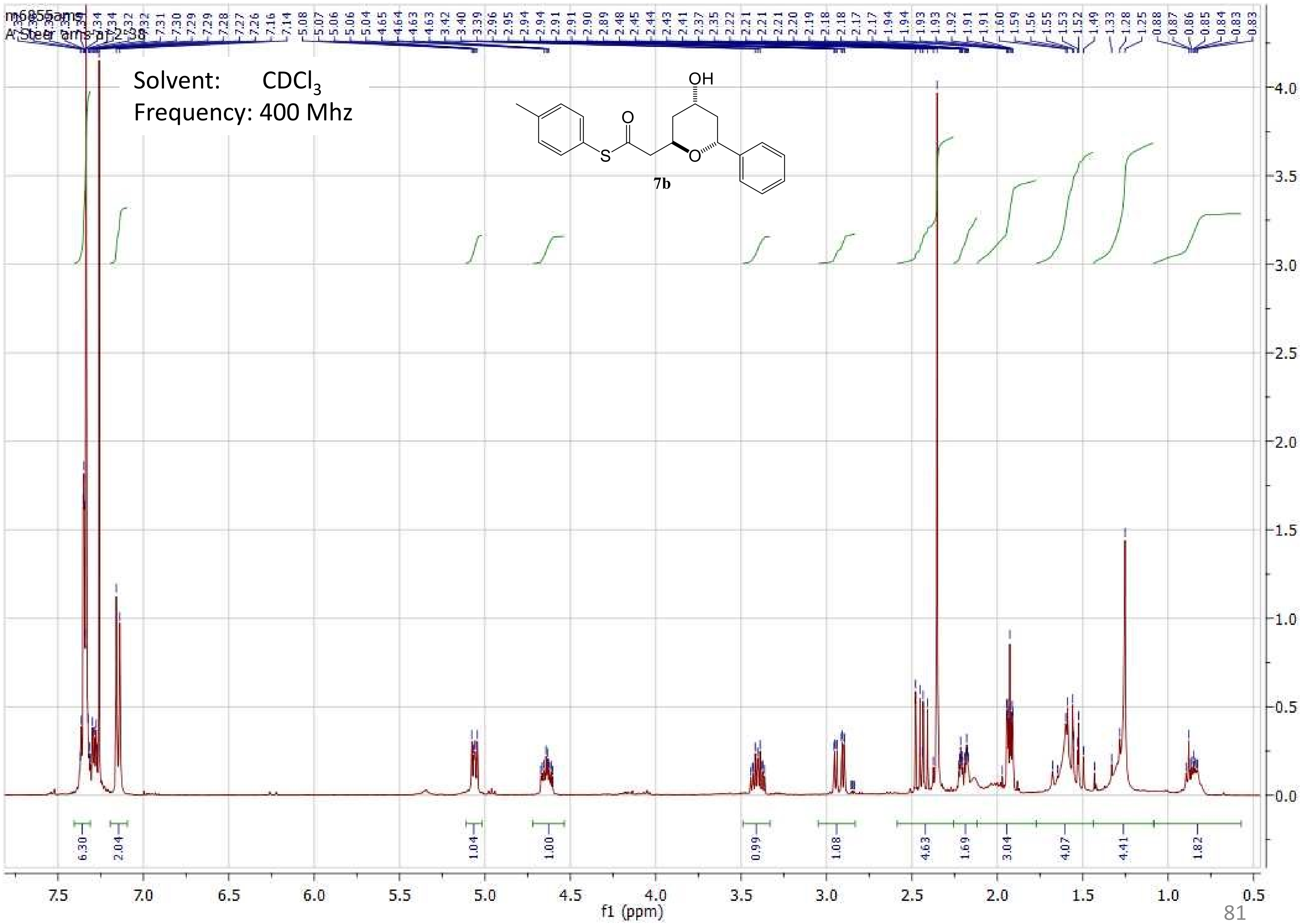
Solvent: $CDCl_3$
Frequency: 400 Mhz
7b
OH
O
S
f1 (ppm)

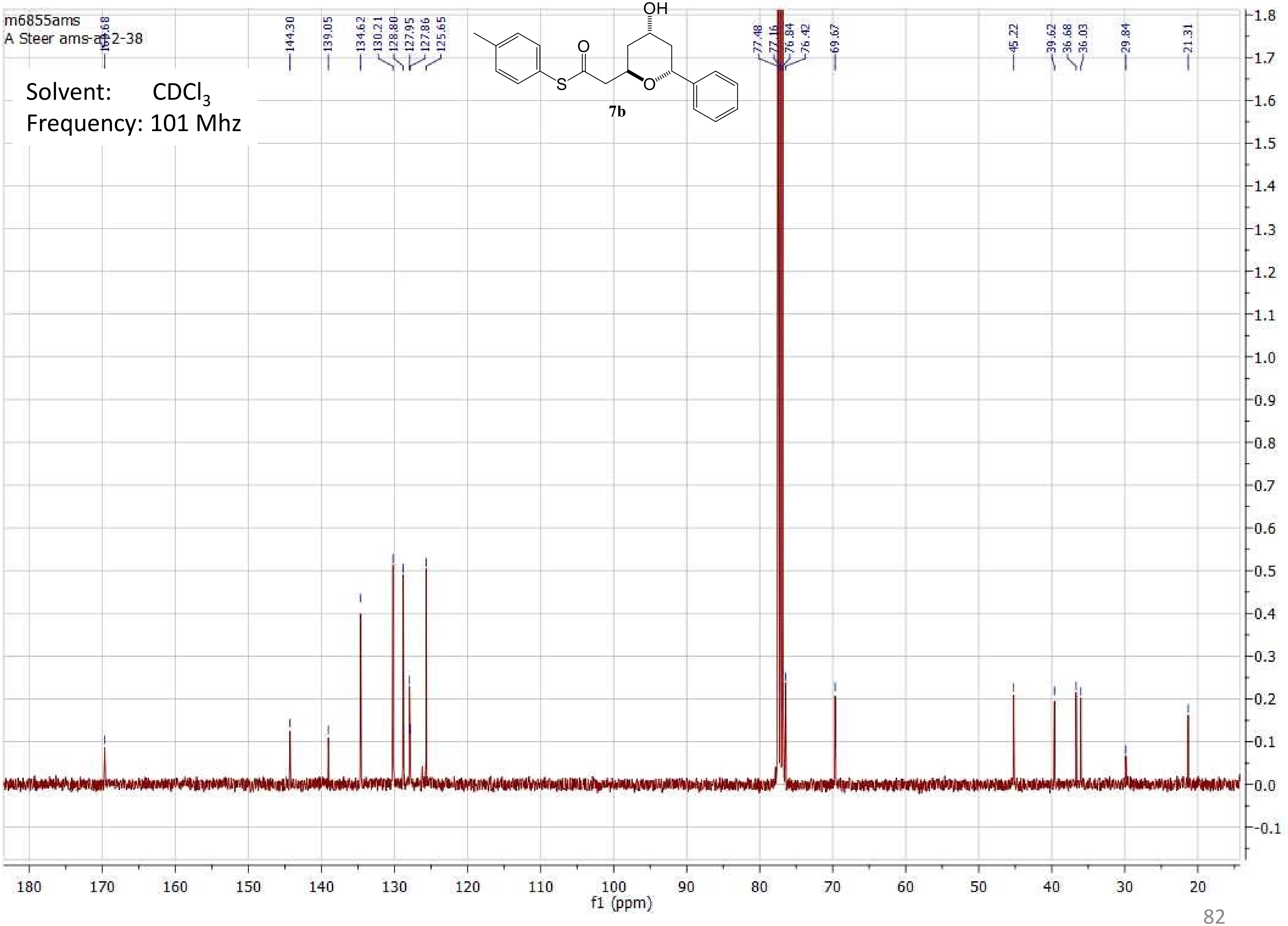

m6855ams
Solvent: $CDCl_3$
Frequency: 101 Mhz
144.30
139.05
134.62
130.21
128.80
127.95
127.86
125.65
OH
O
S
7b
77.48
77.16
76.84
76.42
69.67
45.22
39.62
36.68
36.03
29.84
21.31
f1 (ppm)

Solvent: $CDCl_3$
Frequency: 400 Mhz

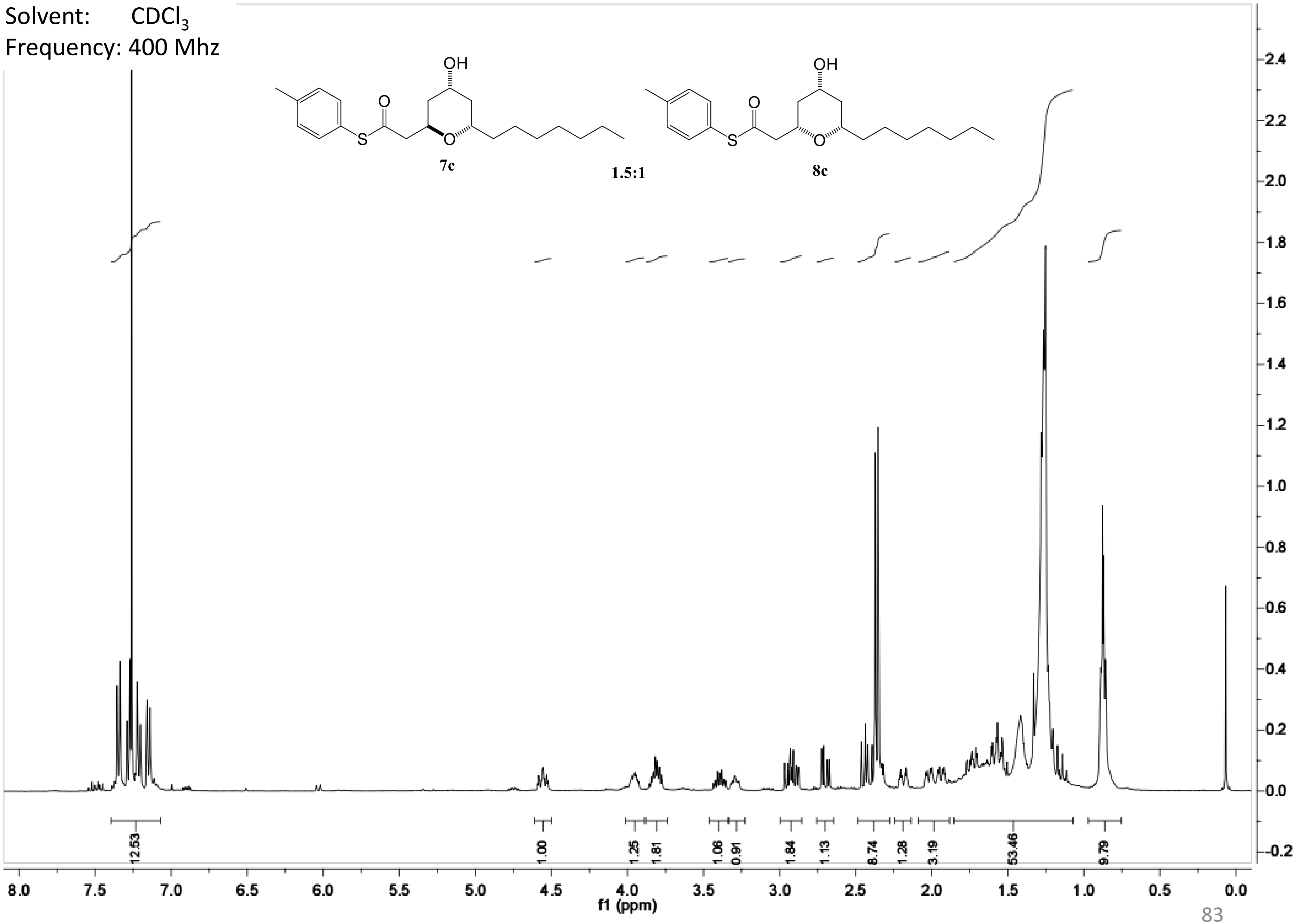

Solvent: $CDCl_3$
Frequency: 125 Mhz

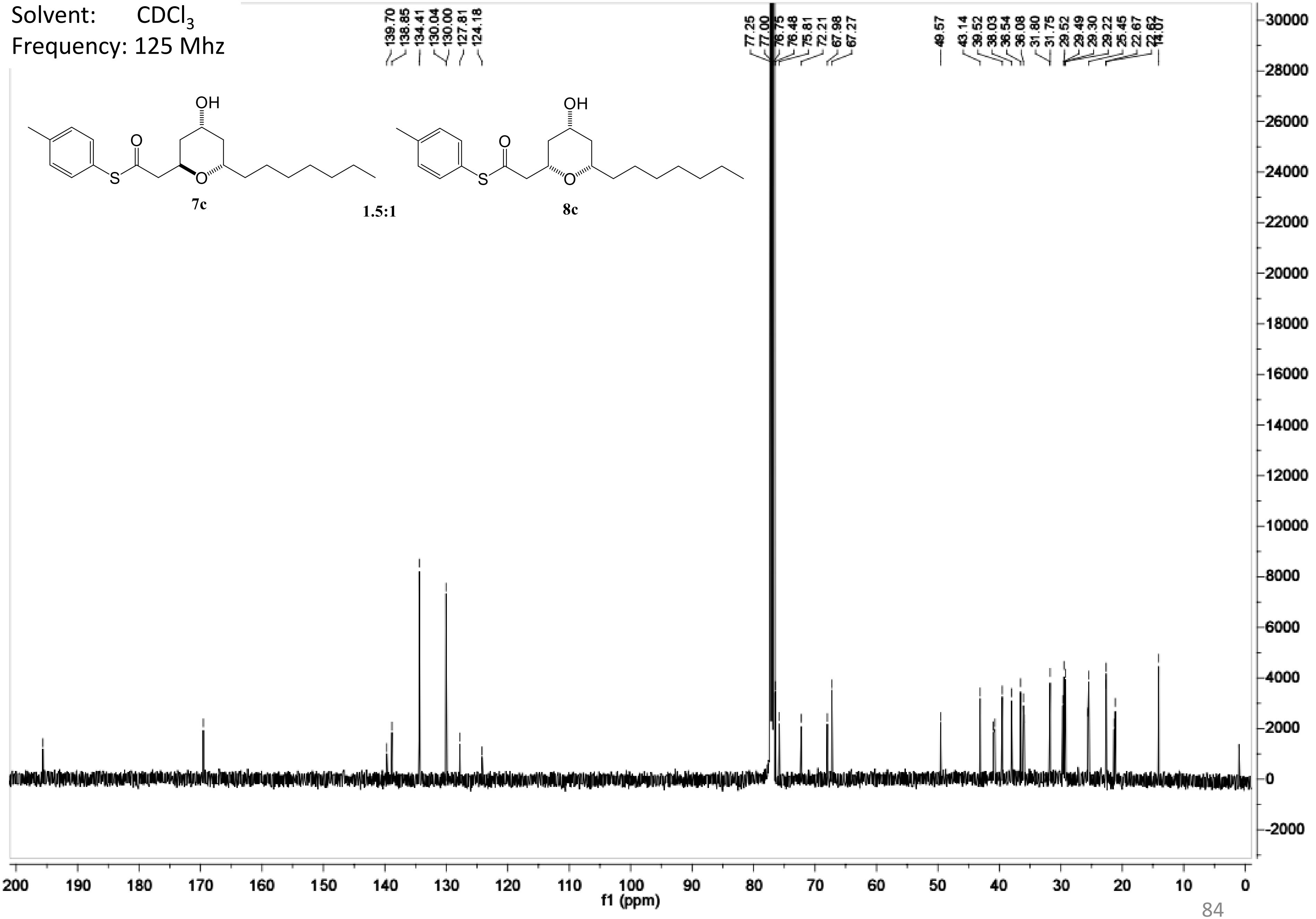

Solvent: $CDCl_3$
Frequency: 400 Mhz

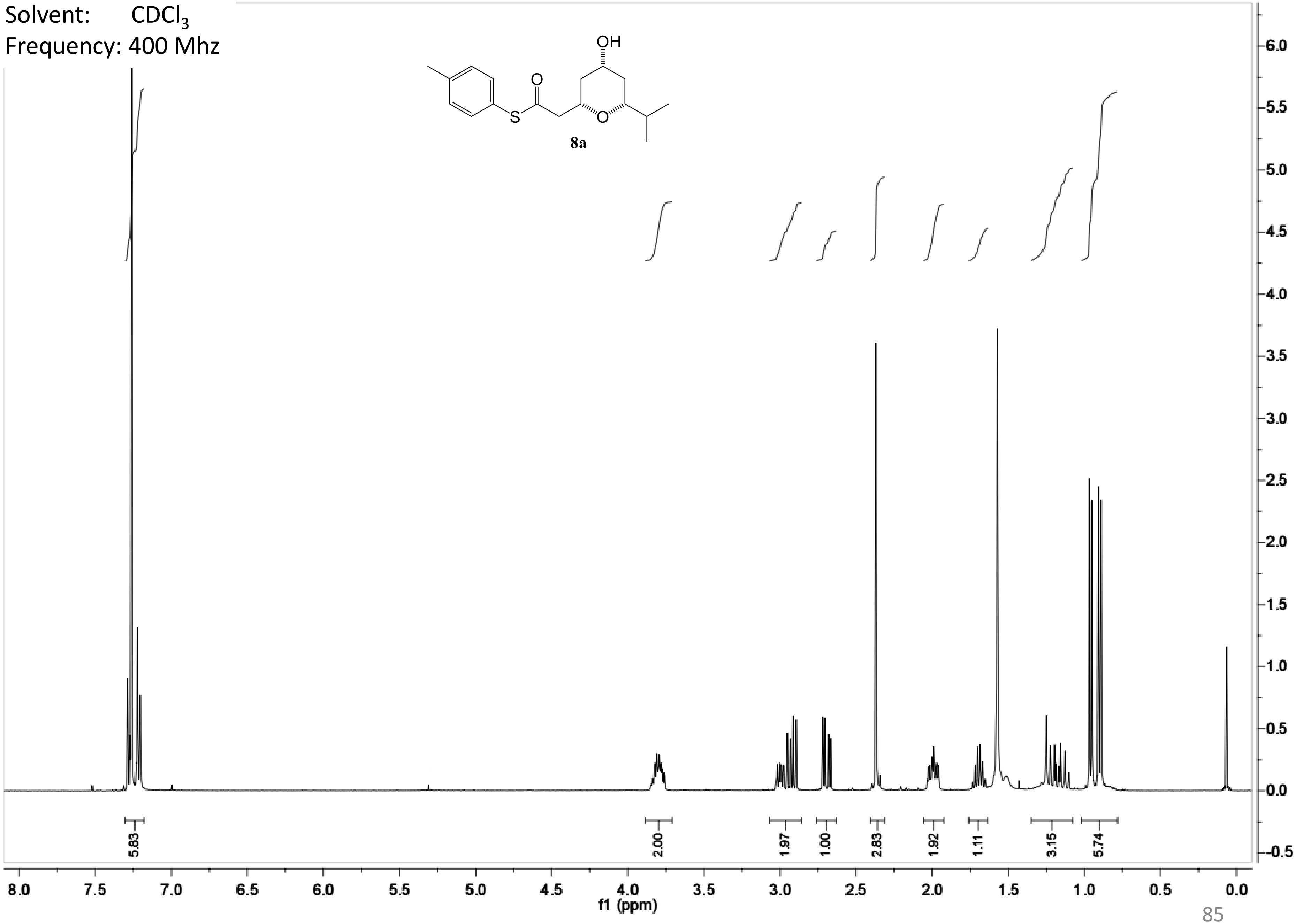

Solvent: $CDCl_3$
Frequency: 101 Mhz

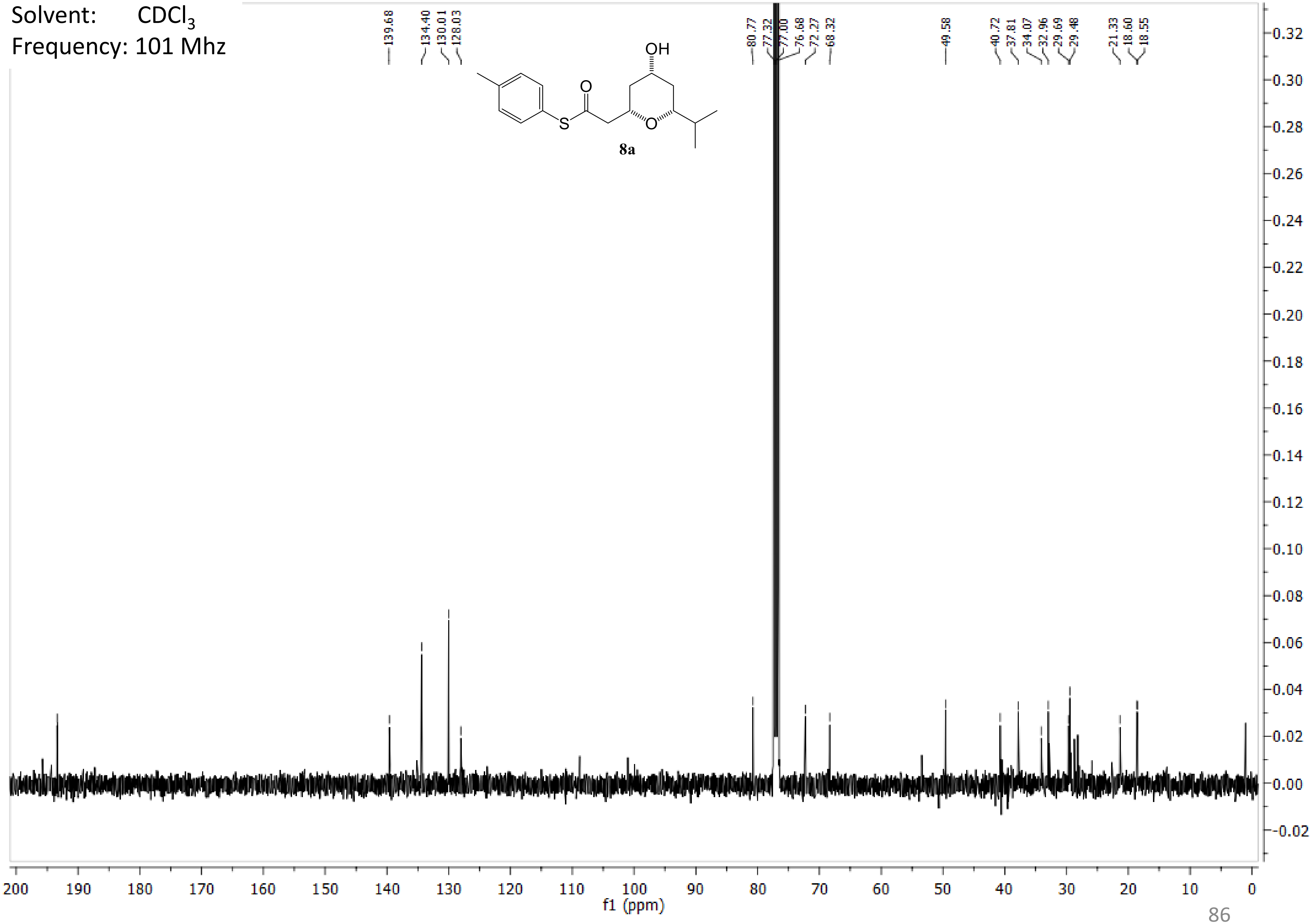

Solvent: $CDCl_3$
Frequency: 400 Mhz

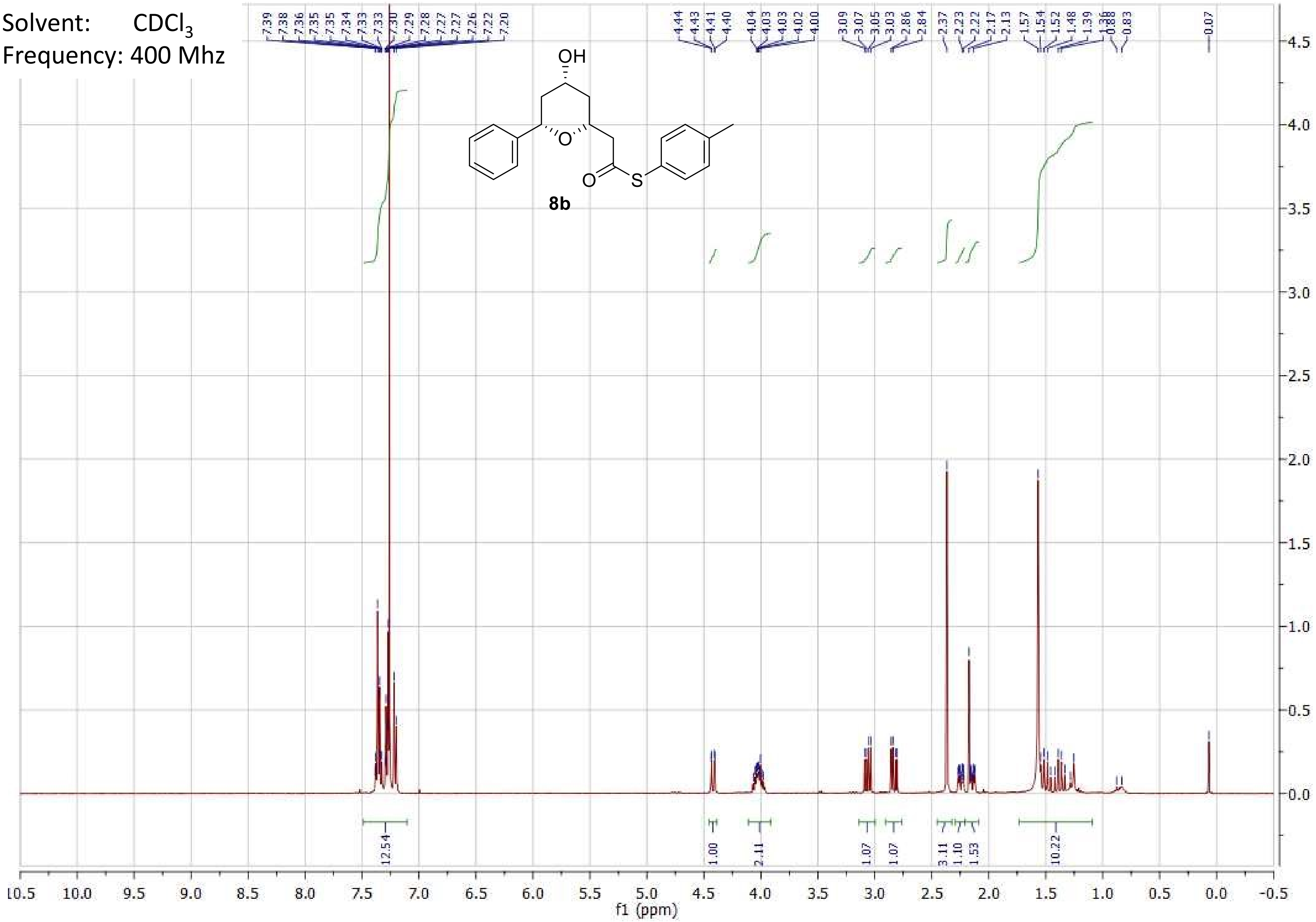

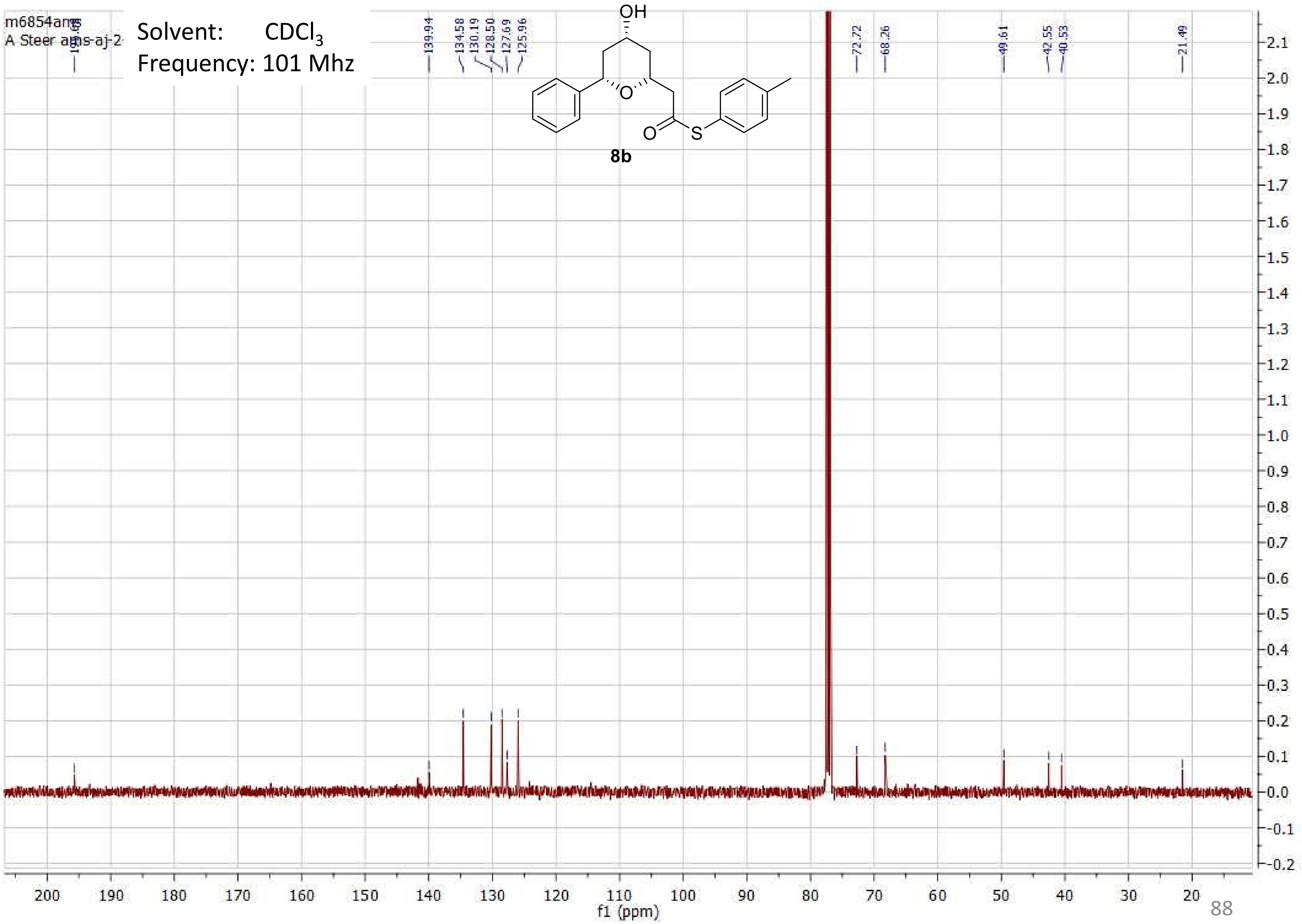
A Steer ams-aj-2
Solvent: CDCl$_3$
Frequency: 101 Mhz
139.94
134.58
130.19
128.50
127.69
125.96
72.72
68.26
49.61
42.55
40.53
21.49
OH
O
O
S
8b
f1 (ppm)

Solvent: $CDCl_3$
Frequency: 400 Mhz

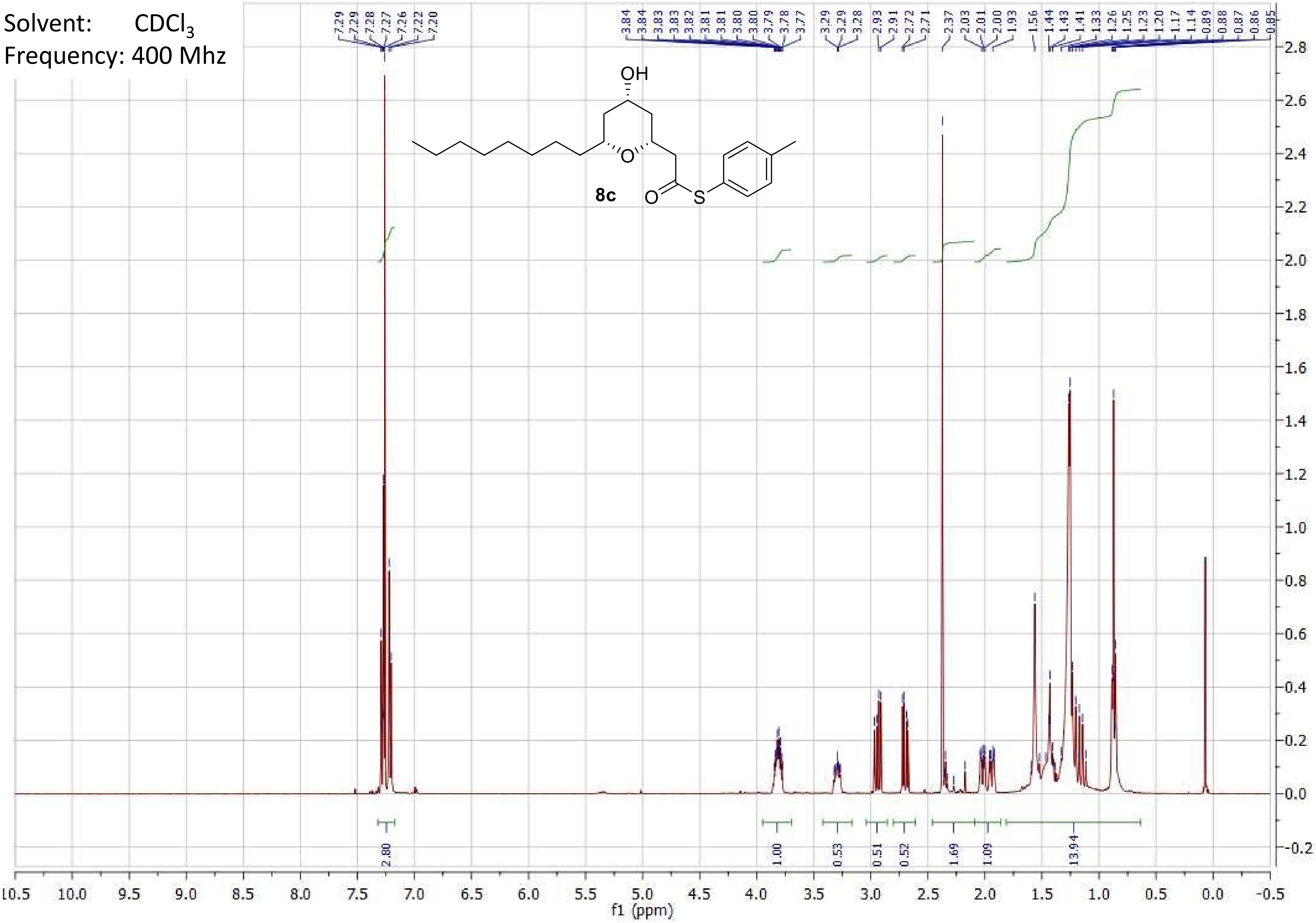

Solvent: $CDCl_3$
Frequency: 101 Mhz

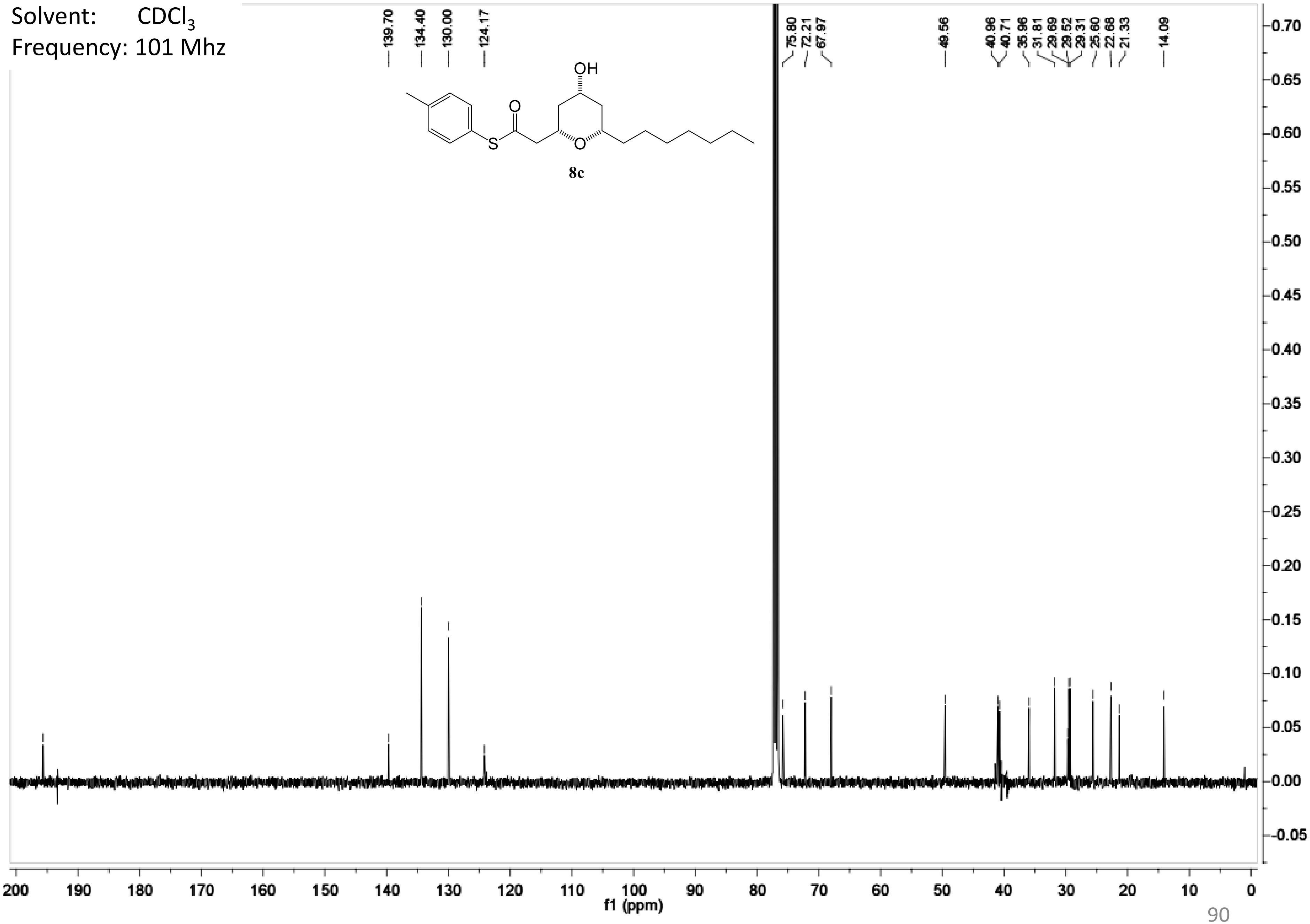

Solvent: $CDCl_3$
Frequency: 400 Mhz

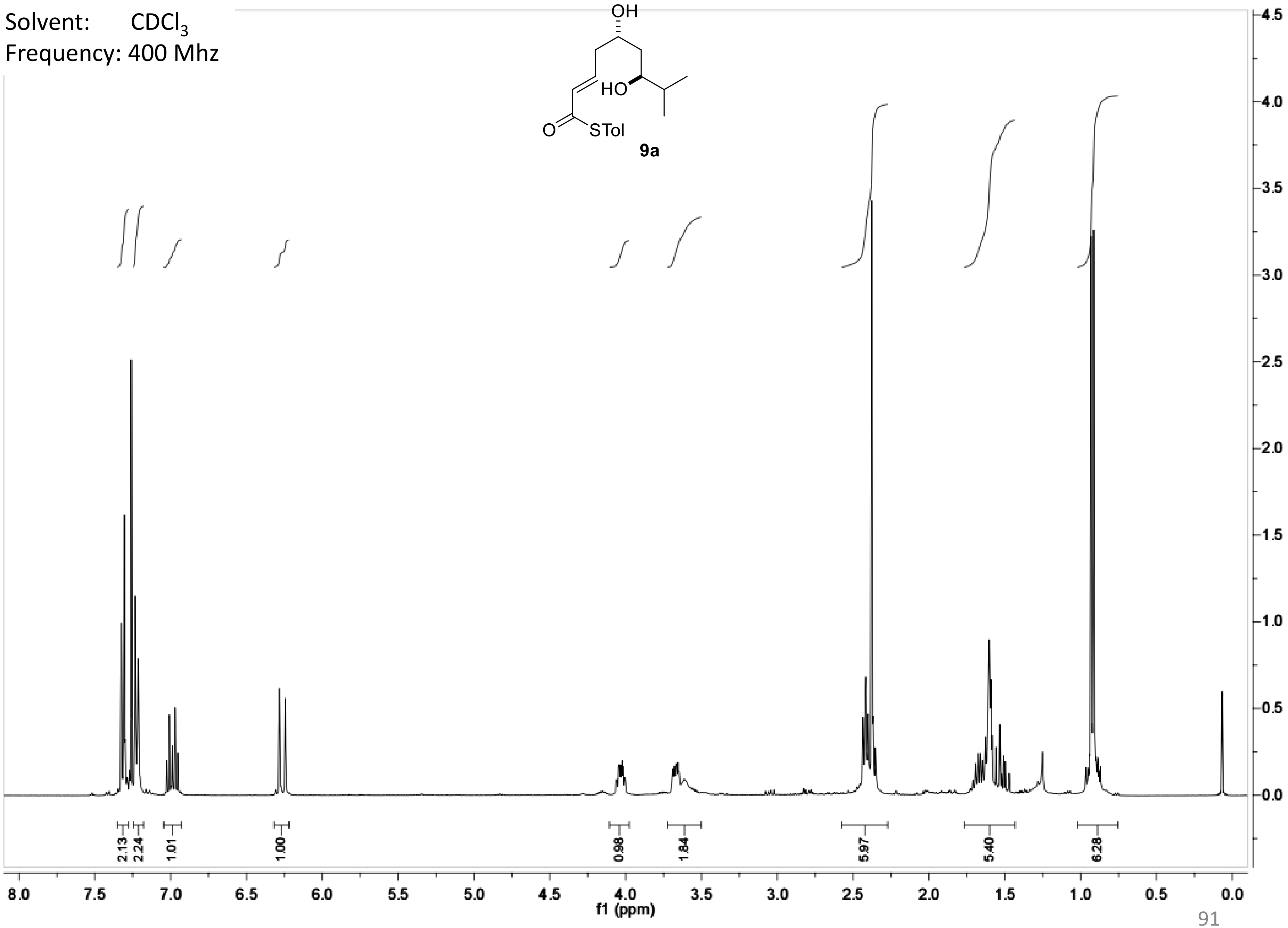

Solvent: $CDCl_3$
Frequency: 101 Mhz

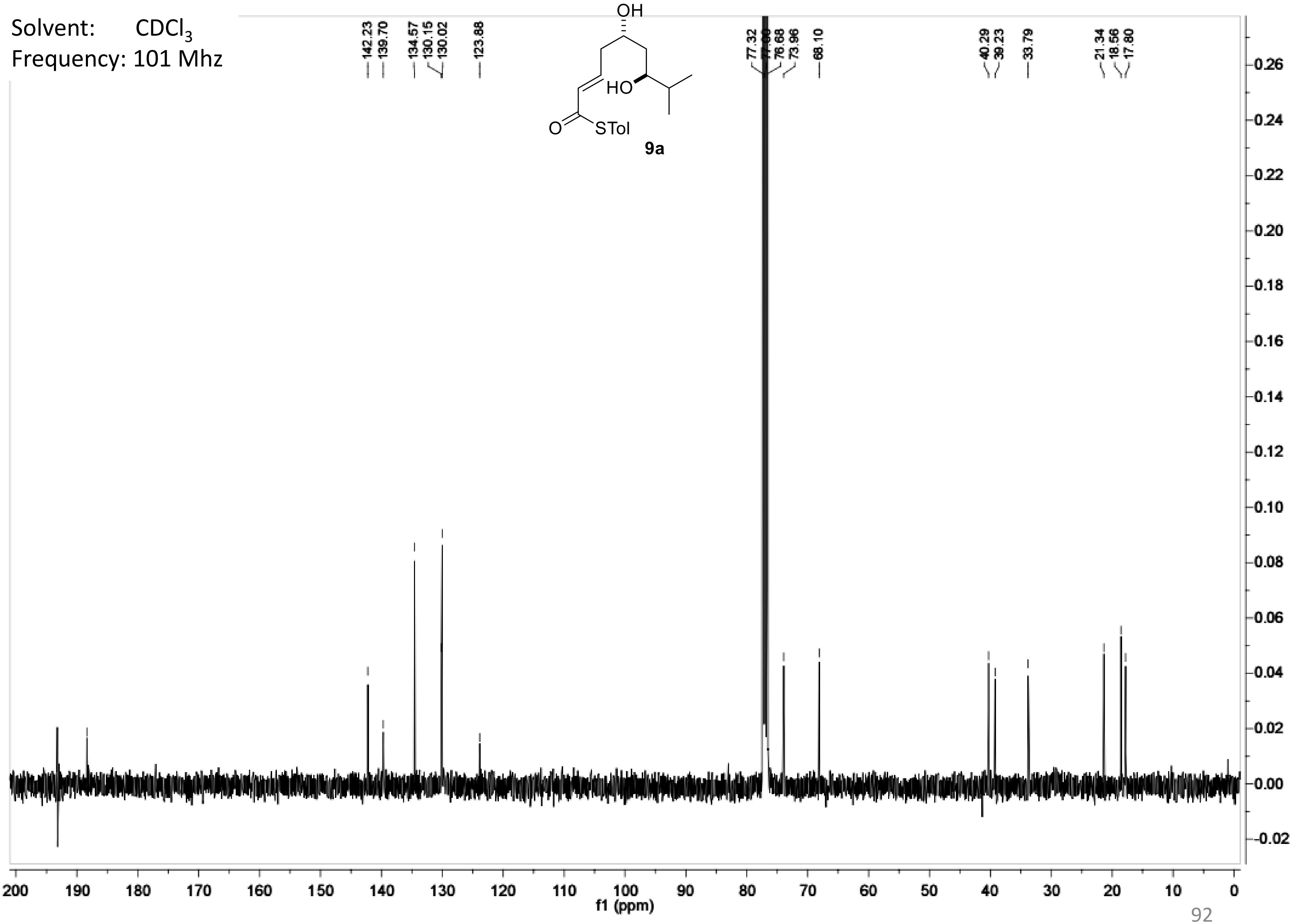

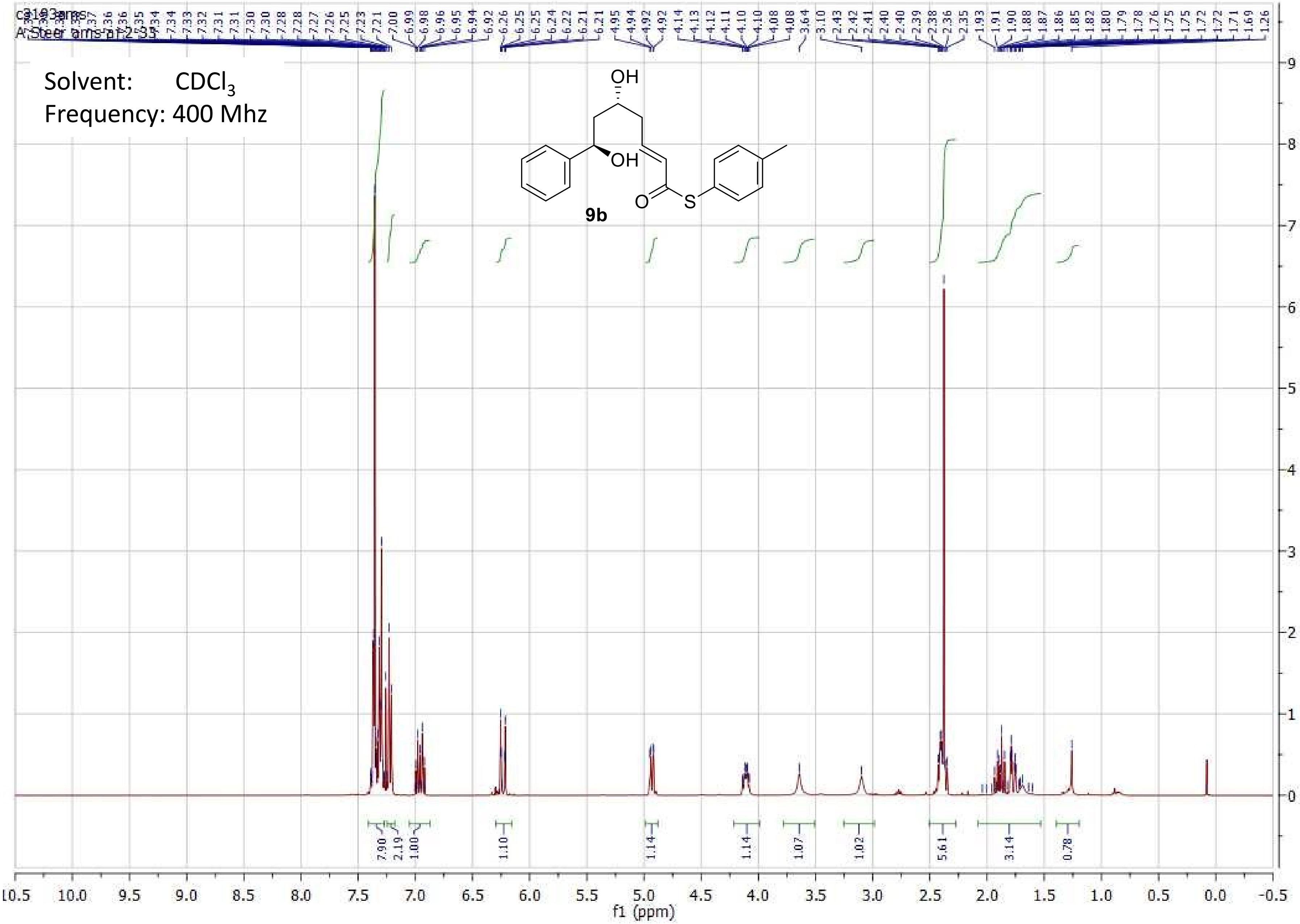
Solvent: CDCl3
Frequency: 400 Mhz
OH
OH
O
S
9b
f1 (ppm)

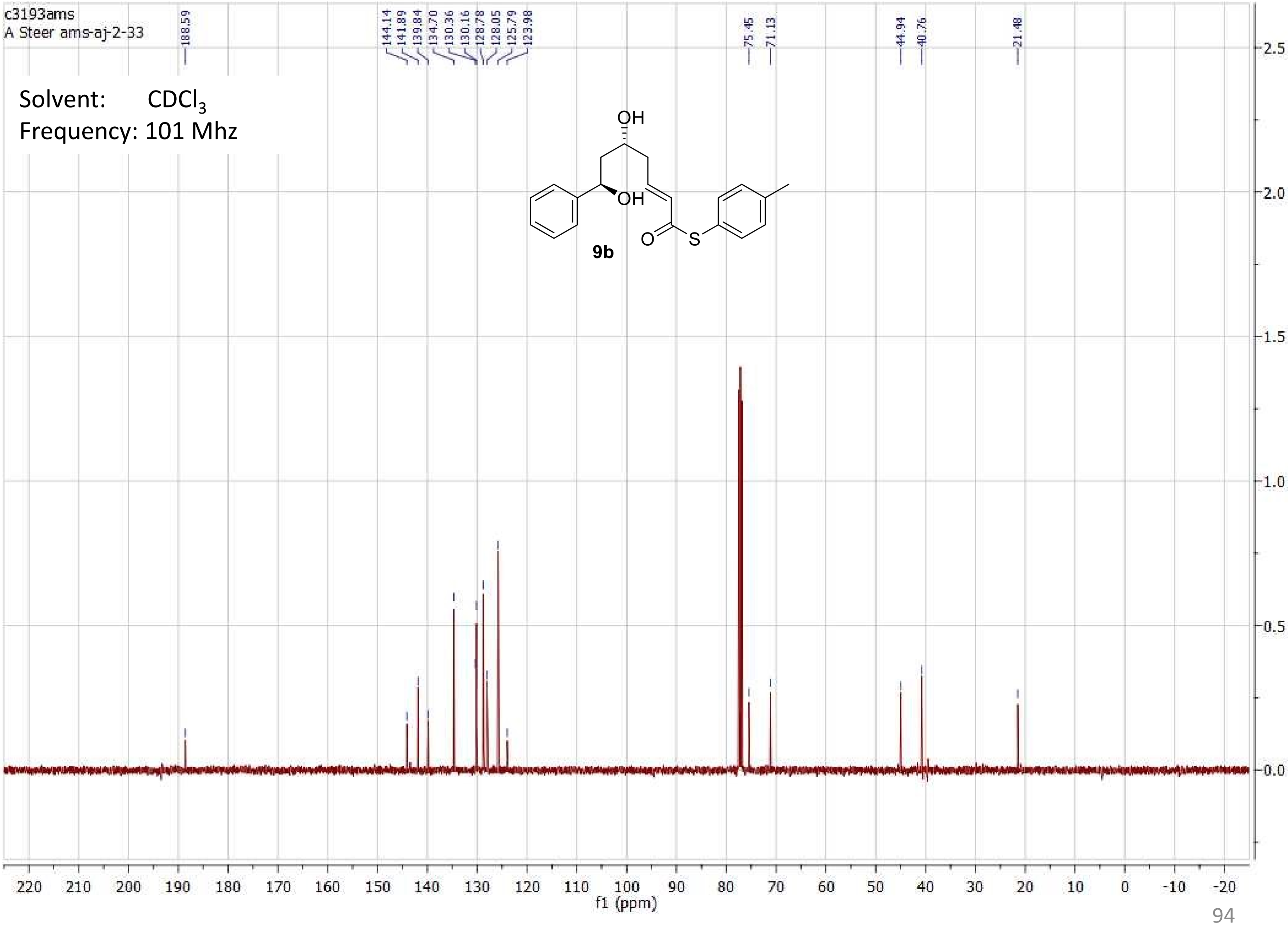
c3193ams
A Steer ams-aj-2-33
188.59
144.14
141.89
139.84
134.70
130.36
130.16
128.78
128.05
125.79
123.98
75.45
71.13
44.94
40.76
21.48
Solvent: CDCl3
Frequency: 101 Mhz
OH
OH
O
S
9b
f1 (ppm)
220 210 200 190 180 170 160 150 140 130 120 110 100 90 80 70 60 50 40 30 20 10 0 -10 -20
2.5 2.0 1.5 1.0 0.5 0.0

Solvent: $CDCl_3$
Frequency: 400 Mhz

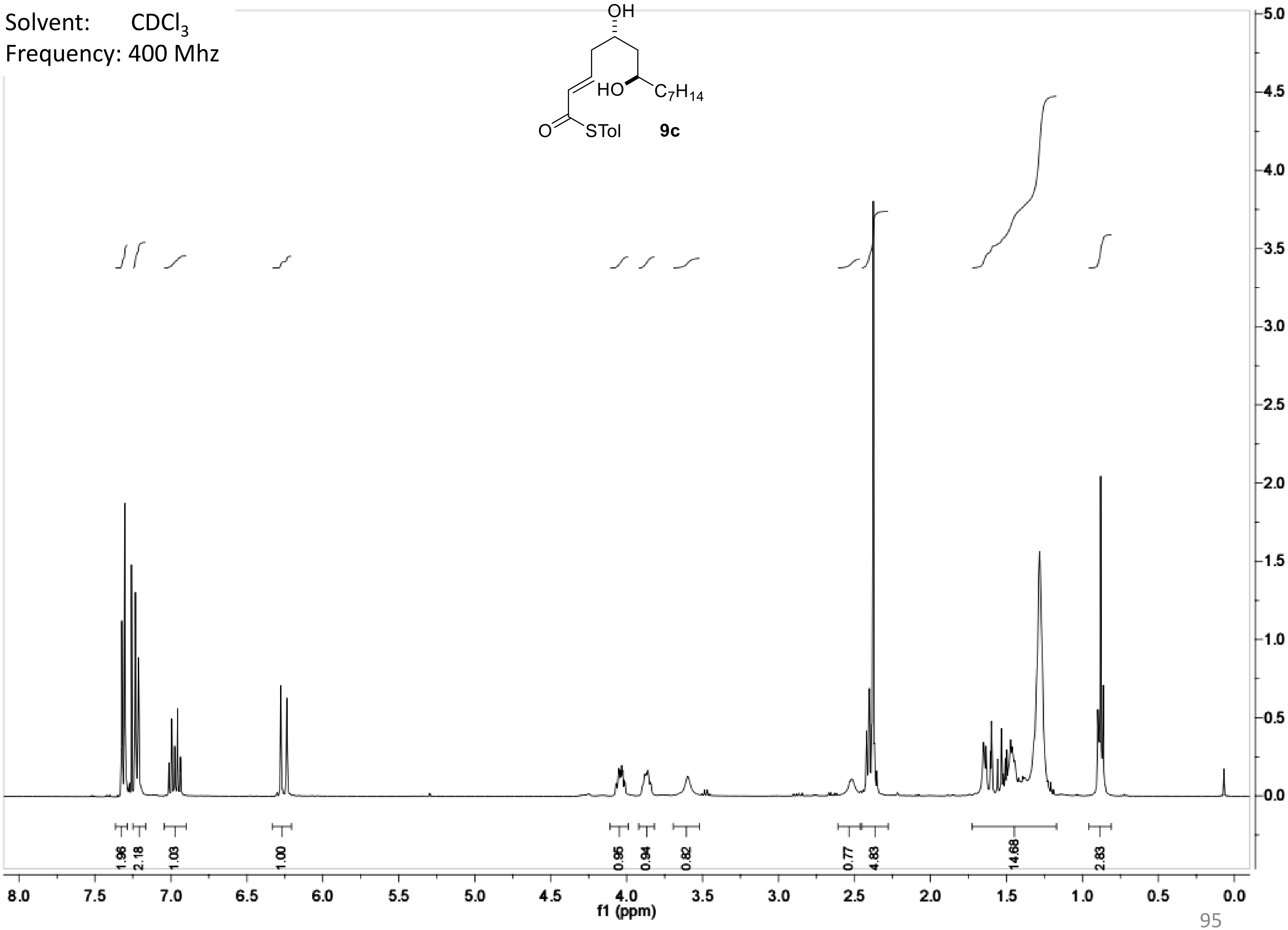

Solvent: $CDCl_3$
Frequency: 101 Mhz

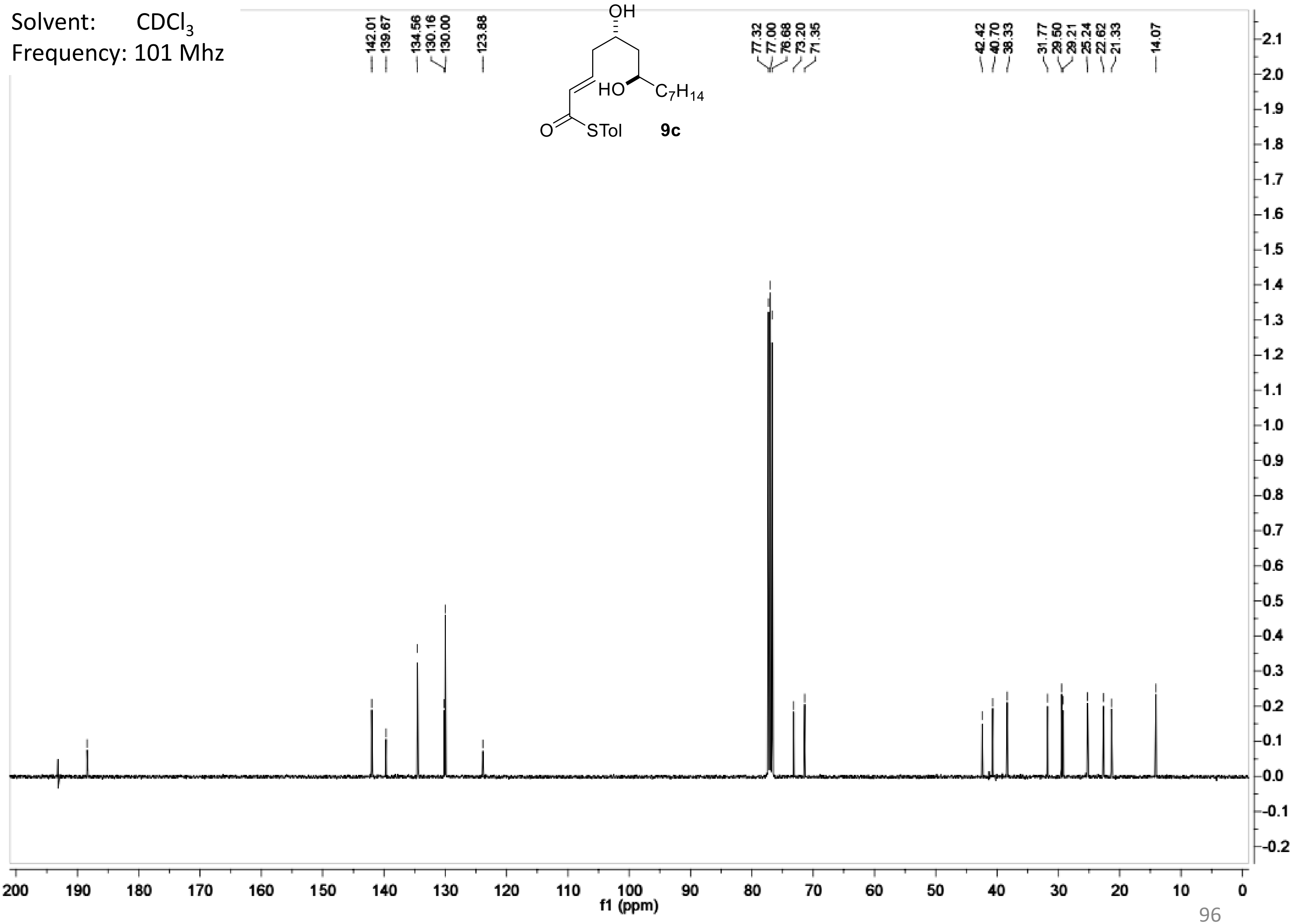

Solvent: $CDCl_3$
Frequency: 400 Mhz

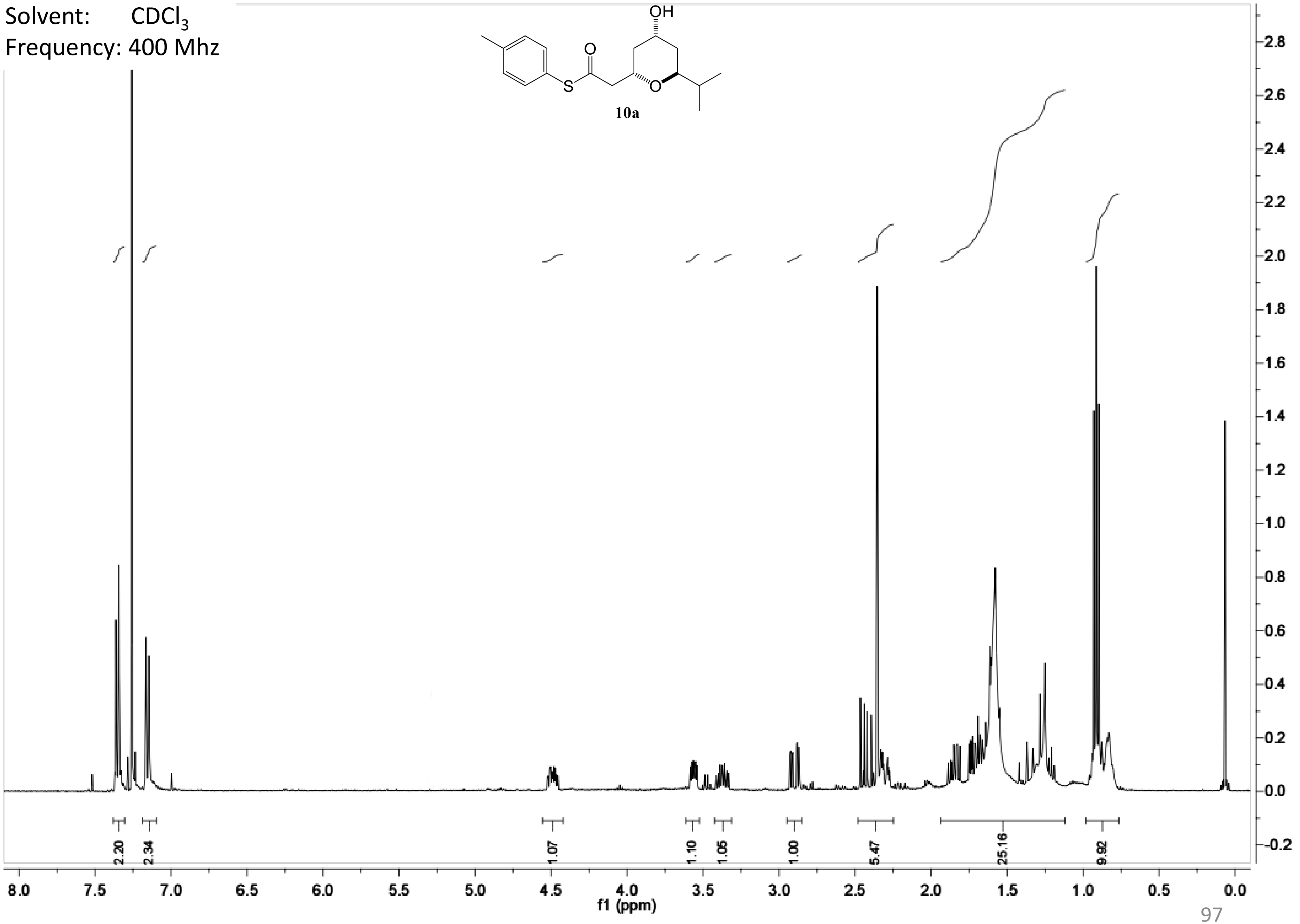

Solvent: $CDCl_3$
Frequency: 125 Mhz

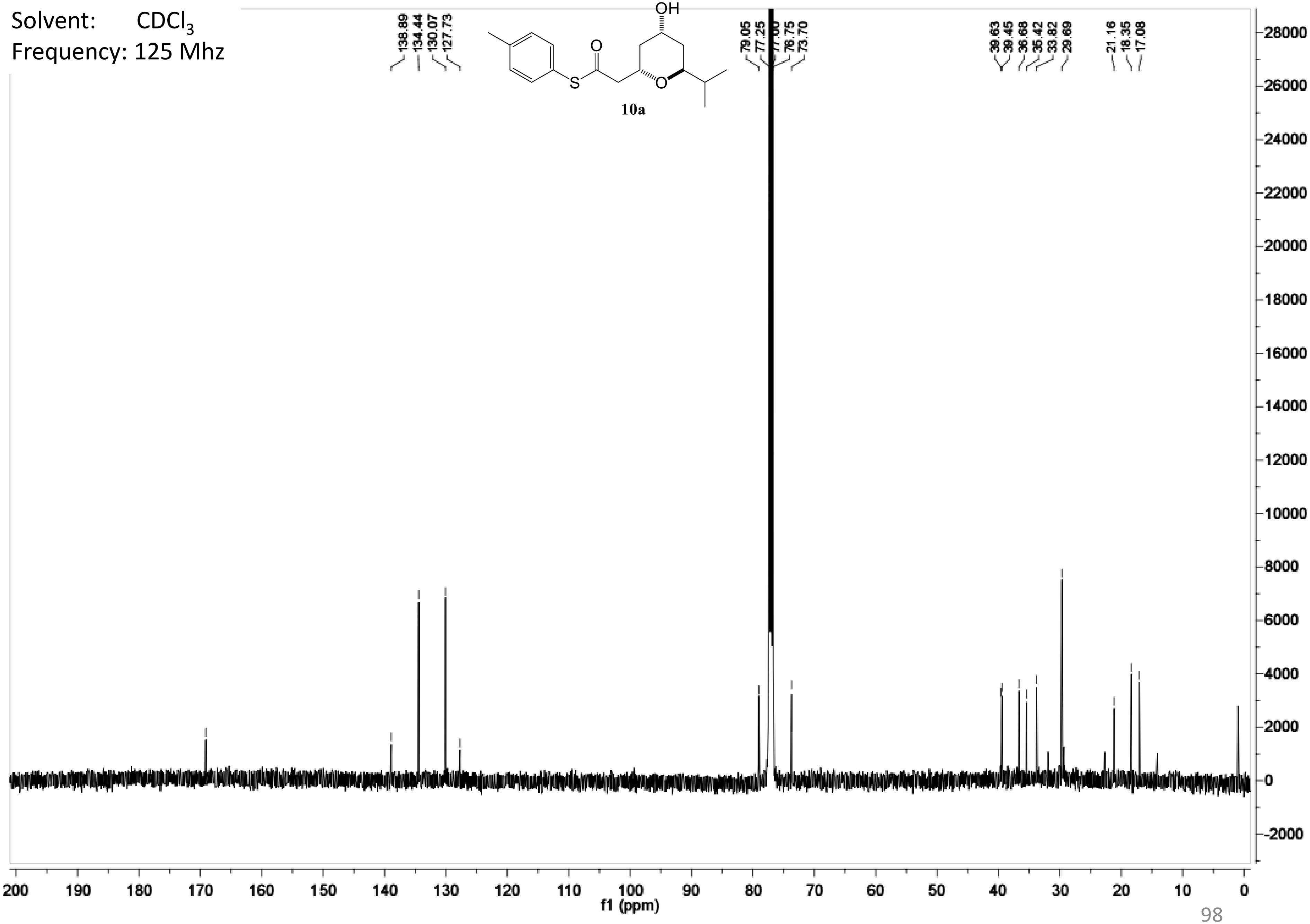

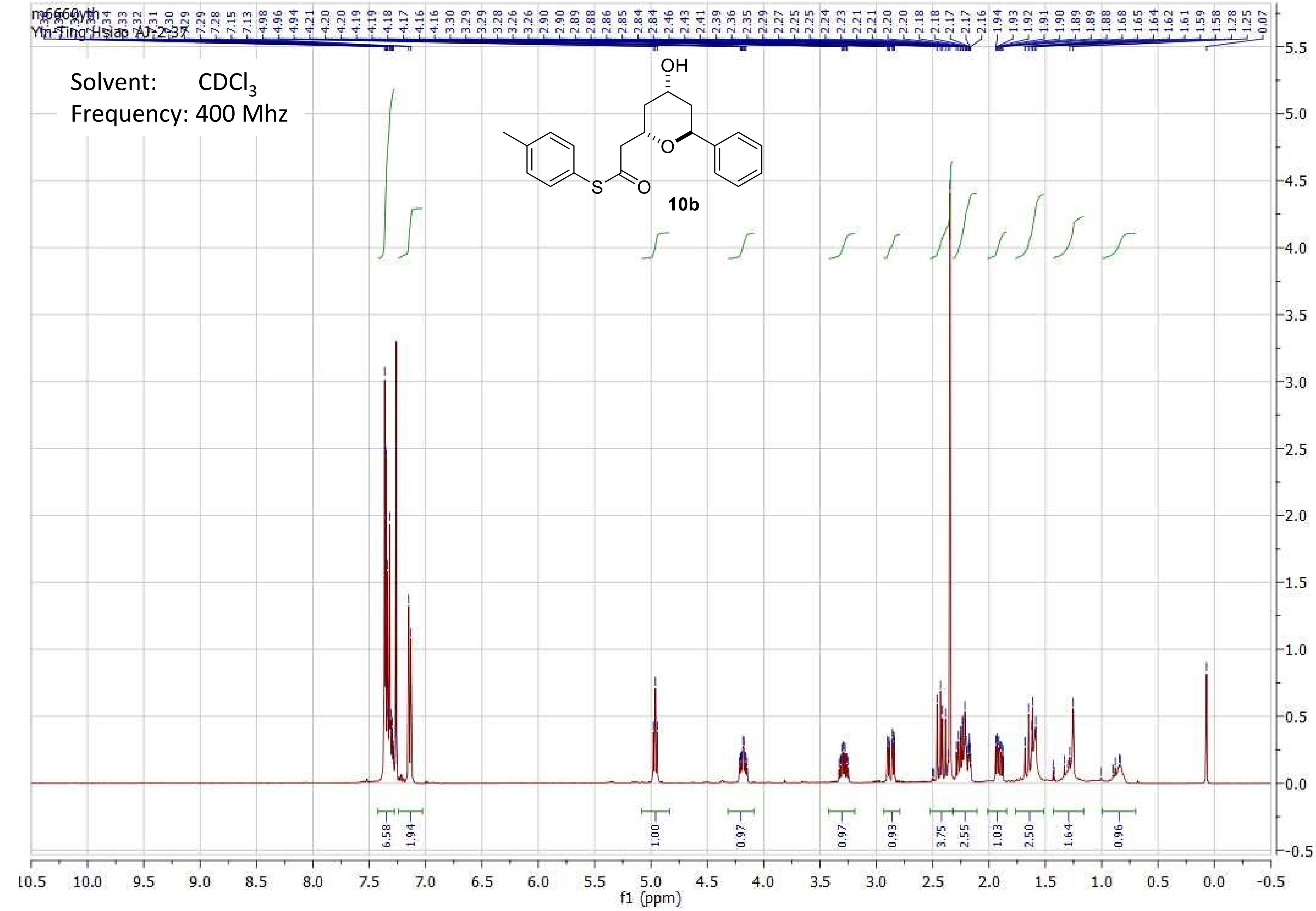

m6660yth
Yin-Ting Hsiao AJ-2-37
Solvent: $CDCl_3$
Frequency: 400 Mhz
OH
O
S
O
10b
f1 (ppm)

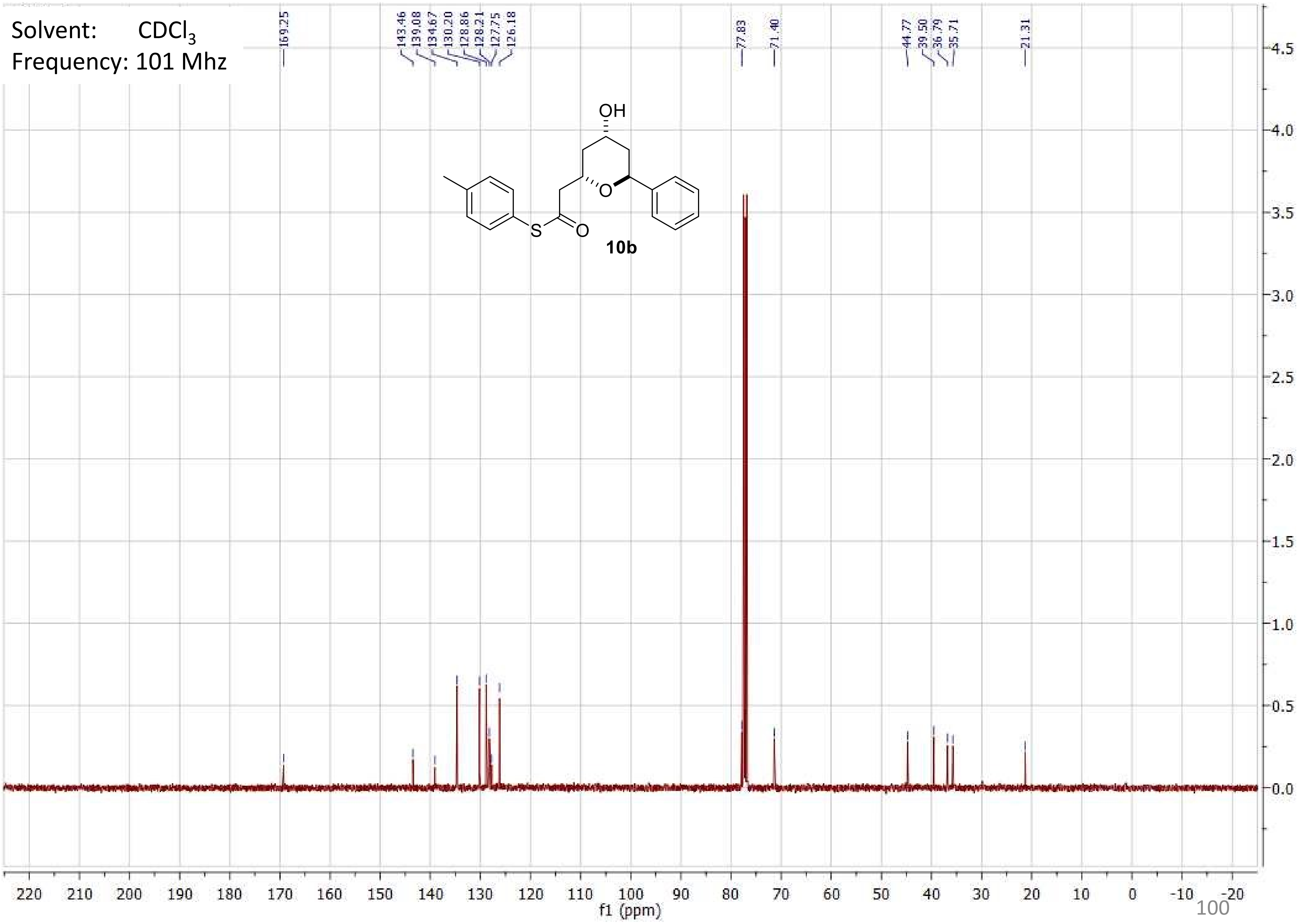
Solvent: CDCl3
Frequency: 101 Mhz
169.25
143.46
139.08
134.67
130.20
128.86
128.21
127.75
126.18
77.83
71.40
44.77
39.50
36.79
35.71
21.31
OH
O
S
O
10b
f1 (ppm)

Solvent: $CDCl_3$
Frequency: 400 Mhz

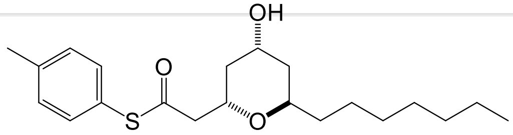

10c

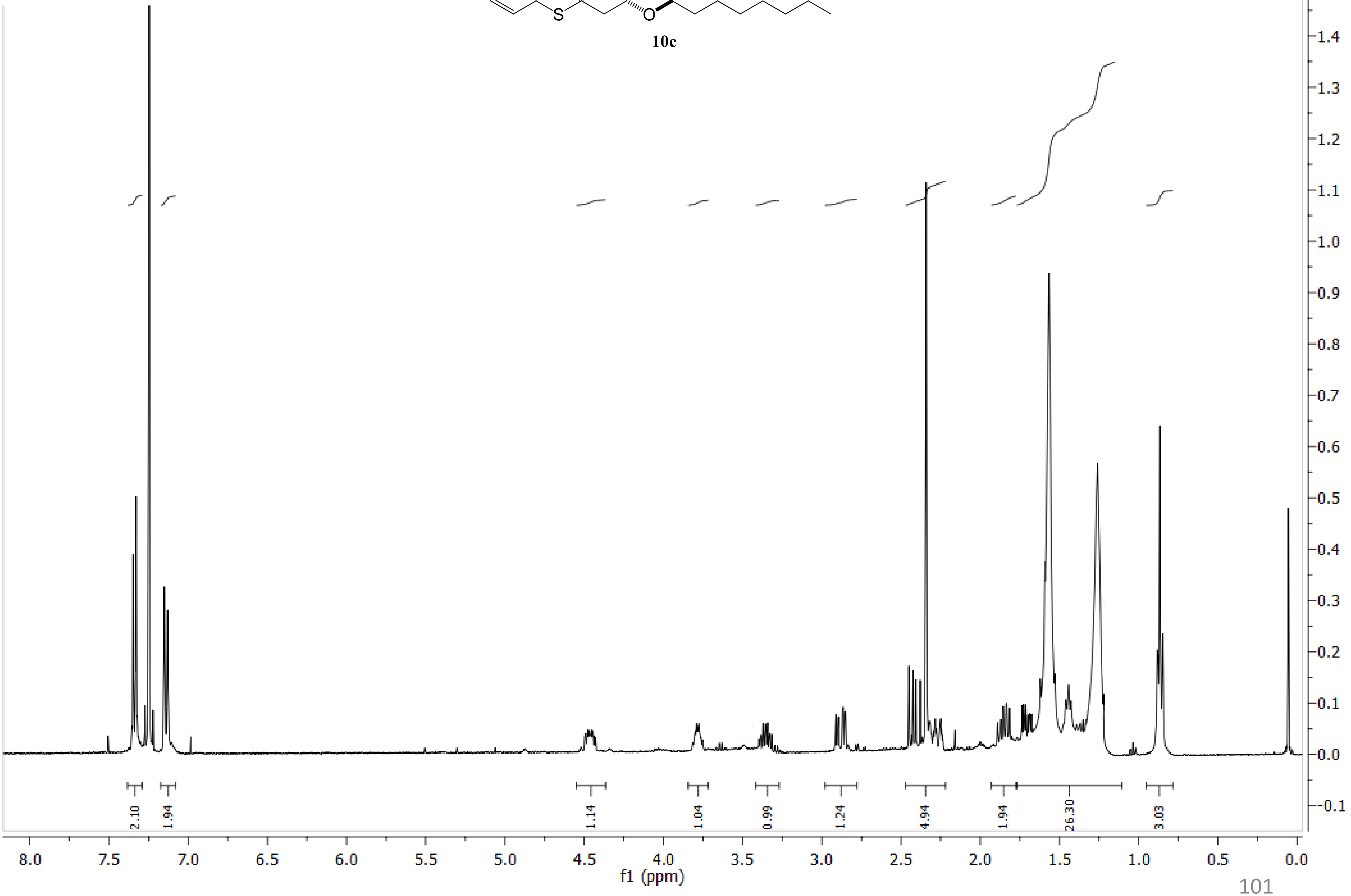

Solvent: $CDCl_3$
Frequency: 125 Mhz

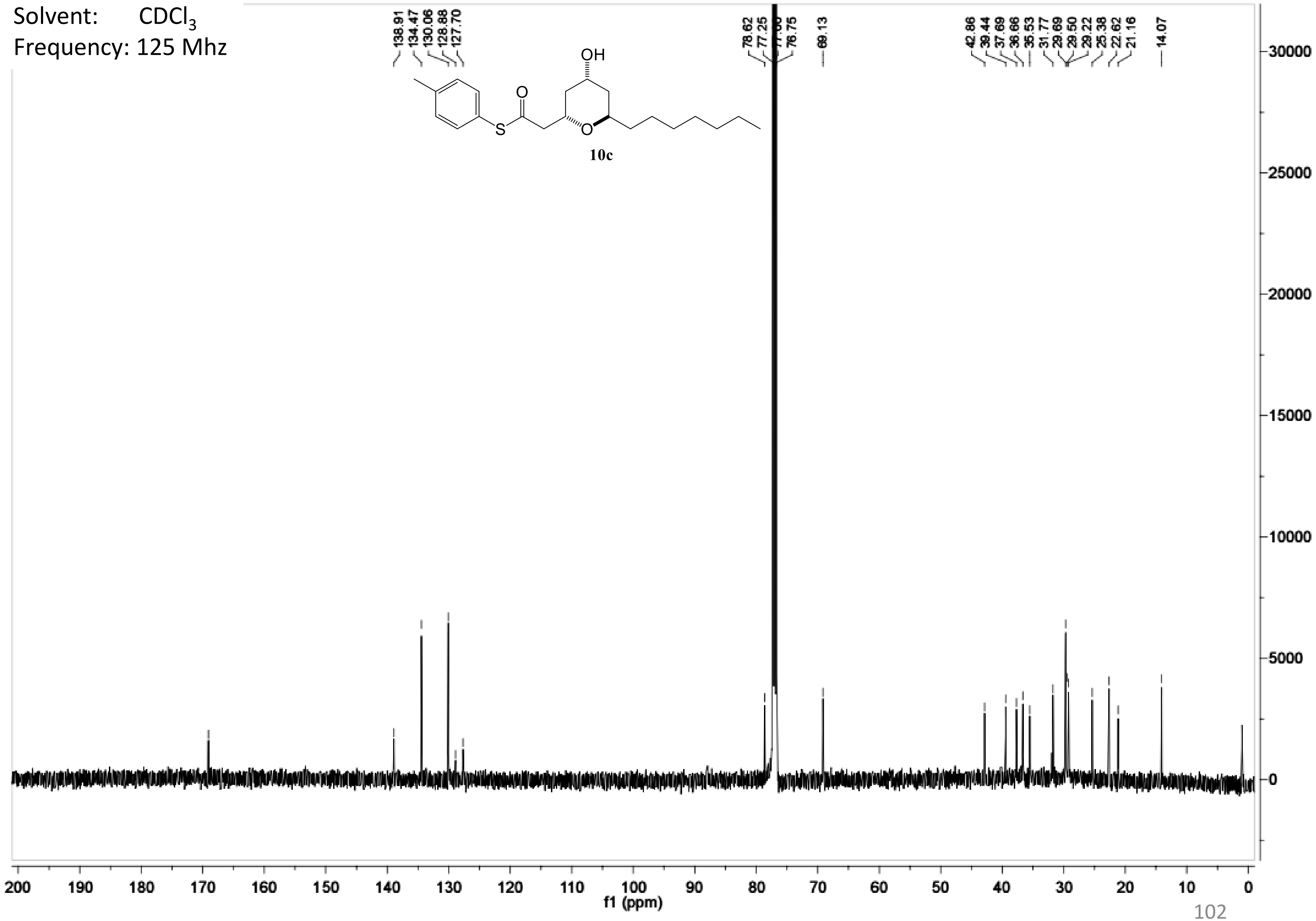

Solvent: $CDCl_3$
Frequency: 400 Mhz

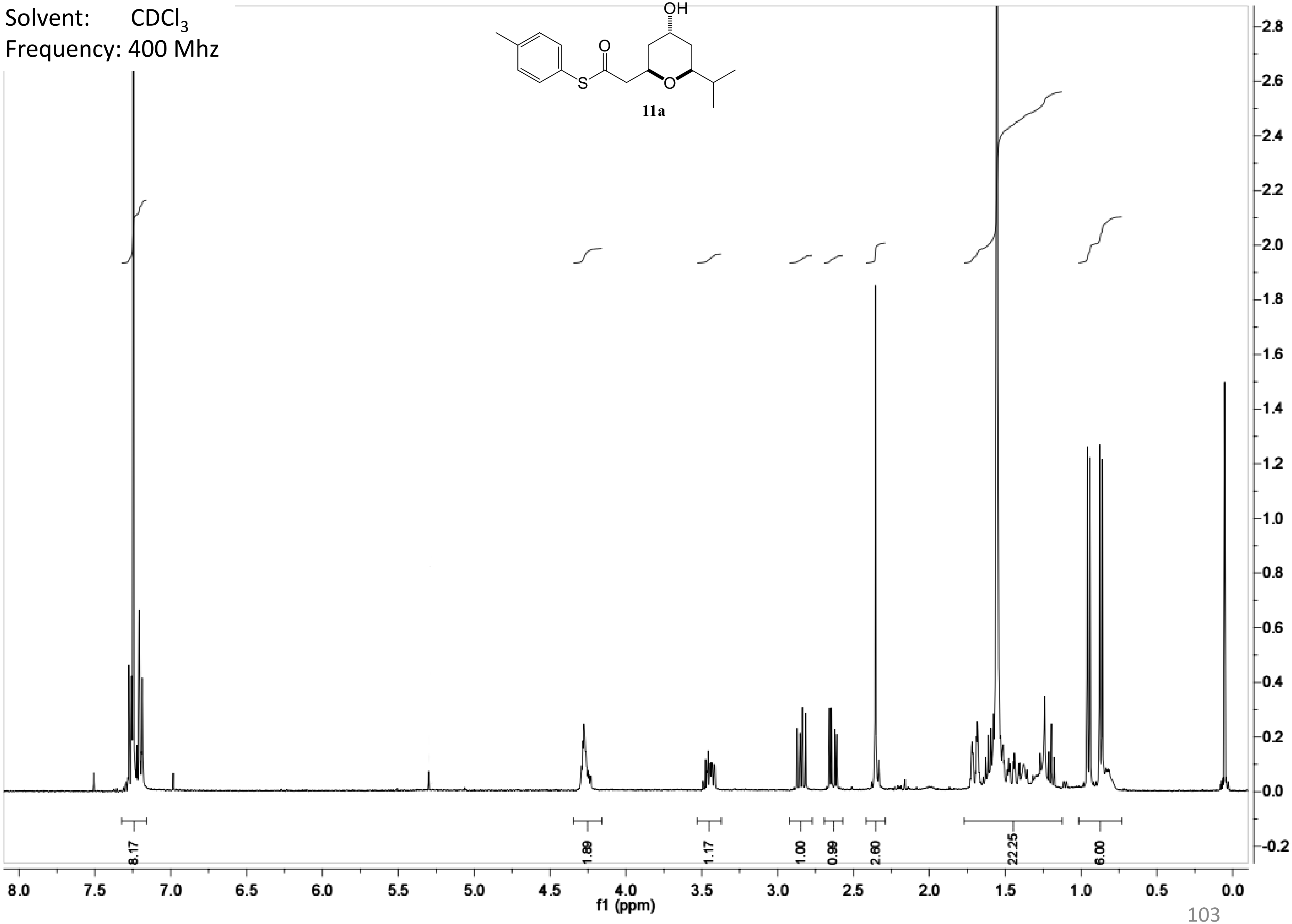

Solvent: $CDCl_3$
Frequency: 101 Mhz

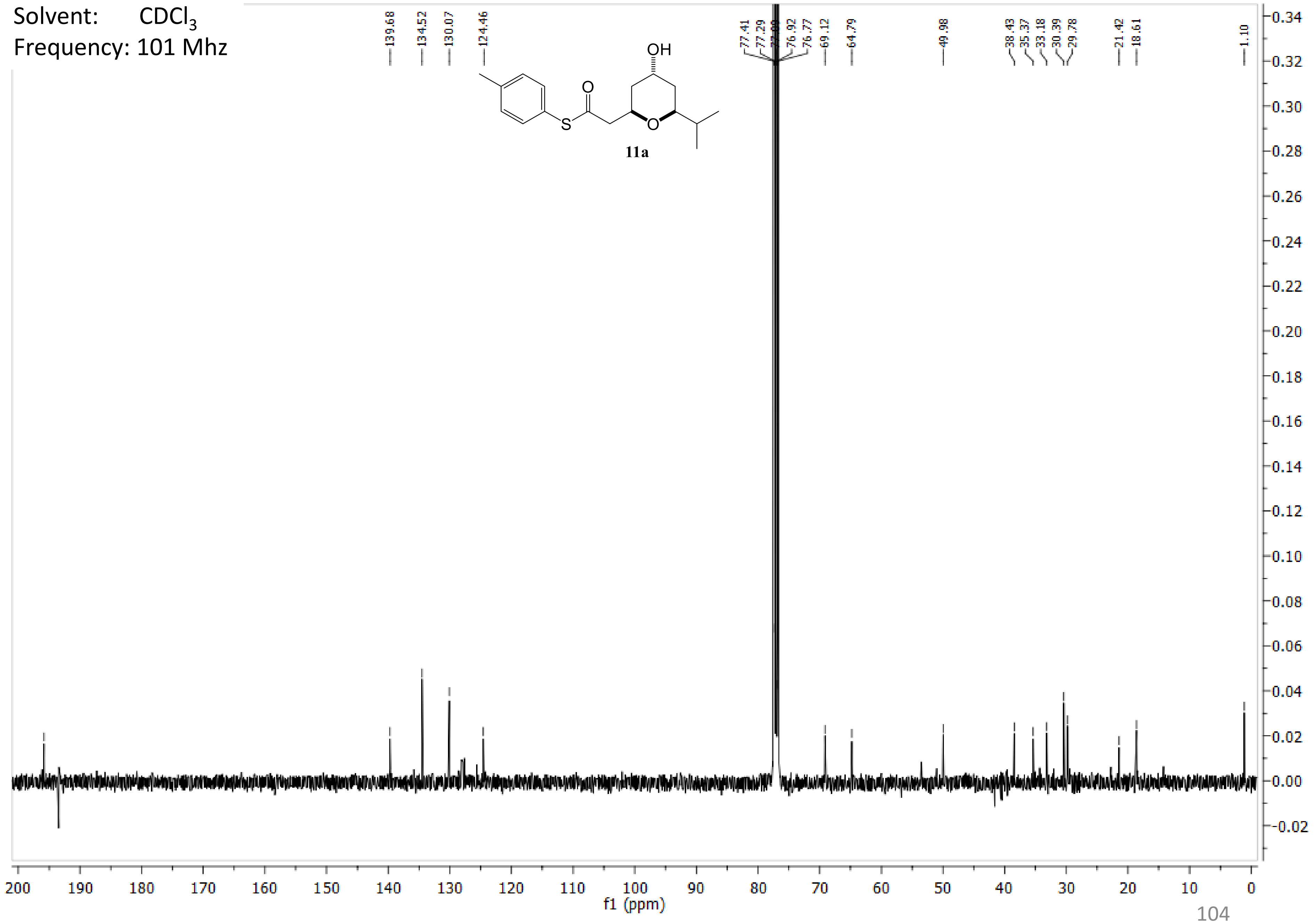

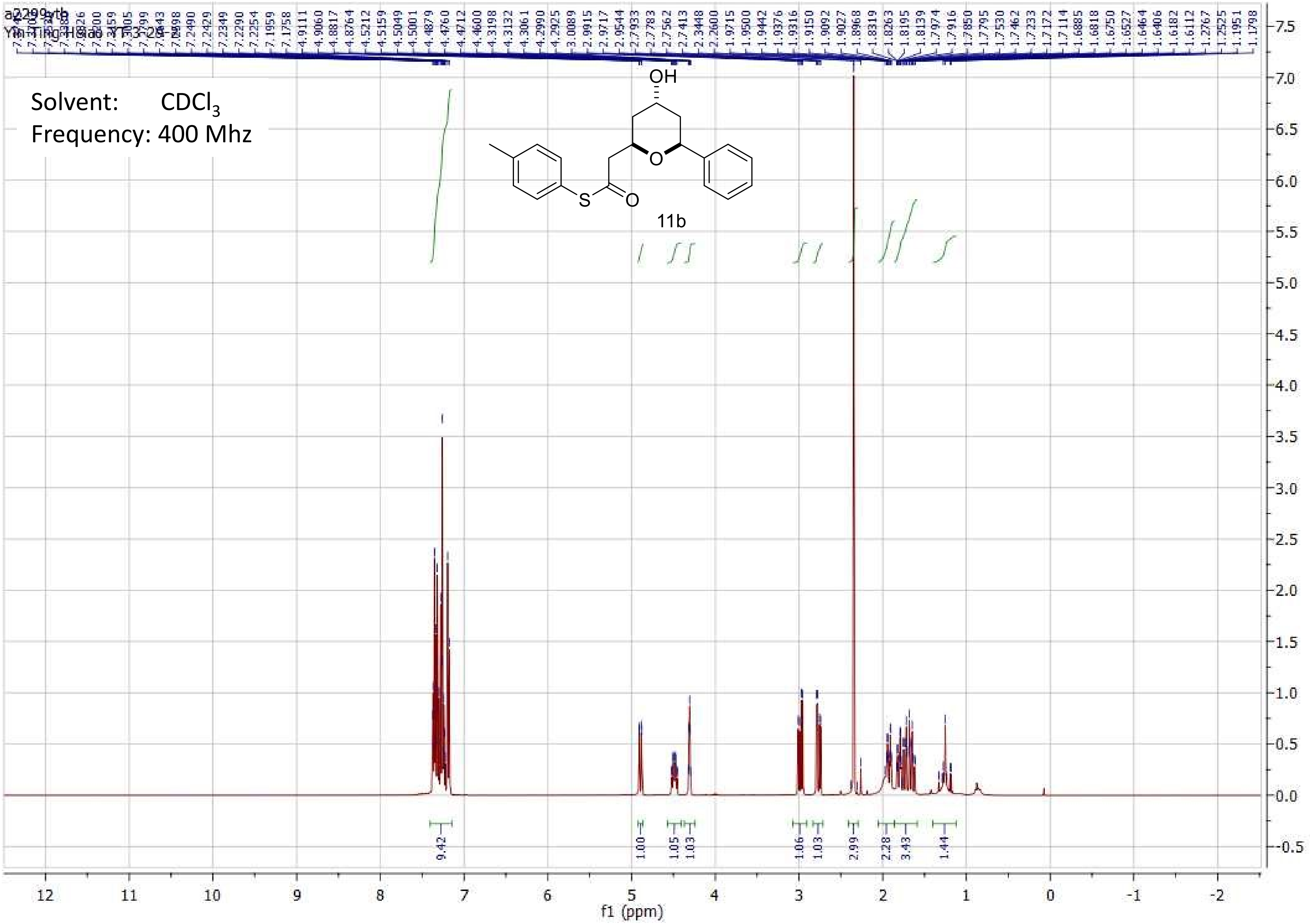
Solvent: CDCl$_3$
Frequency: 400 Mhz
OH
O
S
O
11b
9.42
1.00
1.05
1.03
1.06
1.03
2.99
2.28
3.43
1.44
f1 (ppm)

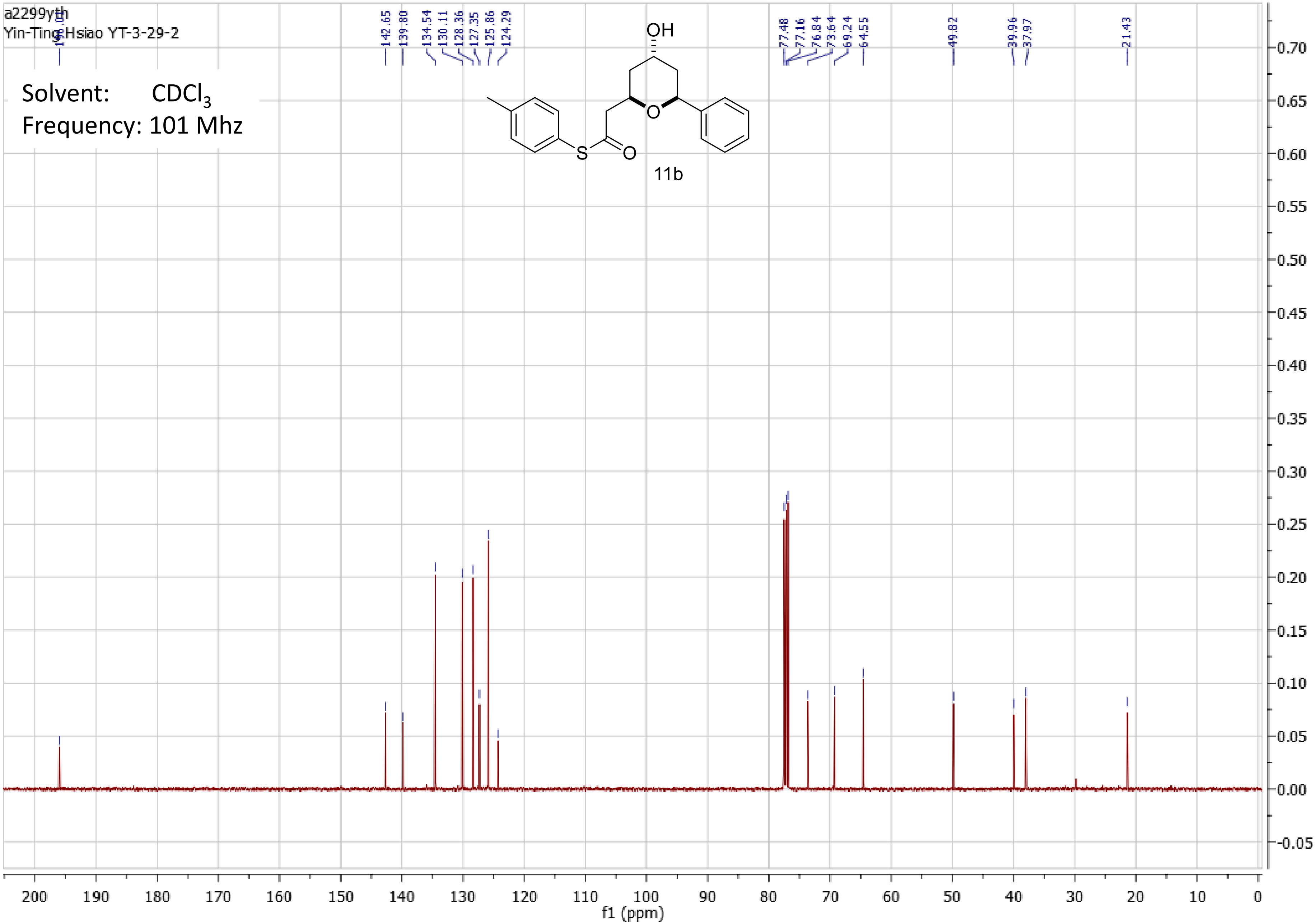
a2299yth
Yin-Ting Hsiao YT-3-29-2
Solvent: CDCl3
Frequency: 101 Mhz
142.65
139.80
134.54
130.11
128.36
127.35
125.86
124.29
77.48
77.16
76.84
73.64
69.24
64.55
49.82
39.96
37.97
21.43
OH
O
S
O
11b
f1 (ppm)

Solvent: $CDCl_3$
Frequency: 400 Mhz

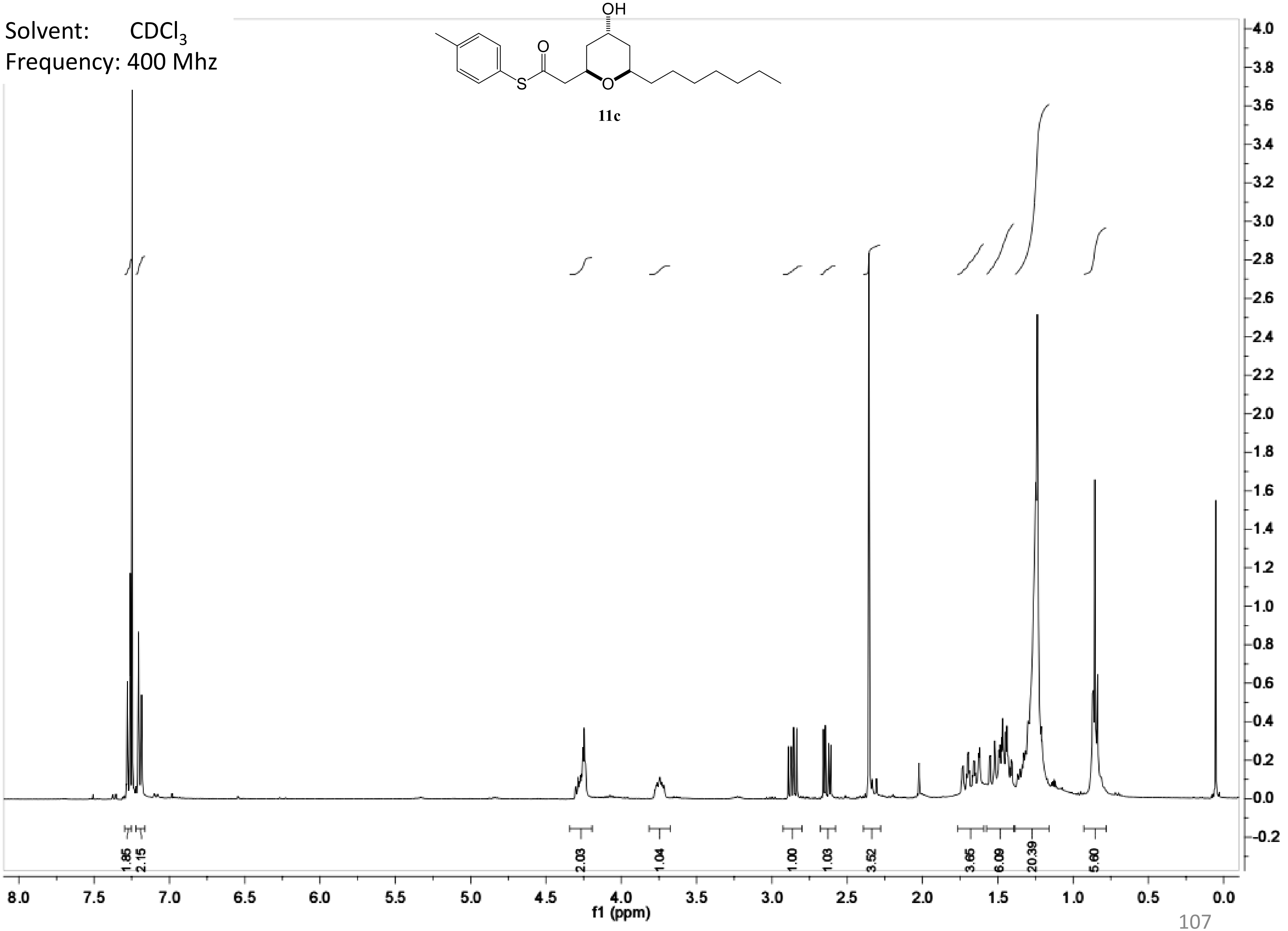

Solvent: $CDCl_3$
Frequency: 101 Mhz

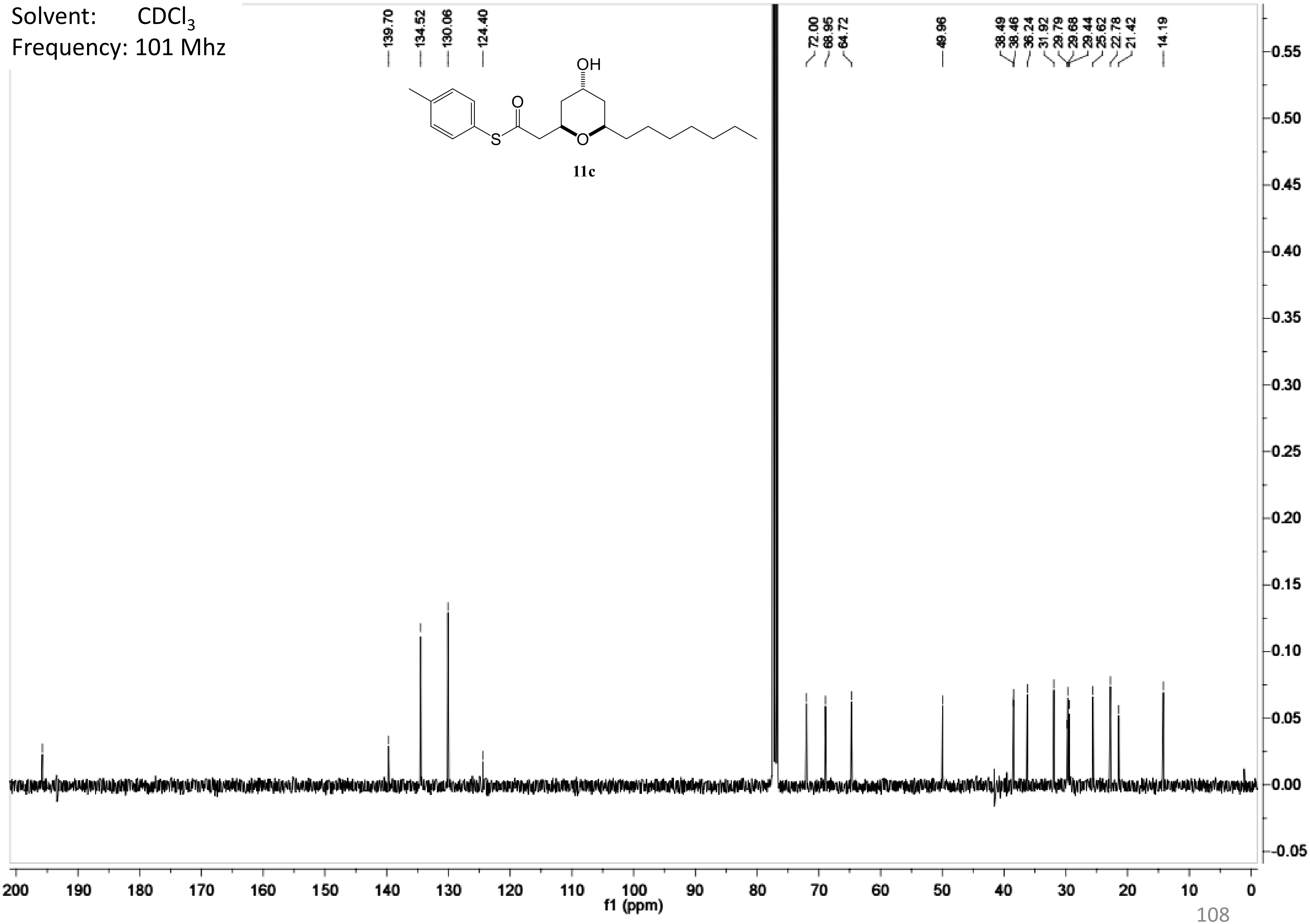

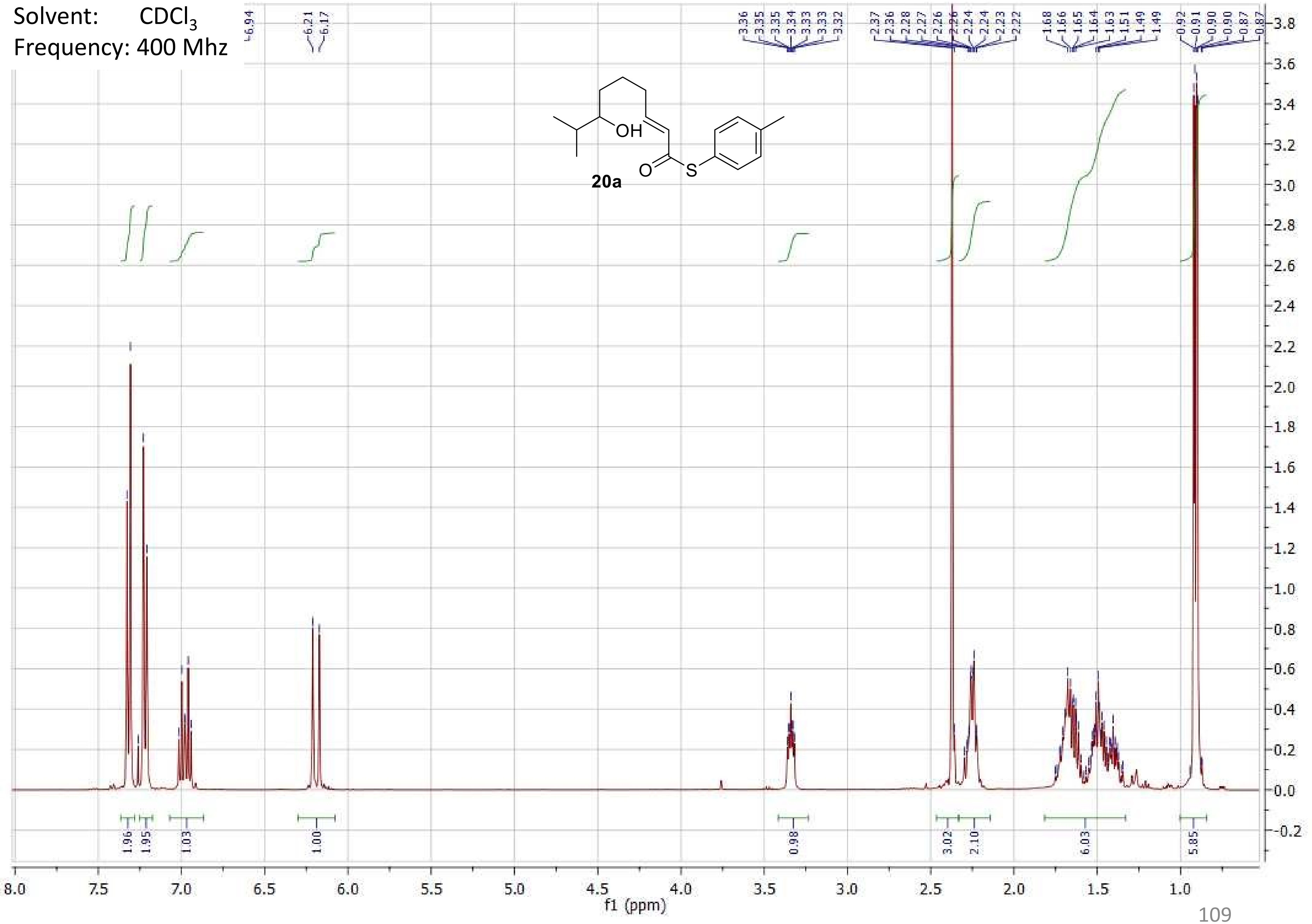
Solvent: $CDCl_3$
Frequency: 400 Mhz
20a
OH
O
S
6.94
6.21
6.17
3.36
3.35
3.35
3.34
3.33
3.33
3.33
3.32
2.37
2.36
2.28
2.27
2.26
2.26
2.24
2.24
2.23
2.22
1.68
1.66
1.65
1.64
1.63
1.51
1.49
1.49
0.92
0.91
0.90
0.90
0.87
0.87
1.96
1.95
1.03
1.00
0.98
3.02
2.10
6.03
5.85
f1 (ppm)

Solvent: $CDCl_3$
Frequency: 101 Mhz

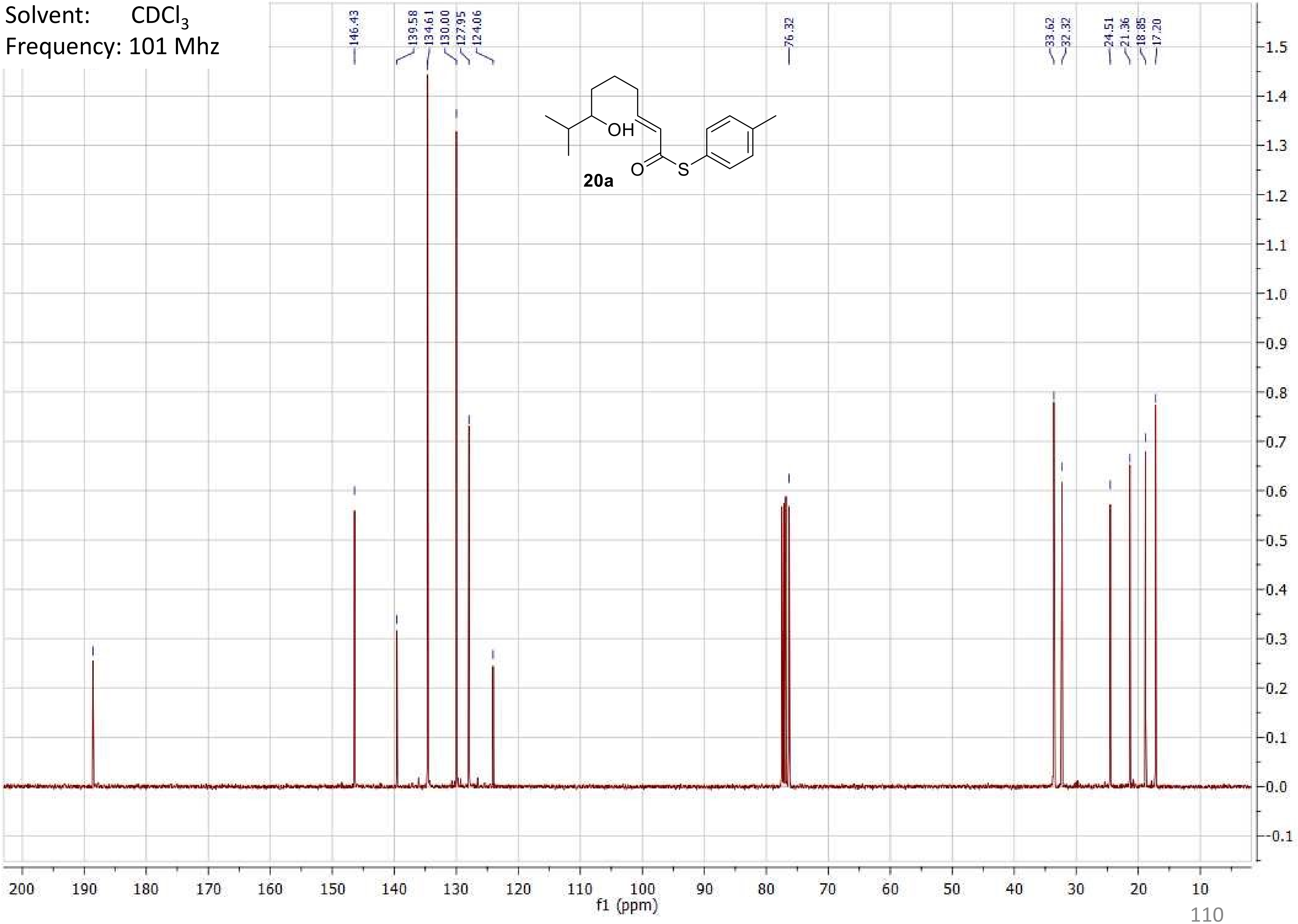

Solvent: $CDCl_3$
Frequency: 400 Mhz

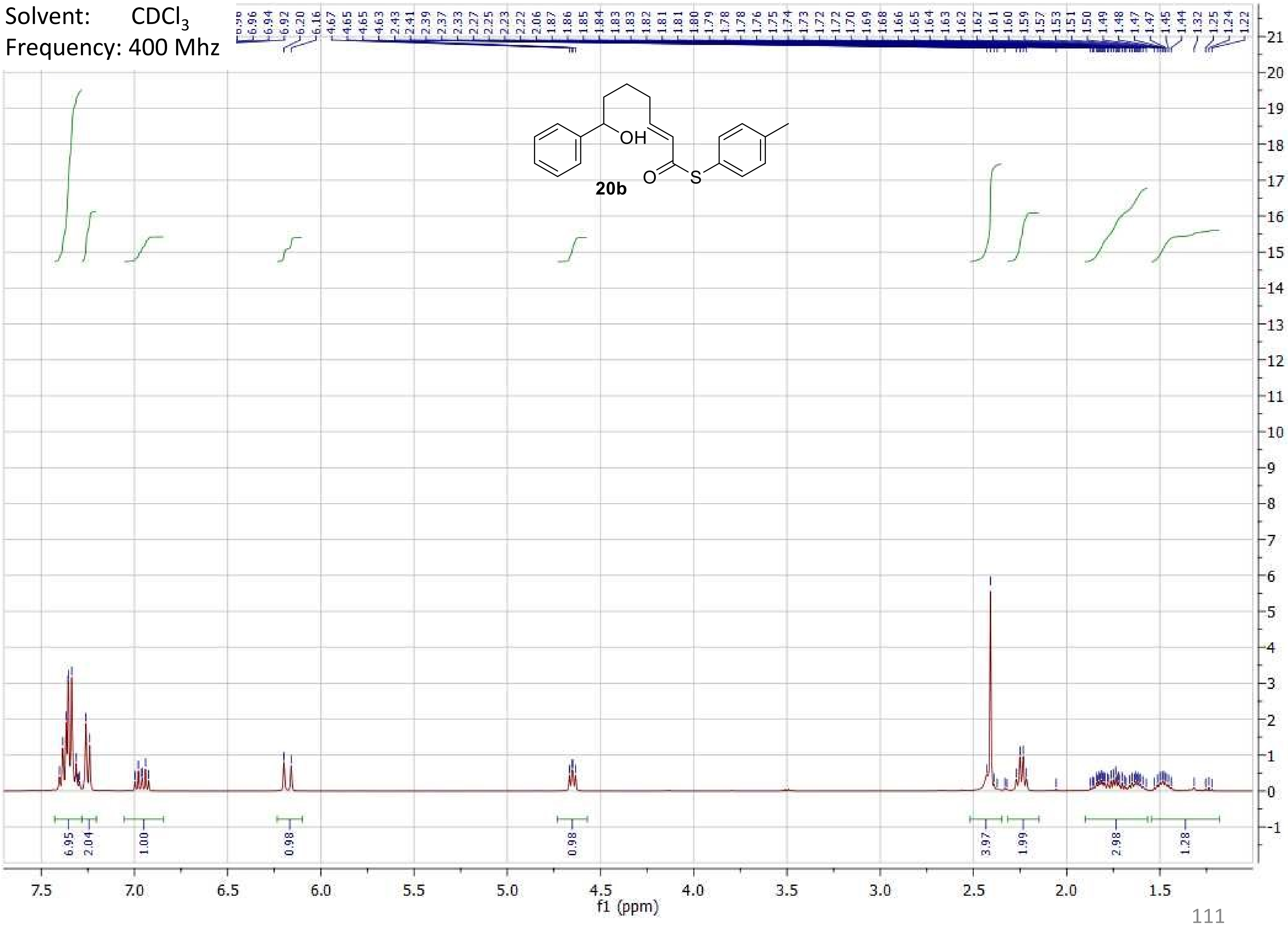

Solvent: $CDCl_3$
Frequency: 101 Mhz

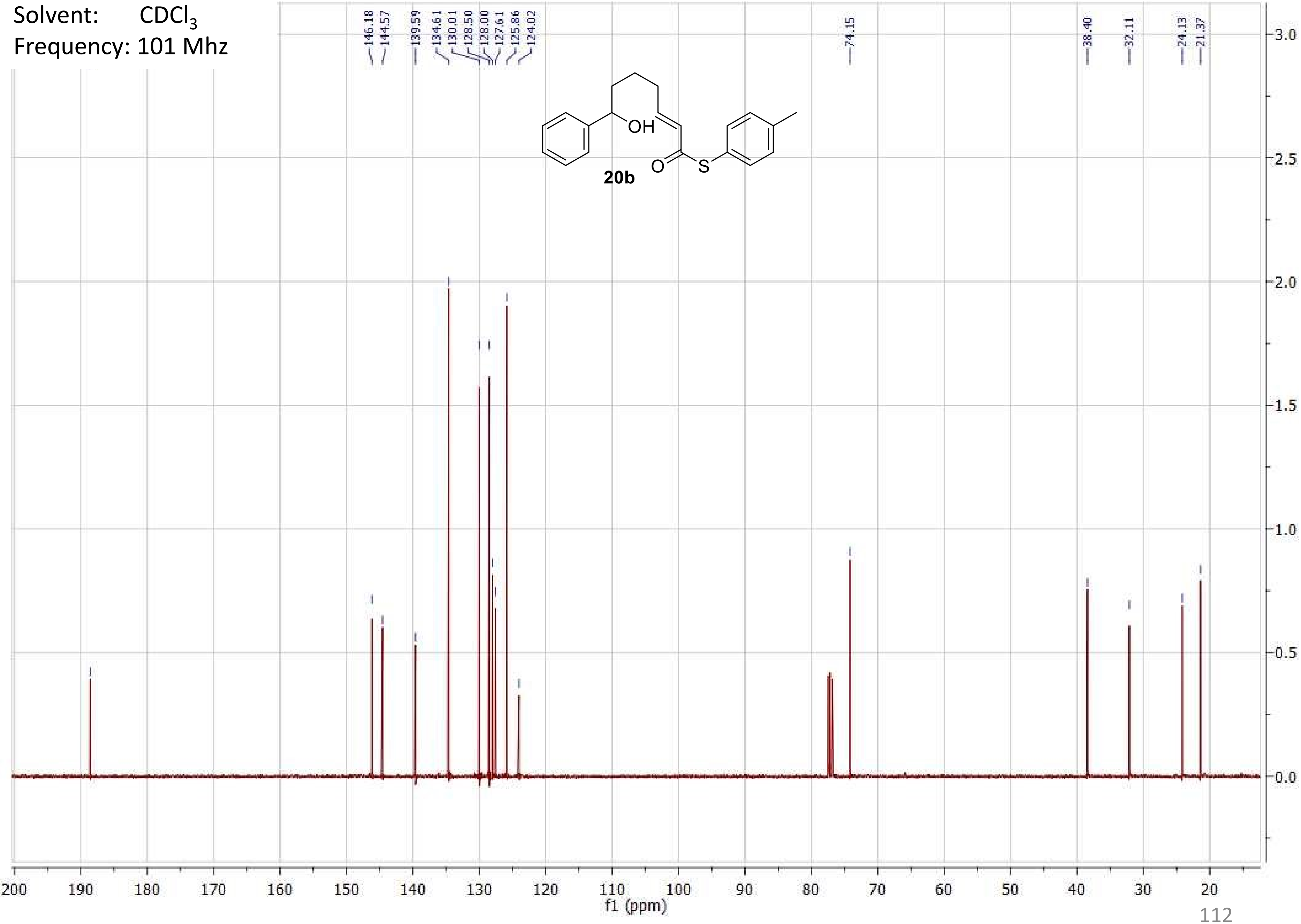

Solvent: $CDCl_3$
Frequency: 400 Mhz

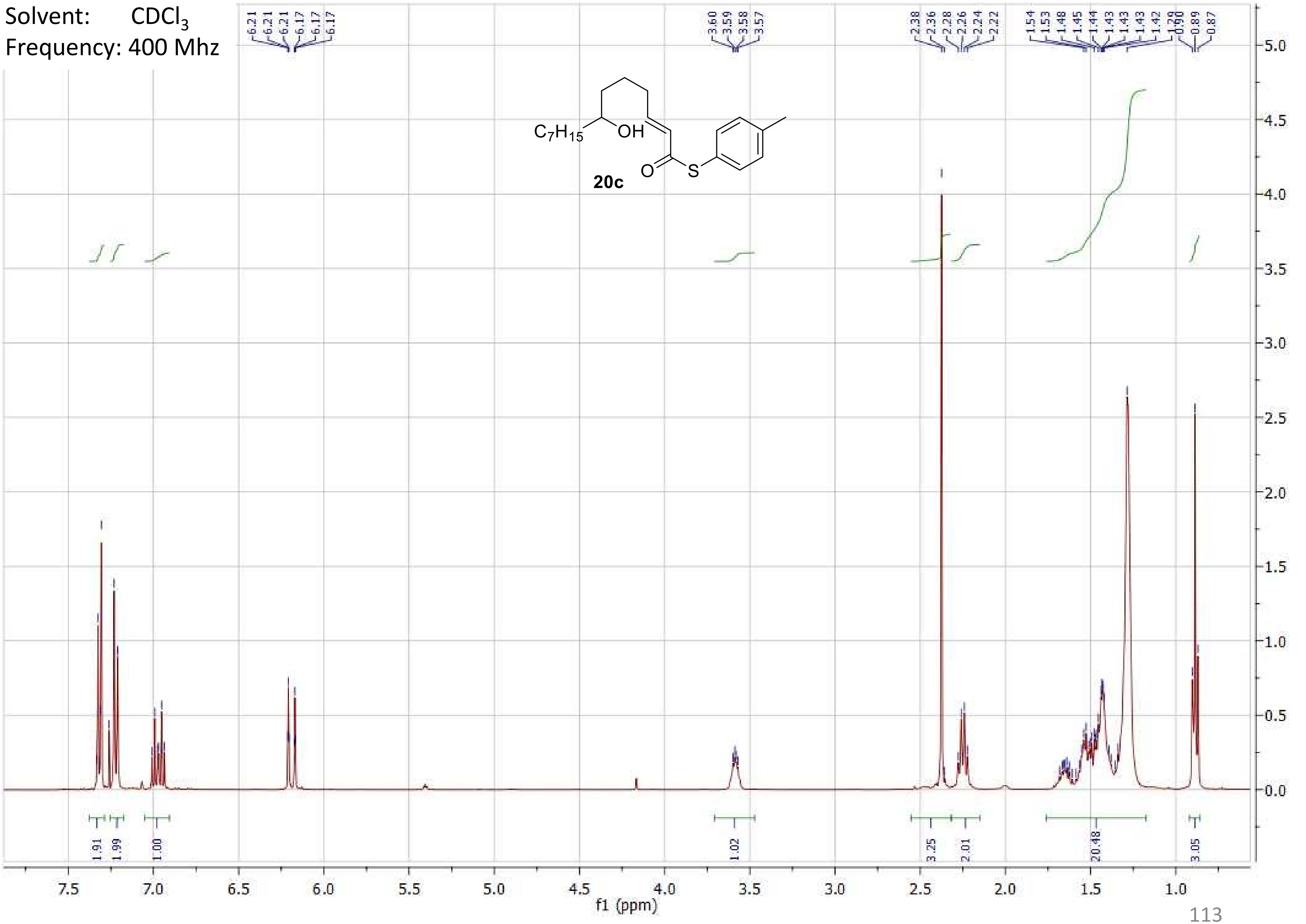

Solvent: $CDCl_3$
Frequency: 101 Mhz

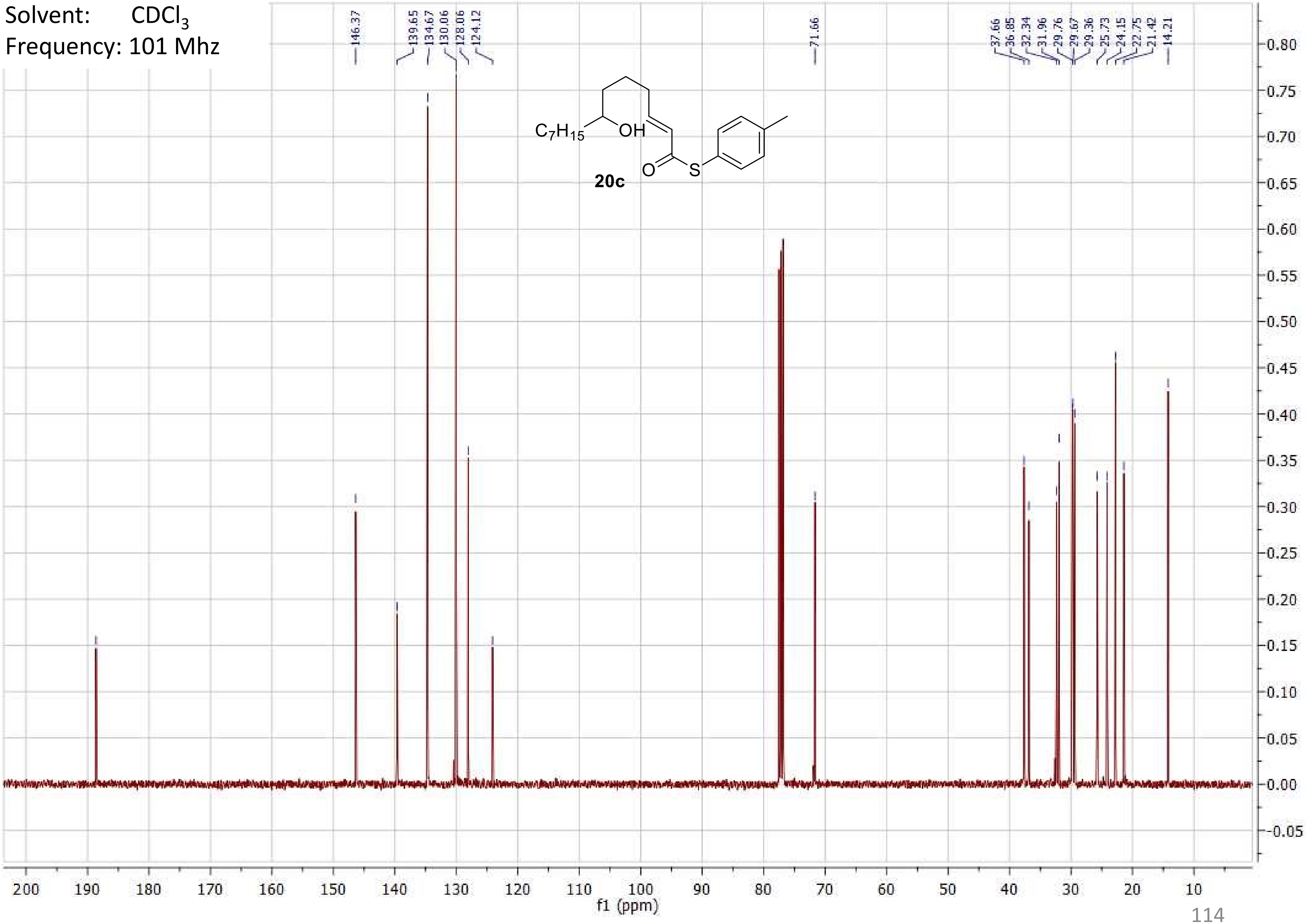

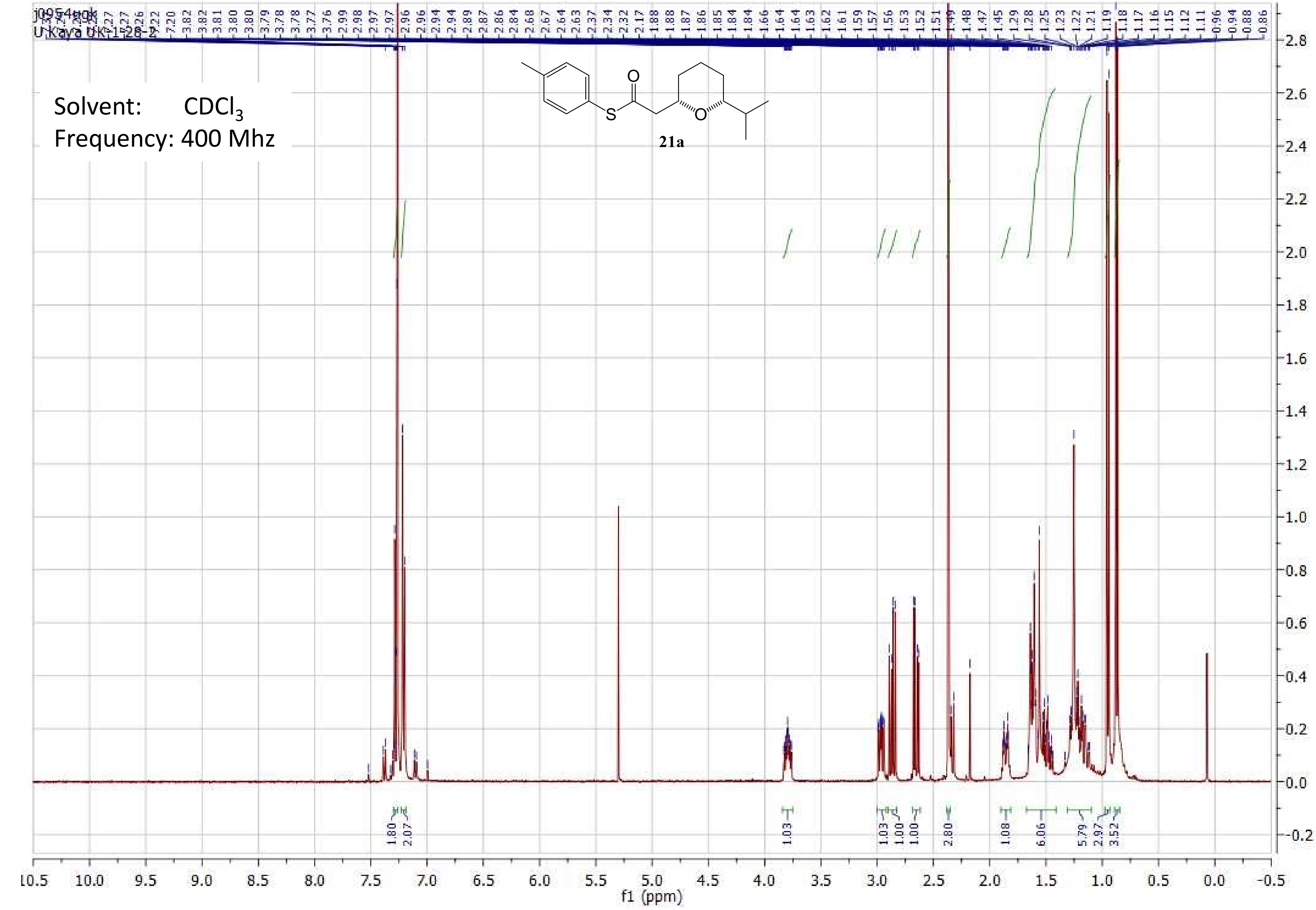
Solvent: CDCl3
Frequency: 400 Mhz
21a
f1 (ppm)

Solvent: $CDCl_3$
Frequency: 101 Mhz

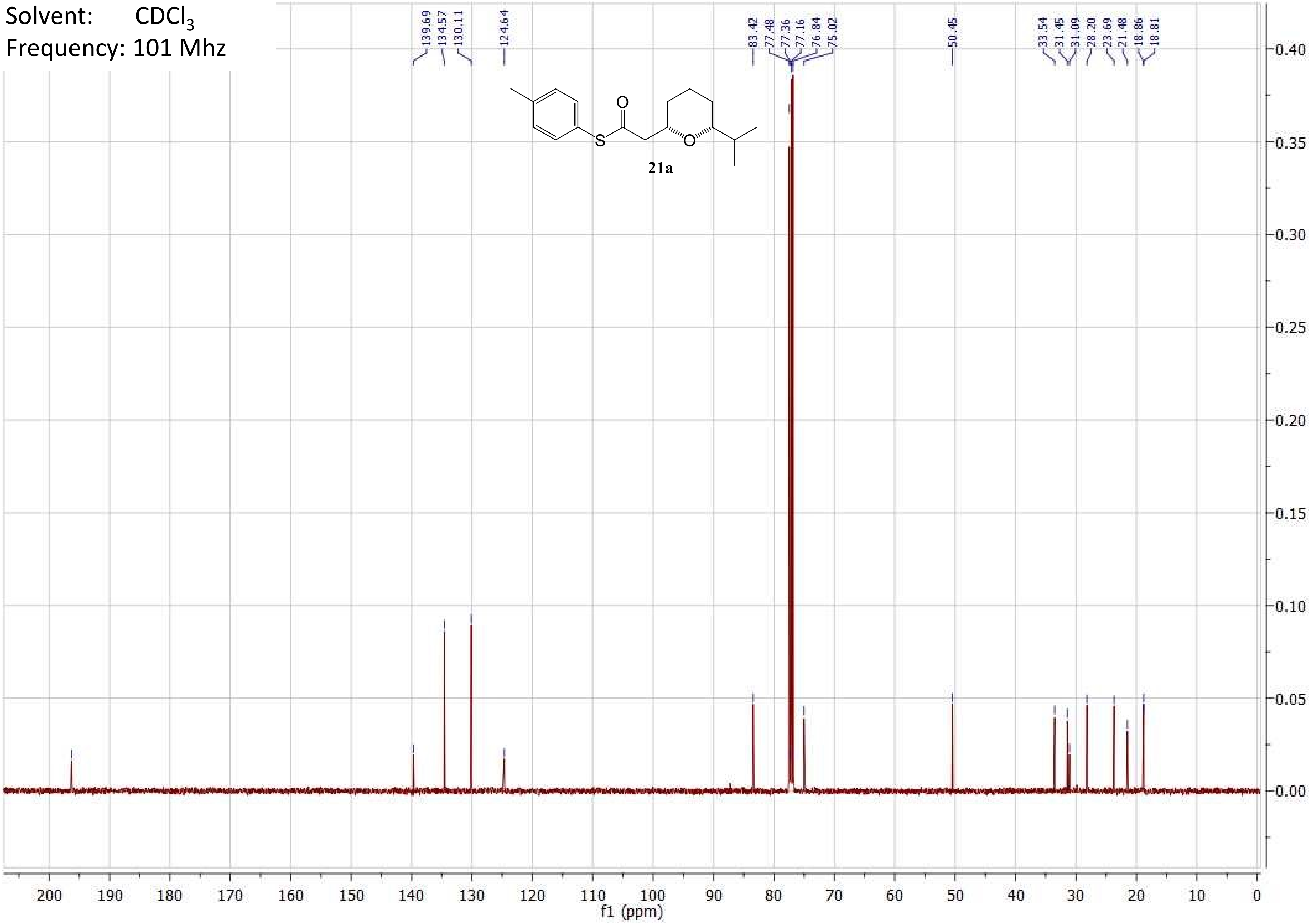

Solvent: $CDCl_3$
Frequency: 400 Mhz

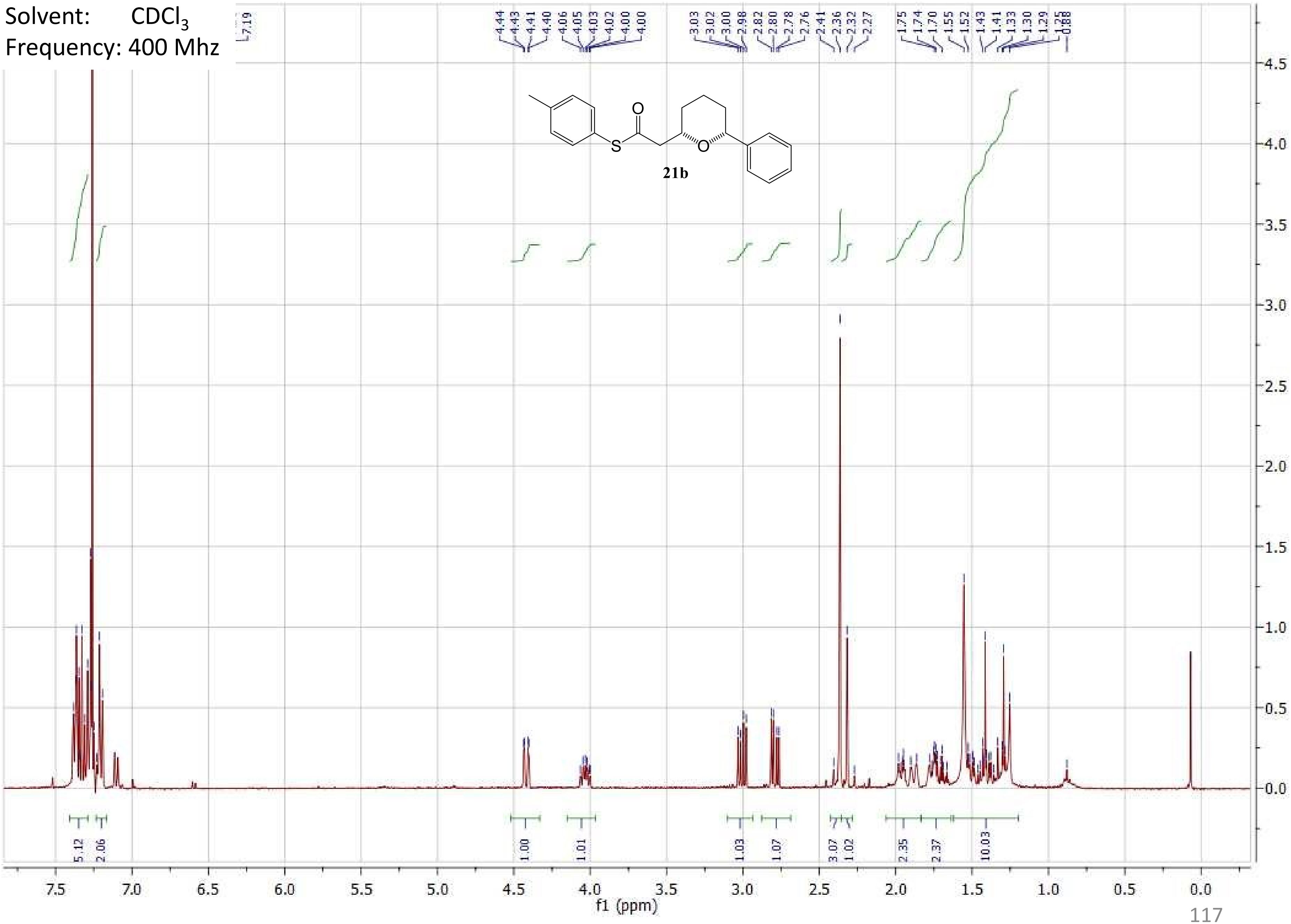

Solvent: $CDCl_3$
Frequency: 101 Mhz

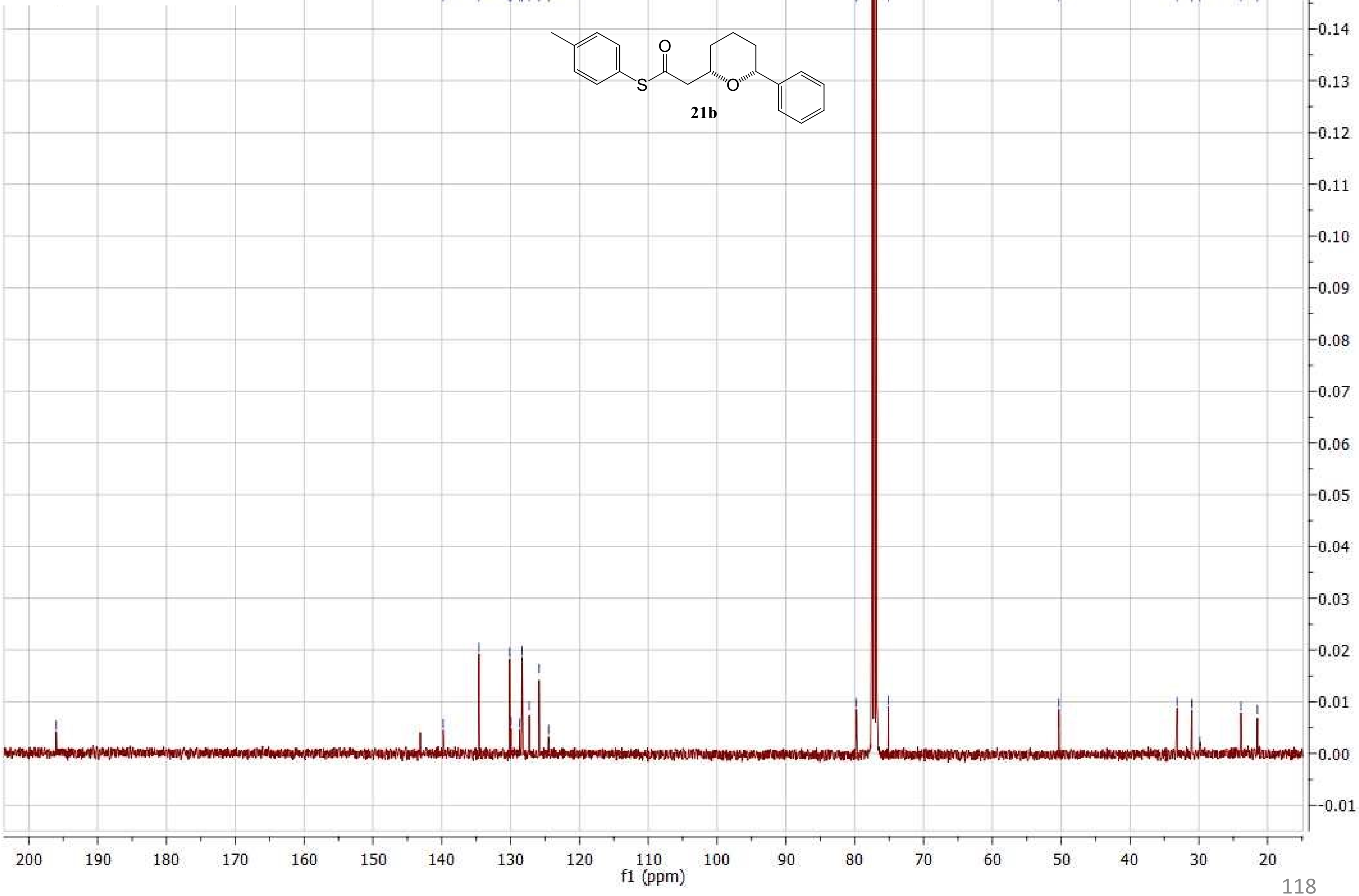

Solvent: $CDCl_3$

Frequency: 400 Mhz

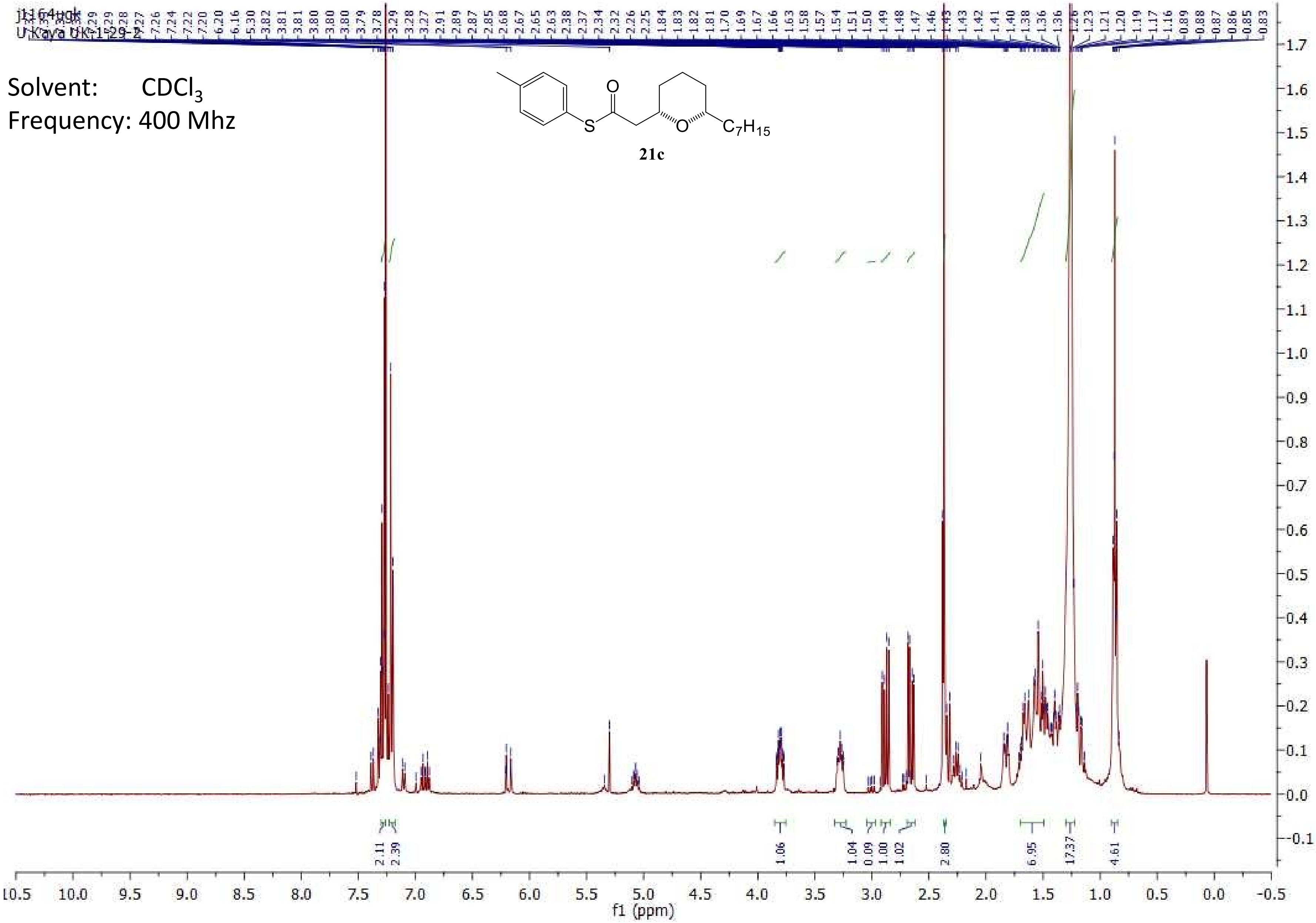

Solvent: $CDCl_3$
Frequency: 101 Mhz

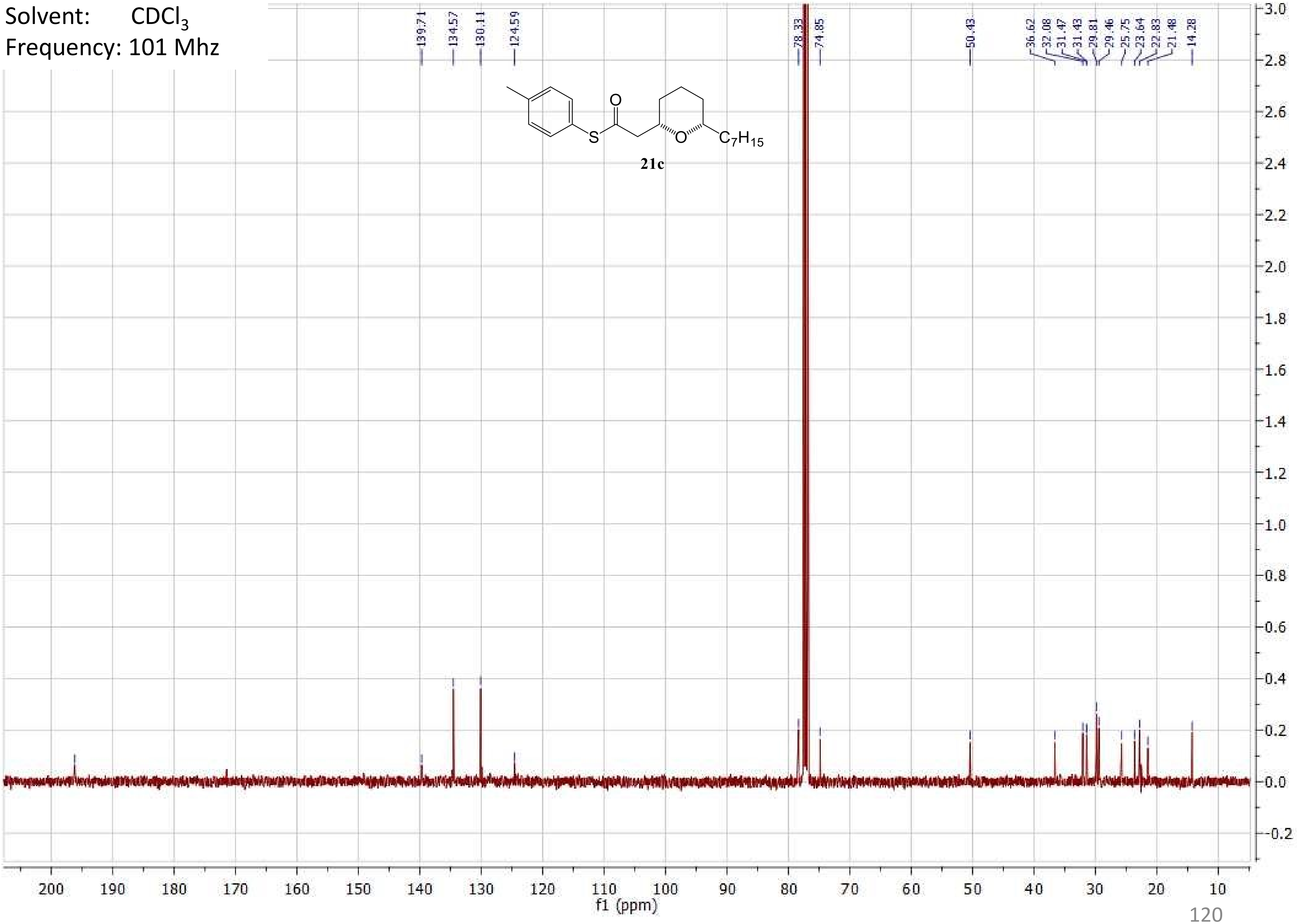

Solvent: $CDCl_3$
Frequency: 400 Mhz

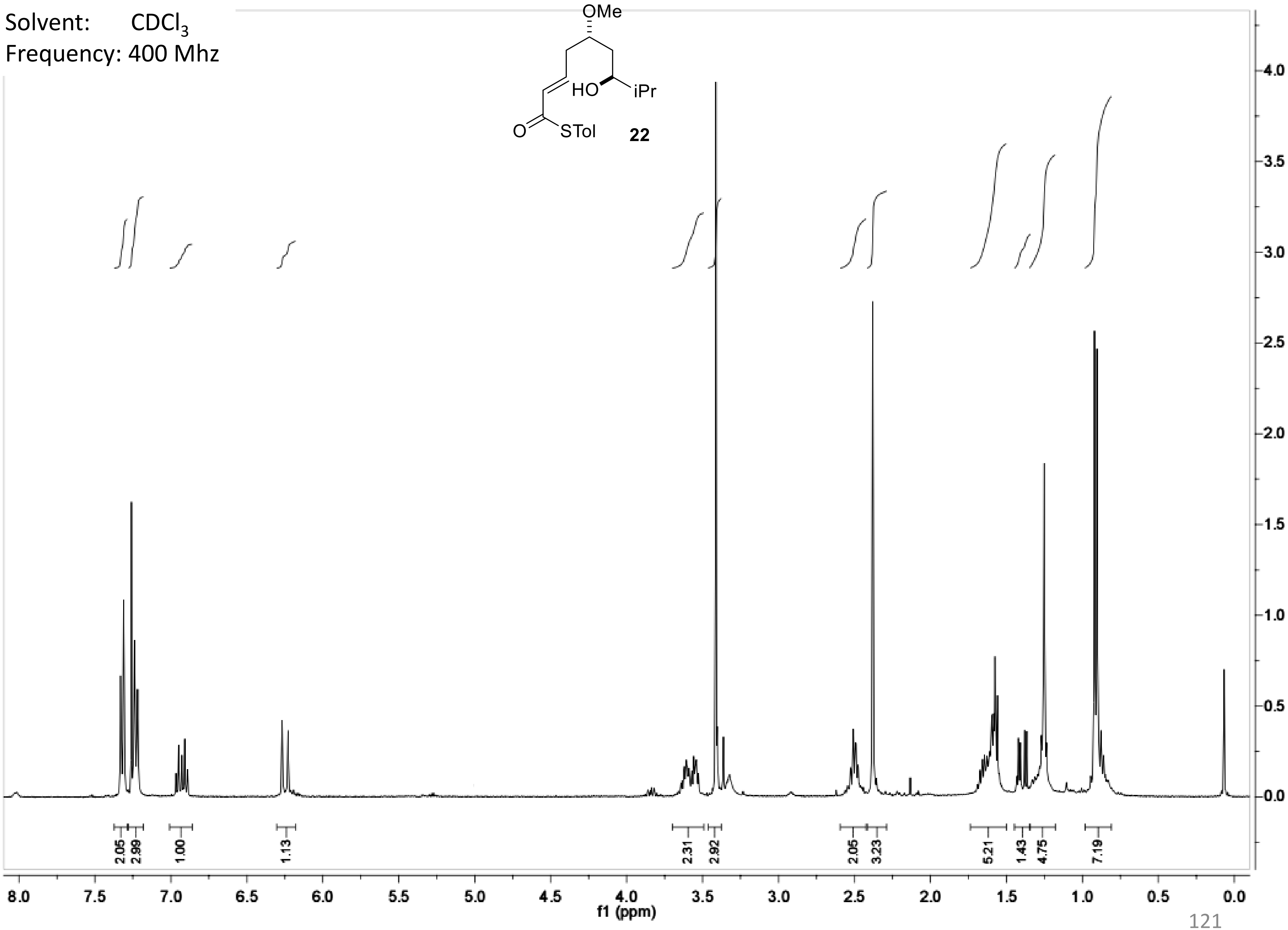

Solvent: $CDCl_3$
Frequency: 101 Mhz

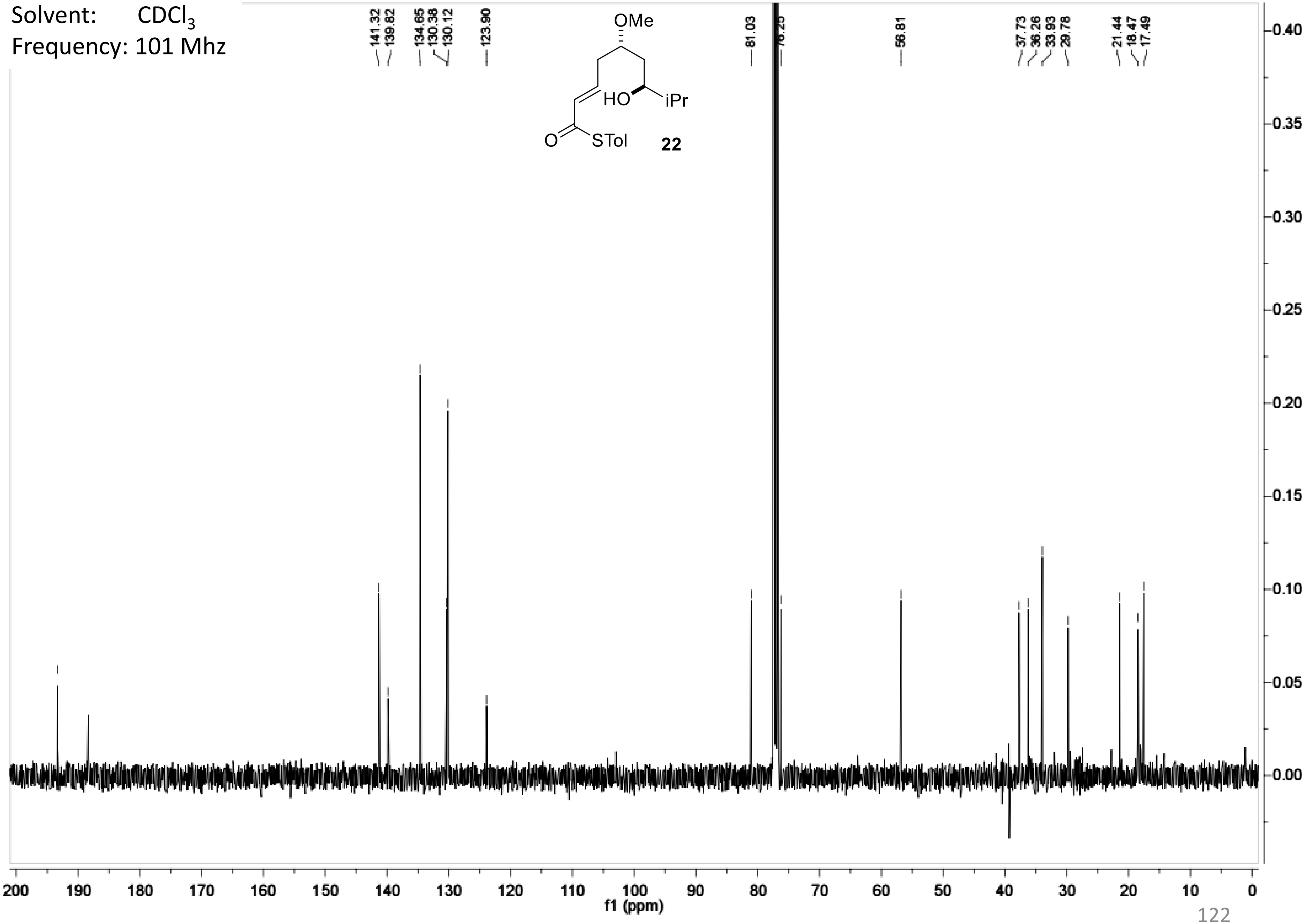

Solvent: $CDCl_3$
Frequency: 400 Mhz

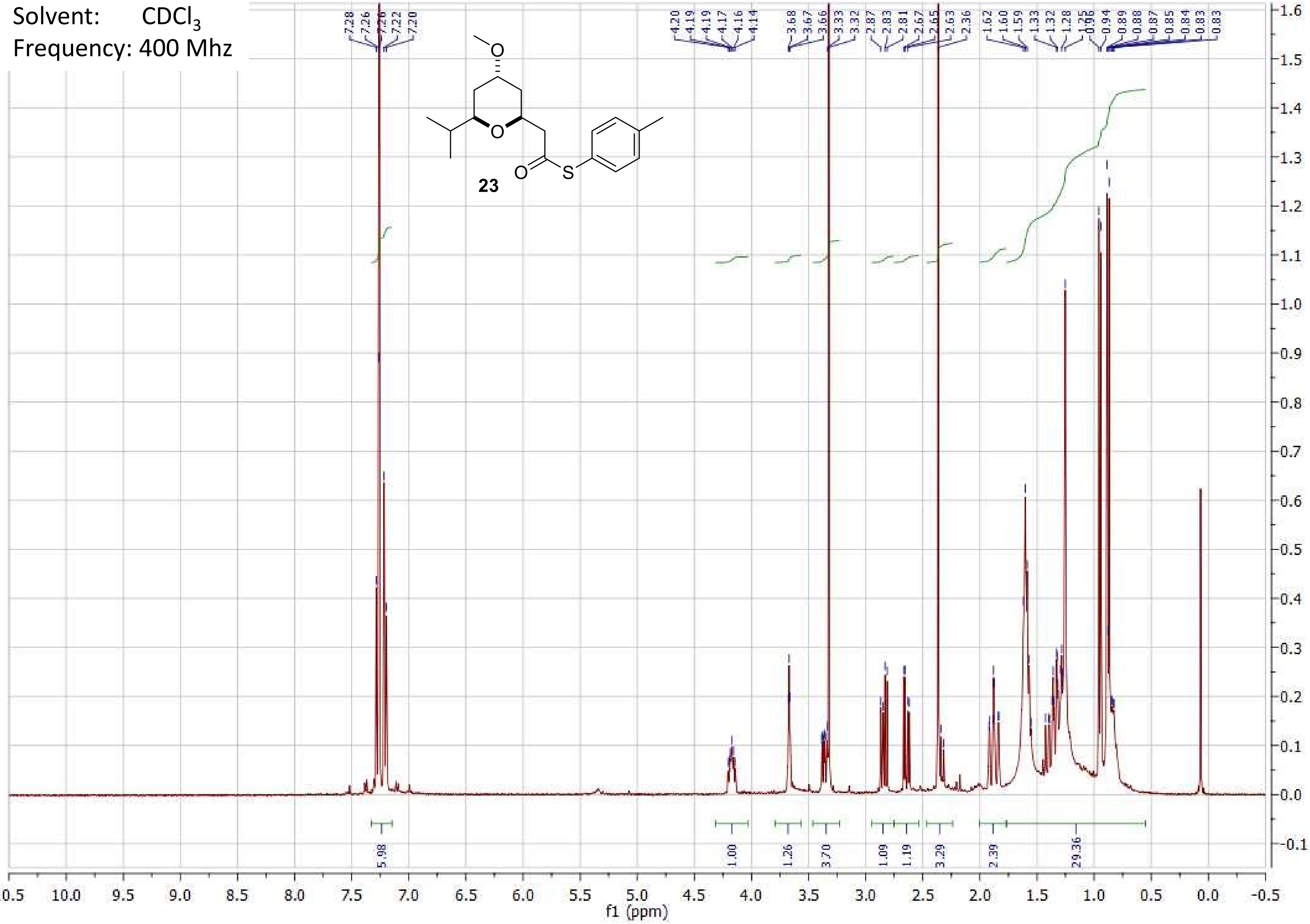

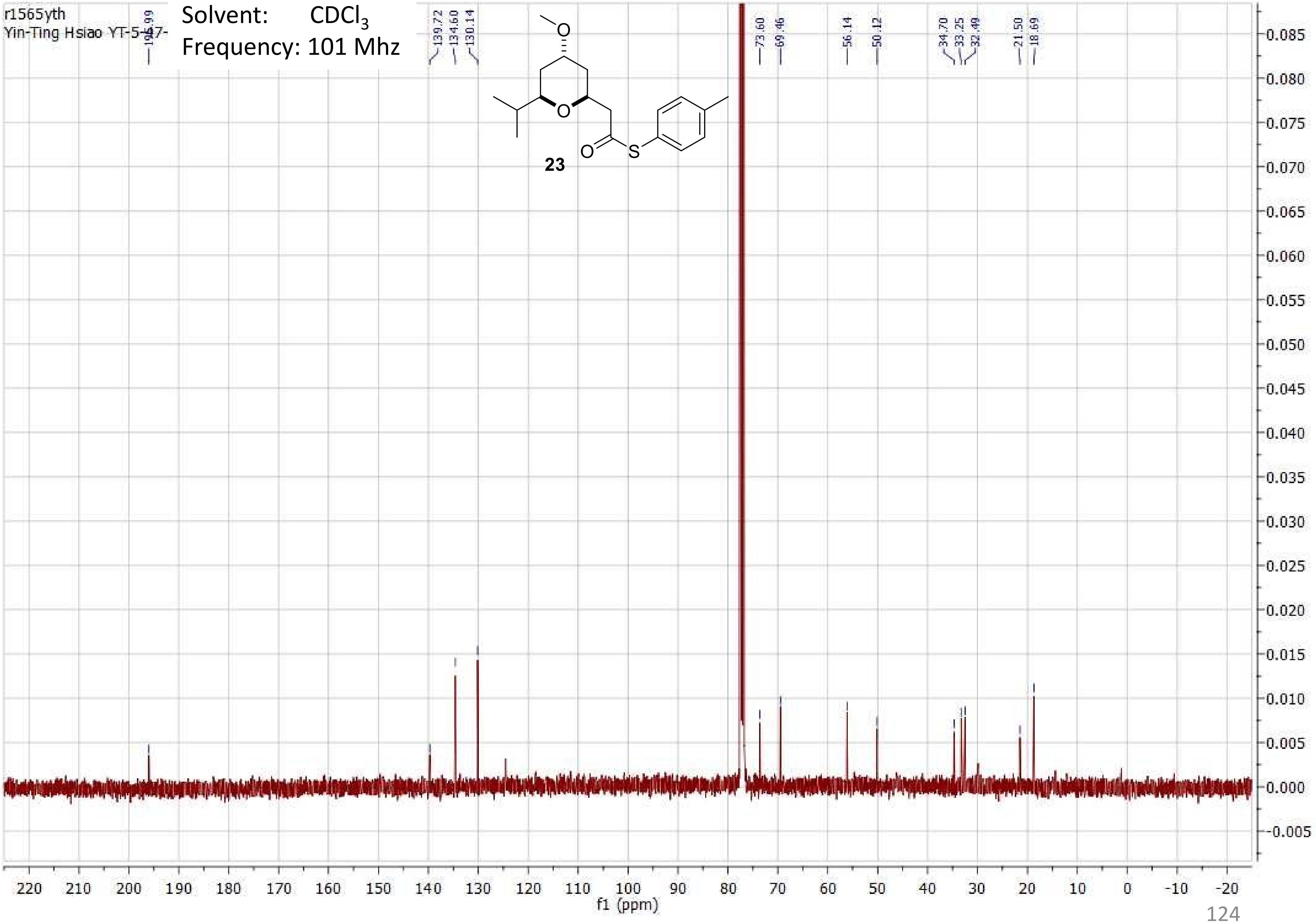
r1565yth
Solvent: CDCl3
Frequency: 101 Mhz
139.72
134.60
130.14
73.60
69.46
56.14
50.12
34.70
33.25
32.49
21.50
18.69
23
f1 (ppm)

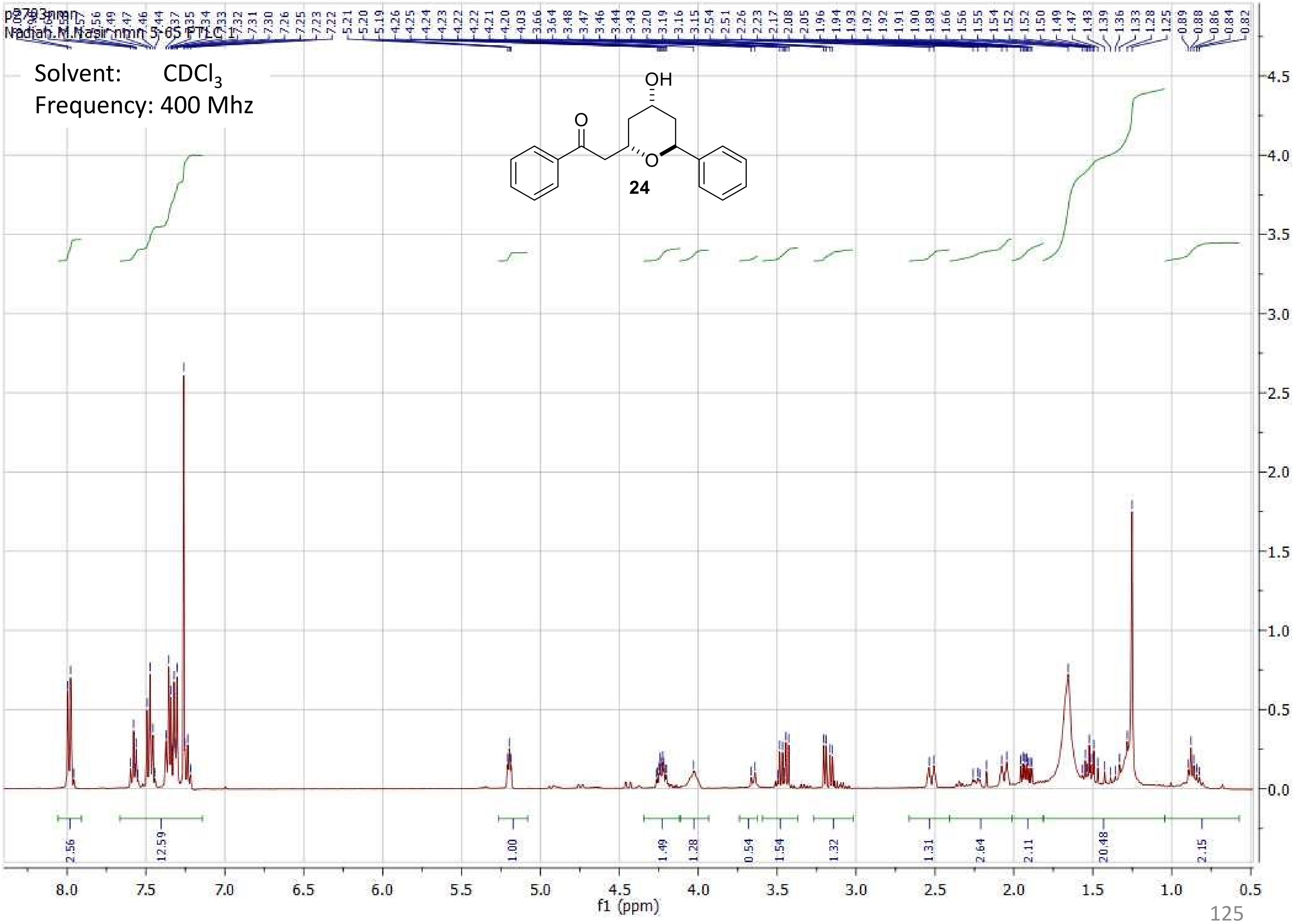
Solvent: CDCl3
Frequency: 400 Mhz
24
f1 (ppm)

Solvent: $CDCl_3$
Frequency: 101 Mhz

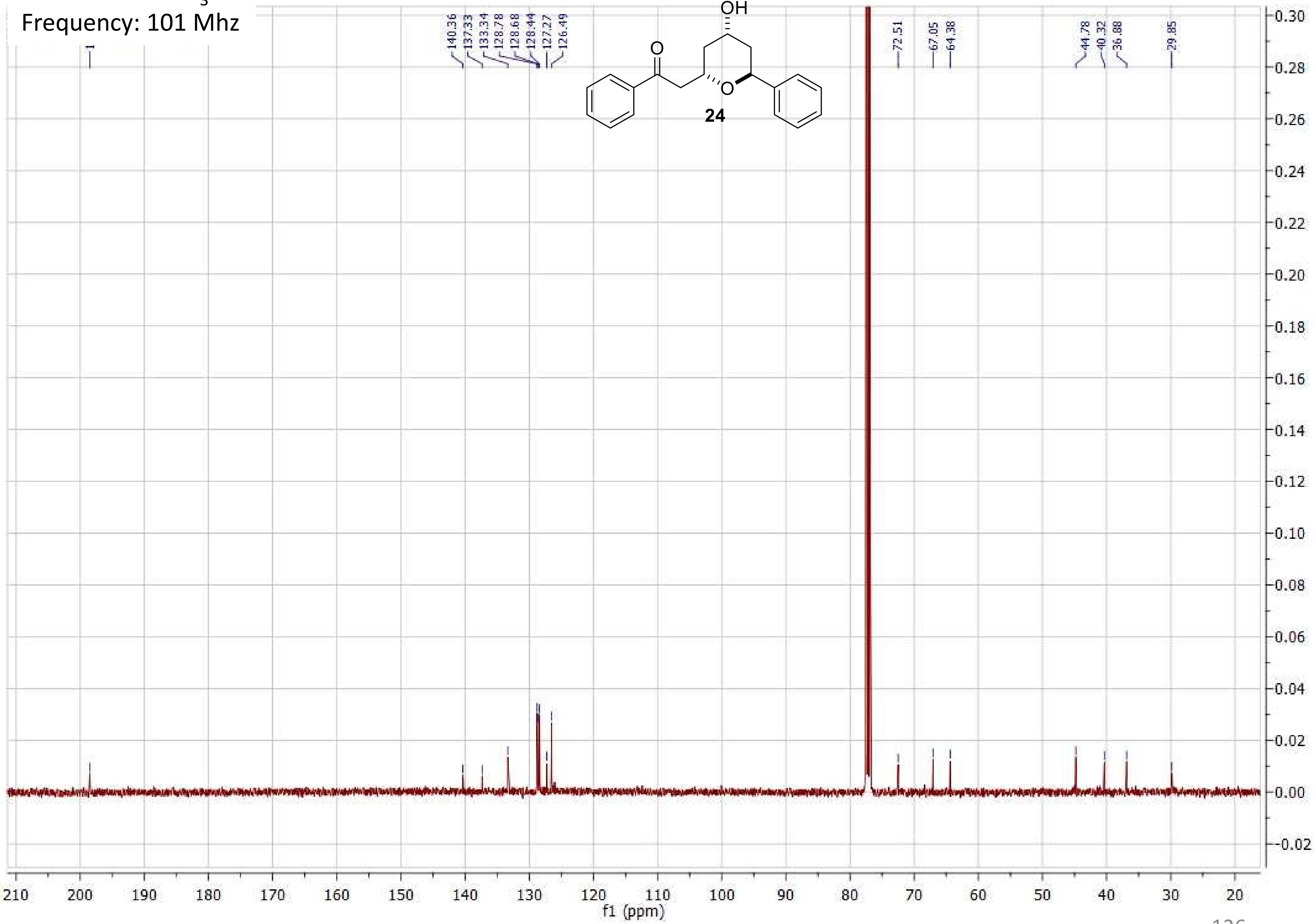

Solvent: $CDCl_3$

Frequency: 400 Mhz

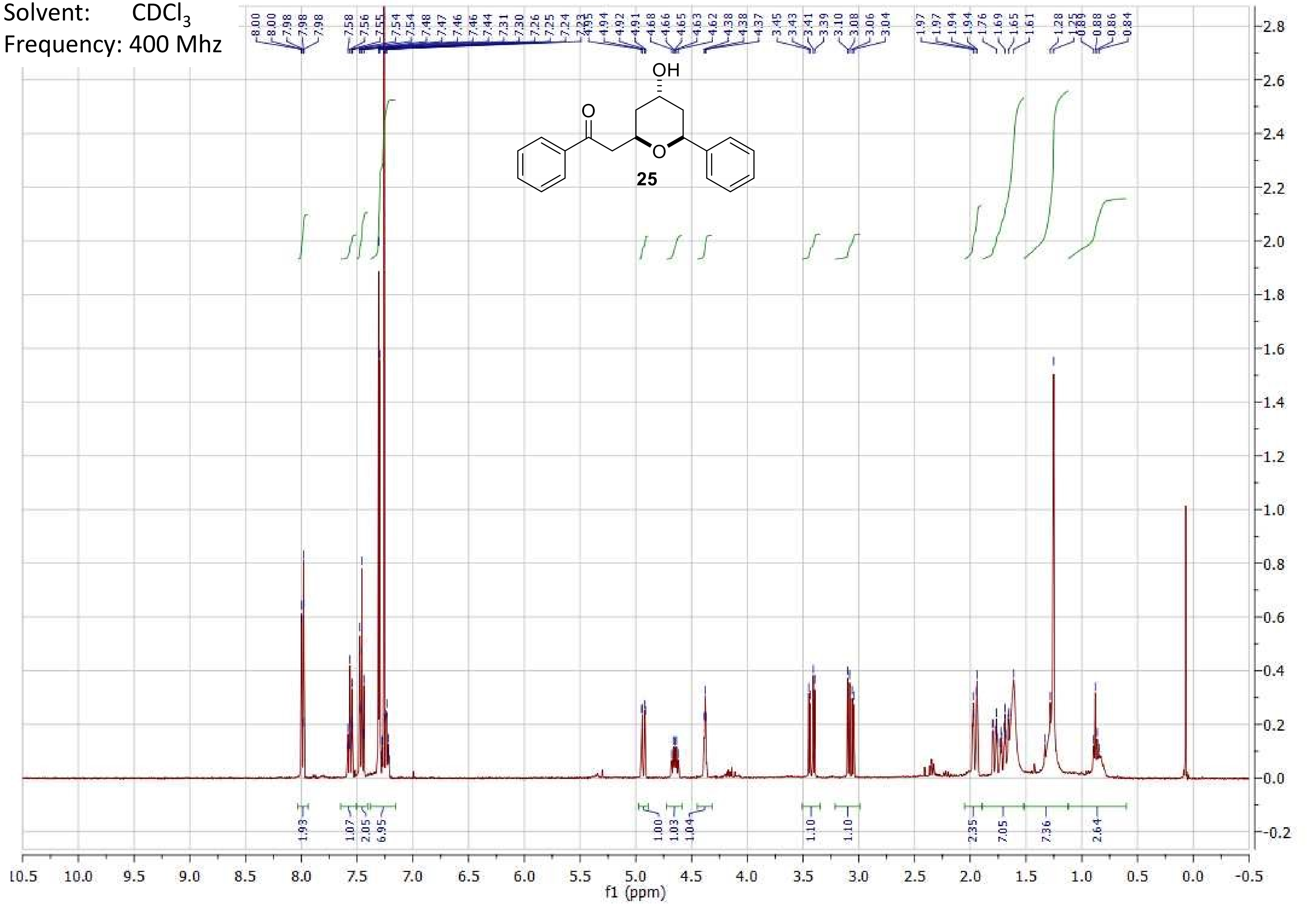

Solvent: $CDCl_3$

Frequency: 101 Mhz

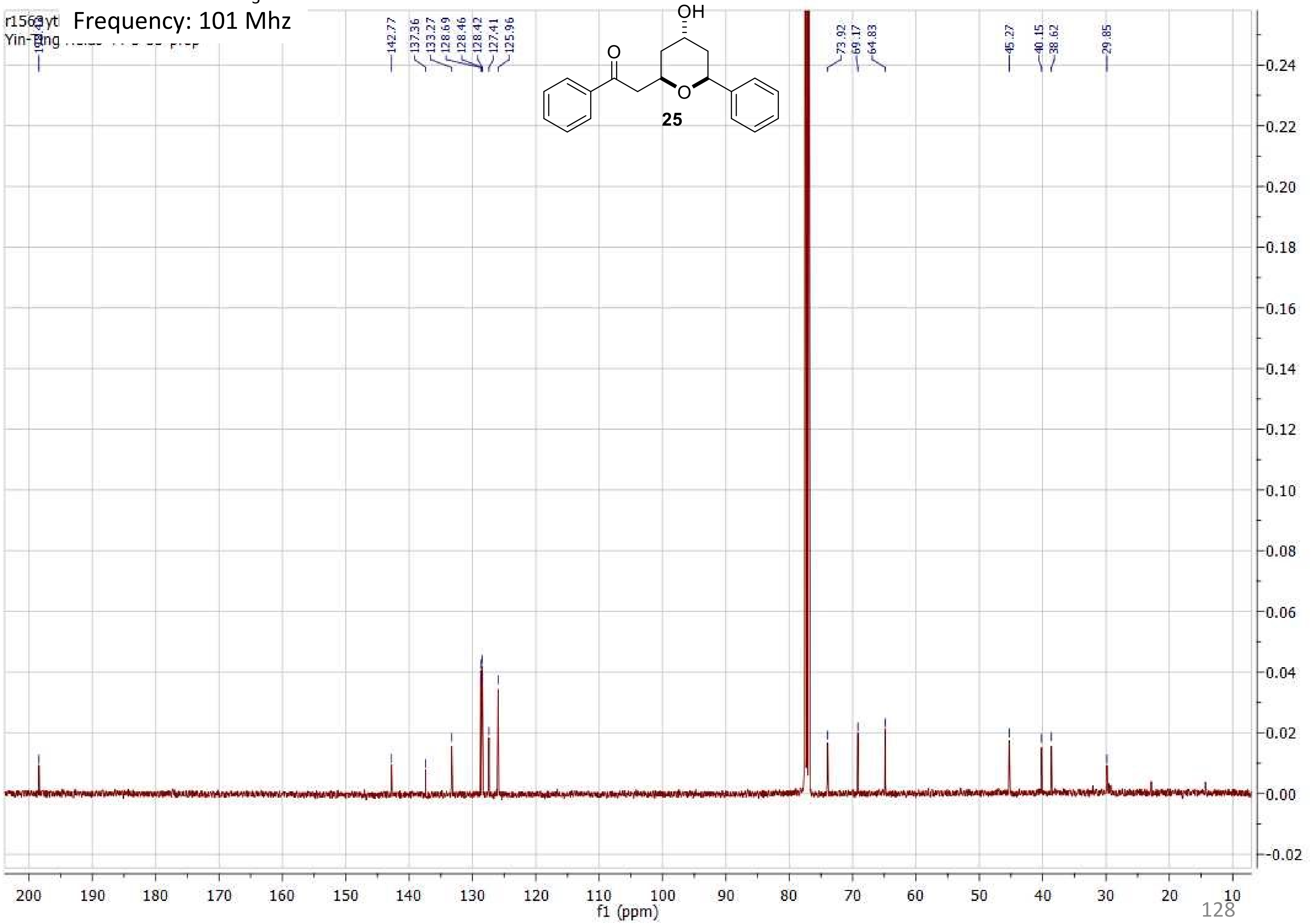

Supplement: Supplementary file 1 [file SC-008-C6SC03478K-s001.pdf]
